# Photocatalytic Enantioselective Radical Cascade Multicomponent Minisci Reaction of β‐Carbolines Using Diazo Compounds as Radical Precursors

**DOI:** 10.1002/advs.202402272

**Published:** 2024-04-19

**Authors:** Yi‐Jie Gu, Mu‐Peng Luo, Hua Yuan, Guo‐Kai Liu, Shou‐Guo Wang

**Affiliations:** ^1^ Shenzhen Institute of Advanced Technology Chinese Academy of Sciences Shenzhen Guangdong 518055 P. R. China; ^2^ Key Laboratory of Theoretical Organic Chemistry and Function Molecule of Ministry of Education School of Chemistry and Chemical Engineering Hunan University of Science and Technology Xiangtan 411201 P. R. China; ^3^ School of Pharmacy Shenzhen University Medical School Shenzhen University Shenzhen Guangdong 518055 P. R. China

**Keywords:** asymmetric photocatalysis, diazo compounds, Minisci reaction, multicomponent reaction, radical cascade

## Abstract

Here, a photocatalytic asymmetric multicomponent cascade Minisci reaction of β‐carbolines with enamides and diazo compounds is reported, enabling an effective enantioselective radical C─H functionalization of β‐carbolines with high yields and enantioselectivity (up to 83% yield and 95% ee). This enantioselective multicomponent Minisci protocol exhibits step economy, high chemo‐/enantio‐selective control, and good functional group tolerance, allowing access to a variety of valuable chiral β‐carbolines. Notably, diazo compounds are suitable radical precursors in enantioselective cascade radical reactions. Moreover, the efficiency and practicality of this approach are demonstrated by the asymmetric synthesis of bioactive compounds and natural products.

## Graphical abstract

The study presents a photocatalytic asymmetric cascade Minisci reaction of β‐carbolines with enamides and diazo compounds, allowing for effective enantioselective radical C─H functionalization of β‐carbolines with high yields and enantioselectivity (up to 83% yield and 95% ee). The effectiveness and viability of this strategy are demonstrated through the asymmetric synthesis of bioactive compounds and natural product.

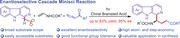

## 1 Introduction

**Figure 1:**
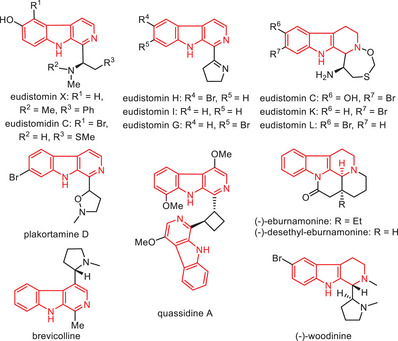
Examples of Alkaloids Containing β‐Carboline Core.

Chiral β‐carbolines and their derivatives are widely acknowledged as remarkable and privileged scaffolds in biologically active compounds, pharmaceuticals, and natural products (**Figure** **1**) ref. [1]. The asymmetric synthesis of chiral β‐carbolines has garnered significant attention due to their unique structural features, diverse potent biological activities, and their ability to access a broad range of bio‐important molecules.^[^ ^2^ ^]^ Traditionally, the asymmetric synthesis of β‐carbolines has primarily relied on the introduction of chirality through preinstalled chiral functional groups during the construction of the β‐carboline core. This strategy guides the stereochemical outcome through subsequent multistep transformations.^[^ ^3^ ^]^ In contrast to advances in the catalytic asymmetric synthesis of enantioenriched tetrahydro‐β‐carbolines,^[^ ^4^ ^]^ the direct enantioselective construction of chiral β‐carbolines using catalytic methods, particularly through the C─H functionalization process, has received comparatively less attention and remains a formidable challenge.^[^ ^5^ ^]^ The deficiency of effective catalytic systems and appropriate transformations that permit efficient enantioselective synthesis is the cause of this constraint.^[^ ^6^ ^]^

**Scheme 1:**
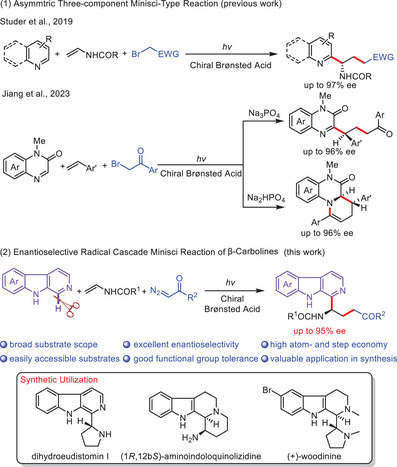
Photocatalytic Enantioselective Radical Cascade Multicomponent Minisci Reaction of β‐Carbolines.

The Minisci‐type reaction has emerged as a significant strategy for constructing complex molecules, demonstrating promising potential in the direct C─H functionalization of *N*‐ heteroarenes via radical addition to electrophilic *N*‐ heteroarenes.^[^ ^7^ ^]^ The recent advancements in catalytic asymmetric Minisci reactions make it accessible to realize the direct enantioselective synthesis of chiral β‐carbolines.^[^ ^8^, ^9^ ^]^ Catalytic asymmetric Minisci reactions employing cooperative photoredox and chiral Brønsted acid catalysis have been achieved with various electron‐deficient azaarenes, such as quinolines, isoquinolines, pyridines, and pyrimidines.^[^ ^10^ ^]^ In 2019, Studer and colleagues explored an enantioselective three‐component Minisci‐type reaction of quinolines or pyridines, enamides, and α‐bromo carbonyl compounds as radical precursors, enabling access to chiral functionalized quinoline or pyridine derivatives in a single synthetic operation.^[^ ^11^ ^]^ More recently, Jiang and co‐workers reported a photocatalyzed asymmetric three‐component Minisci‐type reaction of quinoxalin‐2(1*H*)‐ones, styrenes, and 2‐bromo‐1‐arylenthan‐1‐ones for the enantioselective chemodivergent synthesis of two series of valuable products (**Scheme** **1**).^[^ ^12^ ^]^ In these cascade radical transformations, the presence of bases is essential in the catalytic system to neutralize the generated HBr, thereby suppressing the background reaction and ensuring reliable asymmetric induction. These enantioselective cascade Minisci‐type reactions offer several advantages, including increased efficiency, rapid assembly of molecular diversity, and the versatile ability to access complex structures that may be challenging to synthesize through stepwise approaches.^[^ ^13^ ^]^ However, the development of enantioselective multicomponent Minisci reactions remains in its infancy. It is important to note that such asymmetric cascade Minisci reactions present ongoing challenges, requiring compatible reactivity of each component and precise control over chemo‐/stereochemistry at each step. Considering the significance of chiral β‐carboline derivatives, we envision a catalytic asymmetric multicomponent Minisci reaction of β‐carbolines utilizing diazo compounds as the radical precursors, providing an effective, and straightforward pathway to construct valuable highly‐functionalized chiral β‐carboline products (Scheme 1). However, several challenges need to be circumvented in this enantioselective cascade multicomponent Minisci process, including the reduced reactivity of β‐carbolines due to their inherently more electron‐rich nature,^[^ ^5^, ^14^ ^]^ the highly unstable and easily decomposing nature of diazo compounds,^[^ ^15^ ^]^ and the possible competitive side reactions involving enamides, and diazo compounds.^[^ ^16^ ^]^ This strategy has been successfully applied in the highly efficient asymmetric synthesis of bioactive chemicals and natural products, demonstrating its effectiveness, and viability.

## 2 Results and Discussion

### 2.1 Optimization of Reaction Conditions

Our investigation began with an examination of the asymmetric tandem radical reaction involving β‐carboline **1a**, *N*‐vinylacetamide **2a**, and ethyl bromoacetate as the radical precursor. A cooperative catalytic system combining photoredox catalyst and chiral phosphoric acid (**CPA**) was employed to explore the asymmetric cascade Minisci reaction. With extensive evaluation of various reaction conditions, including catalysts, bases, solvent, temperature, and the influence of visible light, the best result was obtained with desired product in 65% yield and 67% enantiomeric excess under the cooperative catalysis of [Ir(dF(CF_3_)ppy)_2_(dtbpy)]PF_6_ and chiral Brønsted acid (*R*)‐**CPA‐1**, using Na_3_PO_4_ as the base and tert‐butyl methyl ether as the solvent at −40 °C (for more details, see supporting information). We speculated that the racemic background reaction facilitated by in situ generated HBr could not be effectively inhibited in the catalytic system, resulting in moderate enantioselective control.^[^ ^11^, ^12^ ^]^ Inspired by Meggers^[^ ^17^ ^]^ and Doyle's^[^ ^18^ ^]^ work, we designed a novel cascade asymmetric Minisci reaction utilizing diazo compounds as carbon radical precursors to achieve asymmetric radical C─H functionalization of β‐carboline (**Table** **1**). In this process, the diazo compounds are converted to electrophilic carbon radical species through proton‐coupled electron transfer (PCET), while N_2_ is released as a byproduct, excluding its influence on the background reaction.^[^ ^18^ ^]^ We then investigated the three‐component asymmetric Minisci reaction using the model reaction of diazo compound **3a**, *N*‐vinylacetamide **2a**, and β‐carboline **1a**, with 4 Å molecular sieve as an additive in dioxane at 10 °C under blue light‐emitting diode (LED) irradiation, using [Ir(dF(CF_3_)ppy)_2_(dtbpy)]PF_6_ as a photocatalyst and 5 mol.% of chiral Brønsted acid catalyst. After examining various chiral Brønsted acid catalysts, chiral phosphoric acid (*R*)‐**CPA‐1** was found to be optimal in terms of yield and enantioinduction, giving the target product with 62% yield, and 33% ee. Notably, the chiral Brønsted acid catalyst plays several important roles, including protonating the diazo compound to generate a radical intermediate,^[^ ^18b^ ^]^ enhancing the reactivity of β‐carbolines, and controlling the stereochemistry of the key bond‐forming steps during the cascade sequence.^[^ ^9^, ^11^, ^19^ ^]^ To our delight, investigations into the solvent effect indicated that THF exhibited a promising improvement in enantioselectivity, albeit with a slightly lower yield (52% yield and 63% ee). Increasing the catalyst loading of (*R*)‐**CPA‐1** to 10 mol.% further improved the enantioselectivity to 70% ee. Gratifyingly, when one equivalent of diazo compound **3a** was added every 12 h and the reaction was carried out at −40 °C, a dramatic increase in enantioselective control was obtained with a high yield (87% yield, 84% ee). We were pleased to find that setting up the reaction in a more dilute solution enhanced the asymmetric induction successfully, delivering the target product with a good yield, and impressive enantioselectivity (80% yield, 92% ee). Comparatively, adding 3.0 equivalent of diazo compound **3a** at one time at −40 °C gave a diminished outcome (71% yield, 89% ee). Given the unstable and highly reactive nature of diazo compounds, we reasoned that introducing diazo compounds in portions could mitigate undesirable side reactions.^[^ ^18b^ ^]^

**Table 1:** Optimization of reaction conditions.

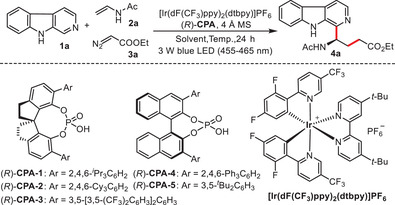					
Entry ^a)^	Solvent [mL]	Catalyst [mol.%]	T [^o^C]	Yield [%]^b)^	ee [%]^c)^
1	Dioxane (2.0)	(*R*)‐**CPA‐1** (5)	10	62	33
2	Dioxane (2.0)	(*R*)‐**CPA‐2** (5)	10	53	15
3	Dioxane (2.0)	(*R*)‐**CPA‐3** (5)	10	51	21
4	Dioxane (2.0)	(*R*)‐**CPA‐4** (5)	10	63	18
5	Dioxane (2.0)	(*R*)‐**CPA‐5** (5)	10	56	11
6	CH_2_Cl_2_ (2.0)	(*R*)‐**CPA‐1** (5)	10	68	29
7	THF (2.0)	(*R*)‐**CPA‐1** (5)	10	52	63
8	DME(2.0)	(*R*)‐**CPA‐1** (5)	10	57	58
9	EtOAc (2.0)	(*R*)‐**CPA‐1** (5)	10	64	21
10	THF (2.0)	(*R*)‐**CPA‐1** (10)	10	56	70
11^d)^	THF (2.0)	(*R*)‐**CPA‐1** (10)	−40	87	84
12^d)^	THF (4.0)	(*R*)‐**CPA‐1** (10)	−40	86	87
13^d)^	THF (8.0)	(*R*)‐CPA‐1 (10)	−40	80	92
14^e)^	THF (8.0)	(*R*)‐**CPA‐1** (10)	−40	71	89

^a)^ Reaction conditions: **1a** (0.1 mmol.), **2a** (0.2 mmol.), **3a** (0.3 mmol.), [Ir(dF(CF_3_)ppy)_2_(dtbpy)]PF_6_ (2 mol.%), 4 Å MS (25 mg), Ar, 24 h;

^b)^ Yield of isolated product;

^c)^ Determined by HPLC analysis on a chiral stationary phase;

^d)^ 48 h, **3a** was added every 12 h, three times in total;

^e)^ 48 h. 4 Å MS = 4 Å molecular sieve; THF = tetrahydrofuran; DME = 1,2‐dimethoxyethane.

### 2.2 Asymmetric Cascade Minisci Reaction of β‐Carbolines with Enamides and Diazo Compounds

**Scheme 2:**
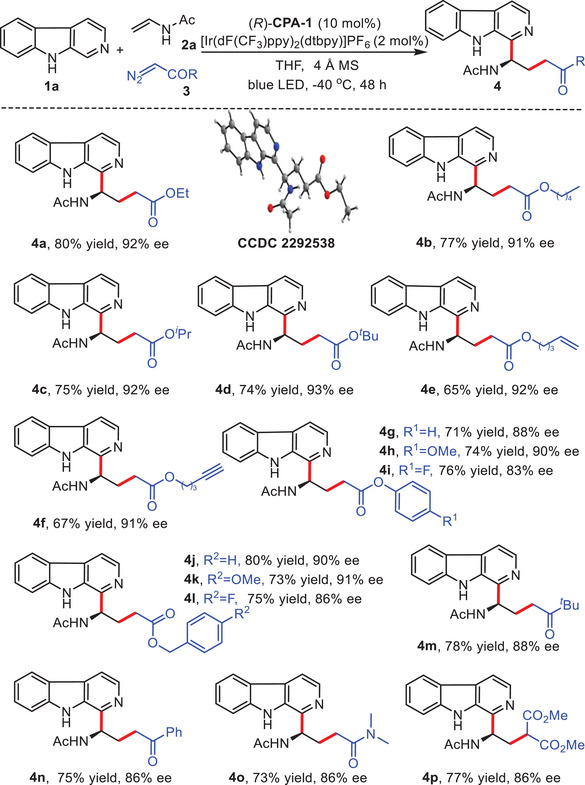
Scope of diazocarbonyl compounds. Reaction conditions: **1a** (0.1 mmol.), **2a** (0.2 mmol.), **3** (0.3 mmol.), [Ir(dF(CF_3_)ppy)_2_(dtbpy)]PF_6_ (2 mol.%), (*R*)‐**CPA‐1** (10 mol.%), 4 Å MS (25 mg), THF (8 mL), 3 W blue LED, −40 °C, Ar, 48 h, **3** was added every 12 h, and 3 times in total.

With the optimized reaction conditions, we proceeded to evaluate diazocarbonyl compounds in the enantioselective cascade Minisci reactions (**Scheme** **2**). Various diazo carbonyl compounds, including α‐diazo esters, α‐diazo ketones, α‐diazo amides, and dimethyl diazomalonate, were investigated to enable the rapid construction of complex chiral β‐carbolines. The transformation successfully accommodated α‐diazo esters with different substituents, yielding the corresponding chiral β‐ carboline products with consistently good to excellent yields, and enantioselectivities (**4a–4l**, 65–80% yields, and 83–93% ee). Encouragingly, α‐diazo esters bearing alkenyl or alkynyl groups were well‐tolerated in this process, exhibiting no detrimental effects on catalytic efficiency or enantioselectivities (**4e,4f**, 65–67% yields, and 91–92% ee). The electronic effects of aryl or benzyl groups in α‐diazo esters had little influence on the performance of the multicomponent asymmetric Minisci reaction (**4g–4l**, 71–80% yields, and 83–91% ee). Furthermore, alkyl or aryl‐substituted α‐diazo ketones proved to be suitable radical precursors for the asymmetric cascade Minisci reaction, delivering the target products **4m** and **4n** with high yields and enantioinduction (75–78% yields, 86–88% ee). The reaction also exhibited favorable performance with α‐diazo amide, providing the desired product **4o** in 73% yield, albeit with a slightly decreased enantioselectivity (86% ee). Moreover, dimethyl diazomalonate was shown to be suitable for this transformation, delivering the target product **4p** with 77% yield and 86% ee. The absolute configuration of product **4a** was determined as *R* through X‐ray crystallographic analysis.^[^ ^20^ ^]^

**Scheme 3:**
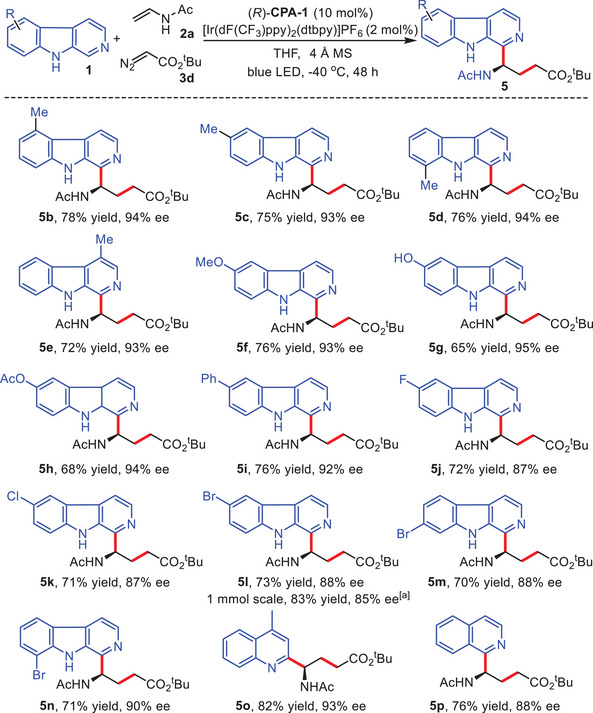
Scope of β‐carbolines. Reaction conditions: **1** (0.1 mmol.), **2a** (0.2 mmol.), **3d** (0.3 mmol.), [Ir(dF(CF_3_)ppy)_2_(dtbpy)]PF_6_ (2 mol.%), (*R*)‐**CPA‐1** (10 mol.%), 4 Å MS (25 mg), THF (8 mL), 3 W blue LED, −40 °C, Ar, 48 h, **3d** was added every 12 h, and 3 times in total. ^a)^ **1l** (1.0 mmol.), **2a** (2.0 mmol.), **3d** (3.0 mmol.), [Ir(dF(CF_3_)ppy)_2_(dtbpy)]PF_6_ (2 mol.%), (*R*)‐**CPA‐1** (10 mol.%), 4 Å MS (250 mg), THF (80 mL), 3 W blue LED, −40 °C, Ar, 48 h, **3d** was added every 12 h, and three times in total.

Regarding the significance of the β‐carboline core, we investigated the compatibility of various β‐carbolines with *N*‐vinylacetamide **2a** and tert‐butyl 2‐diazoacetate **3d** in the cascade asymmetric Minisci protocol (**Scheme** **3**). Remarkably, the catalytic system exhibited wide tolerance toward a diverse array of substituted β‐carbolines, accommodating a broad range of electronic, and steric properties. When employing different methyl‐substituted carbolines, the cascade Minisci reactions were all accomplished with good yields and excellent enantioselectivities (**5b–5e**, 72–78% yields, and 93–94% ee). Furthermore, other β‐carbolines with electron‐rich groups proved suitable for the asymmetric multicomponent Minisci transformation, providing decent yields, and excellent enantioselectivities (**5f–5h**, 65–76% yields, and 93–95% ee). Notably, β‐carboline **1g**, bearing a free phenolic hydroxyl group, was well‐tolerated to generate the target product **5g** smoothly without influencing the reaction efficiency and enantiocontrol (65% yield, 95% ee). The aryl‐substituted β‐carboline was found to be compatible in the transformation, resulting in the target product **5i** with 76% yield, and 92% ee. The introduction of electron‐deficient functional groups on the β‐carboline core displayed a slight impact on enantioselectivity, affording the chiral β‐carboline products **5j–5n** with good yields and slightly lower enantioselectivities (70–73% yields, 87–90% ee). Notably, the asymmetric cascade Minisci reaction of β‐carboline **1l** could be conducted on 1 mmol. scale, providing the desired product **5l** successfully in 83% yield, and 85% ee. Gratifyingly, this approach also exhibited good compatibility with 4‐methyl quinoline and isoquinoline substrates, delivering corresponding target products **5o** ^[^ ^11^ ^]^ and **5p** with high yields and enantioinduction (76–82% yield, 88–92% ee). However, 9‐methyl‐β‐carboline **1q** was not suitable for this transformation.

**Scheme 4:**
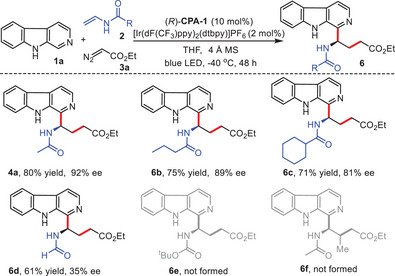
Scope of enamides. Reaction conditions: 1a (0.1 mmol.), 2 (0.2 mmol.), 3a (0.3 mmol.), [Ir(dF(CF_3_)ppy)_2_(dtbpy)]PF_6_ (2 mol.%), (*R*)‐CPA‐1 (10 mol.%), 4 Å MS (25 mg), THF (8 mL), 3 W blue LED, −40 °C, Ar, 48 h, 3a was added every 12 h, and three times in total.

We proceeded to investigate the performance of various substituted *N*‐vinylacetamides in conjunction with β‐carboline **1a** and α‐diazo ester **3a** (**Scheme** **4**). Other substituted *N*‐vinylacetamides, such as *N*‐vinylbutyramide and *N*‐vinylcyclohexanecarboxamide, were demonstrated to be compatible in the transformation, accomplishing the enantioselective cascade Minisci reaction with good yields, and enantioselectivities (**6b,6c**, 71–75% yields, and 81–89% ee). *N*‐vinylformamide could also be employed in this transformation to generate the desired product **6d** with reasonable yield and decreased enantioselectivity (61% yield, 35% ee), which might be dictated by the steric effect. However, tert‐butyl vinylcarbamate **2e** exhibited significantly reduced reactivity, likely due to its electronic properties. Substituted *N*‐vinylacetamide (*E*)‐*N*‐(prop‐1‐en‐1‐yl)acetamide **2f** was demonstrated to be not suitable for this transformation and might be affected by the steric hindrance (for more details, see supporting information).

### 2.3 Derivatizations and Applications

**Scheme 5:**
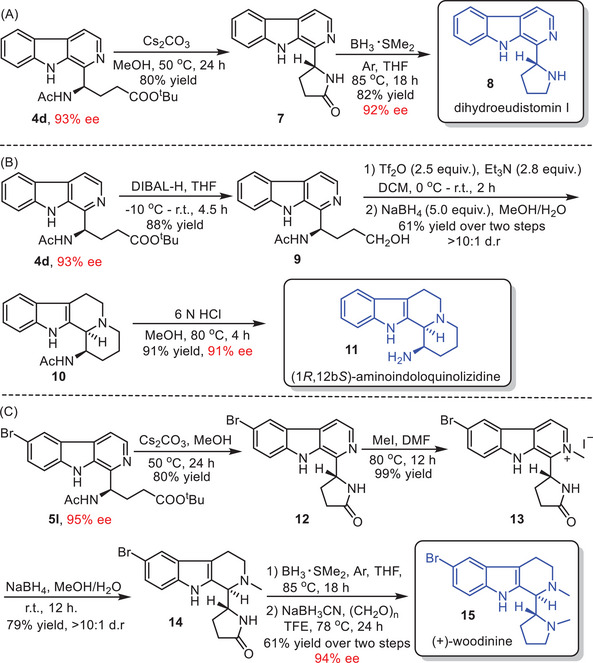
Transformations of chiral β‐carboline products. DIBAL‐H = diisobutylaluminium hydride; DCM = dichloromethane; DMF = *N*, *N*‐dimethylformamide; TFE = 2,2,2‐trifluoroethanol.

Chiral β‐carbolines are highly versatile and valuable scaffolds, offering the capacity to undergo a wide range of transformations for the construction of natural products and bioactive compounds with diverse biological properties.^[^ ^1^, ^21^ ^]^ To showcase the potential applications of the enantioselective multicomponent cascade Minisci reaction of β‐carbolines, various transformations of the chiral β‐carboline products were conducted. Starting with chiral β‐carboline product **4d** (93% ee), an intramolecular lactamization was performed under basic conditions, resulting in chiral γ‐lactam product **7**. Subsequently, γ‐lactam **7** could be further reduced to dihydroeudistomin I **8** (82% yield, 92% ee) with complete retention of enantiomeric purity (**Scheme** **5**A). Importantly, dihydroeudistomin I **8** could be easily transformed into alkaloid eudistomin I through treatment with *N*‐chlorosuccinimide.^[^ ^22^ ^]^ Reduction of the chiral β‐carboline product **4d** (93% ee) with DIBAL‐H yielded the corresponding alcohol **9**, which underwent triflate formation, intermolecular annulation, and reduction of *N*‐alkyl pyridinium salt sequence to provide a four‐membered heterocyclic tetrahydro‐β‐carboline **10**. Subsequent removal of the acetyl group from **10** under acidic conditions led to the formation of 1‐aminoindolo[2,3‐*a*]quinolizidine product **11** (91% ee) without erosion of the ee value (Scheme 5B). This intermediate could be employed for the convenient construction of pharmacologically interesting *E*‐azaburnane‐type compounds.^[^ ^23^ ^]^ Chiral β‐carboline product **5l** (95% ee) underwent intramolecular lactamization to form the γ‐lactam compound **12**, followed by selective methylation to deliver the corresponding *N*‐Methyl β‐carboline salt **13** with 79% yield over two steps. Subsequently, compound **13** underwent reduction using NaBH_4_, affording the corresponding tetrahydro‐β‐carboline **14** with good yield and diastereoselectivity (79% yield, >10:1 dr). Further reduction of **14** led to the formation of a pyrrolidine product. At this stage, reductive *N*‐methylation of the pyrrolidine intermediate was performed, accomplishing the asymmetric total synthesis of alkaloid (+)‐woodinine **15** (94% ee) successfully (Scheme 5C).^[^ ^2^, ^24^ ^]^ The above‐described transformations highlight the versatility and synthetic potential of this enantioselective multicomponent Minisci protocol.

### 2.4 Reaction Insights

**Scheme 6:**
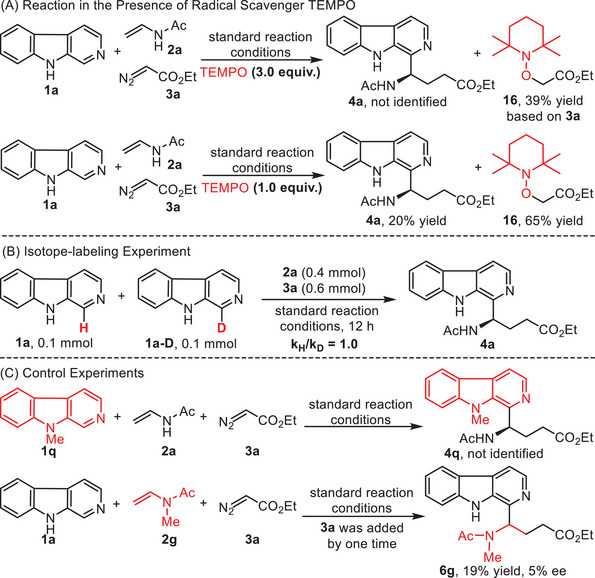
Mechanistic studies. A) Reaction in the presence of radical scavenger TEMPO. B) Isotope‐labeling experiment. C) Control experiments. TEMPO = 2,2,6,6‐tetramethylpiperidinooxy.

To get insights into the mechanism of this enantioselective multicomponent Minisci reaction, several control experiments were performed (**Scheme** **6**). When the reaction was treated with 3.0 equivalents of TEMPO, the cascade Minisci reaction was completely suppressed without forming the target product **4a**, instead generating the radical‐trapping product **16** with a yield of 39% based on diazo compound **3a**.^[^ ^18b^ ^]^ Reducing the amount of TEMPO to 1.0 equivalent produced the target product **4a** with a decreased yield from 80% to 20% and compound **16** with 65% yield (Scheme 6A). A competition experiment between **1a** and **1a‐D** was conducted to give a primary kinetic isotope effect (KIE) of 1.0, suggesting the deprotonation step might not be involved in the rate‐determining step (Scheme 6B).^[^ ^11^, ^18^, ^25^ ^]^ As mentioned above, 9‐methyl‐β‐carboline **1q** was demonstrated to be unreactive in this transformation, which might arise from the steric hindrance of substituents. Furthermore, the replacement of *N*‐vinylacetamide **2a** with *N*‐methyl‐*N*‐vinylacetamide **2g** in the reaction provided the corresponding product **6g** with 19% yield and negligible enantioselectivity, indicating that the hydrogen bonding from vinylacetamide plays an essential role in the reaction activity and enantioselective control (Scheme 6C).^[^ ^11^ ^]^

**Scheme 7:**
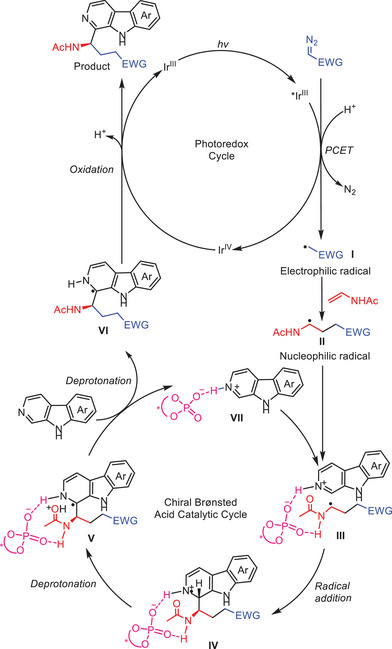
Proposed mechanism for asymmetric cascade multicomponent Minisci reaction of β‐carboline.

Based on the above results and previous research,^[^ ^9^, ^11^, ^18^ ^]^ we proposed the catalytic cycle described in **Scheme** **7**. Upon exposure to blue light irradiation, the Ir(III) complex undergoes photoexcitation and generates a photoexcited *Ir(III) species, which engages in a reduction reaction of the diazo compound through a proton‐coupled electron transfer (PCET) process to generate an oxidized iridium Ir(IV) species and a radical intermediate **I**.^[^ ^26^ ^]^ The generated α‐carbonyl radical species **I** then undergoes intermolecular electrophilic radical addition with the enamide, leading to the formation of nucleophilic radical **II**. Through a bidentate binding process aided by the chiral Brønsted acid catalyst, the α‐aminoalkyl radical **II** engages in a Minisci‐type addition to the protonated β‐carboline **VII**, giving rise to radical cation species **IV**. The radical cation **IV** is transformed into species **V** through an intramolecular deprotonation process assisted by the *N*‐acetyl group.^[^ ^19^ ^]^ Following additional deprotonation by an external β‐carboline molecule, the protonated intermediate **V** produces the protonated β‐carboline **VII**, and radical species **VI**.^[^ ^19^ ^]^ The radical species **VI** is oxidized by the Ir(IV) complex, followed by deprotonation, providing the target chiral β‐carboline product, and renewing the photosensitizer Ir(III) catalyst.

## 3 Conclusion 

In summary, we have demonstrated a highly enantioselective radical cascade multicomponent Minisci reaction of β‐carbolines using diazo compounds as radical precursors, enabling straightforward access to a wide range of valuable highly‐functionalized chiral β‐carbolines with good yields and high enantioselectivities (up to 83% yield and 95% ee). The cascade asymmetric Minisci protocol features its step‐economy, high efficiency, and wide functional group compatibility. Noteworthy, the diazo compound has been illustrated to be an effective radical precursor in enantioselective multicomponent radical reactions. The efficiency and practicality of this approach have been demonstrated by its successful application in the asymmetric syntheses of bioactive compounds and natural product. Further exploring and leveraging the potential of valuable catalytic asymmetric cascade Minisci‐type reactions is ongoing research in our lab.

## Conflict of Interest

The authors declare no conflict of interest.

## Supporting information

Supporting Information

## Data Availability

The data that support the findings of this study are available in the supplementary material of this article.
