# Supplementary material for: Photocatalytic Enantioselective Radical Cascade Multicomponent Minisci Reaction of β‐Carbolines Using Diazo Compounds as Radical Precursors
Source: Adv Sci (Weinh). 2024 Apr 19;11(25):2402272. doi: 10.1002/advs.202402272 (PMC11220658; doi:10.1002/advs.202402272)

ADVANCED SCIENCE
Open Access

# Supporting Information

for *Adv. Sci.*, DOI 10.1002/advs.202402272

Photocatalytic Enantioselective Radical Cascade Multicomponent Minisci Reaction of $\beta$-Carbolines Using Diazo Compounds as Radical Precursors

*Yi-Jie Gu, Mu-Peng Luo, Hua Yuan*, Guo-Kai Liu and Shou-Guo Wang**

*Supporting Information for*:

# Photocatalytic Enantioselective Radical Cascade Multicomponent Minisci Reaction of β-Carbolines Using Diazo Compounds as Radical Precursors

Yi-Jie Gu,‡[a,b] Mu-Peng Luo,‡[a] Hua Yuan,*[b] Guo-Kai Liu,[c] and Shou-Guo Wang*[a]

[a] Y.-J. Gu, Dr. M.-P. Luo, Prof. Dr. S.-G. Wang
Shenzhen Institute of Advanced Technology, Chinese Academy of Sciences, Shenzhen 518055, Guangdong, P. R. China. Email: shouguo.wang@siat.ac.cn

[b] Y.-J. Gu, Prof. Dr. H Yuan
Key Laboratory of Theoretical Organic Chemistry and Function Molecule of Ministry of Education, School of Chemistry and Chemical Engineering, Hunan University of Science and Technology, Xiangtan 411201, P. R. China. E-mail: hyuan@hnust.edu.cn

[c] Prof. Dr. G.-K. Liu
School of Pharmacy, Shenzhen University Medical School, Shenzhen University, Shenzhen 518055, Guangdong, P. R. China

‡ These authors contributed equally.

## Table of Contents

1 **General information** ........ 2
2 **Procedure for the preparation of starting materials** ........ 3
2.1 **General procedure for the preparation of β-Carbolines**[1-4] ........ 3
2.2 **General procedure for the preparation of diazocarbonyl compounds**[5] ........ 4
2.3 **Synthesis of *N*-(prop-1-en-1-yl)acetamide 2f**[6] ........ 5
2.4 **Synthesis of 1-deuterated β-Carboline 1a-D**[7-8] ........ 6
3 **Optimization of reaction conditions** ........ 8
4 **General Procedure for the Photocatalytic Enantioselective Radical Cascade Multicomponent Minisci Reaction of β-Carbolines Using Diazo Compounds as Radical Precursors** ........ 11
5 **Scale-up reaction** ........ 12
6 **Reaction with non-terminal alkene substrates** ........ 12
7 **Total synthesis of (*R*)-dihydroeudistomin I from 4d**[9-10] ........ 13
8 **Total synthesis of (1*R*, 12b*S*)-aminoindoloquinolizidine from 4d**[11-12] ........ 15
9 **Total synthesis of (+)-woodinine from 5l**[1,9,13] ........ 18
10 **Mechanistic studies** ........ 21
11 **X-ray data of compound 4a (CDCC number:2292538)** ........ 24
12 **Characterization of starting materials** ........ 35
13 **Characterization of products** ........ 39
14 **References** ........ 73
15 **Copies of NMR spectra** ........ 74

## 1 General information

Commercially available chemicals were obtained from Adamas-Beta, Acros Organics, Aldrich Chemical Co., Alfa Aesar, Bidepharm, TCI and used as received unless otherwise stated. Anhydrous solvent, purchased from Adamas and J&K Chemical, were used as received. (*R*)-STRIP (*R*)-CPA-1 (production batch: 134900) purchased from Daicel Chiral Technologies (China) Co., Ltd. $[Ir(dF(CF_3)ppy)_2(dtbpy)]PF_6$ (production batch: BRY343) purchased from Bide Pharmaceutical Co. Ltd. All reactions were carried out using 20 mL schlenk tube at Argon atmosphere unless otherwise stated. TLC were performed on silica gel Huanghai HSGF254 plates and visualization of the developed chromatogram was performed by fluorescence quenching ($\lambda_{max}$= 254 nm). Flash chromatography was carried out on $SiO_2$ (silica gel 60, 200-300 mesh). Melting points are corrected and recorded using digital Büchi Melting Point Apparatus B540.

All the $^{1}H$, $^{13}C$ and $^{19}F$ NMR were recorded on a Bruker Avance II-400 MHz spectrometer. Solvent used for spectra was DMSO-$d_6$, chloroform-*d* or Benzene-$d_6$. $^{1}H$ chemical shifts are reported in ppm on the δ-scale relative to TMS (δ 0.00), chloroform-*d* (δ 7.26), DMSO-$d_6$ (δ 2.50) or Benzene-$d_6$(δ 7.16), and $^{13}C$ NMR are reported in ppm relative to chloroform-*d* (δ 77.16), DMSO-$d_6$ (δ 39.52) or Benzene-$d_6$(δ 128.06). Data for $^{1}H$ NMR are recorded as follows: chemical shift (δ, ppm) and multiplicity [s = singlet, d = doublet, t = triplet, m = multiplet or unresolved, brs = broad singlet, coupling constant (s) in Hz, integration]. Data for $^{13}C$ NMR are reported in terms of chemical shift (δ, ppm). IR spectra were recorded on an INVENIO® FT- IR spectrometer. ESI-HRMS data were acquired using a Thermo LTQ Orbitrap XL Instrument equipped with an ESI source and controlled by Xcalibur software. Enantiomeric excess (ee) value was determined by high-performance liquid chromatography (HPLC) analysis using a chiral stationary phase on Agilent Technologies 1260 Infinity II instrument in comparison with the authentic racemates. All the chiral stationary phases including Chiral AD-H, MX(2) and NX(2),used were purchased from Daicel Chiral Technologies (China) Co., Ltd. or Guangzhou FLM Scientific Instrument Co. Ltd.. Optical rotations were measured using a Rudolph Autopol I polarimeter.

## 2 Procedure for the preparation of starting materials

### 2.1 General procedure for the preparation of β-Carbolines[1-4]

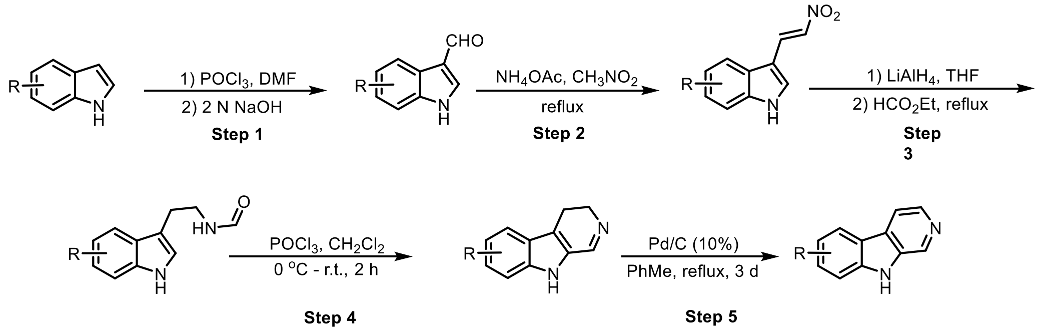


**Step 1:** To a three-necked flask, indole (1.0 equiv.) and DMF (1.0 M) were added. The mixture was stirred at 0 °C, freshly distilled $POCl_3$ (1.2 equiv.) was added in 15 min. The obtained mixture was stirred at 40 °C for another 2 h before 2 N NaOH aqueous solution was added, then continued to stir at 90 °C for another 1 h. EtOAc was added into the mixture to dissolve the solid and extracted the aqueous layer with EtOAc. The combined organic phase was washed with brine, dried over anhydrous $Na_2SO_4$, and evaporated under reduced pressure to obtain the product.

**Step 2:** To a round bottom flask, aldehyde (1.0 equiv.), ammonium acetate (0.7 equiv.) and nitromethane (15 mL/g of aldehyde) were added, then the reaction mixture was refluxed for 1-2 h. The solvent was removed in vacuum and the residue was washed with water, filtered and dried over infrared light to furnish the desired nitro-olefin. The crude nitro-olefin was used directly for further transformation without purification.

**Step 3:** Under the argon atmosphere, a tetrahydrofuran solution of lithium aluminum hydride (6.0 equiv., 1.0 M) was added to a tetrahydrofuran solution of nitro-olefin (1.0 equiv.) at -78 °C. The resulting mixture was allowed to stirred for another 16 h. The reaction was quenched by dropwise addition of water until effervescence ceased. The mixture was then diluted with EtOAc before addition of saturated aqueous solution of Rochelle's salt and the subsequent biphasic mixture was stirred for 24 h. The organic layer was separated, aqueous phase was extracted with EtOAc. The combined organic layer was washed with brine, dried over anhydrous $Na_2SO_4$, and concentrated to dryness. The remaining residue was purified by flash column chromatography on basic alumina (200−300 mesh) using $CH_2Cl_2$ and MeOH as eluents to give the corresponding tryptamines. Then a mixture of the tryptamine in $HCO_2Et$ (1.0 M) was refluxed for 16 h. The solvent was removed in vacuum and the corresponding *N*-(2-(1*H*-indol-3-yl)ethyl)-formamide was obtained without further purification.

**Step 4:** To a round bottom flask, *N*-formyltryptamine (1.0 equiv.) and $CH_2Cl_2$ were added, the mixture was cooled to 0 °C, then freshly distilled $POCl_3$ (3.0 equiv.) was added slowly to the above solution. The reaction mixture was stirred at room temperature for another 2 h. The mixture was concentrated in vacuo to remove unconsumed $POCl_3$ and $CH_2Cl_2$ to give a dark solid residue. The dark solid residue was then suspended in EtOAc and extracted with 10% AcOH/$H_2O$. The combined aquous phase was basified with conc. aquous ammonia until pH = 9. The obtained mixture was extracted with $CH_2Cl_2$. The organic layers

were combined and washed with brine, dried over anhydrous $Na_2SO_4$, and concentrated to dryness to obtain 4,9-dihydro-3*H*-pyrido[3,4-*b*]indole.

**Step 5:** To a round bottom flask, 10% Pd/C (75.0 mg/mmol), 4,9-dihydro-3*H*-pyrido[3,4-*b*]indole (1.0 equiv.) and toluene were added, then the reaction mixture was refluxed for 3 days. The solution was filtrated and the residue was washed with a $CH_2Cl_2$: MeOH (9:1) solution three times, and concentrated to dryness. The remaining residue was purified by flash column chromatography on basic alumina (200−300 mesh) using *n*-hexane and EtOAc as eluents to give the corresponding products.

**β-Carbolines and heteroarenes included in the manuscript**:

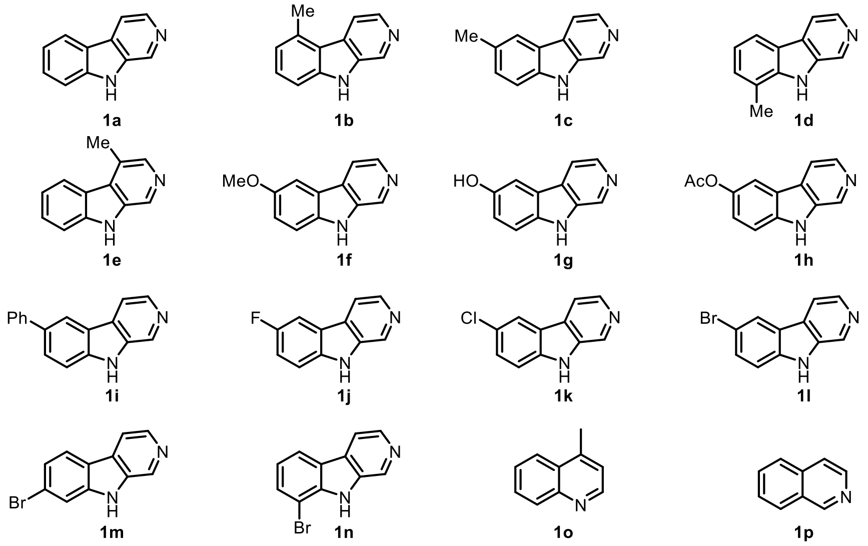


## 2.2 General procedure for the preparation of diazocarbonyl compounds[5]

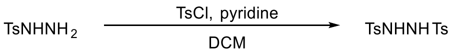


**Synthesis of *N,N'*-ditosylhydrazine (TsNHNHTs)**: A flame-dried round-bottomed flask fitted with a magnetic stir bar was charged with *p*-toluenesulfonyl hydrazide (9.3 g, 50.0 mmol) and *p*-toluenesulfonyl chloride (14.3 g, 75.0 mmol) in 50 mL of anhydrous $CH_2Cl_2$. The suspension was stirred at room temperature and pyridine (6.0 mL, 75.0 mmol) was added dropwise over 1 min. During addition, the reaction mixture became homogenous and turned yellow. White precipitate was observed within 3 min and the reaction mixture was stirred for 1.5 h. $Et_2O$ (200 mL) and $H_2O$ (100 mL) were added and stirred at 0 °C for 15 min. The precipitated white solid was collected in a Büchner funnel using suction filtration and washed with $Et_2O$ (100 mL). The obtained solid was dissolved in boiling MeOH (400 mL). After cooling to the room temperature, precipitate appeared. About 200 mL of MeOH was removed in vacuum before cooled to 0 °C. The precipitate was collected in a Büchner funnel using suction filtration and washed with cold MeOH (20 mL) and $Et_2O$ (100 mL) to give TsNHNHTs (14.0 g, 82%).

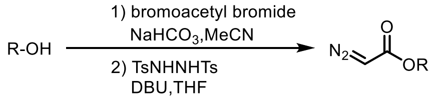


**Synthesis of diazocarbonyl compounds**: To a round bottom flask, alcohols (1.0 equiv.) and $NaHCO_3$ (3.0 equiv.) were dissolved in acetonitrile (0.2 M) and bromoacetyl bromide (1.5 equiv.) was added slowly at 0 °C. After stirring at the temperarture for 10 min, the reaction was quenched with $H_2O$. The solution was extracted with $CH_2Cl_2$ three times. The organic phase was washed with brine and dried over anhydrous $Na_2SO_4$. The solvent was evaporated, and the residue was used without further purification. The bromoacetate and TsNHNHTs (2.0 equiv.) were dissolved in THF and cooled to 0 °C, DBU (5.0 equiv.) was added dropwise and stirred at that temperature for 10 min. After addition of the saturated $NaHCO_3$ solution, the mixture was extracted with $CH_2Cl_2$ three times. The combined organic phase was washed with brine, dried over $Na_2SO_4$ and evaporated to give the crude diazoacetate. Purification of the crude diazoacetate was performed with neutral silicagel to give diazocarbonyl compounds.

**Diazocarbonyl compounds included in the manuscript**:

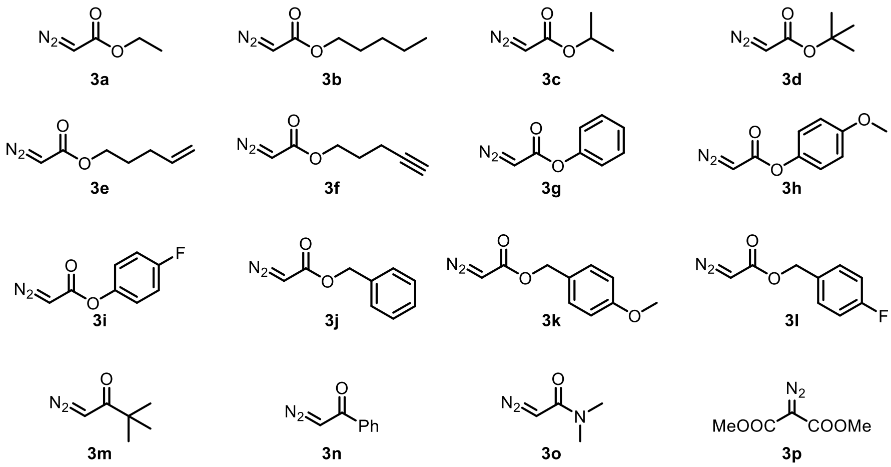


## 2.3 Synthesis of *N*-(prop-1-en-1-yl)acetamide 2f[6]

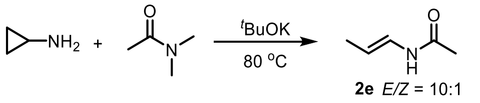


To a mixture of cyclopropyl amine (1 mmol, 1 equiv.) and *N,N*-dimethylacetamide (10 mmol, 10 equiv.), $^t$BuOK (1.5 mmol, 1.5 equiv.) was added. The mixture was stirred at 80 °C in a sealed tube. The progress of the reaction was monitored by TLC visualized with UV short wavelength followed by iodine or ninhydrin stain. After completion, the mixture was diluted with water (10 mL) and extracted with EtOAc (3 x 10 mL). The combined organic phase was washed with brine, dried over anhydrous $Na_2SO_4$, filtered and concentrated in vacuo. The crude residue was then purified by column chromatography on silica gel with EtOAc-hexane (20/80 to 40/60) to obtain pure products **2f** (70.4 mg, 71% yield, *E/Z* = 10:1).

## 2.4 Synthesis of 1-deuterated β-Carboline 1a-D[7-8]

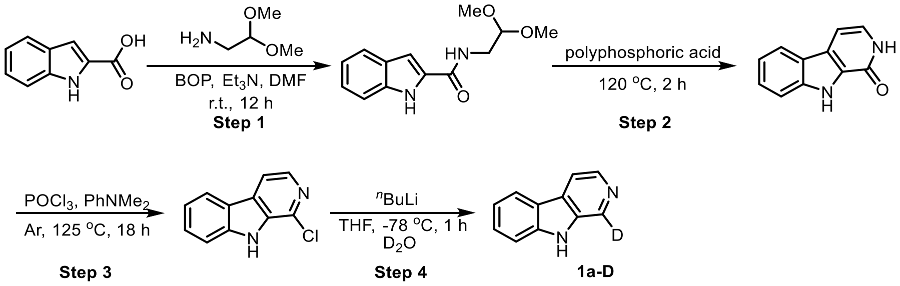


**Step 1[7]:** To a round bottom flask, indole-2-carboxylic acid (10 mmol, 1.0 equiv.), aminoacetaldehyde dimethyl acetal (20 mmol, 2.0 equiv.), $Et_3N$ (30 mmol, 3.0 equiv.), 1*H*-benzotriazol-1-yloxytris-(dimethylamino)phosphonium hexafluorophosphate BOP (10 mmol, 1.0 equiv.) and DMF 50 mL were added. The mixture was stirred at room temperature for 12 h, then diluted with EtOAc and $H_2O$. The aqueous phase was extracted with EtOAc. The combined organic layers were washed with brine, and dried over anhydrous $Na_2SO_4$. After filtration and concentration, the residue was purified by flash column chromatograph ($CH_2Cl_2$/MeOH = 95:5) to give *N*-(2,2-dimethoxyethyl)-1*H*-indole-2-carboxamide (2.008 g, 81% yield).

**Step 2[7]:** A mixture of the *N*-(2,2-dimethoxyethyl)-1*H*-indole-2-carboxamide (8 mmol, 1.0 equiv.) and polyphosphoric acid (2 g) were stirred at 120 °C for 2 h. After cooling to room temperature, treated the reaction mixture with saturated aqueous $NaHCO_3$ until pH = 7. Extracted the reaction mixture with EtOAc. The combined organic phases were washed with brine, dried over anhydrous $Na_2SO_4$, filtered and concentrated in vacuo. The crude residue was then purified by column chromatography on silica gel with $CH_2Cl_2$-MeOH (95/5) to obtain pure products 2,9-dihydro-1*H*-pyrido[3,4-*b*]indol-1-one (0.927 g, 63% yield).

**Step 3[8]:** To a round bottom flask, 2,9-dihydro-1*H*-pyrido[3,4-*b*]indol-1-one (5 mmol, 1.0 equiv.), *N*,*N*-dimethylaniline (0.125 mmol, 0.025 equiv.) and $POCl_3$ (250 mmol, 50 equiv.) were added. Then the reaction mixture was refluxed for 18 h. Slowly pour the reaction mixture into a saturated aqueous solution of sodium carbonate and extracted with EtOAc. The combined organic phases were washed with brine, dried over anhydrous $Na_2SO_4$, filtered and concentrated in vacuo. The crude residue was then purified by column chromatography on silica gel with hexane-actone (90/10) to obtain pure products 1-chloro β-Carboline (0.805 g, 79% yield).

**Step 4:** Under an inert argon atmosphere, a tetrahydrofuran solution of $^n$BuLi (2.4 mmol, 2.4 equiv., 2.4 M) was added to a tetrahydrofuran solution of 1-chloro β-Carboline (1 mmol, 1.0 equiv.) at -78 °C. After stirring 1 h at the temperarture, the reaction was quenched with $D_2O$ and extracted with EtOAc. The combined organic phases were washed with brine, dried over anhydrous $Na_2SO_4$, filtered and concentrated in vacuo. The crude residue was then purified by column chromatography on silica gel with $CH_2Cl_2$-MeOH (95/5) to obtain pure products **1a-D** (35.1 mg, 21% yield, 95% D). **$^1$H NMR** (400 MHz, DMSO) δ 11.61 (s, 1H), 8.33 (d, *J* = 5.2 Hz, 1H), 8.24 (d, *J* = 7.9 Hz, 1H), 8.11 (d, *J* = 5.2 Hz, 1H), 7.63 – 7.50 (m, 2H), 7.24 (t, *J* = 8.1 Hz, 1H). **HRMS** (ESI-TOF) m/z: calcd for $C_{11}H_8DN_2^+$ $[M + H]^+$, 170.0823;

found, 170.0850.

## Substrate limitations:

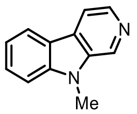
**1q**

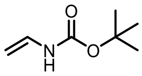
**2e**

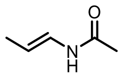
**2f**

## 3 Optimization of reaction conditions

**Table S1**. Optimization of Asymmetric Multicomponent Minisci Reaction of β-carbolines Using α-Bromo Carbonyl Compounds as Radical Processor[a]

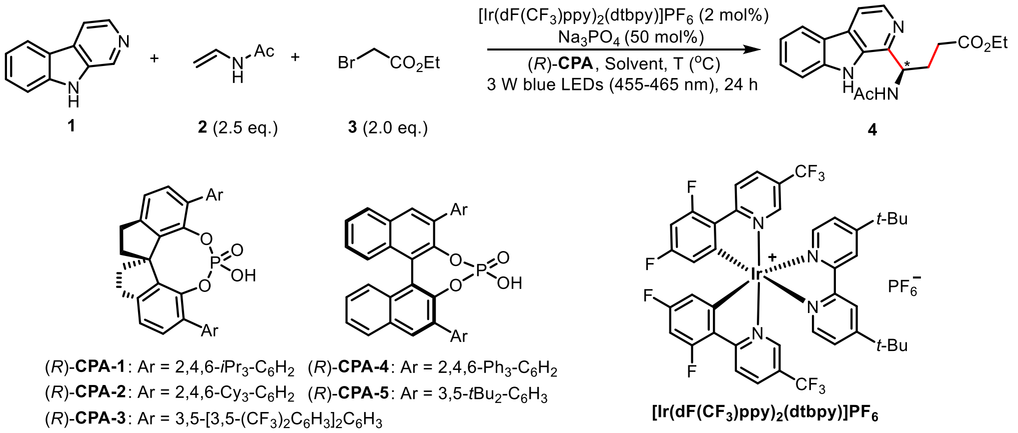


| Entry | Solvent (mL) | **CPA** (mol%) | *T* (°C) | Yield (%)[b] | ee (%)[c] |
|---|---|---|---|---|---|
| 1 | Dioxane (2.0) | (*R*)-**CPA-1** (5) | 25 | 72 | 23 |
| 2 | Dioxane (2.0) | (*R*)-**CPA-2** (5) | 25 | 63 | 19 |
| 3 | Dioxane (2.0) | (*R*)-**CPA-3** (5) | 25 | 56 | 16 |
| 4 | Dioxane (2.0) | (*R*)-**CPA-4** (5) | 25 | 72 | 12 |
| 5 | Dioxane (2.0) | (*R*)-**CPA-5** (5) | 25 | 76 | 9 |
| 6 | $CH_2Cl_2$ (2.0) | (*R*)-**CPA-1** (5) | 25 | 66 | 31 |
| 7 | $CH_3CN$ (2.0) | (*R*)-**CPA-1** (5) | 25 | 16 | 5 |
| 8 | EtOAc (2.0) | (*R*)-**CPA-1** (5) | 25 | 61 | 19 |
| 9 | THF (2.0) | (*R*)-**CPA-1** (5) | 25 | <10 | - |
| 10 | MTBE (2.0) | (*R*)-**CPA-1** (5) | 25 | 50 | 59 |
| 11 | MTBE (3.0) | (*R*)-**CPA-1** (5) | 25 | 62 | 64 |
| 12 | MTBE (3.0) | (*R*)-**CPA-1** (10) | 25 | 71 | 67 |
| 13 | MTBE (3.0) | (*R*)-**CPA-1** (10) | 10 | 88 | 64 |
| 14 | MTBE (3.0) | (*R*)-**CPA-1** (10) | -40 | <10 | - |
| 15[d] | MTBE (3.0) | (*R*)-**CPA-1** (10) | -40 | 63 | 66 |
| 16[e] | MTBE (3.0) | (*R*)-**CPA-1** (10) | -40 | 65 | 67 |

[a]Reaction conditions: **1** (0.1 mmol), **2** (0.25 mmol), **3** (0.2 mmol), $[Ir(dF(CF_3)ppy)_2(dtbpy)]PF_6$ (2 mol%), $Na_3PO_4$ (0.05 mmol), argon atmosphere, 24 h. [b]Yield of isolated product. [c]Determined by HPLC analysis on a chiral stationary phase. [d]40 W Kessil blue LEDs (456 nm). [e]48 h. THF = tetrahydrofuran; MTBE = methyl tert-butyl ether.

**Table S2**. Screening of Chiral Phosphoric Acid for Asymmetric Multicomponent Minisci Reaction of β-carbolines Using Diazo Compounds as Radical Processor[a]

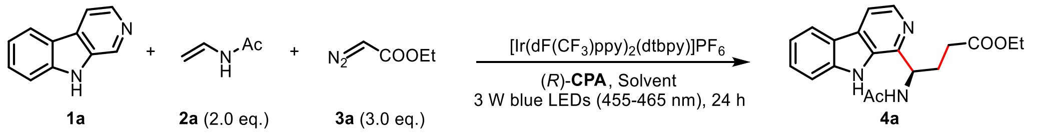


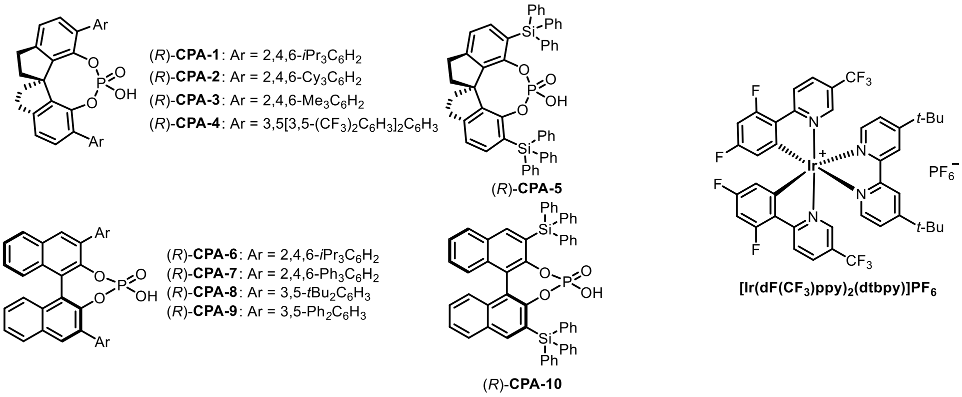


| Entry | Solvent (mL) | **CPA** (mol%) | *T* (°C) | Yield (%)[b] | ee (%)[c] |
|---|---|---|---|---|---|
| 1 | Dioxane (2.0) | (*R*)-**CPA-1** (5) | 10 | 62 | 33 |
| 2 | Dioxane (2.0) | (*R*)-**CPA-2** (5) | 10 | 53 | 15 |
| 3 | Dioxane (2.0) | (*R*)-**CPA-3** (5) | 10 | 77 | 8 |
| 4 | Dioxane (2.0) | (*R*)-**CPA-4** (5) | 10 | 51 | 21 |
| 5 | Dioxane (2.0) | (*R*)-**CPA-5** (5) | 10 | 58 | 0 |
| 6 | Dioxane (2.0) | (*R*)-**CPA-6** (5) | 10 | 52 | 0 |
| 7 | Dioxane (2.0) | (*R*)-**CPA-7** (5) | 10 | 63 | 18 |
| 8 | Dioxane (2.0) | (*R*)-**CPA-8** (5) | 10 | 56 | 11 |
| 9 | Dioxane (2.0) | (*R*)-**CPA-9** (5) | 10 | 77 | 5 |
| 10 | Dioxane (2.0) | (*R*)-**CPA-10** (5) | 10 | 53 | 4 |

[a]Reaction conditions: **1a** (0.1 mmol), **2a** (0.2 mmol), **3a** (0.3 mmol), $[Ir(dF(CF_3)ppy)_2(dtbpy)]PF_6$ (2 mol%), 4 Å MS (25.0 mg), argon atmosphere, 24 h. [b]Yield of isolated product. [c]Determined by HPLC analysis on a chiral stationary phase.

**Table S3**. Optimization of Asymmetric Multicomponent Minisci Reaction of β-carbolines Using Diazo Compounds as Radical Processors[a]

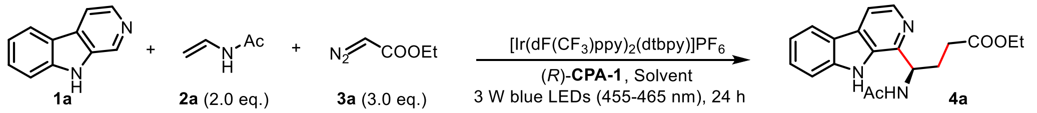


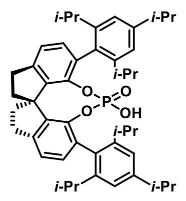

(*R*)-**CPA-1**

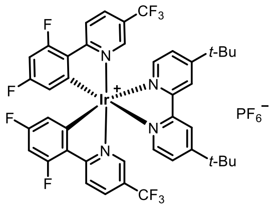

**[Ir(dF(CF$_3$)PPy)$_2$(dtbpy)]PF$_6$**

| Entry | Solvent (mL) | **CPA** (mol%) | Time (h) | *T* (°C) | Yield | ee (%)[c] |
|---|---|---|---|---|---|---|
| 1 | Dioxane (2.0) | (*R*)-**CPA-1** (5) | 24 | 10 | 62 | 33 |
| 2 | $CH_2Cl_2$ (2.0) | (*R*)-**CPA-1** (5) | 24 | 10 | 68 | 29 |
| 3 | THF (2.0) | (*R*)-**CPA-1** (5) | 24 | 10 | 52 | 63 |
| 4 | MTBE (2.0) | (*R*)-**CPA-1** (5) | 24 | 10 | 50 | 59 |
| 5 | Isopropyl ether | (*R*)-**CPA-1** (5) | 24 | 10 | 29 | 54 |
| 6 | DME (2.0) | (*R*)-**CPA-1** (5) | 24 | 10 | 57 | 58 |
| 7 | EtOAc (2.0) | (*R*)-**CPA-1** (5) | 24 | 10 | 64 | 21 |
| 8 | THF (2.0) | (*R*)-**CPA-1** (10) | 24 | 10 | 56 | 70 |
| 9 | THF (2.0) | (*R*)-**CPA-1** (10) | 24 | -25 | 67 | 76 |
| 10 | THF (2.0) | (*R*)-**CPA-1** (10) | 48 | -25 | 74 | 76 |
| 11[d] | THF (2.0) | (*R*)-**CPA-1** (10) | 48 | -40 | 87 | 84 |
| 12[d] | THF (4.0) | (*R*)-**CPA-1** (10) | 48 | -40 | 86 | 87 |
| 13[d] | **THF (8.0)** | (*R*)-**CPA-1** (10) | 48 | **-40** | **80** | **92** |
| 14[de] | THF (8.0) | (*R*)-**CPA-1** (10) | 48 | -40 | 61 | 88 |
| 15[df] | THF (8.0) | (*R*)-**CPA-1** (10) | 48 | -40 | 0 | - |
| 16[dg] | THF (8.0) | **-** | 48 | -40 | 11 | - |
| 17 | THF (8.0) | (*R*)-**CPA-1** (10) | 48 | -40 | 71 | 89 |
| 18[h] | THF (8.0) | (*R*)-**CPA-1** (10) | 48 | -40 | 56 | 92 |
| 19[i] | THF (8.0) | (*R*)-**CPA-1** (10) | 48 | -40 | 26 | 93 |
| 20[j] | THF (8.0) | (*R*)-**CPA-1** (10) | 48 | -40 | trace | - |
| 21[k] | THF (8.0) | (*R*)-**CPA-1** (10) | 48 | -40 | 12 | 82 |

[a]Reaction conditions: **1a** (0.1 mmol), **2a** (0.2 mmol), **3a** (0.3 mmol), [Ir] cat. (2 mol%), 4 Å MS (25.0 mg), argon atmosphere, 24 h. [b]Yield of isolated product. [c]Determined by HPLC analysis on a chiral stationary phase. [d]The **3a** add once every 12 h, 3 times in total. [e]no 4 Å MS. [f]No [Ir] cat.. [g]No CPA. [h][Ir(dF(CF$_3$)ppy)$_2$(dtbpy)]PF$_6$ (1 mol%). [i][Ir(dF(CF$_3$)ppy)$_2$(dtbpy)]PF$_6$ (0.5 mol%). [j]390 nm LEDs instead of 455-465 nm LEDs. [k]520nm LEDs instead of 455-465 nm LEDs. THF = tetrahydrofuran; MTBE = tert-butyl methyl ether; DME = 1,2-dimethoxyethane.

## 4 General Procedure for the Photocatalytic Enantioselective Radical Cascade Multicomponent Minisci Reaction of β-Carbolines Utilizing Diazo Compounds as Radical Precursors

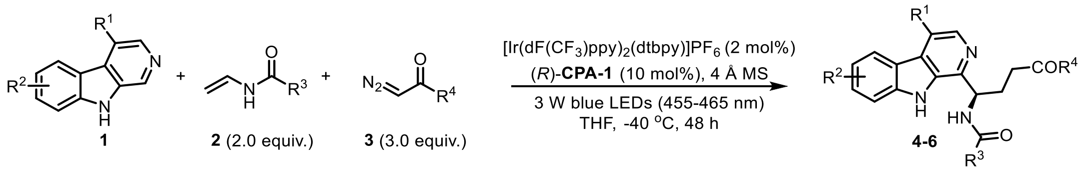


Sequentially, β-carbolines (0.20 mmol, 1.0 equiv.), alkenyl amide (0.40 mmol, 2.0 equiv.), Ir[dF($CF_3$)ppy)$_2$(dtbpy)]$PF_6$ (4.4 mg, 0.004 mmol, 2 mol%), (*R*)-**CPA-1** (14.4 mg, 0.02 mmol, 10 mol%) and 4 Å MS (50.0 mg) were added into a 20 mL dried Schlenk tube containing a stirrer bar. After evacuated and refilled with argon for three times, anhydrous, freshly argon-sparged THF (16.0 mL) was added via syringe. The reaction mixture was stirred under an argon atmosphere at −40 °C in the incubator for 1 h without light. It has been confirmed that the incubator is capable of keeping the temperature constantly at -40 °C during the reaction time. The diazocarbonyl compounds (1.0 equiv.) was then added every 12 h by injection for 3 times in total. The obtained mixture was irradiated by 3 W blue LED (λ = 455−465 nm) for another 48 h. The reaction was monitored by TLC. After completion of the reaction, the solvent was removed in vacuo and the crude residue was purified via flash column chromatography on silica gel using *n*-hexane/acetone or $CH_2Cl_2$/MeOH to give the corresponding products.

### Incubator and setup of reactions:

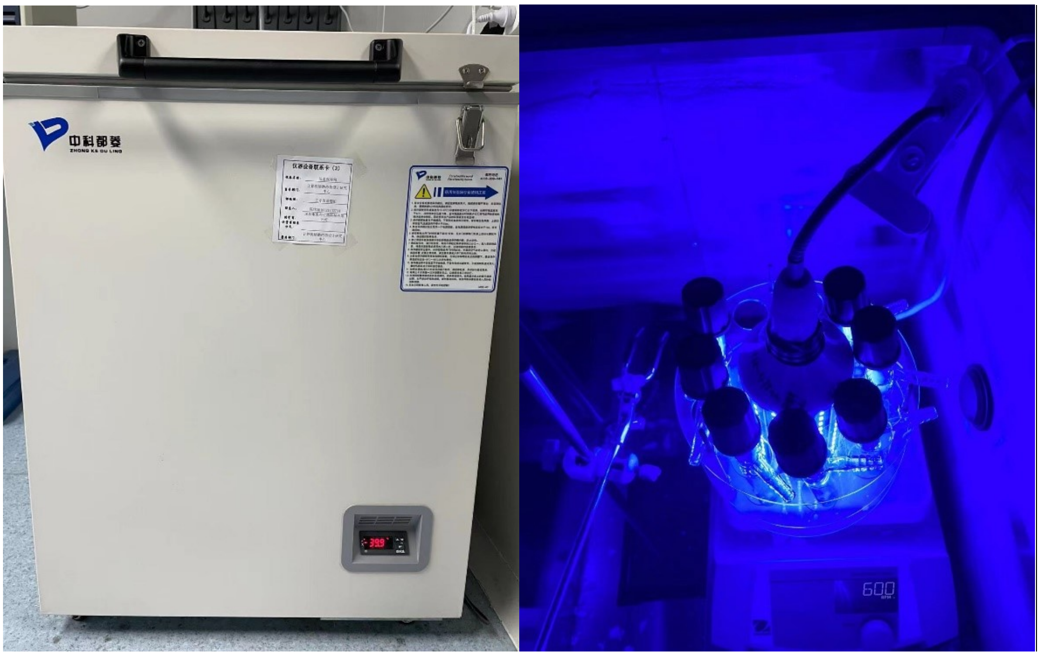

## 5 Scale-up reaction

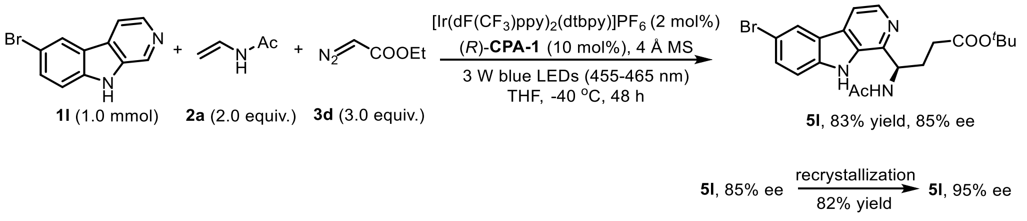


Sequentially, 6-bromo-9*H*-pyrido[3,4-*b*]indole (1.0 mmol, 1.0 equiv.), *N*-vinylacetamide (2.0 mmol, 2.0 equiv.), Ir[dF(CF$_3$)ppy)$_2$(dtbpy)]PF$_6$ (22.4 mg, 0.02 mmol, 2 mol%), (*R*)-**CPA-1** (14.4 mg, 0.02 mmol, 10 mol%) and 4 Å MS (250.0 mg) were added to a 100 mL dried Schlenk tube containing a stirrer bar. After evacuated and refilled with argon three times, anhydrous, freshly argon-sparged THF (80.0 mL) was added via syringe. The reaction mixture was stirred under an argon atmosphere at −40 ºC in the incubator for 1 h without light. It has been confirmed that the incubator is capable of keeping the temperature constantly at -40 ºC during the reaction time. The diazocarbonyl compounds (1.0 equiv.) was then added every 12 h by injection for 3 times in total. The obtained mixture was irradiated by 3 W blue LED (λ = 455−465 nm) for another 48 h. The reaction was monitored by TLC. After completion of the reaction, the solvent was removed in vacuo and the crude residue was purified via flash column chromatography on silica gel using CH$_2$Cl$_2$/MeOH=3:97 acetate as eluents to give the corresponding products **5l** (352.2 mg, 83% yield, 85% ee). Recrystallization of compound **5l** in dichloromethane and petroleum ether increased the ee value to 95% with 82% recrystallization yield.

## 6 Reaction with substituted *N*-vinylacetamide (*E*)-*N*-(prop-1-en-1-yl)acetamide 2f

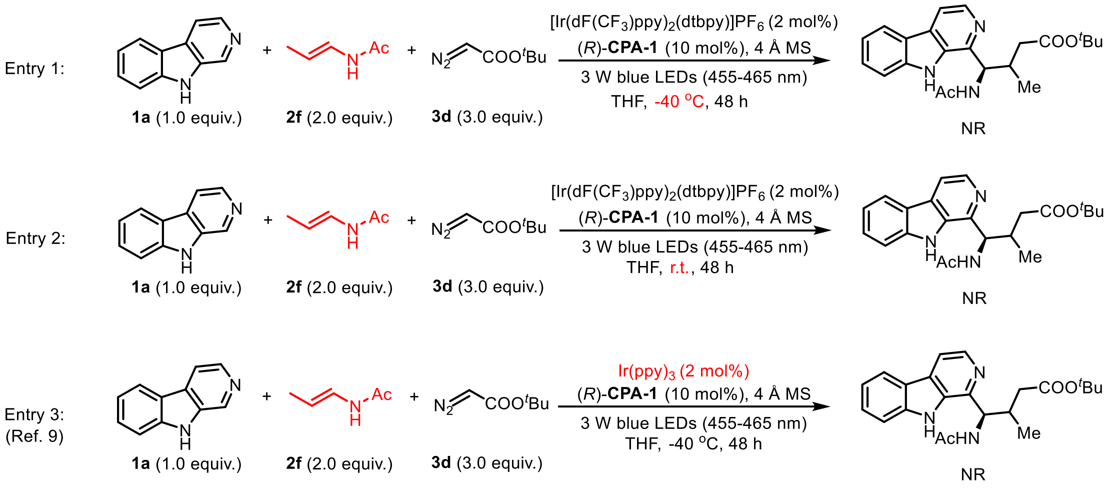

## 7 Total synthesis of (*R*)-dihydroeudistomin I from 4d[9-10]

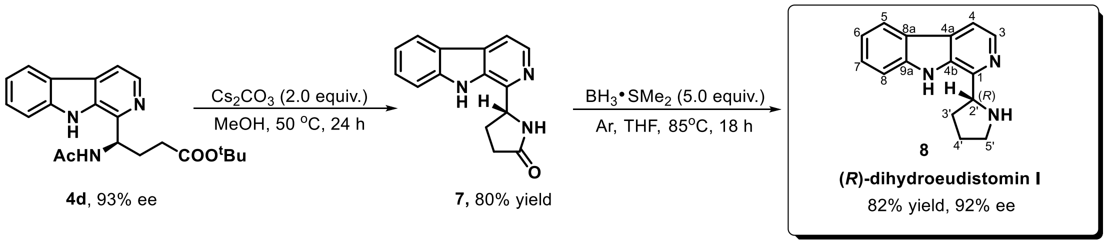


**Cyclization of 4d[9].** To a stirred solution of **4d** (185.0 mg, 0.5 mmol, 93% ee) in MeOH (5.0 mL), $Cs_2CO_3$ (325.0 mg, 1.0 mmol) was added at room temperature. The obtained mixture was stirred at 50 °C for 16 h. After completion of reaction as detected by TLC, the reaction was evaporated and the residue was purified directly by flash column chromatograph ($CH_2Cl_2$/MeOH = 96:4) to give **7** (101.1 mg, 80% yield) as a white solid. **$^1$H NMR** (400 MHz, DMSO) δ 11.64 (s, 1H), 8.31 (d, *J* = 5.1 Hz, 1H), 8.23 (d, *J* = 7.9 Hz, 1H), 8.07 (s, 1H), 8.04 (d, *J* = 5.3 Hz, 1H), 7.63 (d, *J* = 7.8 Hz, 1H), 7.59 – 7.51 (m, 1H), 7.30 – 7.20 (m, 1H), 5.33 (dd, *J* = 7.9, 5.2 Hz, 1H), 2.59 – 2.51 (m, 1H), 2.46 – 2.35 (m, 1H), 2.35 – 2.25 (m, 1H), 2.21 (ddd, *J* = 15.8, 7.8, 3.6 Hz, 1H). **$^{13}$C NMR** (101 MHz, DMSO) δ 177.8, 145.8, 141.0, 138.1, 133.3, 128.7, 122.2, 121.2, 119.8, 114.5, 112.5, 55.3, 30.6, 27.6. **HRMS** (ESI-TOF) m/z: calcd for $C_{15}H_{14}N_3O^+$ $[M + H]^+$, 252.1131; found, 252.1128. **$[\alpha]_D^{20}$** = -21.2 (c = 0.2, $CHCl_3$).

**Synthesis of (*R*)-dihydroeudistomin I (8).** Under an inert argon atmosphere, borane dimethyl sulfide complex (2.0 mmol) was added dropwise via syringe to a stirred solution of **7** (101.1 mg, 0.4 mmol) in 10 mL THF at 0 °C. The reaction mixture was then heated under reflux overnight before it was cooled to room temperature. The solvent was removed under reduced pressure, and the residue was taken up in a 3:1 mixture of THF and water (8.0 mL) and aqueous NaOH (4.0 mL, 5 N). The resulting mixture was heated at 90 °C for 1.5 h, and then cooled to room temperature, diluted with EtOAc (30 mL) and $H_2O$ (40 mL). The aqueous phase was extracted with EtOAc (3×20 mL). The combined organic layers were washed with water (20 mL), brine (20 mL), and dried over anhydrous $Na_2SO_4$. After filtration and concentration, the residue was purified by flash column chromatograph ($CH_2Cl_2$/MeOH = 91:9) to give **(*R*)-dihydroeudistomin I (8)** (77.9 mg, 82% yield, 92% ee) as a light yellow solid. The structure was confirmed by comparing with the reported spectroscopic data as shown in **Table S4** of **dihydroeudistomin I**[10]. **$^1$H NMR** (400 MHz, $CDCl_3$) δ 10.63 (s, 1H), 8.30 (d, *J* = 5.3 Hz, 1H), 8.08 (d, *J* = 7.9 Hz, 1H), 7.79 (d, *J* = 5.3 Hz, 1H), 7.61 – 7.46 (m, 2H), 7.30 – 7.18 (m, 1H), 4.82 (t, *J* = 7.7 Hz, 1H), 3.40 – 3.08 (m, 3H), 2.48 – 2.31 (m, 1H), 2.15 – 2.01 (m, 1H), 2.01 – 1.81 (m, 2H). **$^{13}$C NMR** (101 MHz, $CDCl_3$) δ 146.4, 140.2, 137.8, 134.2, 129.3, 128.2, 121.7, 121.4, 119.6, 113.4, 111.9, 63.9, 47.2, 32.7, 25.7. **HRMS** (ESI-TOF) m/z: calcd for $C_{15}H_{16}N_3^+$ $[M + H]^+$, 238.1339; found, 238.1335. **HPLC analysis:** Chiral NX(2) (150 x 4.6 mm, 3 μm, hexane/*i*-PrOH = 80:20, 1.0 mL/min, 25 °C, 254 nm), $t_r$ (major) = 8.84 min, $t_r$ (minor) = 10.74 min. **$[\alpha]_D^{20}$** = -19.3 (c = 0.2, $CHCl_3$).

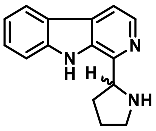


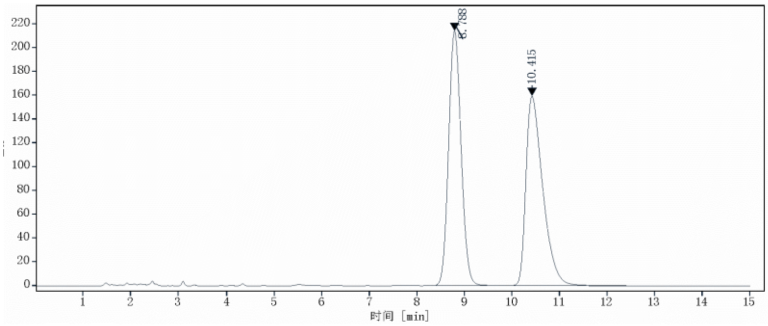


| Entry | Retention Time | Height | Area | Area% |
|---|---|---|---|---|
| 1 | 8.79 | 214.16 | 3909.05 | 49.93 |
| 2 | 10.41 | 159.33 | 3920.54 | 50.07 |

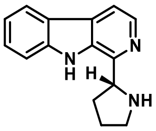


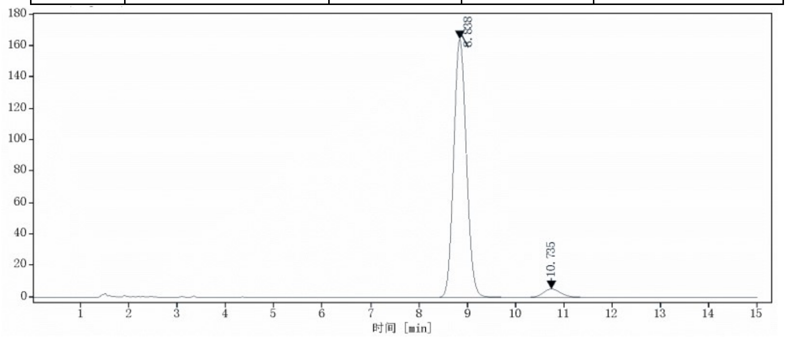


| Entry | Retention Time | Height | Area | Area% |
|---|---|---|---|---|
| 1 | 8.84 | 164.70 | 3007.52 | 95.76 |
| 2 | 10.74 | 5.27 | 133.34 | 4.24 |

**Table S4**. $^1$H and $^{13}$C NMR data for synthetic structure of **dihydroeudistomin I** in $CDCl_3$

| Position | $\delta_H$ (400 MHz, $CDCl_3$) | $\delta_H$ (400 MHz, $CDCl_3$) | $\delta_C$ (100 MH, $CDCl_3$) | $\delta_C$ (100 MHz, $CDCl_3$) |
|---|---|---|---|---|
| | Synthetic | Reported[a] | Synthetic | Reported[a] |
| 1 | - | - | 146.4 | 145.4 |
| 2’ | 4.82 (t, 7.7) | 4.90 (t, 7.6) | 63.9 | 63.1 |
| 3 | 8.30 (d, 5.3) | 8.25 (d, 5.2) | 137.8 | 137.7 |
| 3’ | 2.01-2.48 (m) | 1.80-3.20 (m) | 32.7 | 32.6 |
| 4 | 7.79 (d, 5.3) | 7.75 (d, 5.2) | 113.4 | 113.7 |
| 4’ | 1.81-2.01 (m) | 1.80-3.20 (m) | 25.7 | 25.7 |
| 4a | - | - | 121.4 | 121.4 |
| 4b | - | - | 129.3 | 129.4 |
| 5 | 8.08 (d, 7.9) | 8.05 (d, 8.0) | 121.7 | 121.7 |
| 5’ | 3.08-3.40 (m) | 1.80-3.20 (m) | 47.2 | 47.0 |
| 6 | 7.18-7.30 (m) | 7.22 (m) | 119.6 | 119.6 |
| 7 | 7.46-7.61 (m) | 7.46-7.55 (m) | 128.2 | 128.3 |
| 8 | 7.46-7.61 (m) | 7.46-7.55 (m) | 111.9 | 112.1 |
| 8a | - | - | 134.2 | 134.1 |
| 9a | - | - | 140.2 | 140.5 |
| N-H(indole) | 10.63 (br s) | 10.80 (br s) | - | - |
| N-H(pyrrolidine) | 3.08-3.40 (m) | 4.22 (br s) | - | - |

[a]Data from Ref. 10.

## 8 Total synthesis of (1*R*, 12b*S*)-aminoindoloquinolizidine from 4d[11-12]

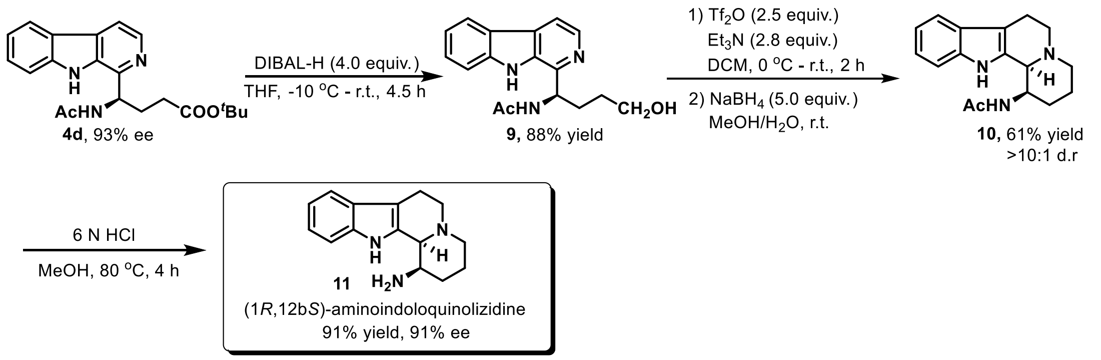


**Reduction of 4d.** A solution of **4d** (370.0 mg, 1.0 mmol, 93% ee) in anhydrous THF (10.0 mL) was placed under nitrogen atmosphere, cooled to -10 °C, and treated with DIBAL-H (4.0 mL of a 1.0 M solution in THF, 4.0 mmol). After 30 min, the cooling bath was removed and the reaction was warmed to room temperature. After stirring at that temperature for another 4 h, the mixture was quenched with saturated $NaHCO_3$ aq. (20 mL) and extracted with $CH_2Cl_2$ (3 x 20 mL). The combined organic layer was washed with brine, dried over $Na_2SO_4$, the solvent was evaporated and the residue was purified by flash column chromatograph ($CH_2Cl_2$/MeOH = 96:4) to give **9** (261.5 mg, 88% yield) as a white solid. **$^1$H NMR** (400 MHz, DMSO) δ 11.61 (s, 1H), 8.39 (d, *J* = 8.2 Hz, 1H), 8.31 (d, *J* = 5.2 Hz, 1H), 8.22 (d, *J* = 7.8 Hz, 1H), 8.01 (d, *J* = 5.2 Hz, 1H), 7.62 (d, *J* = 8.2 Hz, 1H), 7.55 (t, *J* = 7.0 Hz, 1H), 7.24 (t, *J* = 6.8 Hz, 1H), 5.54 (q, *J* = 7.5 Hz, 1H), 4.35 (t, *J* = 5.3 Hz, 1H), 3.36 (q, *J* = 6.3 Hz, 2H), 1.97 – 1.80 (m, 5H), 1.58 – 1.43 (m, 1H), 1.41 – 1.28 (m, 1H). **$^{13}$C NMR** (101 MHz, DMSO) δ 168.9, 145.6, 140.5, 137.2, 133.0, 128.1, 127.9, 121.6, 120.8, 119.3, 113.5, 112.0, 60.7, 49.9, 31.0, 29.1, 22.6. **HRMS** (ESI-TOF) m/z: calcd for $C_{17}H_{21}N_3O_2^+$ $[M + H]^+$, 298.1550; found, 298.1553.

**Synthesis of 10.**

**1) Cyclization of 9.** To a stirred solution of **7** (150.0 mg, 0.5 mmol) in dry $CH_2Cl_2$ (10 mL), $Et_3N$ (0.2 mL, 1.4 mmol) and trifluoromethanesulfonic anhydride (0.21 mL, 1.25 mmol) were added at 0 °C under argon atmosphere. The reaction mixture was stirred at room temperature for another 2 h. After completion of the reaction, concentrated in vacuo to afford crude intermediate, which was used in the next step without further purification.

**2) Reduction of intermediate.** To a solution of crude intermediate in a 3:1 mixture of MeOH and water (8.0 mL), $NaBH_4$ (94.5 mg, 2.5 mmol) was added. The obtained mixture was stirred at room temperature for 2 h. After completion, the reaction was quenched with saturated $NaHCO_3$ aq. (20 mL) and extracted with $CH_2Cl_2$ (3 x 20 mL). The combined organic layer was washed with brine, dried over anhydrous $Na_2SO_4$, the solvent was evaporated and the residue was purified directly by flash column chromatograph ($CH_2Cl_2$/MeOH = 95:5) to give **10** (86.4 mg, 61% yield, >10:1 d.r., according to NMR spectrum) as a yellow solid. **$^1$H NMR** (400 MHz, $CDCl_3$) δ 8.93 (s, 1H), 7.46 (d, *J* = 7.6 Hz, 1H), 7.30 (d, *J* = 7.9 Hz, 1H), 7.10 (dt, *J* = 18.8, 7.3 Hz, 2H), 6.29 (d, *J* = 8.7 Hz, 1H), 4.71 (d, *J* = 5.9 Hz, 1H), 3.44 (s, 1H), 3.11 – 2.90 (m, 3H), 2.72 (d, *J* = 14.6 Hz, 1H), 2.68 – 2.56 (m, 1H), 2.39 (t, *J* = 11.8 Hz,

1H), 2.06 (d, $J$ = 13.5 Hz, 1H), 1.96 – 1.86 (m, 1H), 1.79 (s, 3H), 1.66 (m, 2H). **$^{13}$C NMR** (101 MHz, $CDCl_3$) δ 170.2, 136.6, 132.2, 127.1, 121.5, 119.1, 117.8, 111.5, 109.6, 63.1, 55.9, 53.6, 45.5, 28.9, 23.5, 21.4, 21.0. **HRMS** (ESI-TOF) m/z: calcd for $C_{17}H_{22}N_3O^+$ [M + H]$^+$, 284.1757; found, 284.1761. **[α]$_D^{20}$** = -56.3 (c = 0.2, $CHCl_3$).

**Synthesis of (1*R*,12b*S*)-aminoindoloquinolizidine (11).** To a solution of **10** (86.4 mg, 0.3 mmol) in MeOH (4.0 mL), 6 N hydrochloric acid (6.0 mL) was added. The obtained mixture was stirred at 80 °C. After completion of the reaction as checked by TLC, the solvent was evaporated. Suspended the residues in saturated aq. $Na_2CO_3$ (30 mL) and extracted with $CH_2Cl_2$ (3 x 20 mL). The organic layers were combined and washed with brine, dried over anhydrous $Na_2SO_4$, the solvent was evaporated and the residue was purified directly by flash column chromatograph ($CH_2Cl_2$/MeOH = 90:10) to give **(1*R*,12b*S*)-aminoindoloquinolizidine (11)** (65.8 mg, 91% yield, 91% ee). The structure and absolute configuration were confirmed by comparing with the reported spectroscopic data as shown in **Table S5** and optical rotation of **aminoindoloquinolizidine**[11]**.** **$^1$H NMR** (400 MHz, $CDCl_3$) δ 9.02 (br s, 1H), 7.50 – 7.38 (m, 2H), 7.15 (t, $J$ = 7.5 Hz, 1H), 7.09 (t, $J$ = 7.4 Hz, 1H), 3.41 (d, 3.0 Hz H), 3.11 – 2.53 (m, 6H), 2.44 – 2.31 (m, 1H), 1.76 – 1.48 (m, 4H). **$^1$H NMR** (400 MHz, DMSO) δ 11.06 (s, 1H), 7.39 (d, $J$ = 7.8 Hz, 1H), 7.32 (d, $J$ = 8.1 Hz, 1H), 7.05 (t, $J$ = 7.5 Hz, 1H), 6.97 (t, $J$ = 7.4 Hz, 1H), 6.73 (br s, 2H), 3.93 (s, 1H), 3.51 (s, 1H), 3.07 – 2.90 (m, 2H), 2.87 – 2.75 (m, 1H), 2.68 – 2.52 (m, 2H), 2.41 (t, $J$ = 11.5 Hz, 1H), 2.02 (d, $J$ = 13.5 Hz, 1H), 1.97 – 1.82 (m, 1H), 1.82 – 1.66 (m, 1H), 1.55 (d, $J$ = 13.2 Hz, 1H). **$^{13}$C NMR** (101 MHz, DMSO) δ 136.5, 131.3, 126.6, 120.7, 118.3, 117.6, 111.4, 109.1, 61.5, 54.9, 52.4, 47.2, 27.6, 21.1, 19.4. **HRMS** (ESI-TOF) m/z: calcd for $C_{15}H_{20}N_3^+$ [M + H]$^+$, 242.1652; found, 242.1655. **HPLC analysis:** Chiral MX (2) (150 x 4.6 mm, 3 µm, hexane/*i*-PrOH = 80:20, 0.1% $Et_3N$ (v/v), 1.0 mL/min, 25 °C, 254 nm), $t_r$ (major) = 3.22 min, $t_r$ (minor) = 4.28 min. **[α]$_D^{20}$** = -10.6 (c = 0.1, MeOH).

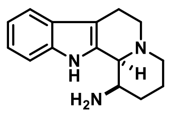


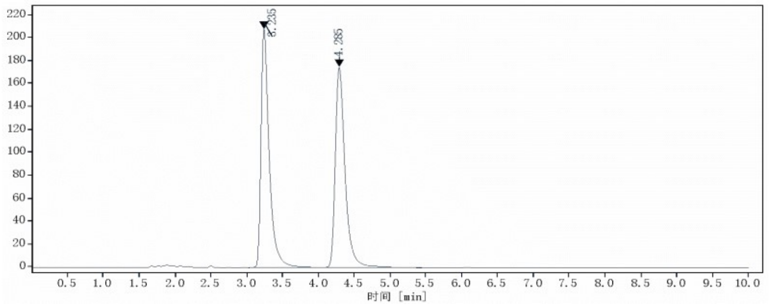

| Entry | Retention Time | Height | Area | Area% |
|---|---|---|---|---|
| 1 | 3.23 | 207.78 | 1660.25 | 50.08 |
| 2 | 4.28 | 174.69 | 1655.08 | 49.92 |

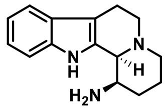


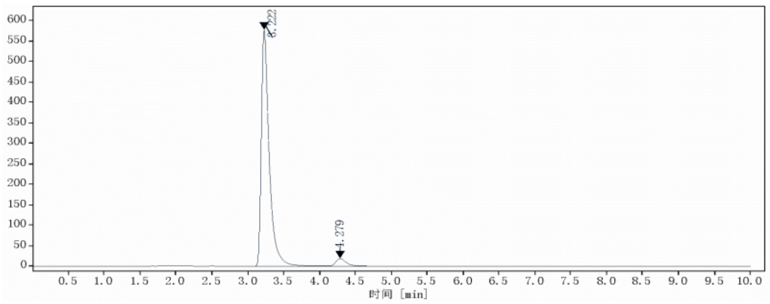


| Entry | Retention Time | Height | Area | Area% |
|---|---|---|---|---|
| 1 | 3.22 | 578.45 | 4452.08 | 95.55 |
| 2 | 4.28 | 19.12 | 207.31 | 4.45 |

**Table S5**. $^{1}$H and $^{13}$C NMR data for synthetic structure of **(1*R*,12b*S*)-aminoindoloquinolizidine** in $CDCl_3$

| Position | $\delta_H$ (400 MHz, $CDCl_3$) | $\delta_H$ (400 MHz, $CDCl_3$) | $\delta_H$ (400 MHz, $CDCl_3$) |
|---|---|---|---|
| | Synthetic | Reported[a] (cis 12b β H) | Reported[a](trans 12b α H) |
| 1 | 2.53-3.11 (m) | 2.5-3.1 (m) | 2.6-3.1 (m) |
| 2a | 1.48-1.76 (m) | 1.5-2.0 (m) | 1.1-1.9 (m) |
| 2e | 1.48-1.76 (m) | 1.5-2.0 (m) | 1.1-1.9 (m) |
| 3a | 1.48-1.76 (m) | 1.5-2.0 (m) | 1.1-1.9 (m) |
| 3e | 1.48-1.76 (m) | 1.5-2.0 (m) | 1.1-1.9 (m) |
| 4a | 2.31-2.44 (m) | 2.37 (td, 11, 11, 2.5) | 2.28 (dt, 7.0, 7.0, 1.5) |
| 4e | 2.53-3.11 (m) | 2.5-3.1 (m) | 2.6-3.1 (m) |
| 6a | 2.53-3.11 (m) | 2.5-3.1 (m) | 2.6-3.1 (m) |
| 6e | 2.53-3.11 (m) | 2.5-3.1 (m) | 2.6-3.1 (m) |
| 7a | 2.53-3.11 (m) | 2.5-3.1 (m) | 2.6-3.1 (m) |
| 7e | 2.53-3.11 (m) | 2.5-3.1 (m) | 2.6-3.1 (m) |
| 8 | 7.38-7.50 (m) | 7.50(dd, 7.0, 2.0) | 7.48 (dd, 7.0, 1.5) |
| 9 | 7.09 (t, 7.4) | 7.11 (td, 7.0, 7.0, 2.0) | 7.08 (dt, 7.0, 7.0, 1.5) |
| 10 | 7.15 (t, 7.5) | 7.20 (td, 7.0, 7.0, 2.0) | 7.13 (dt, 7.0, 7.0, 1.5) |
| 11 | 7.38-7.50 (m) | 7.35 (dd, 7.0, 2.0) | 7.35 (dd, 7.0, 1.5) |
| 12 | **9.02 (br s)** | **8.90 (br s)** | **10.45 (br s)** |
| 12b | **3.41 (d, 3.0)** | **3.32 (d, 3.0)** | **2.6-3.1 (m)** |

[a]Data from Ref. 11-12

## 9 Total synthesis of (+)-woodinine from 5l[1,9,13]

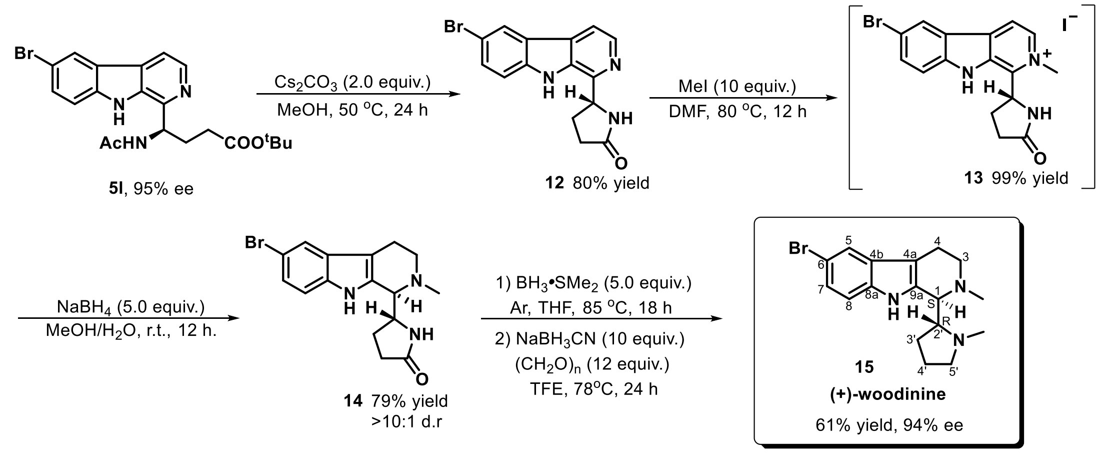


**Cyclization of 5l[9].** To a stirred solution of **5l** (310.0 mg, 0.7 mmol, 95% ee) in MeOH (10 mL), $Cs_2CO_3$ (456.1 mg, 1.4 mmol) was added at room temperature. The obtained mixture was stirred at 50 °C for 24 h. After completion of reaction as detected by TLC, the solvent was evaporated and the residue was purified directly by flash column chromatograph ($CH_2Cl_2$/MeOH = 96: 4) to give **12** (184.8 mg, 80% yield) as a white solid. **$^1$H NMR** (400 MHz, DMSO) δ 11.83 (s, 1H), 8.52 (s, 1H), 8.33 (dd, J = 5.2, 1.2 Hz, 1H), 8.11 (d, J = 5.2 Hz, 1H), 8.05 (s, 1H), 7.67 (dt, J = 8.7, 1.6 Hz, 1H), 7.59 (d, J = 8.7 Hz, 1H), 5.30 (dd, J = 7.9, 5.2 Hz, 1H), 2.58 – 2.52 (m, 1H), 2.44-2.34 (m, 1H), 2.34 – 2.25 (m, 1H), 2.25 – 2.14 (m, 1H). **$^{13}$C NMR** (101 MHz, DMSO) δ 177.8, 146.3, 139.7, 138.3, 133.7, 131.1, 127.6, 124.8, 123.1, 114.8, 114.6, 111.8, 55.2, 30.5, 27.6. **HRMS** (ESI-TOF) m/z: calcd for $C_{15}H_{13}BrN_3O^+$ $[M + H]^+$, 330.0237; found, 330.0239. **$[\alpha]_D^{20}$** = -34.7 (c = 0.2, $CHCl_3$).

**Synthesis of β-carboline methiodide intermediate (13)[1].** To a solution of **12** (184.8 mg, 0.56 mmol) in 4.0 mL DMF, MeI (350 uL, 5.6 mmol) was added. The obtained mixture was stirred at 80 °C for 12 h. After completion of the reaction, filter the mixture to obtain solids, and drying under high vacuum afforded pure β-carboline methiodide intermediate **(13)**, which was used in the next step without further purification.

**Synthesis of 14[1].** To a solution of β-carboline methiodide intermediate **(13)** (67.4 mg, 0.14 mmol) in a 3:1 mixture of MeOH and water (6.0 mL), $NaBH_4$ (26.5 mg, 0.7 mmol) was added. The obtained mixture was stirred at room temperature for 12 h. After completion, the reaction was quenched with saturated $NaHCO_3$ aq. (20 mL) and extracted with $CH_2Cl_2$ (3×15 mL). The combined organic layer was washed with brine (15 mL), dried over $Na_2SO_4$, the solvent was evaporated and the residue was purified directly by flash column chromatograph ($CH_2Cl_2$/MeOH = 96:4) to give **14** (39.3 mg, 79% yield, >10:1 d.r., according to NMR spectrum) as a white solid. **$^1$H NMR** (400 MHz, DMSO) δ 10.83 (s, 1H), 7.58 (d, *J* = 1.9 Hz, 1H), 7.42 (s, 1H), 7.29 (d, *J* = 8.5 Hz, 1H), 7.14 (dd, *J* = 8.6, 1.9 Hz, 1H), 3.88 (q, *J* = 6.6 Hz, 1H), 3.42 (d, *J* = 6.6 Hz, 1H), 3.16 – 3.04 (m, 1H), 2.84-2.71 (m, 2H), 2.49 – 2.42 (m, 1H), 2.39 (s, 3H), 2.15-2.05 (m, 2H), 2.05 – 1.94 (m, 2H). **$^{13}$C NMR** (101 MHz, DMSO) δ 176.9, 135.3, 133.8, 128.8,

123.5, 120.5, 113.5, 111.3, 108.0, 63.2, 56.9, 46.6, 42.7, 30.6, 24.2, 16.8. **HRMS** (ESI-TOF) m/z: calcd for $C_{16}H_{19}BrN_3O^+$ [M + H]$^+$, 348.0706; found, 348.0709. **[α]$_D$$^{20}$** = +26.3 (c = 0.2, $CHCl_3$).

**Synthesis of (+)-woodinine (15).**

**(1) Reduction of 14.** Borane dimethyl sulfide complex (75.0 µL, 0.15 mmol) was added dropwise via syringe to a stirred solution of **14** (35.0 mg, 0.1 mmol) in 1.0 mL THF at 0 °C. The obtained mixture was heated under reflux for 18 h. The solvent was removed under reduced pressure, and the residue was taken up in a 3:1 mixture of THF and water (4.0 mL) and aqueous NaOH (2.0 mL, 5 N). The resulting mixture was heated at 90 °C for 1.5 h, and then cooled to room temperature, diluted with EtOAc (15 mL) and $H_2O$ (20 mL). The aqueous phase was extracted with EtOAc (3×10 mL). The combined organic layers were washed with water (20 mL), brine (20 mL), and dried over $Na_2SO_4$. After filtration and concentration, the crude reduction product pyrrolidine compound was obtained, which was used in the next step without further purification.

**(2) Methylation of Pyrrolidine Compound.** To a stirred solution of pyrrolidine compound in 2,2,2-Trifluoroethanol (2.0 mL), formaldehyde (0.1 mL, 1.2 mmol) and sodium cyanoborohydride (62.0 mg, 1.0 mmol) were added. The obtained mixture was stirred at 78 °C for 24 h. After completion of the reaction as checked by TLC, the solvent was evaporated and the residue was purified directly by flash column chromatograph ($CH_2Cl_2$/MeOH = 95:5) to give **(+)-woodinine (15)** (21.2 mg, 61% yield, 94% ee) as a yellowish solid. The structure and absolute configuration were confirmed by comparing with the reported spectroscopic data as shown in **Table S6** and optical rotation of **woodinine**[13]. **$^1$H NMR** (400 MHz, $C_6D_6$) δ 9.91 (s, 1H), 7.79 (s, 1H), 7.31 (d, *J* = 8.4 Hz, 1H), 6.87 (d, *J* = 8.6 Hz, 1H), 3.42 (br s, 1H), 2.83 – 2.67 (m, 3H), 2.56-2.48 (m, 1H), 2.46-2.33 (m, 2H), 2.25 (s, 3H), 2.11 (s, 3H), 1.82 – 1.72 (m, 1H), 1.65 – 1.55 (m, 1H), 1.50 – 1.40 (m, 1H), 1.39 – 1.25 (m, 2H). **$^{13}$C NMR** (101 MHz, $C_6D_6$) δ 136.0, 134.8, 129.4,124.2, 121.2, 112.7, 112.7, 110.0, 67.2, 61.0, 57.9, 54.3, 43.6, 41.0, 26.1, 23.1, 21.5. **HRMS** (ESI-TOF) m/z: calcd for $C_{17}H_{23}BrN_3^+$ [M + H]$^+$, 348.1070; found, 348.1073. **HPLC analysis:** Chiral MX (2) (150 x 4.6 mm, 3 µm, hexane/*i*-PrOH = 80:20, 1.0 mL/min, 25 °C, 254 nm), $t_r$ (major) = 14.22 min, $t_r$ (minor) = 19.40 min. **[α]$_D$$^{20}$** = +69.2 (c = 0.6, MeOH). Ref. 13: **[α]$_D$$^{23}$** = -81.0 (c = 0.6, MeOH).

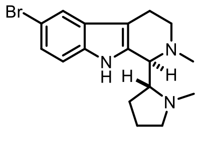


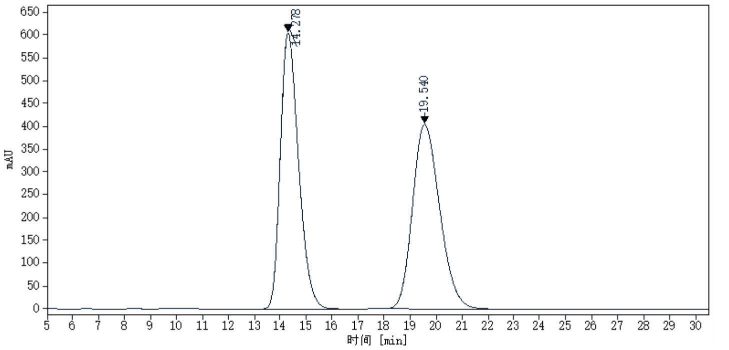


| Entry | Retention Time | Height | Area | Area% |
|---|---|---|---|---|
| 1 | 14.28 | 606.40 | 29608.90 | 50.06 |
| 2 | 19.54 | 405.24 | 29535.41 | 49.94 |

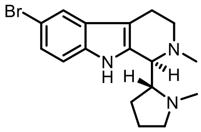

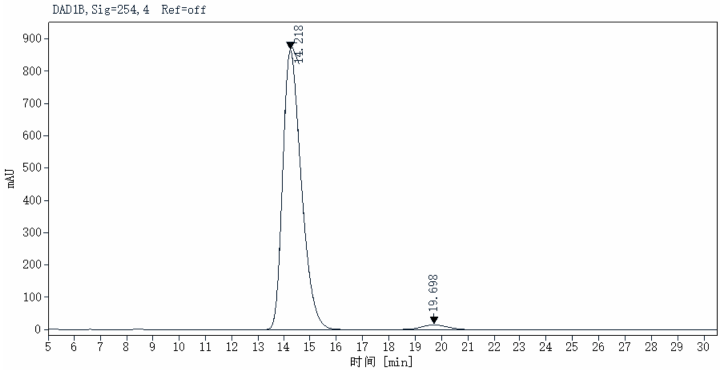


| Entry | Retention Time | Height | Area | Area% |
|---|---|---|---|---|
| 1 | 14.22 | 864.81 | 42649.03 | 97.12 |
| 2 | 19.40 | 15.19 | 1264.72 | 2.88 |

**Table S6**. $^{1}$H and $^{13}$C NMR data for synthetic structure of **woodinine** in Benzene-*d*6

| Position | $\delta_H$ (400 MHz, Benzene-*d*6) | $\delta_H$ (400 MHz, Benzene-*d*6) | $\delta_C$ (100 MH, Benzene-*d*6) | $\delta_C$ (100 MHz, Benzene-*d*6) |
|---|---|---|---|---|
| | Synthetic | Reported[a] | Synthetic | Reported[a] |
| 1 | **3.42 (br s)** | **3.34 (br s)** | 61.03 | 61.05 |
| 2’ | 2.48-2.56 (m) | 2.48-2.55 (m) | 67.47 | 67.20 |
| 3 | 2.33-2.46 (m) | 2.31-2.45 (m) | 54.29 | 54.48 |
| | 2.67-2.83 (m) | 2.68-2.84 (m) | | |
| 3’ | 1.40-1.50(m) | 1.30-1.41 (m) | 26.07 | 25.90 |
| | 1.55-1.65 (m) | 1.56-1.68 (m) | | |
| 4 | 2.33-2.46 (m) | 2.31-2.45 (m) | 21.48 | 21.52 |
| | 2.67-2.83 (m) | 2.68-2.84 (m) | | |
| 4’ | 1.25-1.39(m) | 1.10 (m) | 23.12 | 23.07 |
| | 1.25-1.39(m) | 1.30-1.41 (m) | | |
| 4a | - | - | 109.98 | 109.82 |
| 4b | - | - | 129.37 | 129.27 |
| 5 | 7.79 (s) | 7.78 (s) | 121.21 | 121.05 |
| 5’ | 1.72-1.83 (m) | 1.72-1.84 (m) | 57.85 | 57.80 |
| | 2.67-2.83 (m) | 2.68-2.84 (m) | | |
| 6 | - | - | 112.69 | 112.55 |
| 7 | 7.30-7.32 (d, 8.4) | 7.26-7.30 (d) | 124.20 | 123.99 |
| 8 | 6.86-6.87 (d, 8.6) | 6.84-6.88 (d) | 112.74 | 112.66 |
| 8a | - | - | 136.04 | 136.17 |
| 9a | - | - | 134.84 | 134.72 |
| N-H(indole) | 9.91 (br s) | 9.87 (br s) | - | - |
| N-$CH_3$ (pyrrolidine) | 2.11 (s) | 2.13 (s) | 41.02 | 40.93 |
| N-$CH_3$ (piperideine) | 2.25 (s) | 2.23 (s) | 43.63 | 43.67 |

[a]Data from Ref. 13.

## 10 Mechanistic studies

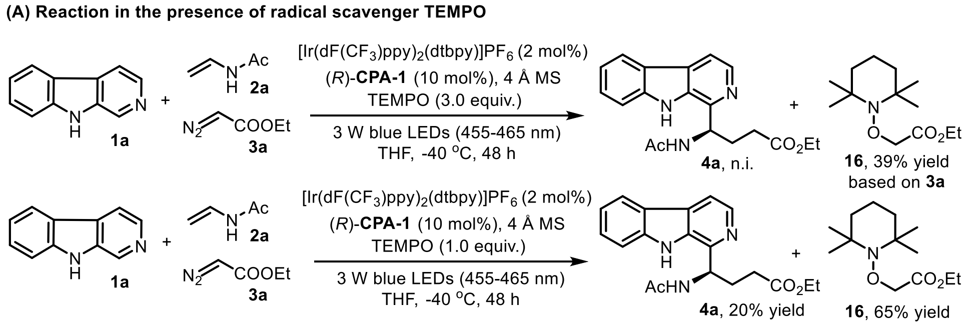


Sequentially, **1a** (0.20 mmol, 1.0 equiv.), **2a** (0.40 mmol, 2.0 equiv.), Ir[dF($CF_3$)ppy)$_2$(dtbpy)]$PF_6$ (4.4 mg, 0.004 mmol, 2 mol%), (*R*)-**CPA-1** (14.4 mg, 0.02 mmol, 10 mol%), TEMPO (3.0 equiv. or 1.0 equiv.) and 4 Å MS (50.0 mg) were added into a 20 mL Schlenk tube containing a stirrer bar. After evacuated and refilled with argon for three times, anhydrous, freshly argon-sparged THF (16.0 mL) was then added via syringe. The diazocarbonyl compounds **3a** (1.0 equiv.) was add once every 12 h by injection for a total of 3 times. The reaction mixture was stirred under an argon atmosphere at −40 °C (the temperature was maintained in an incubator without fan) for 1 h without light, then irradiated by 3 W blue LED (λ = 455−465 nm) for another 48 h. The reaction was monitored by TLC. After completion of the reaction, the solvent was removed in vacuo and the crude residue was purified via flash column chromatography on silica gel using $CH_2Cl_2$/MeOH to give the products **16**[14]. **$^1$H NMR** (400 MHz, CDCl3) δ 4.43 (s, 2H), 4.20 (q, J = 7.1 Hz, 2H), 1.48 – 1.40 (m, 4H), 1.31 – 1.24 (m, 5H), 1.15 (s, 12H).

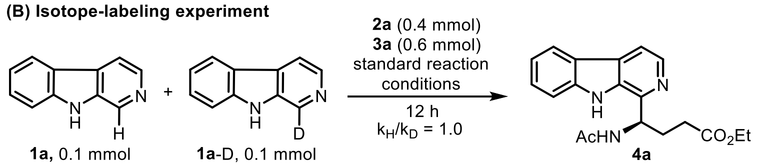


Sequentially, **1a** (0.10 mmol, 1.0 equiv.), **1a**-D (0.10 mmol, 1.0 equiv.), **2a** (0.40 mmol, 2.0 equiv.), Ir[dF($CF_3$)ppy)$_2$(dtbpy)]$PF_6$ (4.4 mg, 0.004 mmol, 2 mol%), (*R*)-**CPA-1** (14.4 mg, 0.02 mmol, 10 mol%) and 4 Å MS (50.0 mg) were added into a 20 mL Schlenk tube containing a stirrer bar. After evacuated and refilled with argon three times, anhydrous, freshly argon-sparged THF (16.0 mL) and **3a** (0.60 mmol, 3.0 equiv.) was then added via syringe. The reaction mixture was stirred under an argon atmosphere at −40 °C (the temperature was maintained in an incubator without fan) for 1 h without light, then irradiated by 3 W blue LED (λ = 455−465 nm) for another 12 h. After completion of the reaction, solvent was removed in vacuo. β-Carboline **1a** and 1-deuterated β-Carboline **1a**-D were recovered after flash chromatography on silica gel ($CH_2Cl_2$:MeOH = 97:3). Analysis of the NMR of the remaining **1a** and **1a**-D showed a consumption ratio of 1:1 from **1a** to **1a**-D. After the reaction, 18.8 mg (0.11 mmol) **1a** to **1a**-D were isolated and the ratio of **1a** / **1a**-D is 1:1 according to $^1$H NMR.

(C) Control experiments

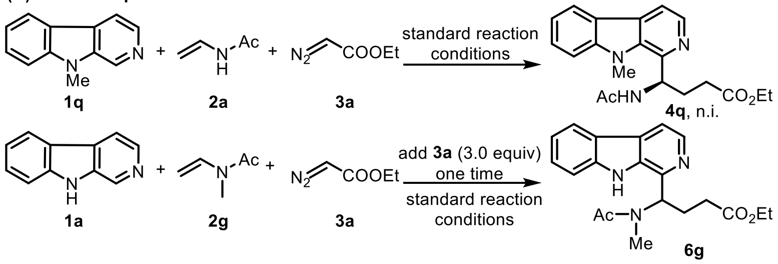


Sequentially, **1q** (0.20 mmol, 1.0 equiv.), **2a** (0.40 mmol, 2.0 equiv.), Ir[dF($CF_3$)ppy)$_2$(dtbpy)]$PF_6$ (4.4 mg, 0.004 mmol, 2 mol%), (*R*)-**CPA-1** (14.4 mg, 0.02 mmol, 10 mol%) and 4 Å MS (50.0 mg) were added to a 20 mL Schlenk tube containing a stirrer bar. After evacuated and refilled with argon three times, anhydrous, freshly argon-sparged THF (16.0 mL) was then added via syringe. The diazocarbonyl compounds **3a** (1.0 equiv.) was add once every 12 h by injection for a total of 3 times. The reaction mixture was stirred under an argon atmosphere at −40 °C (the temperature was maintained in an incubator without fan) for 1 h without light, then irradiated by 3 W blue LED (λ = 455−465 nm) for another 48 h. But the product **4q** was no identified.

Sequentially, **1a** (0.20 mmol, 1.0 equiv.), **2g** (0.40 mmol, 2.0 equiv.), Ir[dF($CF_3$)ppy)$_2$(dtbpy)]$PF_6$ (4.4 mg, 0.004 mmol, 2 mol%), (*R*)-**CPA-1** (14.4 mg, 0.02 mmol, 10 mol%) and 4 Å MS (50.0 mg) were added to a 20 mL Schlenk tube containing a stirrer bar. After evacuated and refilled with argon three times, anhydrous, freshly argon-sparged THF (16.0 mL) and **3a** (0.60 mmol, 3.0 equiv.) was then added via syringe. The reaction mixture was stirred under an argon atmosphere at −40 °C (the temperature was maintained in an incubator without fan) for 1 h without light, then irradiated by 3 W blue LED (λ = 455−465 nm) for another 48 h. After completion of the reaction, the solvent was removed in vacuo and the crude residue was purified via flash column chromatography on silica gel using $CH_2Cl_2$/MeOH to give the products **6g** (13.5 mg, 19% yield, 5% ee) as a white solid. **$^1$H NMR** (400 MHz, $CDCl_3$) δ 9.99 (s, 1H), 8.37 (d, *J* = 5.3 Hz, 1H), 8.10 (d, *J* = 7.9 Hz, 1H), 7.91 (d, *J* = 5.3 Hz, 1H), 7.61 – 7.51 (m, 2H), 7.30 – 7.23 (m, 1H), 6.40 – 6.29 (m, 1H), 4.15 (q, *J* = 7.1 Hz, 2H), 2.85 (s, 3H), 2.77 – 2.66 (m, 1H), 2.63 – 2.52 (m, 1H), 2.51 – 2.40 (m, 1H), 2.40 – 2.29 (m, 1H), 2.13 (s, 3H), 1.26 (s, 3H). **$^{13}$C NMR** (101 MHz, $CDCl_3$) δ 173.0, 172.8, 141.2, 140.4, 135.0, 128.6, 121.7, 120.1, 114.6, 112.4, 60.7, 52.7, 31.4, 30.7, 23.6, 22.2, 14.4. **HRMS** (ESI-TOF) m/z: calcd for $C_{20}H_{24}N_3O_3^+$ [M + H]$^+$, 354.1812; found, 354.1831. **HPLC analysis:** Chiral MX (2) (150 x 4.6 mm, 3 μm, hexane/*i*-PrOH = 80:20, 1.0 mL/min, 25 °C, 254 nm), $t_r$ (major) = 10.72 min, $t_r$ (minor) = 15.44 min.

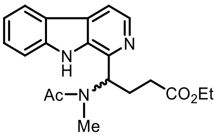


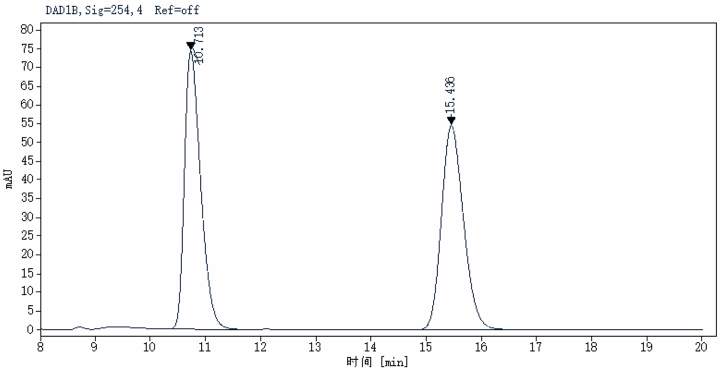


| Entry | Retention Time | Height | Area | Area% |
|---|---|---|---|---|
| 1 | 10.71 | 74.35 | 1514.02 | 49.95 |
| 2 | 15.44 | 54.53 | 1517.08 | 50.05 |

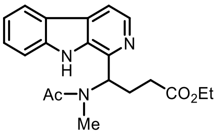


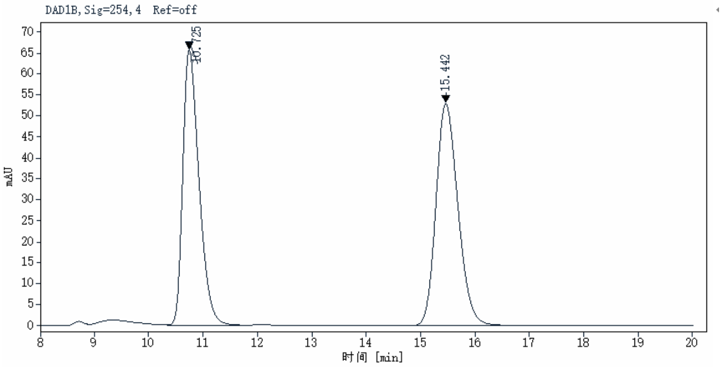


| Entry | Retention Time | Height | Area | Area% |
|---|---|---|---|---|
| 1 | 10.72 | 65.57 | 1485.84 | 52.25 |
| 2 | 15.44 | 52.90 | 1357.78 | 47.72 |

## 11 X-ray data of compound 4a (CDCC number:2292538)

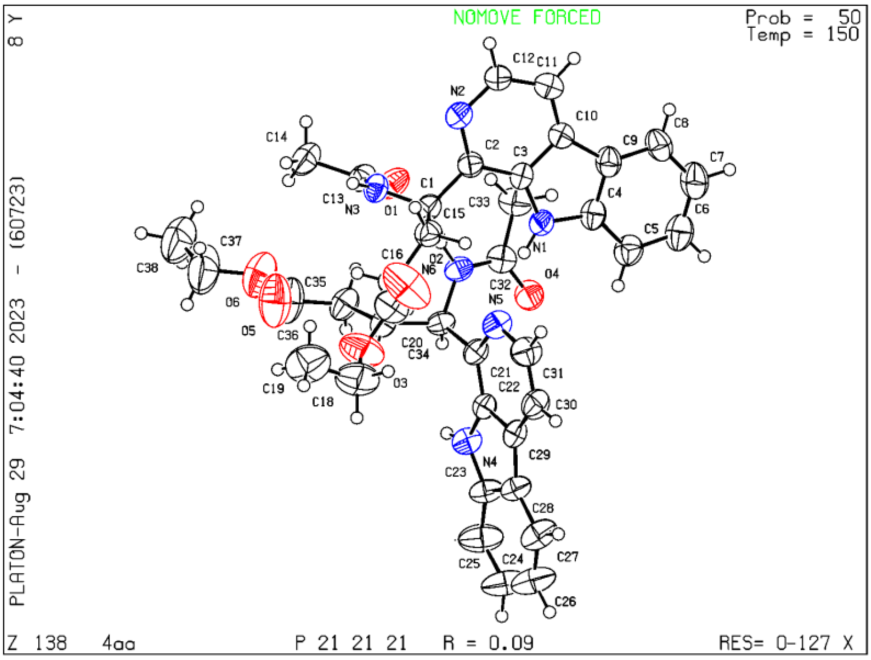


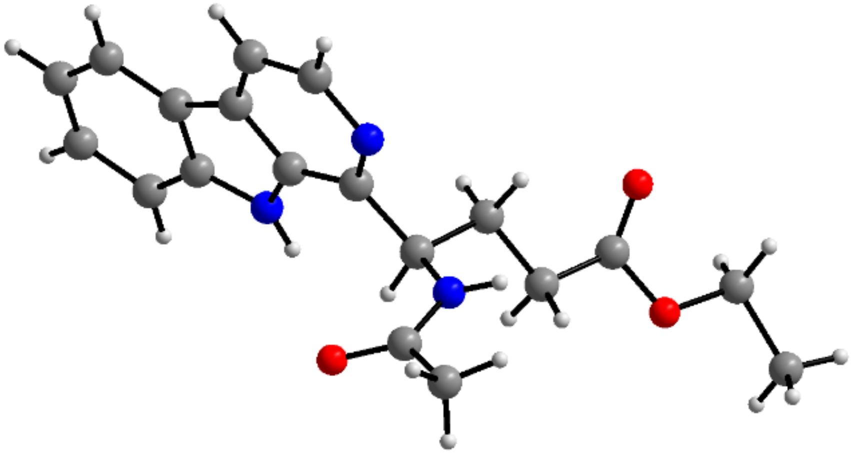

**Table S7**. **Crystal data and structure refinement for 4a.**

| Index | Result |
|---|---|
| Empirical Formula | $C_{19}H_{21}N_3O_3$ |
| Formula weight | 339.397 |
| Temperature/K | 150.00 |
| Crystal system | orthorhombic |
| Space group | $P2_12_12_1$ |
| Unit cell | a/Å 10.888(3) b/Å 16.620(4) c/Å 21.294(5) |
| dimensions | α=90°, β=90°, γ=90° |
| Volume | 3853.5(16)Å3 |
| Z | 8 |
| ρcalcg/cm$^3$ | 1.170 |
| μ/mm$^{-1}$ | 0.655 |
| F(000) | 1445.0 |
| Crystal size/mm$^3$ | 0.3 × 0.04 × 0.04 |
| Radiation | Cu Kα (λ = 1.54178) |
| 2Θ range for data collection/° | 6.74 to 133.44 |
| Index ranges | -12≤ h ≤10, -19≤ k ≤19, -25≤ l ≤23 |
| Reflections collected | 28092 |
| Independent reflections | 6787 [$R_{int}$ = 0.0741, $R_{sigma}$ = 0.0642] |
| Data/restraints/parameters | 6787/0/454 |
| Goodness-of-fit on $F^2$ | 1.007 |
| Final R indexes [I>=2σ (I)] | $R_1$ = 0.0901, $wR_2$ = 0.2442 |
| Final R indexes [all data] | $R_1$ = 0.1151, $wR_2$ = 0.2688 |
| Largest diff. peak/hole / e Å$^{-3}$ | 1.12/-0.51 |
| Flack parameter | 0.22(17) |

**Table S8. Fractional Atomic Coordinates (×10$^4$) and Equivalent Isotropic Displacement Parameters (Å$^2$×10$^3$) for 4a. $U_{eq}$ is defined as 1/3 of the trace of the orthogonalised $U_{IJ}$ tensor.**

| Atom | *x* | *y* | *z* | U(eq) |
|---|---|---|---|---|
| O4 | -6578(3) | -4311.2(18) | -2748.6(17) | 56.8(9) |
| O1 | -5690(4) | -1755.7(19) | -1676.2(15) | 59.7(9) |
| N1 | -7454(3) | -2087(2) | -3397.8(17) | 44.1(9) |
| N6 | -5619(3) | -3182(2) | -2434.1(17) | 43.9(8) |
| N3 | -5059(4) | -664(2) | -2215.9(17) | 45.8(9) |
| N4 | -3102(4) | -4488(2) | -3646.4(17) | 47.2(9) |
| N5 | -5150(4) | -2737(2) | -3700.6(17) | 47.9(9) |
| N2 | -7510(4) | -169(2) | -2531(2) | 56.0(10) |
| C10 | -9070(4) | -1279(3) | -3111(2) | 47.3(11) |
| C32 | -6596(4) | -3657(3) | -2464(2) | 47.7(11) |
| C22 | -3821(4) | -3852(2) | -3816.4(19) | 36.8(9) |
| C29 | -3805(4) | -3762(2) | -4477(2) | 42.2(10) |
| C3 | -7766(4) | -1384(3) | -3101(2) | 41.4(10) |
| C9 | -9546(4) | -1981(3) | -3451(2) | 50.2(11) |
| C4 | -8509(4) | -2450(3) | -3605(2) | 47.6(11) |
| C2 | -7010(4) | -819(3) | -2801(2) | 43.7(10) |
| O3 | -1954(4) | -385(3) | -4042(2) | 96.3(15) |
| C21 | -4492(4) | -3320(2) | -3442.4(19) | 41.0(9) |
| C23 | -2632(5) | -4833(3) | -4187(2) | 51.7(12) |
| C28 | -3038(5) | -4395(3) | -4715(2) | 49.3(11) |
| C13 | -5126(5) | -1106(3) | -1696(2) | 50.7(12) |
| C1 | -5633(4) | -920(3) | -2803(2) | 46.4(11) |
| O5 | -1830(6) | -1915(4) | -1709(3) | 115.2(19) |
| C30 | -4476(5) | -3131(3) | -4733(2) | 54.1(12) |
| C20 | -4455(4) | -3396(3) | -2727.1(19) | 41.5(10) |
| C34 | -3407(4) | -2876(3) | -2467(2) | 46.4(11) |
| C16 | -3698(5) | -670(3) | -3439(3) | 55.2(12) |
| C8 | -10705(4) | -2242(3) | -3611(3) | 57.3(13) |
| C31 | -5118(5) | -2654(3) | -4340(2) | 58.1(13) |
| C11 | -9556(5) | -614(3) | -2832(2) | 58.8(12) |
| O6 | -2211(6) | -2599(3) | -864(2) | 104.1(17) |
| C6 | -9818(5) | -3436(3) | -4074(3) | 63.5(14) |
| C27 | -2661(6) | -4643(3) | -5312(3) | 65.1(15) |
| C5 | -8639(5) | -3193(3) | -3912(2) | 57.4(13) |
| O2 | -3592(5) | 352(4) | -4218(3) | 117(2) |
| C33 | -7736(5) | -3373(3) | -2135(3) | 58.4(13) |
| C15 | -5055(5) | -467(3) | -3359(2) | 48.8(11) |

**Table S8. Fractional Atomic Coordinates (×$10^4$) and Equivalent Isotropic Displacement Parameters (Å$^2$×$10^3$) for 4a. $U_{eq}$ is defined as 1/3 of the trace of the orthogonalised $U_{IJ}$ tensor.**

| Atom | *x* | *y* | *z* | U(eq) |
|---|---|---|---|---|
| C7 | -10835(5) | -2965(4) | -3928(3) | 66.3(15) |
| C12 | -8761(5) | -73(3) | -2557(3) | 63.6(14) |
| C14 | -4479(6) | -769(3) | -1130(2) | 66.0(15) |
| C35 | -3162(5) | -3049(3) | -1763(2) | 57.6(13) |
| C17 | -3133(6) | -209(4) | -3962(3) | 67.8(15) |
| C24 | -1904(7) | -5512(4) | -4250(3) | 81(2) |
| C36 | -2213(7) | -2518(5) | -1488(3) | 87(2) |
| C26 | -1946(7) | -5305(4) | -5373(3) | 84(2) |
| C25 | -1565(7) | -5738(4) | -4856(3) | 89(2) |
| C18 | -1278(7) | -3(5) | -4549(3) | 94(2) |
| C19 | -586(9) | 693(5) | -4304(4) | 107(3) |
| C38 | -1458(10) | -2327(6) | 169(4) | 117(3) |
| C37 | -1291(9) | -2101(6) | -542(4) | 126(3) |

**Table S9**. **Anisotropic Displacement Parameters (Å$^2$×10$^3$) for 4a. The Anisotropic displacement factor exponent takes the form: -2π$^2$ [h$^2$a*$^2$U$_{11}$+2hka*b*U$_{12}$+…].**

| Atom | $U_{11}$ | $U_{22}$ | $U_{33}$ | $U_{12}$ | $U_{13}$ | $U_{23}$ |
|---|---|---|---|---|---|---|
| O4 | 57.3(19) | 37.0(16) | 76(2) | 0.7(15) | -6.4(17) | -3.8(16) |
| O1 | 87(2) | 42.3(18) | 49.9(18) | -11.9(18) | 10.8(17) | -3.8(14) |
| N1 | 45(2) | 39.3(19) | 48(2) | -0.3(17) | -3.3(17) | -6.2(16) |
| N6 | 54(2) | 32.9(17) | 44.5(19) | 1.9(17) | 4.4(17) | -0.8(15) |
| N3 | 51(2) | 37.5(19) | 48(2) | -5.0(17) | -7.5(18) | -2.8(16) |
| N4 | 59(2) | 38.3(19) | 45(2) | 3.2(18) | 3.7(18) | 5.0(16) |
| N5 | 63(2) | 43(2) | 38(2) | 4.5(18) | -0.7(18) | 8.1(15) |
| N2 | 53(2) | 53(2) | 62(3) | 2.6(19) | -4(2) | -17.4(19) |
| C10 | 43(2) | 52(3) | 47(2) | 0(2) | 1(2) | -4(2) |
| C32 | 54(3) | 45(2) | 44(2) | 2(2) | 4(2) | 11(2) |
| C22 | 42(2) | 30(2) | 39(2) | -5.6(17) | 0.0(17) | 1.5(17) |
| C29 | 51(3) | 38(2) | 38(2) | -8(2) | -3.8(19) | -1.9(17) |
| C3 | 44(2) | 41(2) | 39(2) | -4(2) | -4.4(18) | -2.1(18) |
| C9 | 49(2) | 52(3) | 50(3) | -13(2) | -2(2) | 4(2) |
| C4 | 55(3) | 37(2) | 51(3) | -9(2) | -7(2) | 4.8(19) |
| C2 | 45(2) | 38(2) | 48(2) | 1.7(19) | -2.9(19) | -1.2(19) |
| O3 | 75(3) | 119(4) | 95(3) | 12(3) | 36(2) | 25(3) |
| C21 | 50(2) | 35(2) | 38(2) | -3.4(19) | -0.4(19) | -1.0(17) |
| C23 | 66(3) | 36(2) | 52(3) | 4(2) | 19(2) | -2(2) |
| C28 | 61(3) | 40(2) | 47(2) | -1(2) | 12(2) | -6(2) |
| C13 | 64(3) | 38(2) | 50(3) | 6(2) | -5(2) | -7(2) |
| C1 | 48(2) | 43(2) | 49(2) | 1(2) | -5(2) | -9.3(19) |
| O5 | 121(4) | 116(4) | 108(4) | -58(4) | -33(3) | 20(3) |
| C30 | 66(3) | 58(3) | 39(2) | 1(3) | -5(2) | 2(2) |
| C20 | 52(2) | 38(2) | 35(2) | 2.0(19) | 8.6(19) | 2.2(17) |
| C34 | 57(3) | 40(2) | 41(2) | -4(2) | -3(2) | 1.2(18) |
| C16 | 55(3) | 46(3) | 64(3) | 4(2) | 3(2) | 3(2) |
| C8 | 42(3) | 67(3) | 62(3) | -12(2) | 0(2) | 10(3) |
| C31 | 67(3) | 57(3) | 50(3) | 16(3) | -5(2) | 8(2) |
| C11 | 47(3) | 64(3) | 65(3) | 6(2) | 4(2) | -9(3) |
| O6 | 134(4) | 106(4) | 73(3) | -11(3) | -31(3) | -3(3) |
| C6 | 75(4) | 46(3) | 70(3) | -18(3) | -12(3) | 5(2) |
| C27 | 97(4) | 48(3) | 51(3) | -11(3) | 19(3) | -2(2) |
| C5 | 64(3) | 44(3) | 65(3) | -5(2) | -4(3) | -2(2) |
| O2 | 81(3) | 136(4) | 135(5) | 9(3) | 11(3) | 76(4) |
| C33 | 59(3) | 50(3) | 67(3) | 2(2) | 14(2) | 7(2) |
| C15 | 52(3) | 47(2) | 48(2) | 4(2) | -2(2) | -2(2) |

**Table S9**. **Anisotropic Displacement Parameters (Å$^2$×10$^3$) for 4a. The Anisotropic displacement factor exponent takes the form: -2π$^2$ [h$^2$a*$^2$U$_{11}$+2hka*b*U$_{12}$+…].**

| Atom | $U_{11}$ | $U_{22}$ | $U_{33}$ | $U_{12}$ | $U_{13}$ | $U_{23}$ |
|---|---|---|---|---|---|---|
| C7 | 60(3) | 66(3) | 72(4) | -27(3) | -3(3) | 8(3) |
| C12 | 50(3) | 66(3) | 75(4) | 15(3) | -10(3) | -28(3) |
| C14 | 89(4) | 60(3) | 50(3) | 6(3) | -15(3) | -11(2) |
| C35 | 80(3) | 48(3) | 45(3) | -4(3) | -8(2) | 1(2) |
| C17 | 66(3) | 77(4) | 61(3) | 5(3) | 4(3) | 15(3) |
| C24 | 108(5) | 60(3) | 76(4) | 30(3) | 28(4) | 12(3) |
| C36 | 107(5) | 94(5) | 60(4) | -35(4) | -24(4) | 17(3) |
| C26 | 118(5) | 60(3) | 73(4) | 6(4) | 43(4) | -10(3) |
| C25 | 123(6) | 64(4) | 81(4) | 31(4) | 40(4) | -5(3) |
| C18 | 93(5) | 116(6) | 73(4) | 4(4) | 39(4) | 13(4) |
| C19 | 122(6) | 107(6) | 93(5) | -16(5) | 36(5) | 5(4) |
| C38 | 147(8) | 116(6) | 88(5) | 1(6) | -42(6) | -12(5) |
| C37 | 133(7) | 129(7) | 115(7) | -9(6) | -57(6) | -19(6) |

**Table S10. Bond Lengths for 4a.**

| Atom | Atom | Length/Å | Atom | Atom | Length/Å |
|---|---|---|---|---|---|
| O4 | C32 | 1.245(6) | O3 | C17 | 1.327(8) |
| O1 | C13 | 1.244(6) | O3 | C18 | 1.453(8) |
| N1 | C3 | 1.370(6) | C21 | C20 | 1.529(6) |
| N1 | C4 | 1.370(6) | C23 | C28 | 1.409(7) |
| N6 | C32 | 1.326(6) | C23 | C24 | 1.386(8) |
| N6 | C20 | 1.456(6) | C28 | C27 | 1.399(7) |
| N3 | C13 | 1.330(6) | C13 | C14 | 1.505(7) |
| N3 | C1 | 1.461(6) | C1 | C15 | 1.538(7) |
| N4 | C22 | 1.364(6) | O5 | C36 | 1.184(8) |
| N4 | C23 | 1.385(6) | C30 | C31 | 1.349(7) |
| N5 | C21 | 1.324(6) | C20 | C34 | 1.534(6) |
| N5 | C31 | 1.369(6) | C34 | C35 | 1.548(6) |
| N2 | C2 | 1.339(6) | C16 | C15 | 1.525(7) |
| N2 | C12 | 1.373(7) | C16 | C17 | 1.485(8) |
| C10 | C3 | 1.431(6) | C8 | C7 | 1.386(8) |
| C10 | C9 | 1.468(7) | C11 | C12 | 1.379(7) |
| C10 | C11 | 1.362(7) | O6 | C36 | 1.336(8) |
| C32 | C33 | 1.501(7) | O6 | C37 | 1.468(9) |
| C22 | C29 | 1.414(6) | C6 | C5 | 1.389(8) |
| C22 | C21 | 1.397(6) | C6 | C7 | 1.390(9) |
| C29 | C28 | 1.436(6) | C27 | C26 | 1.354(9) |
| C29 | C30 | 1.390(7) | O2 | C17 | 1.190(7) |
| C3 | C2 | 1.403(6) | C35 | C36 | 1.481(8) |
| C9 | C4 | 1.409(7) | C24 | C25 | 1.395(9) |
| C9 | C8 | 1.377(7) | C26 | C25 | 1.379(10) |
| C4 | C5 | 1.404(7) | C18 | C19 | 1.476(11) |
| C2 | C1 | 1.509(7) | C38 | C37 | 1.570(14) |

**Table S11**. **Bond Angles for 4a.**

| Atom | Atom | Atom | Angle/° | Atom | Atom | Atom | Angle/° |
|---|---|---|---|---|---|---|---|
| C4 | N1 | C3 | 108.4(4) | C23 | C28 | C29 | 106.2(4) |
| C20 | N6 | C32 | 122.2(4) | C27 | C28 | C29 | 135.0(5) |
| C1 | N3 | C13 | 121.9(4) | C27 | C28 | C23 | 118.8(5) |
| C23 | N4 | C22 | 108.2(4) | N3 | C13 | O1 | 122.3(4) |
| C31 | N5 | C21 | 118.2(4) | C14 | C13 | O1 | 121.9(4) |
| C12 | N2 | C2 | 118.7(4) | C14 | C13 | N3 | 115.8(4) |
| C9 | C10 | C3 | 105.1(4) | C2 | C1 | N3 | 113.0(4) |
| C11 | C10 | C3 | 118.6(4) | C15 | C1 | N3 | 110.0(4) |
| C11 | C10 | C9 | 136.3(5) | C15 | C1 | C2 | 110.7(4) |
| N6 | C32 | O4 | 122.0(4) | C31 | C30 | C29 | 118.2(4) |
| C33 | C32 | O4 | 120.9(4) | C21 | C20 | N6 | 112.6(4) |
| C33 | C32 | N6 | 117.0(4) | C34 | C20 | N6 | 110.8(3) |
| C29 | C22 | N4 | 109.8(4) | C34 | C20 | C21 | 109.5(3) |
| C21 | C22 | N4 | 129.8(4) | C35 | C34 | C20 | 111.9(4) |
| C21 | C22 | C29 | 120.4(4) | C17 | C16 | C15 | 111.8(4) |
| C28 | C29 | C22 | 106.3(4) | C7 | C8 | C9 | 119.2(5) |
| C30 | C29 | C22 | 117.6(4) | C30 | C31 | N5 | 124.8(4) |
| C30 | C29 | C28 | 136.1(4) | C12 | C11 | C10 | 118.1(5) |
| C10 | C3 | N1 | 110.1(4) | C37 | O6 | C36 | 114.1(7) |
| C2 | C3 | N1 | 129.4(4) | C7 | C6 | C5 | 121.2(5) |
| C2 | C3 | C10 | 120.5(4) | C26 | C27 | C28 | 119.6(6) |
| C4 | C9 | C10 | 105.7(4) | C6 | C5 | C4 | 117.6(5) |
| C8 | C9 | C10 | 134.1(5) | C16 | C15 | C1 | 111.9(4) |
| C8 | C9 | C4 | 120.2(5) | C6 | C7 | C8 | 121.0(5) |
| C9 | C4 | N1 | 110.7(4) | C11 | C12 | N2 | 124.4(5) |
| C5 | C4 | N1 | 128.4(5) | C36 | C35 | C34 | 113.1(4) |
| C5 | C4 | C9 | 120.9(4) | C16 | C17 | O3 | 112.6(5) |
| C3 | C2 | N2 | 119.8(4) | O2 | C17 | O3 | 121.3(6) |
| C1 | C2 | N2 | 119.6(4) | O2 | C17 | C16 | 125.0(6) |
| C1 | C2 | C3 | 120.5(4) | C25 | C24 | C23 | 117.4(6) |
| C18 | O3 | C17 | 119.3(6) | O6 | C36 | O5 | 118.7(6) |
| C22 | C21 | N5 | 120.6(4) | C35 | C36 | O5 | 126.4(6) |
| C20 | C21 | N5 | 119.2(4) | C35 | C36 | O6 | 109.6(6) |
| C20 | C21 | C22 | 120.1(4) | C25 | C26 | C27 | 121.5(5) |
| C28 | C23 | N4 | 109.5(4) | C26 | C25 | C24 | 121.2(6) |
| C24 | C23 | N4 | 129.0(5) | C19 | C18 | O3 | 109.7(6) |
| C24 | C23 | C28 | 121.5(5) | C38 | C37 | O6 | 103.7(8) |

**Table S12. Torsion Angles for 4a.**

| A | B | C | D | Angle/° | A | B | C | D | Angle/° |
|---|---|---|---|---|---|---|---|---|---|
| O4 | C32 | N6 | C20 | -0.5(5) | N2 | C12 | C11 | C10 | -1.6(7) |
| O1 | C13 | N3 | C1 | -0.9(6) | C10 | C3 | C2 | C1 | -178.7(4) |
| N1 | C3 | C10 | C9 | -0.7(4) | C10 | C9 | C4 | C5 | 177.2(4) |
| N1 | C3 | C10 | C11 | 179.4(4) | C10 | C9 | C8 | C7 | -178.2(6) |
| N1 | C3 | C2 | N2 | -179.2(5) | C22 | C29 | C28 | C23 | 0.0(4) |
| N1 | C3 | C2 | C1 | 3.2(5) | C22 | C29 | C28 | C27 | 179.3(4) |
| N1 | C4 | C9 | C10 | -1.1(4) | C22 | C29 | C30 | C31 | -1.3(5) |
| N1 | C4 | C9 | C8 | -179.4(4) | C22 | C21 | C20 | C34 | 90.4(4) |
| N1 | C4 | C5 | C6 | 179.7(5) | C29 | C28 | C23 | C24 | 177.5(5) |
| N6 | C20 | C21 | N5 | 35.3(4) | C29 | C28 | C27 | C26 | -177.5(7) |
| N6 | C20 | C21 | C22 | -145.9(3) | C3 | C2 | C1 | C15 | 92.7(4) |
| N6 | C20 | C34 | C35 | 66.0(4) | C9 | C4 | C5 | C6 | 1.9(5) |
| N3 | C1 | C2 | N2 | 39.0(5) | C9 | C8 | C7 | C6 | 1.3(6) |
| N3 | C1 | C2 | C3 | -143.5(4) | C4 | C5 | C6 | C7 | -1.0(6) |
| N3 | C1 | C15 | C16 | 64.1(4) | C2 | C1 | C15 | C16 | -170.4(4) |
| N4 | C22 | C29 | C28 | 0.8(4) | O3 | C17 | C16 | C15 | -179.5(5) |
| N4 | C22 | C29 | C30 | -179.0(4) | C21 | C20 | C34 | C35 | -169.2(4) |
| N4 | C22 | C21 | N5 | -179.5(5) | C23 | C28 | C27 | C26 | 1.8(6) |
| N4 | C22 | C21 | C20 | 1.7(5) | C23 | C24 | C25 | C26 | -0.5(9) |
| N4 | C23 | C28 | C29 | -0.9(4) | C28 | C27 | C26 | C25 | -0.9(7) |
| N4 | C23 | C28 | C27 | 179.7(4) | C1 | C15 | C16 | C17 | -177.9(4) |
| N4 | C23 | C24 | C25 | 179.3(7) | O5 | C36 | O6 | C37 | -24.5(10) |
| N5 | C21 | C22 | C29 | 1.8(5) | O5 | C36 | C35 | C34 | 15.1(10) |
| N5 | C21 | C20 | C34 | -88.4(4) | C20 | C34 | C35 | C36 | -176.1(5) |
| N5 | C31 | C30 | C29 | 0.9(7) | C34 | C35 | C36 | O6 | 168.8(6) |
| N2 | C2 | C3 | C10 | -1.1(5) | C8 | C7 | C6 | C5 | -0.6(7) |
| N2 | C2 | C1 | C15 | -84.9(5) | C27 | C26 | C25 | C24 | 0.3(9) |

**Table S13**. **Hydrogen Atom Coordinates (Å×10[4]) and Isotropic Displacement Parameters (Å[2]×10[3]) for 4a.**

| Atom | *x* | *y* | *z* | U(eq) |
|---|---|---|---|---|
| H1 | -6704(3) | -2273(2) | -3447.1(17) | 53.0(10) |
| H6 | -5675(3) | -2723(2) | -2230.4(17) | 52.7(10) |
| H3 | -4658(4) | -204(2) | -2205.8(17) | 54.9(11) |
| H4 | -2962(4) | -4651(2) | -3259.8(17) | 56.6(11) |
| H1a | -5453(4) | -1505(3) | -2860(2) | 55.7(13) |
| H30 | -4482(5) | -3039(3) | -5173(2) | 65.0(14) |
| H20 | -4273(4) | -3970(3) | -2621.4(19) | 49.8(12) |
| H34a | -2650(4) | -2983(3) | -2710(2) | 55.7(13) |
| H34b | -3618(4) | -2301(3) | -2520(2) | 55.7(13) |
| H16a | -3257(5) | -548(3) | -3044(3) | 66.2(14) |
| H16b | -3611(5) | -1253(3) | -3523(3) | 66.2(14) |
| H8 | -11406(4) | -1930(3) | -3506(3) | 68.7(16) |
| H31 | -5584(5) | -2229(3) | -4520(2) | 69.8(15) |
| H11 | -10418(5) | -525(3) | -2828(2) | 70.5(15) |
| H6a | -9931(5) | -3931(3) | -4288(3) | 76.1(17) |
| H27 | -2904(6) | -4348(3) | -5674(3) | 78.1(18) |
| H5 | -7945(5) | -3518(3) | -4005(2) | 68.9(15) |
| H33a | -7940(20) | -3746(13) | -1794(12) | 87.6(19) |
| H33b | -7596(13) | -2835(10) | -1962(16) | 87.6(19) |
| H33c | -8418(10) | -3350(20) | -2435(5) | 87.6(19) |
| H15a | -5144(5) | 120(3) | -3291(2) | 58.6(13) |
| H15b | -5502(5) | -608(3) | -3749(2) | 58.6(13) |
| H7 | -11631(5) | -3142(4) | -4048(3) | 79.6(18) |
| H12 | -9102(5) | 397(3) | -2372(3) | 76.3(17) |
| H14a | -3940(30) | -1181(9) | -949(12) | 99(2) |
| H14b | -3980(30) | -303(16) | -1255(4) | 99(2) |
| H14c | -5087(6) | -600(20) | -817(9) | 99(2) |
| H35a | -3937(5) | -2981(3) | -1526(2) | 69.1(15) |
| H35b | -2898(5) | -3616(3) | -1716(2) | 69.1(15) |
| H24 | -1646(7) | -5813(4) | -3894(3) | 98(2) |
| H26 | -1703(7) | -5475(4) | -5780(3) | 100(2) |
| H25 | -1061(7) | -6198(4) | -4915(3) | 107(3) |
| H18a | -703(7) | -395(5) | -4740(3) | 113(3) |
| H18b | -1857(7) | 178(5) | -4879(3) | 113(3) |
| H19a | -50(50) | 517(9) | -3960(20) | 160(4) |
| H19b | -80(50) | 920(30) | -4640(9) | 160(4) |
| H19c | -1162(9) | 1100(17) | -4150(30) | 160(4) |

| Atom | *x* | *y* | *z* | U(eq) |
|---|---|---|---|---|
| H38a | -2310(20) | -2230(40) | 295(10) | 176(4) |
| H38b | -1270(70) | -2898(12) | 229(7) | 176(4) |
| H38c | -900(50) | -2000(30) | 425(5) | 176(4) |
| H37a | -452(9) | -2232(6) | -689(4) | 151(4) |
| H37b | -1448(9) | -1522(6) | -613(4) | 151(4) |

## 12 Characterization of starting materials

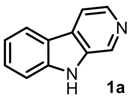 1a 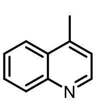 1o 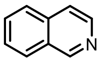 1p

**9*H*-pyrido[3,4-*b*]indole 1a**, **4-methylquinoline 1o, isoquinoline 1p** were purchased from Bidepharm.

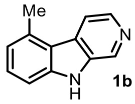

1b

**5-methyl-9*H*-pyrido[3,4-*b*]indole** (**1b**)[2]: **$^1$H NMR** (400 MHz, DMSO-$d_6$) δ 11.63 (s, 1H), 8.90 (s, 1H), 8.34 (d, *J* = 5.3 Hz, 1H), 8.06 (d, *J* = 5.3 Hz, 1H), 7.43 (d, *J* = 4.3 Hz, 2H), 7.03 (t, *J* = 4.2 Hz, 1H), 2.82 (s, 3H).

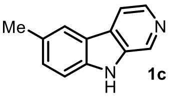

1c

**6-methyl-9*H*-pyrido[3,4-*b*]indole** (**1c**)[2]: **$^1$H NMR** (400 MHz, DMSO-$d_6$) δ 11.47 (s, 1H), 8.85 (s, 1H), 8.30 (d, *J* = 5.3 Hz, 1H), 8.05 (d, *J* = 5.2 Hz, 1H), 8.02 (s, 1H), 7.48 (d, *J* = 8.3 Hz, 1H), 7.37 (dd, *J* = 8.4, 1.7 Hz, 1H), 2.48 (s, 3H).

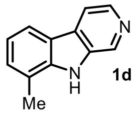

1d

**8-methyl-9*H*-pyrido[3,4-*b*]indole** (**1d**)[2]: **$^1$H NMR** (400 MHz, DMSO-$d_6$) δ 11.56 (s, 1H), 8.91 (s, 1H), 8.33 (d, *J* = 5.2 Hz, 1H), 8.06 (dd, *J* = 9.9, 6.6 Hz, 2H), 7.35 (d, *J* = 7.1 Hz, 1H), 7.15 (t, *J* = 7.5 Hz, 1H), 2.58 (s, 3H).

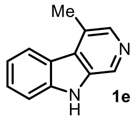

1e

**4-methyl-9*H*-pyrido[3,4-*b*]indole** (**1e**)[2]: **$^1$H NMR** (400 MHz, DMSO-$d_6$) δ 11.63 (s, 1H), 8.75 (s, 1H), 8.20 (d, *J* = 7.9 Hz, 1H), 8.14 (s, 1H), 7.61 (d, *J* = 8.2 Hz, 1H), 7.54 (ddd, *J* = 8.2, 6.9, 1.2 Hz, 1H), 7.26 (ddd, *J* = 8.0, 7.0, 1.2 Hz, 1H), 2.78 (s, 3H).

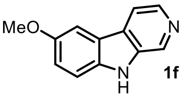

1f

**6-methoxy-9*H*-pyrido[3,4-*b*]indole** (**1f**)[2]: **$^1$H NMR** (400 MHz, DMSO-$d_6$) δ 11.41 (s, 1H), 8.86 (s, 1H), 8.44 – 8.18 (m, 1H), 8.07 (d, *J* = 5.1 Hz, 1H), 7.78 (s, 1H), 7.50 (d, *J* = 8.8 Hz, 1H), 7.19 (d, *J* = 9.0 Hz, 1H), 3.86 (s, 3H).

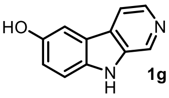

1g

**9*H*-pyrido[3,4-*b*]indol-6-ol** (**1g**)[2]: **$^1$H NMR** (400 MHz, DMSO-$d_6$) δ 12.43 (s, 1H), 9.63 (s, 1H), 9.24 (s, 1H), 8.71 (d, *J* = 6.2 Hz, 1H), 8.50 (d, *J* = 6.2 Hz, 1H), 7.75 (d, *J* = 2.4 Hz, 1H), 7.67 (d, *J* = 8.9 Hz, 1H), 7.35 (dd, *J* = 8.9, 2.4 Hz, 1H).

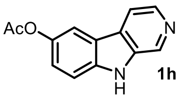

**1h**

**9*H*-pyrido[3,4-*b*]indol-6-yl acetate** (**1h**): **$^{1}$H NMR** (400 MHz, DMSO-$d_6$) δ 11.69 (s, 1H), 8.93 (s, 1H), 8.34 (d, *J* = 5.3 Hz, 1H), 8.10 (d, *J* = 5.3 Hz, 1H), 8.02 (d, *J* = 2.3 Hz, 1H), 7.61 (d, *J* = 8.8 Hz, 1H), 7.31 (dd, *J* = 8.8, 2.3 Hz, 1H), 2.32 (s, 3H); **$^{13}$C NMR** (101 MHz, DMSO) δ 170.35, 143.98, 138.67, 138.51, 137.14, 134.81, 127.72, 123.05, 121.24, 115.29, 114.84, 112.87, 21.52, 21.32; **HRMS** (ESI-TOF) m/z: calcd for $C_{13}H_{11}N_2O_2^+$ $[M + H]^+$, 227.0815; found, 227.0818.

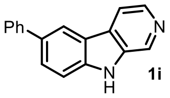

**1i**

**6-phenyl-9*H*-pyrido[3,4-*b*]indole** (**1i**)[2]: **$^{1}$H NMR** (400 MHz, DMSO-$d_6$) δ 11.69 (s, 1H), 8.93 (s, 1H), 8.63 – 8.51 (m, 1H), 8.37 (d, *J* = 5.2 Hz, 1H), 8.20 (d, *J* = 5.3 Hz, 1H), 7.87 (dd, *J* = 8.5, 1.9 Hz, 1H), 7.78 (d, *J* = 7.7 Hz, 2H), 7.68 (d, *J* = 8.5 Hz, 1H), 7.49 (t, *J* = 7.5 Hz, 2H), 7.35 (t, *J* = 7.4 Hz, 1H).

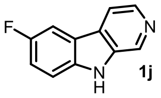

**1j**

**6-fluoro-9*H*-pyrido[3,4-*b*]indole** (**1j**)[2]: **$^{1}$H NMR** (400 MHz, DMSO-$d_6$) δ 11.66 (s, 1H), 8.92 (d, *J* = 1.1 Hz, 1H), 8.33 (d, *J* = 5.2 Hz, 1H), 8.15 – 8.05 (m, 2H), 7.61 (dd, *J* = 8.9, 4.4 Hz, 1H), 7.41 (td, *J* = 9.2, 2.7 Hz, 1H).

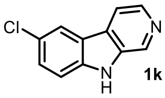

**1k**

**6-chloro-9*H*-pyrido[3,4-*b*]indole** (**1k**)[2]: **$^{1}$H NMR** (400 MHz, DMSO-$d_6$) δ 11.78 (s, 1H), 9.01 – 8.85 (m, 1H), 8.47 – 8.28 (m, 2H), 8.15 (d, *J* = 5.3 Hz, 1H), 7.62 (d, *J* = 8.7 Hz, 1H), 7.55 (dd, *J* = 8.7, 2.1 Hz, 1H).

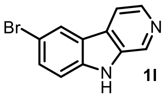

**1l**

**6-bromo-9*H*-pyrido[3,4-*b*]indole** (**1l**)[2]: **$^{1}$H NMR** (400 MHz, DMSO-$d_6$) δ 11.79 (s, 1H), 8.93 (d, *J* = 1.1 Hz, 1H), 8.52 (d, *J* = 2.0 Hz, 1H), 8.36 (d, *J* = 5.3 Hz, 1H), 8.16 (d, *J* = 5.2 Hz, 1H), 7.66 (dd, *J* = 8.7, 2.0 Hz, 1H), 7.57 (d, *J* = 8.7 Hz, 1H).

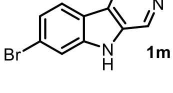

**1m**

**7-bromo-9*H*-pyrido[3,4-*b*]indole** (**1m**)[2]: **$^{1}$H NMR** (400 MHz, DMSO-$d_6$) δ 11.73 (s, 1H), 8.93 (d, *J* = 1.1 Hz, 1H), 8.37 (d, *J* = 5.2 Hz, 1H), 8.21 (d, *J* = 8.4 Hz, 1H), 8.13 (d, *J* = 5.3 Hz, 1H), 7.80 (d, *J* = 1.7 Hz, 1H), 7.39 (dd, *J* = 8.4, 1.8 Hz, 1H).

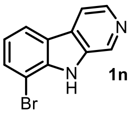

**1n**

**8-bromo-9*H*-pyrido[3,4-*b*]indole** (**1n**)[2]: **$^{1}$H NMR** (400 MHz, DMSO-$d_6$) δ 11.81 (s, 1H), 8.96 (d, *J* = 1.1 Hz, 1H), 8.39 (d, *J* = 5.3 Hz, 1H), 8.28 (d, *J* = 7.8 Hz, 1H), 8.14 (dd, *J* = 5.3, 1.1 Hz, 1H), 7.78 (dd, *J* = 7.7, 1.0 Hz, 1H), 7.20 (t, *J* = 7.8 Hz, 1H).

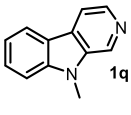
1q

**9-methyl-9*H*-pyrido[3,4-b]indole (1q)**[15]: **$^1$H NMR** (400 MHz, DMSO) δ 9.04 (s, 1H), 8.39 (d, *J* = 5.2 Hz, 1H), 8.27 (d, *J* = 7.8 Hz, 1H), 8.13 (d, *J* = 5.2 Hz, 1H), 7.69 (d, *J* = 8.3 Hz, 1H), 7.62 (t, *J* = 7.0 Hz, 1H), 7.28 (t, *J* = 6.8 Hz, 1H), 3.98 (s, 3H).

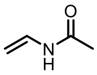 2a 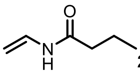 2b 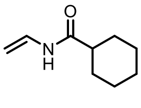 2c 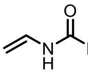 2d 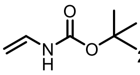 2e 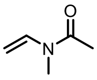 2g

**Enamides 2a**, **2b**, **2c**, **2d**, **2e** and **2g**, were purchased from Adamas-Beta.

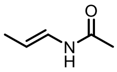
2f

***N*-(prop-1-en-1-yl)acetamide (2f)**[6]: (*E*:*Z*=10:1) **$^1$H NMR** (400 MHz, $CDCl_3$) δ 7.06 (s, 1H), 6.70 (ddq, *J* = 10.8, 9.0, 1.8 Hz, 1H), 4.83 – 4.73 (m, 1H), 2.07 (s, 3H), 1.61 (dd, *J* = 7.1, 1.8 Hz, 3H).

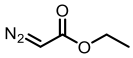 3a 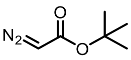 3d 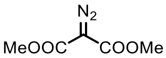 3p

**Diazo Compounds** (**3a**), (**3d**), (**3p**) was purchased from Adamas-Beta.

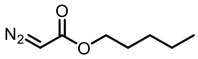
3b

**pentyl 2-diazoacetate (3b)**[16]: **$^1$H NMR** (400 MHz, $CDCl_3$) δ 4.73 (s, 1H), 4.15 (t, *J* = 6.7 Hz, 2H), 1.69 – 1.59 (m, 2H), 1.33 (dh, *J* = 6.8, 3.5, 3.1 Hz, 4H), 0.90 (t, *J* = 6.8 Hz, 3H).

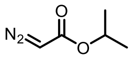
3c

**isopropyl 2-diazoacetate (3c)**[17]: **$^1$H NMR** (400 MHz, $CDCl_3$) δ 5.09 (hept, *J* = 6.3 Hz, 1H), 4.69 (s, 1H), 1.25 (d, *J* = 6.2 Hz, 6H).

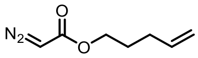
3e

**pent-4-en-1-yl 2-diazoacetate (3e)**[17]: **$^1$H NMR** (400 MHz, DMSO) δ 5.81 (ddt, *J* = 16.9, 10.2, 6.6 Hz, 1H), 5.56 (s, 1H), 5.10 – 4.91 (m, 2H), 4.09 (t, *J* = 6.6 Hz, 2H), 2.06 (qd, *J* = 7.9, 7.5, 1.5 Hz, 2H), 1.74 – 1.60 (m, 2H).

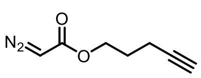
3f

**pent-4-yn-1-yl 2-diazoacetate (3f)**[18]: **$^1$H NMR** (400 MHz, DMSO) δ 5.56 (s, 1H), 4.14 (t, *J* = 6.4 Hz, 2H), 2.80 (t, *J* = 2.7 Hz, 1H), 2.22 (td, *J* = 7.0, 2.7 Hz, 2H), 1.75 (p, *J* = 6.7 Hz, 2H).

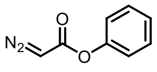
3g

**phenyl 2-diazoacetate (3g)**[16]: **$^1$H NMR** (400 MHz, $CDCl_3$) δ 7.43 – 7.34 (m, 2H), 7.26 – 7.21 (m, 1H), 7.19 – 7.09 (m, 2H), 4.97 (s, 1H).

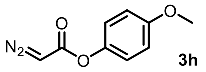
3h

**4-methoxyphenyl 2-diazoacetate (3h)**[19]: **$^1$H NMR** (400 MHz, $CDCl_3$) δ 7.04 (d, *J* = 8.4 Hz, 2H), 6.89 (d, *J* = 8.4 Hz, 2H), 4.94 (s, 1H), 3.80 (s, 3H).

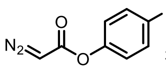

3i

**4-fluorophenyl 2-diazoacetate** (**3i**)[20]: **$^1$H NMR** (400 MHz, $CDCl_3$) δ 7.10 (ttd, *J* = 9.2, 6.7, 5.8, 2.7 Hz, 4H), 5.00 (s, 1H).

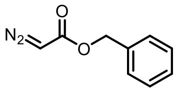

3j

**benzyl 2-diazoacetate** (**3j**)[16]: **$^1$H NMR** (400 MHz, $CDCl_3$) δ 7.41 – 7.30 (m, 5H), 5.20 (s, 2H), 4.80 (s, 1H).

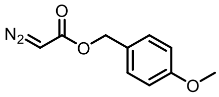

3k

**4-methoxybenzyl 2-diazoacetate** (**3k**)[21]: **$^1$H NMR** (400 MHz, DMSO) δ 7.35 – 7.23 (m, 2H), 6.97 – 6.87 (m, 2H), 5.60 (s, 1H), 5.08 (s, 2H), 3.75 (s, 3H).

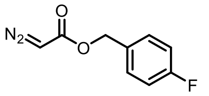

3l

**4-fluorobenzyl 2-diazoacetate** (**3l**)[21]: **$^1$H NMR** (400 MHz, DMSO) δ 7.48 – 7.35 (m, 2H), 7.26 – 7.13 (m, 2H), 5.64 (s, 1H), 5.14 (s, 2H).

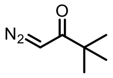

3m

**1-diazo-3,3-dimethylbutan-2-one** (**3m**)[22]: **$^1$H NMR** (400 MHz, $CDCl_3$) δ 5.41 (s, 1H), 1.14 (s, 9H).

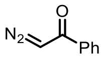

3n

**2-diazo-1-phenylethan-1-one** (**3n**)[22]: **$^1$H NMR** (400 MHz, DMSO) δ 7.88 – 7.80 (m, 2H), 7.65 – 7.58 (m, 1H), 7.56 – 7.47 (m, 2H), 6.97 (s, 1H).

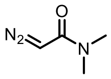

3o

**2-diazo-*N*,*N*-dimethylacetamide** (**3o**)[23]: **$^1$H NMR** (400 MHz, $CDCl_3$) δ 4.96 (s, 1H), 2.89 (s, 6H).

## 13 Characterization of products

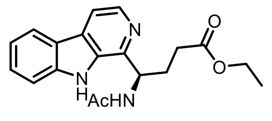

4a

($R$)-ethyl-4-acetamido-4-(9$H$-pyrido[3,4-$b$]indol-1-yl)butanoate (**4a**) (purification by flash column chromatography: 3% MeOH in $CH_2Cl_2$, 80% yield; 92% ee). **Mp**: 146−148 °C. **$^1$H NMR** (400 MHz, $CDCl_3$) δ 10.64 (s, 1H), 8.33 (d, $J$ = 5.2 Hz, 1H), 8.11 (d, $J$ = 7.9 Hz, 1H), 7.90 (d, $J$ = 5.2 Hz, 1H), 7.58 (dd, $J$ = 15.8, 7.7 Hz, 2H), 7.41 (d, $J$ = 8.2 Hz, 1H), 7.28 (d, $J$ = 7.4 Hz, 1H), 5.81 (td, $J$ = 6.7 Hz, 1H), 4.14 (q, $J$ = 7.3 Hz, 2H), 2.68 – 2.55 (m, 1H), 2.52 – 2.34 (m, 2H), 2.25 – 2.16 (m, 1H), 2.12 (s, 3H), 1.22 (t, $J$ = 7.3 Hz, 3H). **$^{13}$C NMR** (101 MHz, $CDCl_3$) δ 174.2, 170.8, 143.2, 140.8, 137.4, 133.8, 129.3, 128.5, 121.6, 121.6, 119.9, 114.3, 112.2, 60.8, 49.5, 30.8, 30.0, 23.5, 14.2. **FTIR** ($\nu_{max}$, $cm^{-1}$): 3351, 3199, 2983, 2905, 2359, 2337, 2161, 1723, 1648, 1541, 1500, 1450, 1398, 1244, 1055, 741, 670, 593. **HRMS** (ESI-TOF) m/z: calcd for $C_{19}H_{22}N_3O_3^+$ [M + H]$^+$, 340.1656; found, 340.1653. **HPLC analysis:** Chiral MX(2) (150 x 4.6 mm, 3 µm, hexane/i-PrOH = 80:20, 1.0 mL/min, 25 °C, 254 nm), $t_r$ (major) = 6.09 min, $t_r$ (minor) = 18.32 min. **[α]$_D^{20}$** = -33.5 (c = 0.2, $CHCl_3$).

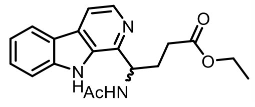

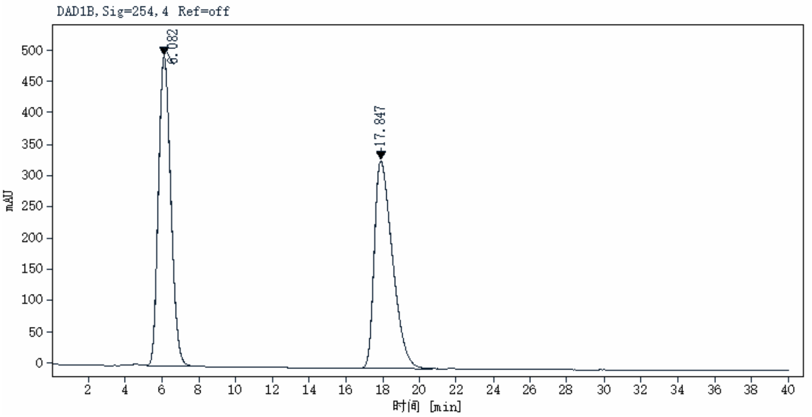


| Entry | Retention Time | Height | Area | Area% |
|---|---|---|---|---|
| 1 | 6.08 | 494.04 | 23101.90 | 50.47 |
| 2 | 17.85 | 331.46 | 22675.11 | 49.53 |

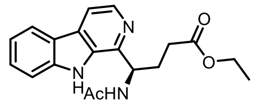

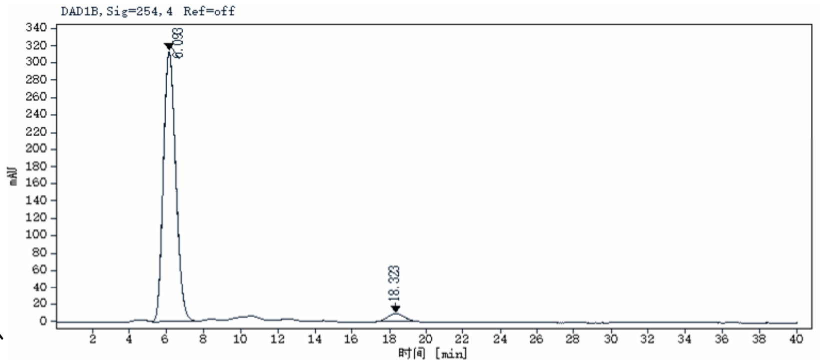


| Entry | Retention Time | Height | Area | Area% |
|---|---|---|---|---|
| 1 | 6.09 | 313.36 | 14594.71 | 96.14 |
| 2 | 18.32 | 9.28 | 586.28 | 3.86 |

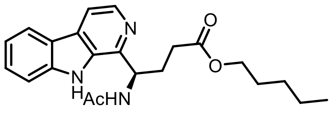

**4b**

($R$)-pentyl-4-acetamido-4-(9$H$-pyrido[3,4-$b$]indol-1-yl)butanoate (**4b**) (purification by flash column chromatography: 3% MeOH in $CH_2Cl_2$, 77% yield; 91% ee). **Mp:** 172−175 °C. **$^1$H NMR** (400 MHz, $CDCl_3$) δ 10.73 (s, 1H), 8.31 (d, $J$ = 5.3 Hz, 1H), 8.09 (d, $J$ = 7.9 Hz, 1H), 7.88 (d, $J$ = 5.3 Hz, 1H), 7.66 – 7.44 (m, 3H), 7.34 – 7.20 (m, 1H), 5.86 (td, $J$ = 8.1, 4.4 Hz, 1H), 4.14 – 4.01 (m, 2H), 2.71 – 2.58 (m, 1H), 2.56 – 2.37 (m, 2H), 2.34 – 2.20 (m, 1H), 2.11 (s, 3H), 1.68 – 1.54 (m, 2H), 1.37 – 1.26 (m, 4H), 0.88 (t, $J$ = 6.8 Hz, 3H). **$^{13}$C NMR** (101 MHz, $CDCl_3$) δ 174.3, 170.8, 143.1, 140.9, 137.2, 133.7, 129.3, 128.5, 121.6, 121.5, 119.9, 114.2, 112.2, 65.0, 49.6, 30.7, 30.0, 28.2, 28.0, 23.5, 23.4, 22.3, 14.0. **FTIR** ($\nu_{max}$, cm$^{-1}$): 3218, 3090, 2930, 2879, 2392, 2323, 1652, 1627, 1554, 1505, 1432, 1370, 1321, 1243, 1071, 821, 738, 626, 577. **HRMS** (ESI-TOF) m/z: calcd for $C_{22}H_{28}N_3O_3^+$ [M + H]$^+$, 382.2125; found, 382.2122. **HPLC analysis:** Chiral MX(2) (150 x 4.6 mm, 3 µm, hexane/i-PrOH = 80:20, 1.0 mL/min, 25 ºC, 254 nm), $t_r$ (major) = 6.00 min, $t_r$ (minor) = 28.65 min. **[α]$_D^{20}$** = -35.1 (c = 0.2, $CHCl_3$).

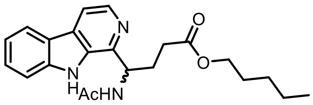

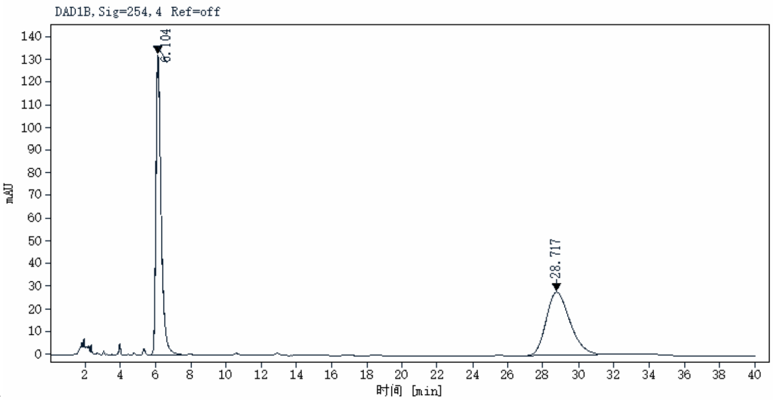


| Entry | Retention Time | Height | Area | Area% |
|---|---|---|---|---|
| 1 | 6.10 | 132.41 | 2720.34 | 51.14 |
| 2 | 28.72 | 27.84 | 2598.89 | 48.86 |

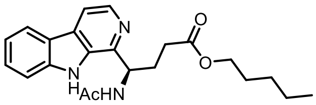

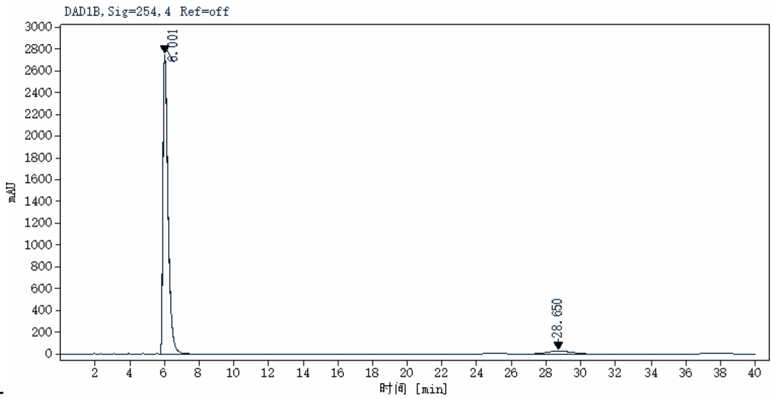


| Entry | Retention Time | Height | Area | Area% |
|---|---|---|---|---|
| 1 | 6.00 | 2749.03 | 55828.46 | 95.59 |
| 2 | 28.65 | 26.94 | 2576.50 | 4.41 |

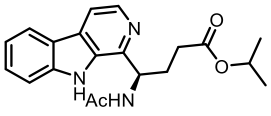


4c

($R$)-isopropyl-4-acetamido-4-(9$H$-pyrido[3,4-$b$]indol-1-yl)butanoate (**4c**) (purification by flash column chromatography: 3% MeOH in $CH_2Cl_2$, 75% yield; 92% ee). **Mp:** 170−174 °C. **$^1$H NMR** (400 MHz, $CDCl_3$) δ 10.65 (s, 1H), 8.33 (d, $J$ = 5.4 Hz, 1H), 8.11 (d, $J$ = 7.9 Hz, 1H), 7.90 (d, $J$ = 5.3 Hz, 1H), 7.57 (dt, $J$ = 15.1, 8.1 Hz, 2H), 7.46 (d, $J$ = 8.3 Hz, 1H), 7.29 (d, $J$ = 6.7 Hz, 1H), 5.80 (td, $J$ = 8.4, 4.5 Hz, 1H), 5.05 (hept, $J$ = 6.2 Hz, 1H), 2.63 – 2.53 (m, 1H), 2.49 – 2.32 (m, 2H), 2.25 – 2.15 (m, 1H), 2.10 (s, 3H), 1.22 (dd, $J$ = 9.9, 6.2 Hz, 6H). **$^{13}$C NMR** (101 MHz, $CDCl_3$) δ 173.7, 170.8, 143.2, 140.9, 137.1, 133.7, 129.4, 128.6, 121.6, 121.5, 119.9, 114.3, 112.2, 68.3, 49.4, 31.1, 30.0, 23.5, 21.8. **FTIR** ($\nu_{max}$, cm$^{-1}$): 3649, 3175, 2993, 2899, 2357, 2345, 1655, 1539, 1503, 1459, 1314, 1239, 1058, 874, 738, 564. **HRMS** (ESI-TOF) m/z: calcd for $C_{20}H_{24}N_3O_3^+$ [M + H]$^+$, 354.1812; found, 354.1809. **HPLC analysis:** Chiral MX(2) (150 x 4.6 mm, 3 µm, hexane/i-PrOH = 80:20, 1.0 mL/min, 25 ºC, 254 nm), $t_r$ (major) = 5.61 min, $t_r$ (minor) = 15.58 min. **$[\alpha]_D^{20}$** = -30.3 (c = 0.2, $CHCl_3$).

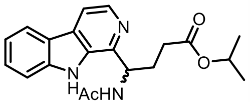


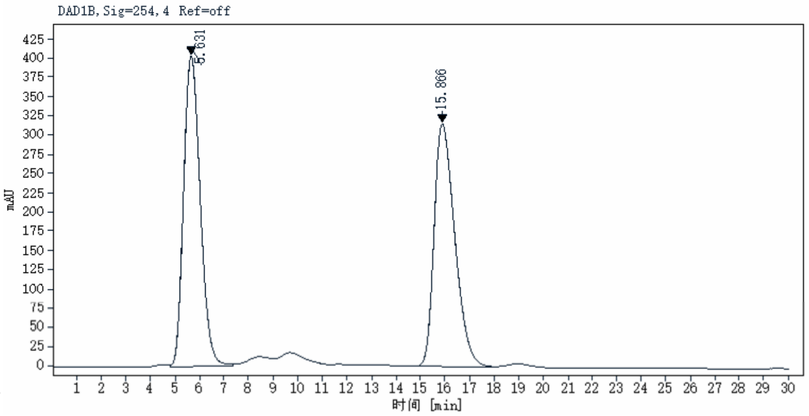


| Entry | Retention Time | Height | Area | Area% |
|---|---|---|---|---|
| 1 | 5.63 | 403.62 | 19021.79 | 50.77 |
| 2 | 15.87 | 315.37 | 18445.48 | 49.23 |

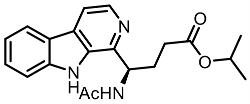


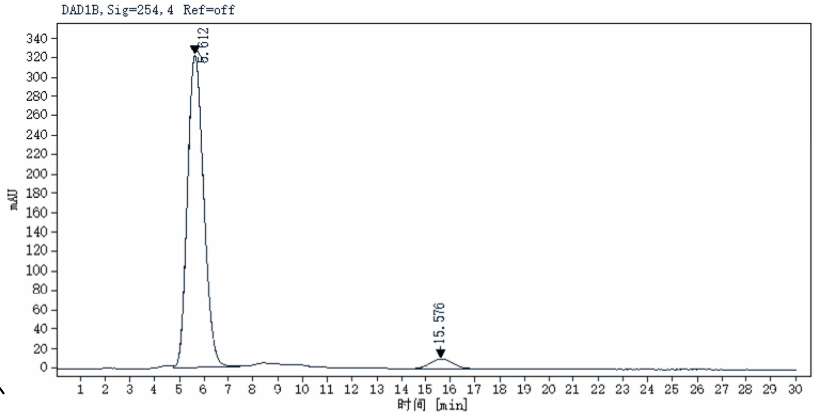


| Entry | Retention Time | Height | Area | Area% |
|---|---|---|---|---|
| 1 | 5.61 | 322.03 | 14777.32 | 95.98 |
| 2 | 15.58 | 9.91 | 618.70 | 4.02 |

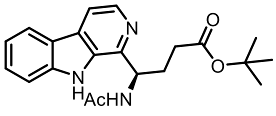

4d

($R$)-tert-butyl-4-acetamido-4-(9$H$-pyrido[3,4-$b$]indol-1-yl)butanoate (**4d**) (purification by flash column chromatography: 3% MeOH in $CH_2Cl_2$, 74% yield; 93% ee). **Mp:** 182−185 °C. **$^1$H NMR** (400 MHz, $CDCl_3$) δ 10.67 (s, 1H), 8.32 (d, $J$ = 5.3 Hz, 1H), 8.11 (d, $J$ = 7.9 Hz, 1H), 7.90 (d, $J$ = 5.3 Hz, 11H), 7.65 – 7.50 (m, 2H), 7.41 (d, $J$ = 8.3 Hz, 1H), 7.29 (d, $J$ = 8.0 Hz, 1H), 5.77 (td, $J$ = 8.4, 4.4 Hz, 1H), 2.62 – 2.51 (m, 1H), 2.44 – 2.26 (m, 2H), 2.23 – 2.13 (m, 1H), 2.10 (s, 3H), 1.47 (s, 9H). **$^{13}$C NMR** (101 MHz, $CDCl_3$) δ 173.8, 170.8, 143.3, 140.9, 137.0, 133.7, 129.5, 128.6, 121.6, 121.5, 120.0, 114.3, 112.2, 81.1, 49.4, 31.9, 30.2, 28.1, 23.5. **FTIR** ($\nu_{max}$, $cm^{-1}$): 3654, 3228, 3160, 2988, 2911, 2382, 2342, 1701, 1650, 1638, 1544, 1505, 1457, 1398, 1237, 1075, 1053, 874, 738, 622. **HRMS** (ESI-TOF) m/z: calcd for $C_{21}H_{26}N_3O_3^+$ $[M + H]^+$, 368.1969; found, 368.1966. **HPLC analysis:** Chiral MX(2) (150 x 4.6 mm, 3 μm, hexane/i-PrOH = 80:20, 1.0 mL/min, 25 °C, 254 nm), $t_r$ (major) = 5.23 min, $t_r$ (minor) = 15.49 min. **$[\alpha]_D^{20}$** = -24.8 (c = 0.2, $CHCl_3$).

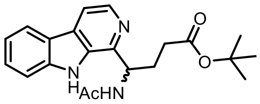

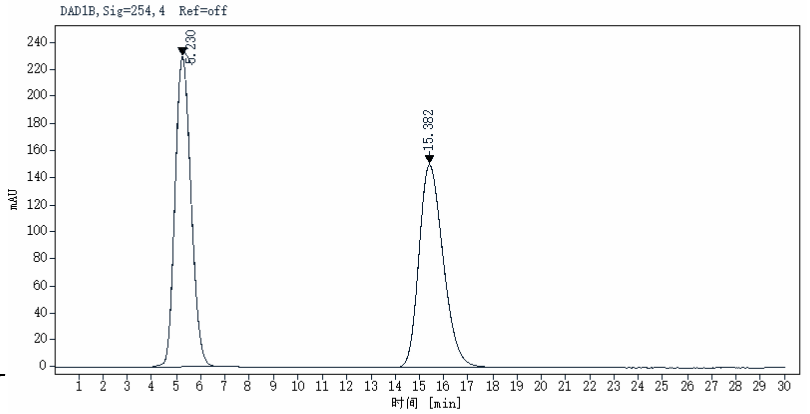


| Entry | Retention Time | Height | Area | Area% |
|---|---|---|---|---|
| 1 | 5.23 | 229.62 | 10482.00 | 50.35 |
| 2 | 15.38 | 150.24 | 10335.83 | 49.65 |

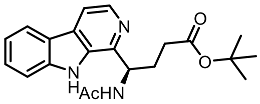

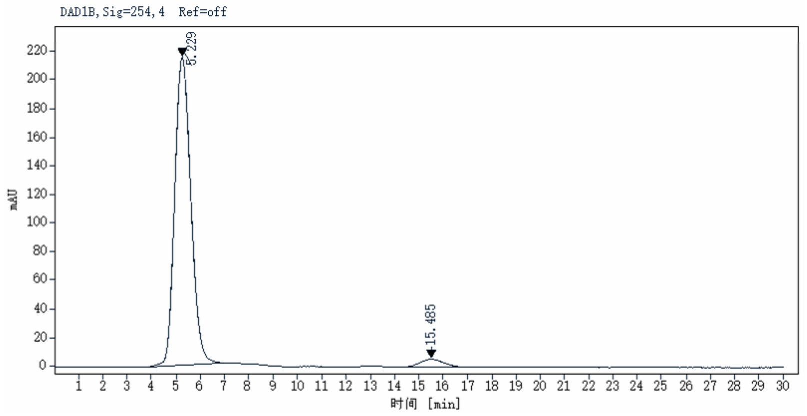


| Entry | Retention Time | Height | Area | Area% |
|---|---|---|---|---|
| 1 | 5.23 | 215.04 | 9940.05 | 96.47 |
| 2 | 15.49 | 5.49 | 363.50 | 3.53 |

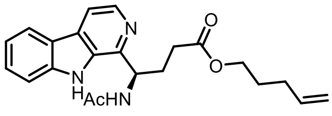

4e

(*R*)-pent-4-en-1-yl-4-acetamido-4-(9*H*-pyrido[3,4-*b*]indol-1-yl)butanoate (**4e**) (purification by flash column chromatography: 3% MeOH in $CH_2Cl_2$, 65% yield; 92% ee). **Mp:** 156−158 °C. **$^1$H NMR** (400 MHz, $CDCl_3$) δ 10.86 (s, 1H), 8.30 (d, *J* = 5.4 Hz, 1H), 8.07 (d, *J* = 7.9 Hz, 1H), 7.86 (d, *J* = 5.3 Hz, 1H), 7.74 (d, *J* = 8.2 Hz, 1H), 7.51 (d, *J* = 3.4 Hz, 2H), 7.29 – 7.20 (m, 1H), 5.89 (td, *J* = 8.0, 4.5 Hz, 1H), 5.82 – 5.69 (m, 1H), 5.04 – 4.92 (m, 2H), 4.10 – 4.01 (m, 2H), 2.64 – 2.53 (m, 1H), 2.53 – 2.38 (m, 2H), 2.33 – 2.22 (m, 1H), 2.09 (s, 3H), 2.08 – 2.01 (m, 2H), 1.71 – 1.63 (m, 2H). **$^{13}$C NMR** (101 MHz, $CDCl_3$) δ 174.0, 170.8, 143.1, 140.9, 137.4, 137.2, 133.6, 129.3, 128.4, 121.5, 121.4, 119.8, 115.2, 114.2, 112.1, 64.2, 49.6, 30.6, 29.9, 29.9, 27.6, 23.4. **FTIR** ($\nu_{max}$, $cm^{-1}$): 3223, 2964, 2848, 2357, 2163, 1994, 1650, 1544, 1459, 1237, 1120, 808, 741, 582. **HRMS** (ESI-TOF) m/z: calcd for $C_{22}H_{26}N_3O_3^+$ [M + H]$^+$, 380.1969; found, 380.1966. **HPLC analysis:** Chiral MX(2) (150 x 4.6 mm, 3 μm, hexane/i-PrOH = 80:20, 1.0 mL/min, 25 °C, 254 nm), $t_r$ (major) = 5.87 min, $t_r$ (minor) = 16.45 min. **$[\alpha]_D^{20}$** = -31.0 (c = 0.2, $CHCl_3$).

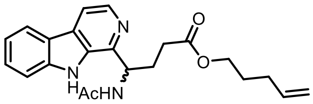

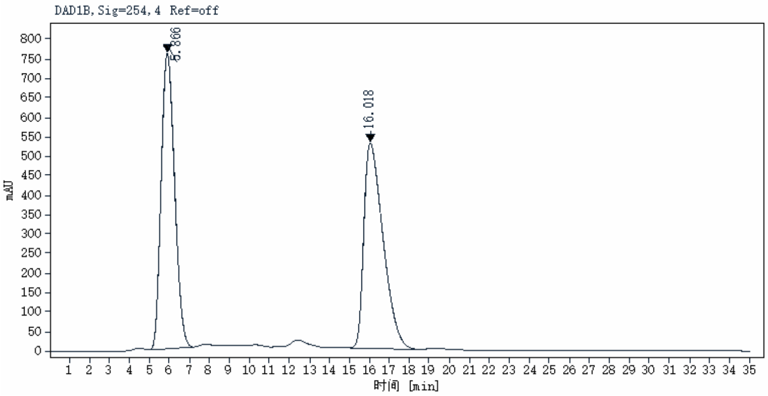


| Entry | Retention Time | Height | Area | Area% |
|---|---|---|---|---|
| 1 | 5.87 | 758.89 | 35468.59 | 50.14 |
| 2 | 16.02 | 528.01 | 35272.55 | 49.86 |

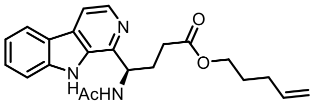

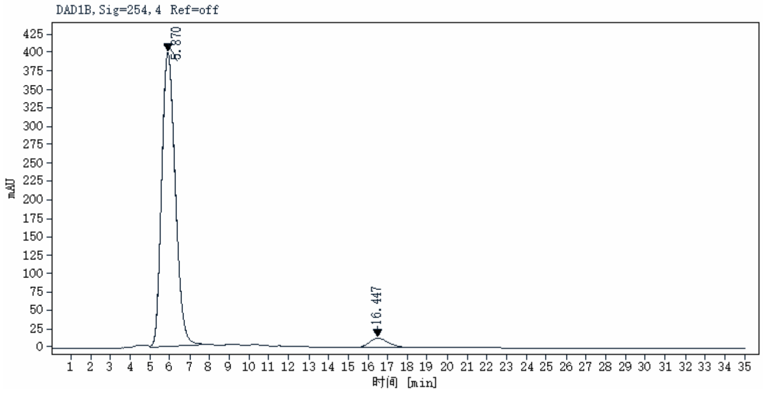


| Entry | Retention Time | Height | Area | Area% |
|---|---|---|---|---|
| 1 | 5.87 | 399.01 | 19009.93 | 96.03 |
| 2 | 16.45 | 12.26 | 784.90 | 3.97 |

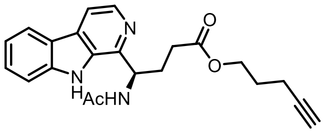

**4f**

(*R*)-pent-4-yn-1-yl-4-acetamido-4-(9*H*-pyrido[3,4-*b*]indol-1-yl)butanoate (**4f**) (purification by flash column chromatography: 3% MeOH in $CH_2Cl_2$, 67% yield; 91% ee). **Mp:** 124−126 °C**.** **$^1$H NMR** (400 MHz, DMSO) δ 11.60 (s, 1H), 8.48 (d, J = 8.1 Hz, 1H), 8.32 (d, J = 5.1 Hz, 1H), 8.23 (d, J = 7.8 Hz, 1H), 8.04 (d, J = 5.2 Hz, 1H), 7.63 (d, J = 8.1 Hz, 1H), 7.55 (t, J = 7.6 Hz, 1H), 7.25 (t, J = 7.4 Hz, 1H), 5.58 (td, J = 7.3 Hz, 1H), 3.98 (td, J = 6.5, 2.8 Hz, 2H), 2.78 (d, J = 2.6 Hz, 1H), 2.41 – 2.29 (m, 2H), 2.23 – 2.10 (m, 4H), 1.90 (s, 3H), 1.67 (p, J = 6.5 Hz, 2H). **$^{13}$C NMR** (101 MHz, $CDCl_3$) δ 174.0, 170.8, 143.0, 140.8, 137.2, 133.6, 129.3, 128.4, 121.5, 121.4, 119.8, 114.2, 112.1, 83.0, 69.0, 63.3, 49.4, 30.6, 30.0, 27.5, 23.4, 15.1. **FTIR** ($\nu_{max}$, cm$^{-1}$): 3332, 3305, 3225, 2974, 2908, 2384, 1750, 1699, 1648, 1508, 1241, 1070, 840, 731, 685, 657, 600, 554. **HRMS** (ESI-TOF) m/z: calcd for $C_{22}H_{24}N_3O_3^+$ [M + H]$^+$, 378.1812; found, 378.1809. **HPLC analysis:** Chiral MX(2) (150 x 4.6 mm, 3 μm, hexane/i-PrOH = 80:20, 1.0 mL/min, 25 °C, 254 nm), $t_r$ (major) = 6.72 min, $t_r$ (minor) = 24.22 min. **[α]$_D^{20}$** = -30.6 (c = 0.2, $CHCl_3$).

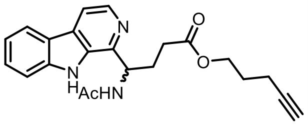

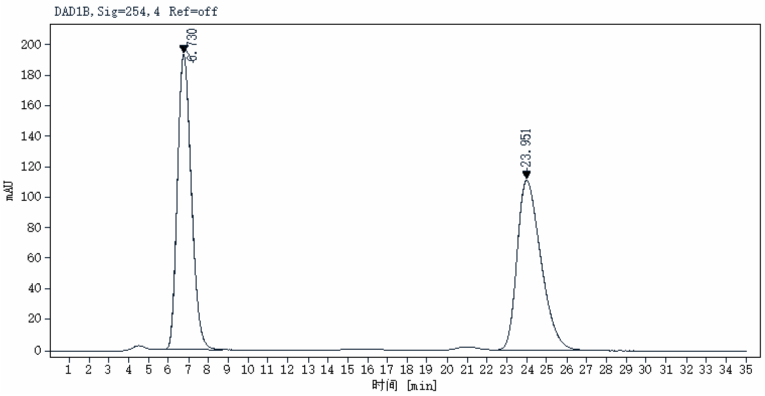


| Entry | Retention Time | Height | Area | Area% |
|---|---|---|---|---|
| 1 | 6.73 | 193.21 | 9396.32 | 50.50 |
| 2 | 23.95 | 111.13 | 9208.43 | 49.50 |

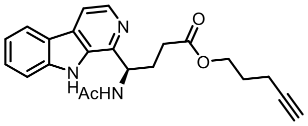

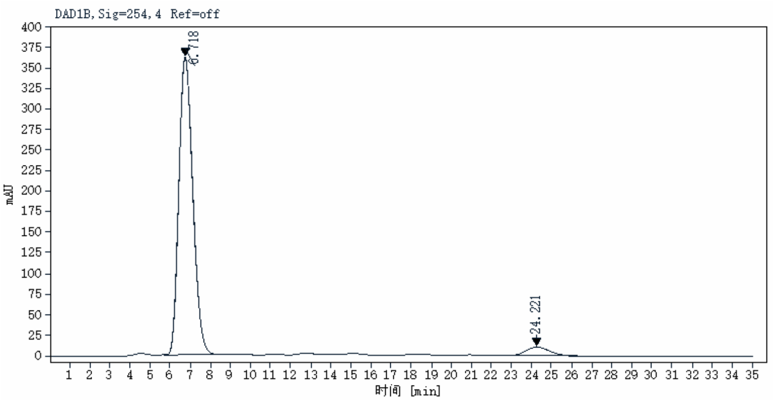


| Entry | Retention Time | Height | Area | Area% |
|---|---|---|---|---|
| 1 | 6.72 | 362.30 | 17498.96 | 95.52 |
| 2 | 24.22 | 10.26 | 821.01 | 4.48 |

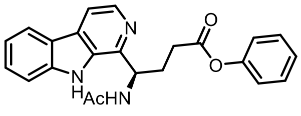

**4g**

($R$)-phenyl-4-acetamido-4-(9$H$-pyrido[3,4-$b$]indol-1-yl)butanoate (**4g**) (purification by flash column chromatography: 25% acetone in hexanes, 71% yield; 88% ee). **Mp:** 155−161 °C. **$^1$H NMR** (400 MHz, DMSO) δ 11.65 (s, 1H), 8.55 (d, $J$ = 8.2 Hz, 1H), 8.35 (d, $J$ = 5.3 Hz, 1H), 8.23 (d, $J$ = 7.9 Hz, 1H), 8.06 (d, $J$ = 5.1 Hz, 1H), 7.65 (d, $J$ = 8.2 Hz, 1H), 7.56 (t, $J$ = 7.5 Hz, 1H), 7.40 – 7.32 (m, 2H), 7.28 – 7.19 (m, 2H), 7.06 (d, $J$ = 8.1 Hz, 2H), 5.68 (td, $J$ = 7.2 Hz, 1H), 2.74 – 2.56 (m, 2H), 2.36 – 2.21 (m, 2H), 1.93 (s, 3H). **$^{13}$C NMR** (101 MHz, DMSO) δ 171.8, 169.8, 150.9, 144.7, 141.0, 137.8, 133.4, 129.8, 128.6, 128.6, 126.1, 122.2, 122.1, 121.3, 119.9, 114.3, 112.5, 49.7, 30.9, 29.7, 23.1. **FTIR** ($\nu_{max}$, cm$^{-1}$): 3215, 3178, 3025, 2991, 2825, 2361, 2341, 1649, 15556, 15041, 1429, 1375, 1320, 1241, 1065, 741, 705, 656, 602, 566. **HRMS** (ESI-TOF) m/z: calcd for $C_{23}H_{22}N_3O_3^+$ [M + H]$^+$, 388.1656; found, 388.1650. **HPLC analysis:** Chiral MX(2) (150 x 4.6 mm, 3 μm, hexane/i-PrOH = 90:10, 1.0 mL/min, 25 °C, 254 nm), $t_r$ (major) = 5.80 min, $t_r$ (minor) = 13.80 min. **[α]$_D^{20}$** = -27.1 (c = 0.2, $CHCl_3$).

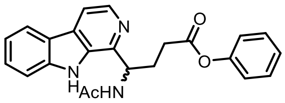

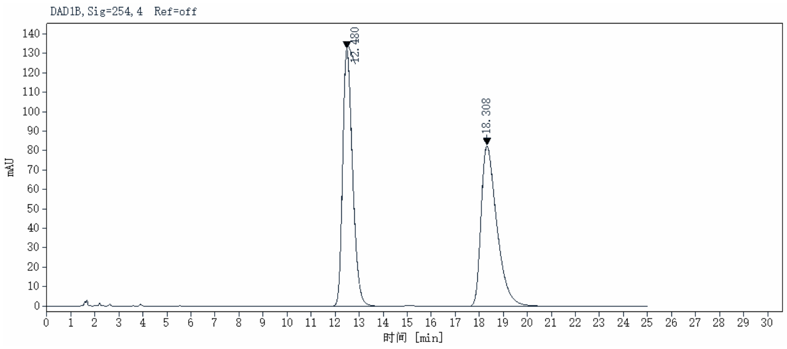


| Entry | Retention Time | Height | Area | Area% |
|---|---|---|---|---|
| 1 | 12.31 | 133.71 | 3941.31 | 50.20 |
| 2 | 18.40 | 80.92 | 3909.22 | 49.80 |

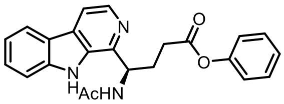

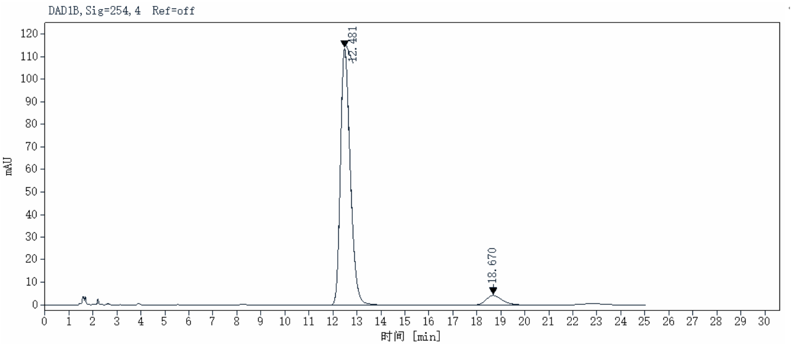


| Entry | Retention Time | Height | Area | Area% |
|---|---|---|---|---|
| 1 | 12.47 | 114.45 | 3213.41 | 93.91 |
| 2 | 18.59 | 6.31 | 208.39 | 6.09 |

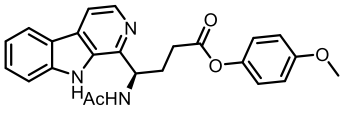

4h

($R$)-4-methoxyphenyl-4-acetamido-4-(9$H$-pyrido[3,4-$b$]indol-1-yl) butanoate (**4h**) (purification by flash column chromatography: 25% acetone in hexanes, 74% yield; 90% ee). **Mp**: 188−190 °C. **$^1$H NMR** (400 MHz, $CDCl_3$) δ 10.18 (br s, 1H), 8.35 (d, $J$ = 5.4 Hz, 1H), 8.11 (d, $J$ = 7.9 Hz, 1H), 7.93 (d, $J$ = 5.4 Hz, 1H), 7.61 – 7.52 (m, 2H), 7.46 (d, $J$ = 8.2 Hz, 1H), 7.32 – 7.27 (m, 1H), 7.09 – 7.02 (m, 2H), 6.93 – 6.85 (m, 2H), 5.87 (td, $J$ = 8.6, 4.5 Hz, 1H), 3.80 (s, 3H), 2.98 – 2.87 (m, 1H), 2.72 – 2.63 (m, 1H), 2.59 – 2.49 (m, 1H), 2.33 – 2.24 (m, 1H), 2.13 (s, 3H). **$^{13}$C NMR** (101 MHz, $CDCl_3$) δ 173.6, 171.1, 157.5, 144.3, 142.9, 141.0, 137.1, 133.6, 129.9, 128.9, 122.6, 121.8, 121.6, 120.3, 114.6, 114.5, 112.5, 55.7, 49.1, 31.0, 30.4, 23.6. **FTIR** ($\nu_{max}$, $cm^{-1}$): 3216, 3175, 2985, 2920, 2901, 2359, 2341, 1748, 1701, 1650, 1637, 1558, 1505, 1462, 1432, 1378, 1319, 1183, 1083, 880, 814, 775, 738, 668, 629, 557. **HRMS** (ESI-TOF) m/z: calcd for $C_{24}H_{24}N_3O_4^+$ $[M + H]^+$, 418.1761; found, 418.1764. **HPLC analysis:** Chiral MX(2) (150 x 4.6 mm, 3 µm, hexane/i-PrOH = 80:20, 1.0 mL/min, 25 ºC, 254 nm), $t_r$ (major) = 3.44 min, $t_r$(minor) = 16.99 min. **$[\alpha]_D^{20}$** = -28.6 (c = 0.2, $CHCl_3$).

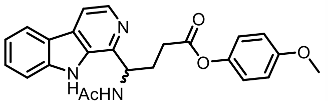

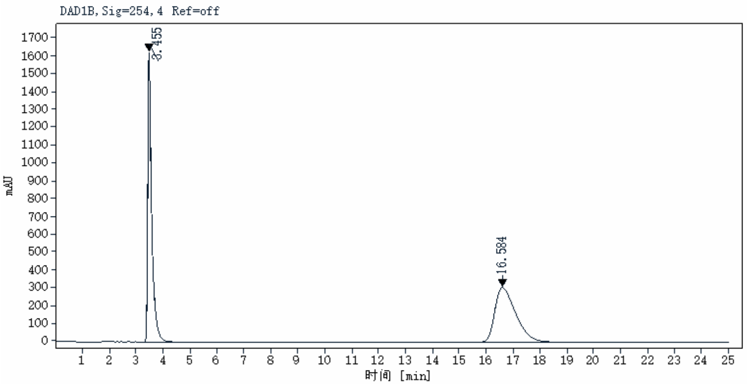


| Entry | Retention Time | Height | Area | Area% |
|---|---|---|---|---|
| 1 | 3.46 | 1623.05 | 16493.27 | 49.87 |
| 2 | 16.58 | 306.52 | 16578.22 | 50.13 |

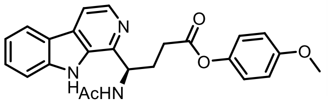

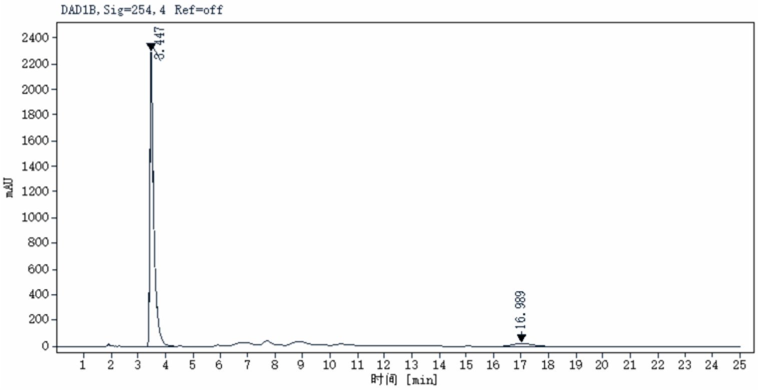


| Entry | Retention Time | Height | Area | Area% |
|---|---|---|---|---|
| 1 | 3.44 | 2297.37 | 22890.45 | 94.79 |
| 2 | 16.99 | 24.14 | 1257.42 | 5.21 |

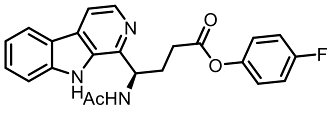

**4i**

(*R*)-4-fluorophenyl-4-acetamido-4-(9*H*-pyrido[3,4-*b*]indol-1-yl)butan-oate (**4i**) (purification by flash column chromatography: 25% acetone in hexanes, 76% yield; 83% ee). **Mp**: 167−170 °C. **$^{1}$H NMR** (400 MHz, $CDCl_3$) δ 10.34 (br s, 1H), 8.35 (d, *J* = 5.4 Hz, 1H), 8.12 (d, *J* = 7.9 Hz, 1H), 7.96 (d, *J* = 5.4 Hz, 1H), 7.70 (d, *J* = 5.3 Hz, 1H), 7.57 (d, *J* = 4.0 Hz, 2H), 7.35 – 7.27 (m, 1H), 7.15 – 6.98 (m, 4H), 5.91 (td, *J* = 8.7, 4.4 Hz, 1H), 2.97 – 2.85 (m, 1H), 2.75 – 2.65 (m, 1H), 2.62 – 2.49 (m, 1H), 2.38 – 2.26 (m, 1H), 2.15 (s, 3H). **$^{13}$C NMR** (101 MHz, $CDCl_3$) δ 173.2, 171.3, 163.2 (d, *J* = 294.0 Hz), 146.6, 142.5, 141.3, 136.3, 133.5, 130.4, 129.3, 123.2 (d, *J* = 8.5 Hz), 121.9, 121.4, 120.6, 116.3 (d, *J* = 23.4 Hz), 114.7, 112.5, 49.0, 30.9, 30.2, 23.6. **$^{19}$F NMR** (376 MHz, $CDCl_3$) δ -116.79 (septet). **FTIR** ($\nu_{max}$, $cm^{-1}$): 3218, 2974, 2911, 2366, 2335, 1754, 1701, 1650, 1556, 1505, 1457, 1396, 1237, 1072, 818, 738, 670, 533. **HRMS** (ESI-TOF) m/z: calcd for $C_{23}H_{21}FN_3O_3^+$ [M + H]$^+$, 406.1561; found, 406.1557. **HPLC analysis:** Chiral MX(2) (150 x 4.6 mm, 3 µm, hexane/i-PrOH = 80:20, 1.0 mL/min, 25 °C, 254 nm), $t_r$ (major) = 3.49 min, $t_r$ (minor) = 16.26 min. **$[\alpha]_D^{20}$** = -17.2 (c = 0.2, $CHCl_3$).

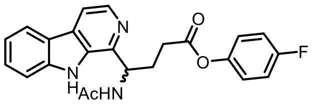

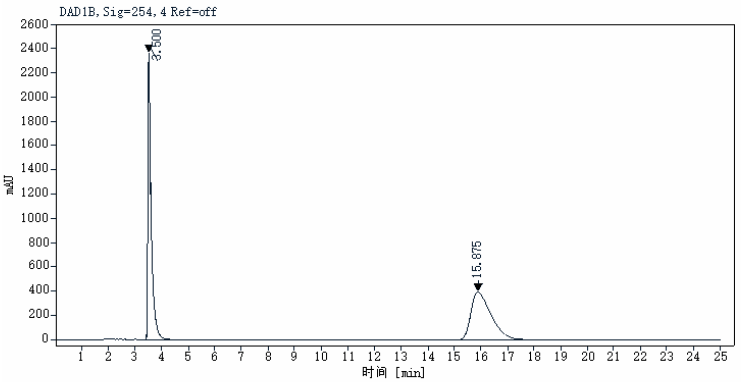


| Entry | Retention Time | Height | Area | Area% |
|---|---|---|---|---|
| 1 | 3.50 | 2366.89 | 20582.27 | 49.68 |
| 2 | 15.87 | 394.66 | 20850.03 | 50.32 |

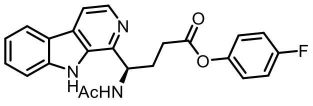

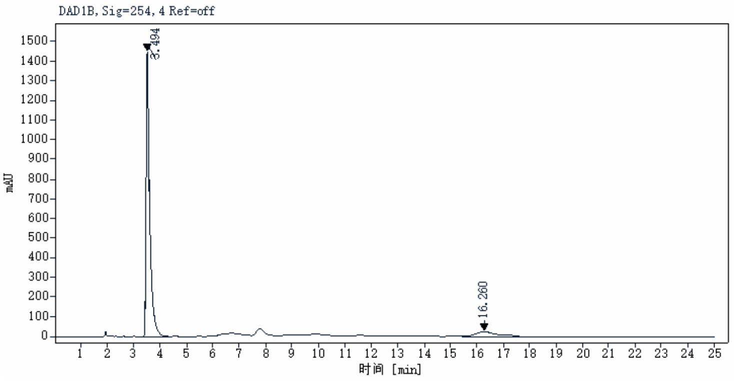


| Entry | Retention Time | Height | Area | Area% |
|---|---|---|---|---|
| 1 | 3.49 | 1448.63 | 13761.05 | 91.68 |
| 2 | 16.26 | 23.46 | 1248.82 | 8.32 |

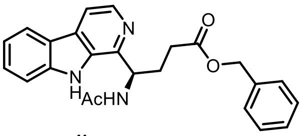

**4j**

(*R*)-benzyl-4-acetamido-4-(9*H*-pyrido[3,4-*b*]indol-1-yl)butanoate(**4j**) (purification by flash column chromatography: 3% MeOH in $CH_2Cl_2$, 80% yield; 90% ee). **Mp:** 182−185 °C. **$^1$H NMR** (400 MHz, $CDCl_3$) δ 10.62 (s, 1H), 8.32 (d, *J* = 5.4 Hz, 1H), 8.11 (d, *J* = 7.9 Hz, 1H), 7.91 (d, *J* = 5.4 Hz, 1H), 7.63 – 7.54 (m, 2H), 7.50 (d, *J* = 5.3 Hz, 1H), 7.40 – 7.26 (m, 6H), 5.85 (td, *J* = 8.4, 4.4 Hz, 1H), 5.17 (d, *J* = 12.3 Hz, 1H), 5.07 (d, *J* = 12.3 Hz, 1H), 2.74 – 2.62 (m, 1H), 2.55 – 2.42 (m, 2H), 2.34 – 2.22 (m, 1H), 2.09 (s, 3H). **$^{13}$C NMR** (101 MHz, $CDCl_3$) δ 173.9, 171.0, 142.8, 141.0, 136.6, 135.7, 133.6, 129.7, 128.8, 128.5, 128.2, 128.2, 121.6, 121.3, 120.1, 114.4, 112.2, 66.6, 49.3, 30.7, 29.7, 23.4. **FTIR** ($\nu_{max}$, cm$^{-1}$): 3216, 3165, 2965, 2905, 2887, 2380, 2325, 2308, 2138, 1922, 1845, 1653, 1558, 15441, 1507, 1473, 1458, 1372, 1323, 1235, 1074, 737, 624. **HRMS** (ESI-TOF) m/z: calcd for $C_{24}H_{24}N_3O_3^+$ [M + H]$^+$, 402.1812; found, 402.1808. **HPLC analysis:** Chiral MX(2) (150 x 4.6 mm, 3 µm, hexane/i-PrOH = 80:20, 1.0 mL/min, 25 °C, 254 nm), $t_r$ (major) = 7.68 min, $t_r$ (minor) = 26.87 min. **$[\alpha]_D^{20}$** = +4.0 (c = 0.2, $CHCl_3$).

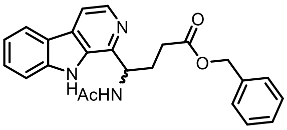

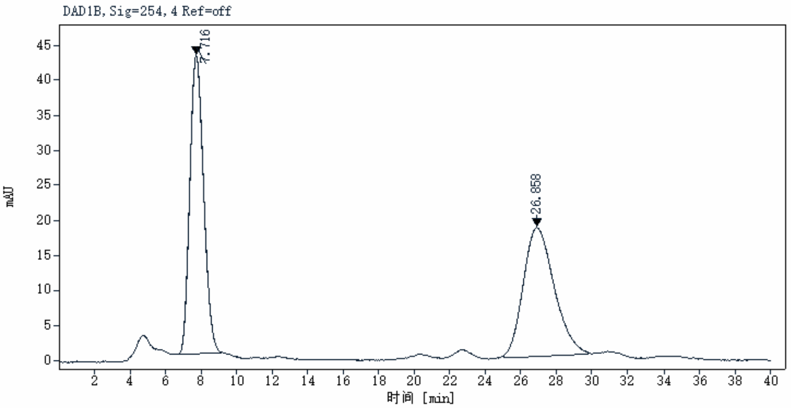


| Entry | Retention Time | Height | Area | Area% |
|---|---|---|---|---|
| 1 | 7.72 | 42.54 | 2290.50 | 50.72 |
| 2 | 26.86 | 18.39 | 2225.53 | 49.28 |

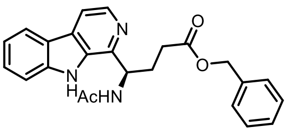

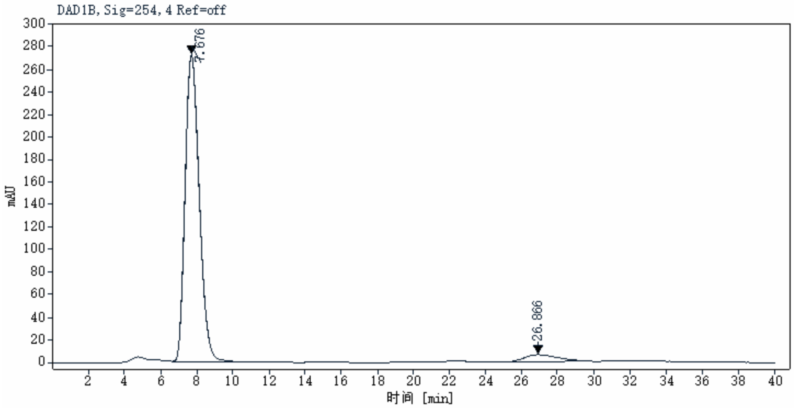


| Entry | Retention Time | Height | Area | Area% |
|---|---|---|---|---|
| 1 | 7.68 | 272.41 | 14785.68 | 95.10 |
| 2 | 26.87 | 6.04 | 762.26 | 4.90 |

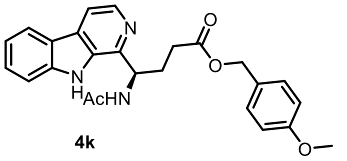

**4k**

(*R*)-4-methoxybenzyl-4-acetamido-4-(9*H*-pyrido[3,4-*b*]indol-1-yl) butanoate (**4k**) (purification by flash column chromatography: 3% MeOH in $CH_2Cl_2$, 73% yield; 91% ee). **Mp**: 191−194 °C. **$^1$H NMR** (400 MHz, $CDCl_3$) δ 10.57 (s, 1H), 8.31 (d, *J* = 5.3 Hz, 1H), 8.09 (d, *J* = 7.9 Hz, 1H), 7.88 (d, *J* = 5.3 Hz, 1H), 7.54 (d, *J* = 3.6 Hz, 2H), 7.39 (d, *J* = 8.2 Hz, 1H), 7.32 – 7.27 (m, 2H), 6.85 (d, *J* = 8.3 Hz, 3H), 5.85 (td, *J* = 8.2, 4.6 Hz, 1H), 5.12 (d, *J* = 11.9 Hz, 1H), 5.02 (d, *J* = 11.8 Hz, 1H), 3.78 (s, 3H), 2.74 – 2.61 (m, 1H), 2.54 – 2.41 (m, 2H), 2.32 – 2.21 (m, 1H), 2.08 (s, 3H). **$^{13}$C NMR** (101 MHz, $CDCl_3$) δ 174.0, 170.9, 159.6, 142.9, 140.9, 133.5, 130.2, 129.5, 128.7, 127.8, 121.6, 121.3, 120.0, 114.3, 113.9, 112.2, 66.5, 55.3, 49.4, 30.7, 29.8, 23.5. **FTIR** ($\nu_{max}$, $cm^{-1}$): 3218, 3179, 2981, 2904, 2381, 2340, 1859, 1771, 1749, 1698, 1653, 1647, 1576, 1541, 1521, 1507, 1457, 1398, 1320, 1235, 883, 748, 705, 670, 518. **HRMS** (ESI-TOF) m/z: calcd for $C_{25}H_{26}N_3O_4^+$ $[M + H]^+$, 432.1918; found, 432.1914. **HPLC analysis:** Chiral MX(2) (150 x 4.6 mm, 3 μm, hexane/i-PrOH = 80:20, 1.0 mL/min, 25 °C, 254 nm), $t_r$ (major) = 7.66 min, $t_r$ (minor) = 26.62 min. **$[\alpha]_D^{20}$** = +19.0 (c = 0.2, $CHCl_3$).

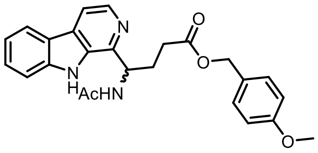

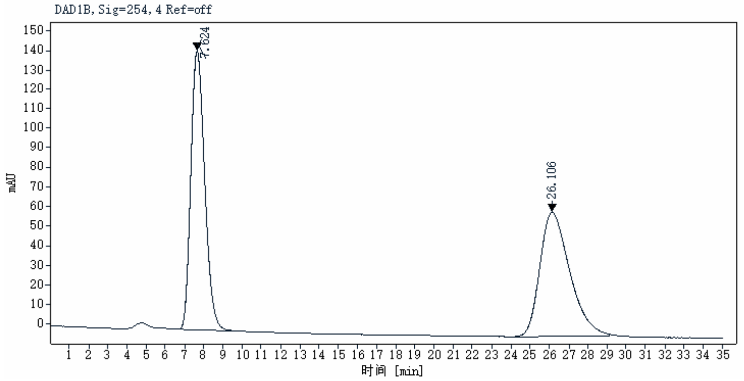


| Entry | Retention Time | Height | Area | Area% |
|---|---|---|---|---|
| 1 | 7.62 | 142.09 | 7105.02 | 50.89 |
| 2 | 26.10 | 63.26 | 6857.78 | 49.11 |

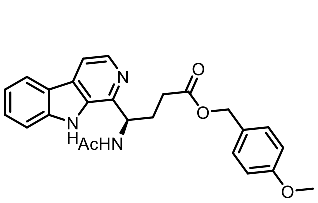

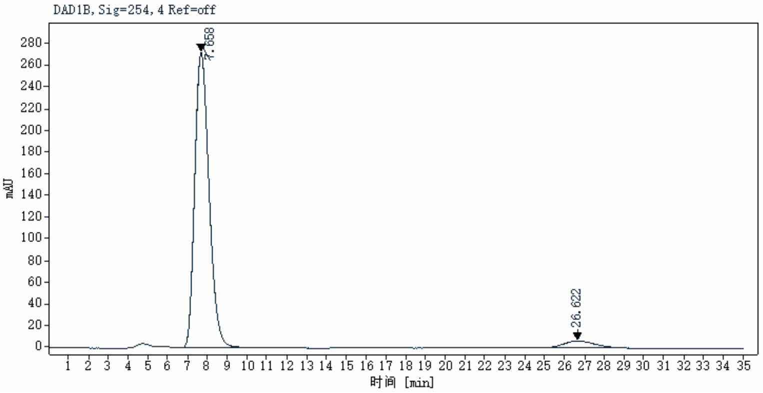


| Entry | Retention Time | Height | Area | Area% |
|---|---|---|---|---|
| 1 | 7.66 | 271.77 | 13529.25 | 95.38 |
| 2 | 26.62 | 6.19 | 654.90 | 4.62 |

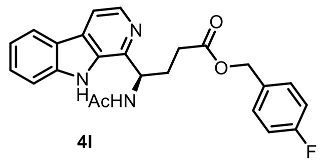

4l

(*R*)-4-fluorobenzyl-4-acetamido-4-(9*H*-pyrido[3,4-*b*]indol-1-yl)-butaoate (**4l**) (purification by flash column chromatography: 3% MeOH in $CH_2Cl_2$, 75% yield; 86% ee). **Mp**: 188−190 °C. **$^1$H NMR** (400 MHz, $CDCl_3$) δ 10.78 (s, 1H), 8.30 (d, *J* = 5.3 Hz, 1H), 8.08 (d, *J* = 7.9 Hz, 1H), 7.87 (d, *J* = 5.3 Hz, 1H), 7.63 (d, *J* = 8.2 Hz, 1H), 7.56 – 7.49 (m, 2H), 7.29 – 7.23 (m, 3H), 6.98 (t, *J* = 8.7 Hz, 3H), 5.90 (td, *J* = 8.0, 4.8 Hz, 1H), 5.07 (d, *J* = 12.3 Hz, 1H), 4.99 (d, *J* = 12.3 Hz, 1H), 2.69 – 2.56 (m, 1H), 2.56 – 2.41 (m, 2H), 2.36 – 2.25 (m, 1H), 2.09 (s, 3H). **$^{13}$C NMR** (101 MHz, CDCl3) δ 173.7, 170.9, 162.6 (d, J = 246.9 Hz), 142.8, 140.9, 137.1, 133.6, 131.5, 130.3 (d, J = 8.2 Hz), 129.4, 128.6, 121.6, 121.4, 119.9, 115.5 (d, J = 21.5 Hz), 114.3, 112.1, 65.9, 49.5, 30.6, 29.9, 23.5. **$^{19}$F NMR** (376 MHz, $CDCl_3$) δ -113.55 (septet). **FTIR** ($\nu_{max}$, $cm^{-1}$): 3218, 3172, 2988, 2923, 2357, 1655, 1563, 1499, 1319, 1241, 1094, 1036, 1019, 738, 576. **HRMS** (ESI-TOF) m/z: calcd for $C_{24}H_{23}FN_3O_3^+$ $[M + H]^+$, 420.1718; found, 420.1714. **HPLC analysis:** Chiral MX(2) (150 x 4.6 mm, 3 μm, hexane/i-PrOH = 70:30, 1.0 mL/min, 25 ºC, 254 nm), $t_r$ (major) = 4.44 min, $t_r$ (minor) = 16.43 min. **[α]$_D^{20}$** = +3.0 (c = 0.2, $CHCl_3$).

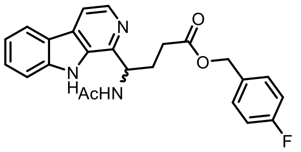

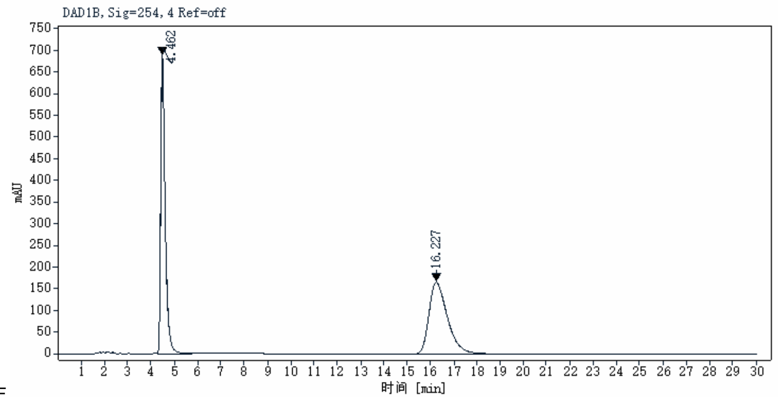


| Entry | Retention Time | Height | Area | Area% |
|---|---|---|---|---|
| 1 | 4.46 | 689.37 | 9208.64 | 50.23 |
| 2 | 16.23 | 165.74 | 9123.27 | 49.77 |

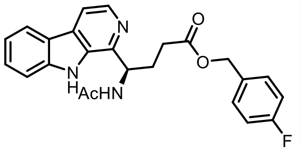

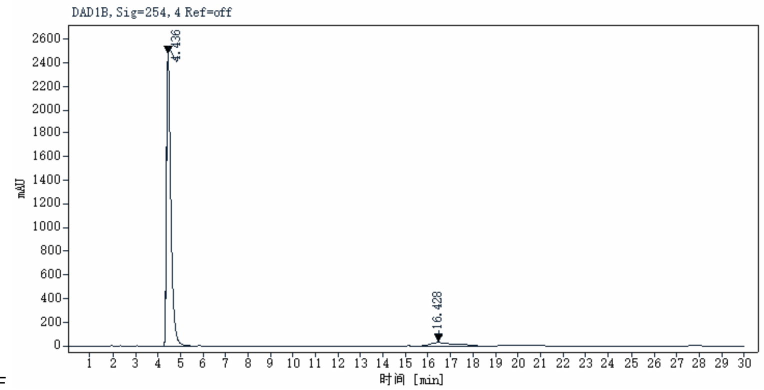


| Entry | Retention Time | Height | Area | Area% |
|---|---|---|---|---|
| 1 | 4.44 | 2470.81 | 33109.13 | 92.99 |
| 2 | 16.43 | 30.28 | 2494.60 | 7.01 |

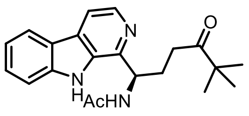

**4m**

(*R*)-*N*-(5,5-dimethyl-4-oxo-1-(9*H*-pyrido[3,4-*b*]indol-1-yl)hexyl)acetamide (**4m**) (purification by flash column chromatography: 3% MeOH in $CH_2Cl_2$, 78% yield; 88% ee). **Mp**: 234−236 °C. **$^1$H NMR** (400 MHz, $CDCl_3$) δ 10.82 (s, 1H), 8.30 (d, *J* = 5.3 Hz, 1H), 8.11 (d, *J* = 7.9 Hz, 1H), 7.89 (d, *J* = 5.3 Hz, 1H), 7.66 (d, *J* = 8.5 Hz, 1H), 7.63 – 7.52 (m, 2H), 7.29 (d, *J* = 7.1 Hz, 1H), 5.62 (td, *J* = 8.1, 4.8 Hz, 1H), 3.04 (ddd, *J* = 18.9, 9.8, 4.6 Hz, 1H), 2.59 (dt, *J* = 18.6, 5.1 Hz, 1H), 2.47 – 2.36 (m, 1H), 2.09 (s, 3H), 2.04 – 1.94 (m, 1H), 1.18 (s, 9H). **$^{13}$C NMR** (101 MHz, $CDCl_3$) δ 217.9, 170.8, 143.5, 140.8, 136.8, 133.5, 129.3, 128.5, 121.5, 121.4, 119.9, 114.1, 112.3, 49.3, 44.2, 33.3, 29.8, 26.7, 23.4. **FTIR** ($\nu_{max}$, cm$^{-1}$): 3216, 3177, 2983, 2928, 2382, 2335, 1760, 1647, 1558, 1542, 1500, 1457, 1431, 1374, 1228, 1071, 914, 822, 738, 668, 623, 570. **HRMS** (ESI-TOF) m/z: calcd for $C_{21}H_{26}N_3O_2^+$ [M + H]$^+$, 352.2020; found, 352.2016. **HPLC analysis:** Chiral MX(2) (150 x 4.6 mm, 3 µm, hexane/i-PrOH = 80:20, 1.0 mL/min, 25 ºC, 254 nm), $t_r$ (major) = 9.81 min, $t_r$ (minor) = 18.42 min. **$[\alpha]_D^{20}$** =-31.3 (c = 0.2, $CHCl_3$).

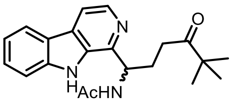

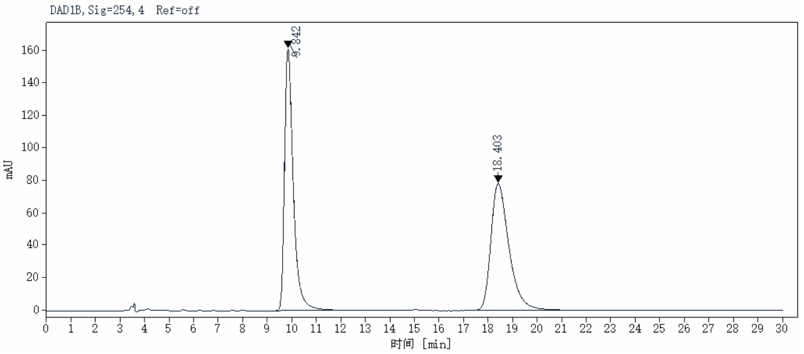


| Entry | Retention Time | Height | Area | Area% |
|---|---|---|---|---|
| 1 | 9.91 | 161.03 | 3787.33 | 49.90 |
| 2 | 18.53 | 79.36 | 3803.14 | 50.10 |

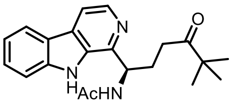

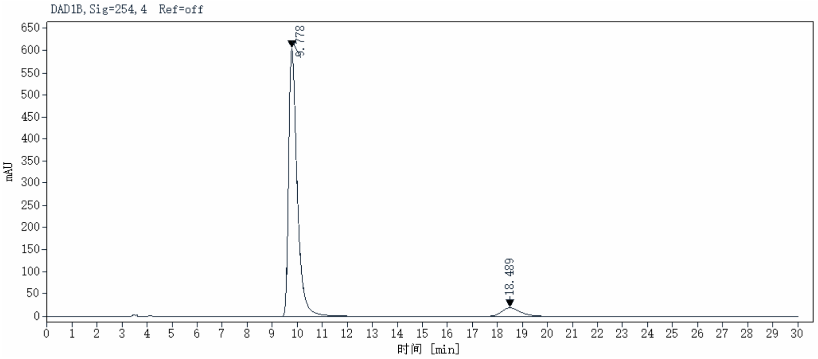


| Entry | Retention Time | Height | Area | Area% |
|---|---|---|---|---|
| 1 | 9.81 | 610.37 | 14337.01 | 93.91 |
| 2 | 18.42 | 20.12 | 929.75 | 6.19 |

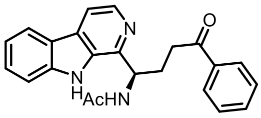

4n

($R$)-$N$-(4-oxo-4-phenyl-1-(9$H$-pyrido[3,4-$b$]indol-1-yl)butyl)acetamide (**4n**) (purification by flash column chromatography: 3% MeOH in $CH_2Cl_2$, 75% yield; 86% ee). **Mp**: 196−200 °C. **$^1$H NMR** (400 MHz, $CDCl_3$) δ 11.04 (s, 1H), 8.32 (d, $J$ = 5.4 Hz, 1H), 8.12 (d, $J$ = 7.9 Hz, 1H), 7.95 (t, $J$ = 6.3 Hz, 3H), 7.81 (br s, 1H), 7.69 (d, $J$ = 8.2 Hz, 1H), 7.59 (t, $J$ = 7.7 Hz, 1H), 7.53 (t, $J$ = 7.3 Hz, 1H), 7.41 (t, $J$ = 7.7 Hz, 2H), 7.31 (t, $J$ = 7.5 Hz, 1H), 5.83 (td, $J$ = 8.8, 3.7 Hz, 1H), 3.48 (ddd, $J$ = 18.2, 9.3, 5.1 Hz, 1H), 3.08 (dt, $J$ = 18.3, 5.4 Hz, 1H), 2.70 – 2.58 (m, 1H), 2.36 – 2.24 (m, 1H), 2.10 (s, 3H). **$^{13}$C NMR** (101 MHz, $CDCl_3$) δ 201.1, 171.1, 143.1, 141.2, 136.7, 135.8, 133.4, 133.3, 130.0, 129.0, 128.6, 128.2, 121.7, 121.3, 120.2, 114.4, 112.4, 49.6, 35.1, 29.6, 23.4. **HRMS** (ESI-TOF) m/z: calcd for $C_{23}H_{22}N_3O_2^+$ [M + H]$^+$, 372.1707; found, 372.1702. **FTIR** ($\nu_{max}$, cm$^{-1}$): 3218, 3179, 2975, 2925, 2364, 2340, 1772, 1748, 1648, 1455, 1419, 1398, 1375, 1318, 1240, 1055, 744, 690, 669, 570; **HPLC analysis:** Chiral MX(2) (150 x 4.6 mm, 3 μm, hexane/i-PrOH = 80:20, 1.0 mL/min, 25 ºC, 254 nm), $t_r$ (major) = 3.86 min, $t_r$ (minor) = 14.12 min. **[α]$_D$$^{20}$** =-29.1 (c = 0.2, $CHCl_3$).

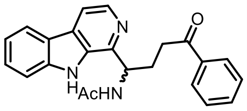

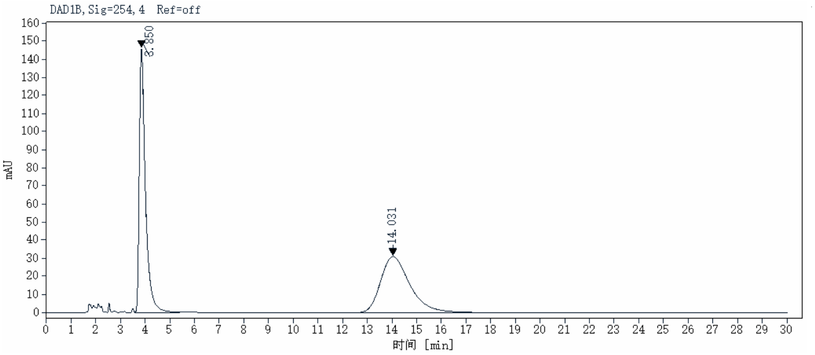


| Entry | Retention Time | Height | Area | Area% |
|---|---|---|---|---|
| 1 | 3.91 | 148.37 | 2521.33 | 49.74 |
| 2 | 14.01 | 32.26 | 2547.61 | 50.26 |

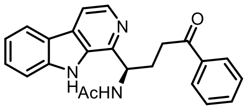

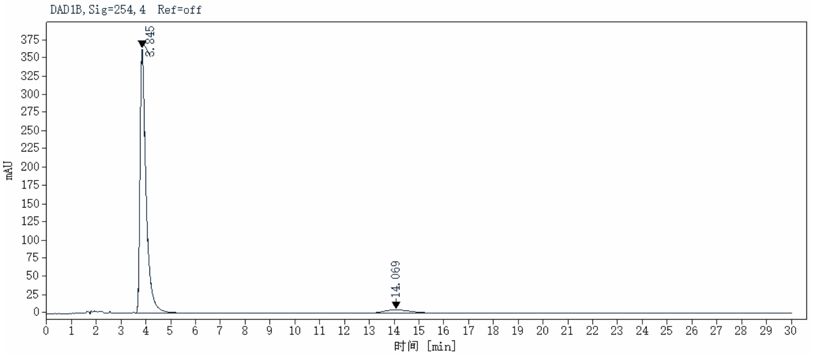


| Entry | Retention Time | Height | Area | Area% |
|---|---|---|---|---|
| 1 | 3.86 | 363.71 | 6133.47 | 93.11 |
| 2 | 14.12 | 4.14 | 453.87 | 6.89 |

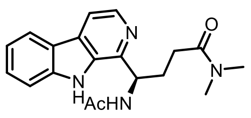

**4o**

($R$)-4-acetamido-$N,N$-dimethyl-4-(9$H$-pyrido[3,4-$b$]indol-1-yl)butanamide (**4o)** (purification by flash column chromatography: 4% MeOH in $CH_2Cl_2$, 73% yield; 86% ee). **Mp**: 198−200 °C. **$^1$H NMR** (400 MHz, $CDCl_3$) δ 11.60 (s, 1H), 8.29 (d, $J$ = 5.4 Hz, 1H), 8.12 (d, $J$ = 7.9 Hz, 1H), 7.91 (d, $J$ = 5.3 Hz, 1H), 7.82 (d, $J$ = 7.7 Hz, 1H), 7.70 (d, $J$ = 8.3 Hz, 1H), 7.62 – 7.51 (m, 1H), 7.33 – 7.27 (m, 1H), 5.72 – 5.63 (m, 1H), 3.14 (s, 3H), 3.08 (s, 3H), 2.99 (ddd, $J$ = 16.5, 11.2, 3.2 Hz, 1H), 2.51 (ddt, $J$ = 14.2, 11.2, 3.1 Hz, 1H), 2.36 (ddd, $J$ = 16.5, 5.7, 3.4 Hz, 1H), 2.13 (s, 3H), 1.98 – 1.88 (m, 1H). **$^{13}$C NMR** (101 MHz, $CDCl_3$) δ 173.5, 170.8, 143.6, 141.0, 136.4, 133.5, 129.0, 128.3, 121.5, 121.3, 119.6, 114.0, 112.5, 49.8, 37.3, 35.8, 32.4, 29.8, 23.5. **FTIR** ($\nu_{max}$, $cm^{-1}$): 3230, 3177, 2979, 2942, 2383, 2338, 2226, 1748, 1715, 1698, 1658, 1648, 1558, 1537, 1455, 1430, 1377, 1318, 1238, 1059, 884, 818, 738, 625, 554. **HRMS** (ESI-TOF) m/z: calcd for $C_{19}H_{23}N_4O_2^+$ [M + H]$^+$, 339.1866; found, 339.1861. **HPLC analysis:** Chiral NX(2) (150 x 4.6 mm, 3 μm, hexane/i-PrOH = 80:20, 1.0 mL/min, 25 °C, 254 nm), $t_r$ (major) = 2.99 min, $t_r$ (minor) = 7.65 min. **$[\alpha]_D^{20}$** =-26.5 (c = 0.2, $CHCl_3$).

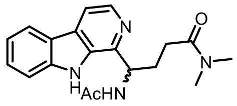

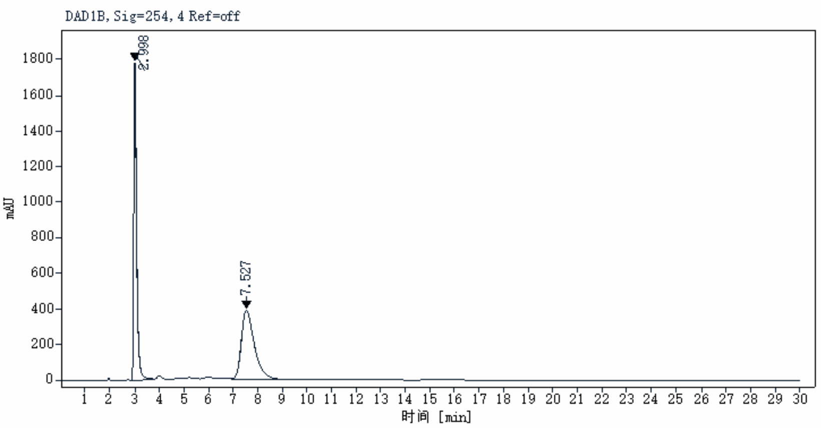


| Entry | Retention Time | Height | Area | Area% |
|---|---|---|---|---|
| 1 | 3.00 | 1787.15 | 14328.00 | 50.11 |
| 2 | 7.53 | 388.81 | 14262.93 | 49.89 |

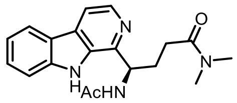

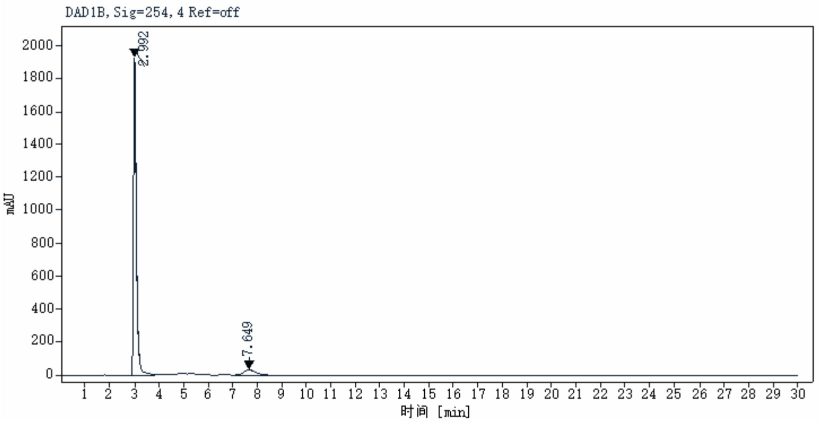


| Entry | Retention Time | Height | Area | Area% |
|---|---|---|---|---|
| 1 | 2.99 | 1926.61 | 15485.20 | 92.91 |
| 2 | 7.65 | 31.39 | 1182.11 | 7.09 |

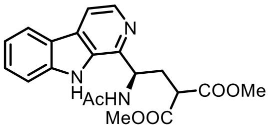


**4p**

($R$)-dimethyl-2-(2-acetamido-2-(9$H$-pyrido[3,4-$b$]indol-1-yl)ethyl)malonate (**4p**) (purification by flash column chromatography: 3% MeOH in $CH_2Cl_2$, 77% yield; 86% ee). **Mp**: 180−182 °C. **$^1$H NMR** (400 MHz, $CDCl_3$) δ 10.18 (s, 1H), 8.31 (d, $J$ = 5.3 Hz, 1H), 8.11 (d, $J$ = 7.9 Hz, 1H), 7.91 (d, $J$ = 5.3 Hz, 1H), 7.63 (d, $J$ = 8.2 Hz, 1H), 7.57 (t, $J$ = 7.0 Hz, 2H), 7.29 (t, $J$ = 7.0 Hz, 1H), 5.78 – 5.56 (m, 1H), 3.88 (s, 3H), 3.85 (dd, $J$ = 4.5, 1.9 Hz, 1H), 3.68 (s, 3H), 2.75 – 2.64 (m, 1H), 2.35 – 2.25 (m, 1H), 2.10 (s, 3H). **$^{13}$C NMR** (101 MHz, $CDCl_3$) δ 171.1, 170.8, 169.8, 142.2, 140.7, 137.2, 133.2, 129.5, 128.7, 121.6, 121.4, 120.1, 114.5, 112.3, 53.1, 52.8, 49.0, 48.2, 35.3, 23.3. **FTIR** ($\nu_{max}$, $cm^{-1}$): 3216, 3118, 2974, 2908, 2376, 2342, 1774, 1752, 1648, 1581, 1544, 1508, 1482, 1385, 1244, 1070, 900, 820, 738, 666, 559. **HRMS** (ESI-TOF) m/z: calcd for $C_{20}H_{23}N_3O_5^+$ [M + H]$^+$, 384.1554; found, 384.1550. **HPLC analysis:** Chiral MX(2) (150 x 4.6 mm, 3 μm, hexane/i-PrOH = 80:20, 1.0 mL/min, 25 °C, 254 nm), $t_r$ (major) = 3.43 min, $t_r$ (minor) = 13.04 min. **$[\alpha]_D^{20}$** =-29.5 (c = 0.2, $CHCl_3$).

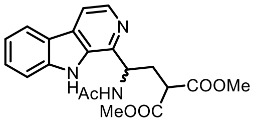


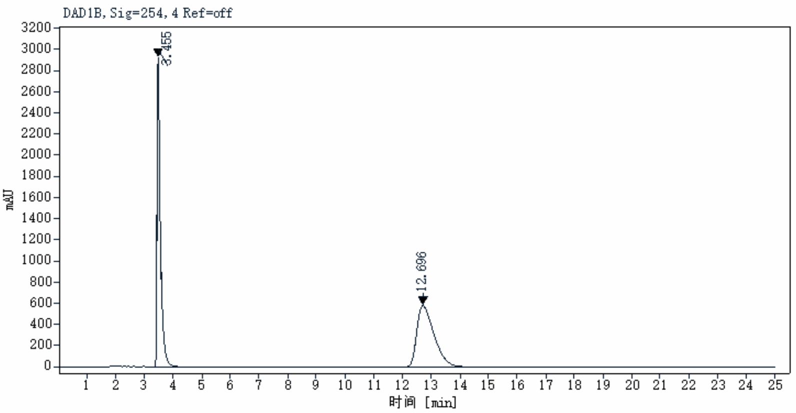


| Entry | Retention Time | Height | Area | Area% |
|---|---|---|---|---|
| 1 | 3.45 | 2932.68 | 23609.77 | 49.02 |
| 2 | 12.69 | 581.92 | 24549.76 | 50.98 |

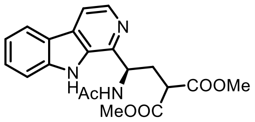


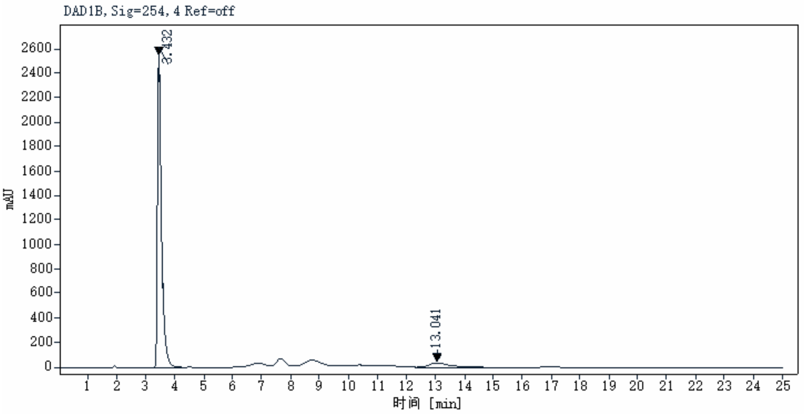


| Entry | Retention Time | Height | Area | Area% |
|---|---|---|---|---|
| 1 | 3.43 | 2545.65 | 24891.76 | 93.13 |
| 2 | 13.04 | 36.34 | 1835.19 | 6.87 |

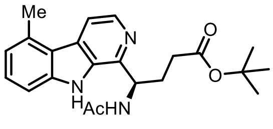

**5b**

($R$)-tert-butyl-4-acetamido-4-(5-methyl-9$H$-pyrido[3,4-$b$]indol-1-yl)butanoate (**5b**) (purification by flash column chromatography: 3% MeOH in $CH_2Cl_2$, 78% yield; 94% ee). **Mp**: 207−209 °C. **$^1$H NMR** (400 MHz, $CDCl_3$) δ 10.53 (s, 1H), 8.32 (d, $J$ = 5.3 Hz, 1H), 7.95 (d, $J$ = 5.4 Hz, 1H), 7.50 – 7.39 (m, 2H), 7.29 (s, 1H), 7.05 (d, $J$ = 6.2 Hz, 1H), 5.76 (td, $J$ = 8.4, 4.3 Hz, 1H), 2.86 (s, 3H), 2.69 – 2.57 (m, 1H), 2.43 – 2.30 (m, 2H), 2.20 – 2.12 (m, 1H), 2.09 (s, 3H), 1.52 (s, 9H). **$^{13}$C NMR** (101 MHz, $CDCl_3$) δ 174.0, 170.7, 143.0, 140.8, 137.1, 134.6, 133.6, 129.6, 128.3, 121.2, 120.5, 116.1, 109.6, 81.2, 49.3, 31.9, 30.3, 28.1, 23.4, 20.5. **FTIR** ($\nu_{max}$, cm$^{-1}$): 3298, 3155, 2920, 2879, 2384, 2337, 1653, 1459, 1273, 1108, 731, 520. **HRMS** (ESI-TOF) m/z: calcd for $C_{22}H_{28}N_3O_3^+$ [M + H]$^+$, 382.2125; found, 382.2121. **HPLC analysis:** Chiral MX(2) (150 x 4.6 mm, 3 μm, hexane/i-PrOH = 70:30, 1.0 mL/min, 25 °C, 254 nm), $t_r$ (major) = 3.06 min, $t_r$ (minor) = 18.05 min. **[α]$_D^{20}$** = -31.2 (c = 0.2, $CHCl_3$).

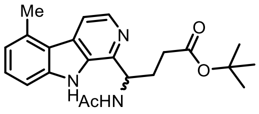

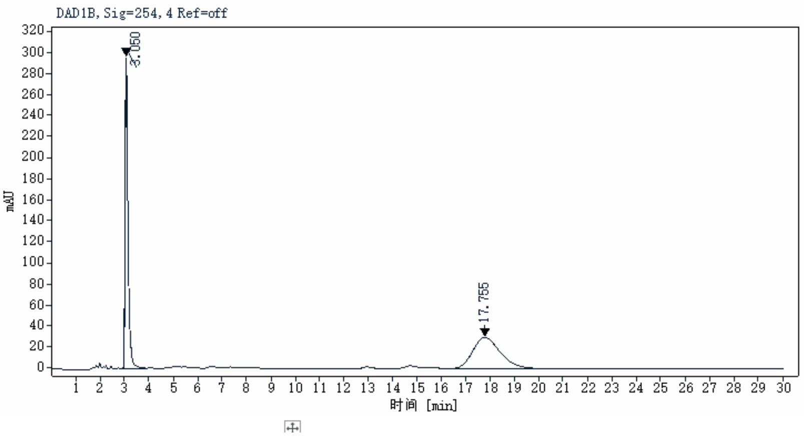


| Entry | Retention Time | Height | Area | Area% |
|---|---|---|---|---|
| 1 | 3.05 | 295.72 | 2439.86 | 50.70 |
| 2 | 17.76 | 29.71 | 2372.93 | 49.30 |

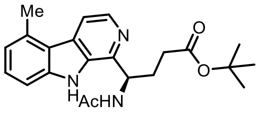

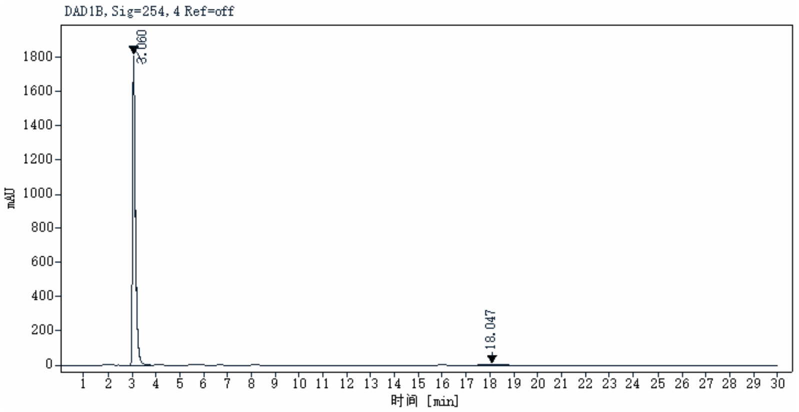


| Entry | Retention Time | Height | Area | Area% |
|---|---|---|---|---|
| 1 | 3.06 | 1813.59 | 14936.63 | 96.77 |
| 2 | 18.05 | 6.50 | 498.87 | 3.23 |

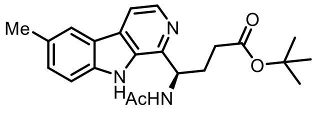

**5c**

(*R*)-tert-butyl-4-acetamido-4-(6-methyl-9*H*-pyrido[3,4-*b*]indol-1-yl)butanoate (**5c**) (purification by flash column chromatography: 3% MeOH in $CH_2Cl_2$, 75% yield; 93% ee). **Mp**: 192−195 °C. **$^1$H NMR** (400 MHz, $CDCl_3$) δ 10.40 (s, 1H), 8.28 (d, *J* = 5.4 Hz, 1H), 7.89 (s, 1H), 7.86 (d, *J* = 5.4 Hz, 1H), 7.48 (d, *J* = 8.4 Hz, 1H), 7.38 (d, *J* = 8.5 Hz, 2H), 5.75 (td, *J* = 8.5, 4.4 Hz, 1H), 2.65 – 2.55 (m, 1H), 2.53 (s, 3H), 2.42 – 2.29 (m, 2H), 2.22 – 2.13 (m, 1H), 2.08 (s, 3H), 1.51 (s, 9H). **$^{13}$C NMR** (101 MHz, $CDCl_3$) δ 173.9, 170.8, 143.1, 139.2, 136.4, 133.9, 130.3, 129.4, 129.3, 121.5, 121.2, 114.1, 111.9, 81.2, 49.2, 31.8, 30.1, 28.1, 23.4, 21.4. **FTIR** ($\nu_{max}$, $cm^{-1}$): 3157, 2983, 2923, 2858, 2378, 1658, 1560, 1508, 1448, 1371, 1296, 1242, 1152, 1059, 793, 620, 564. **HRMS** (ESI-TOF) m/z: calcd for $C_{22}H_{28}N_3O_3^+$ [M + H]$^+$, 382.2125; found, 382.2122. **HPLC analysis:** Chiral MX(2) (150 x 4.6 mm, 3 µm, hexane/i-PrOH = 80:20, 1.0 mL/min, 25 $^o$C, 254 nm), $t_r$ (major) = 3.80 min, $t_r$ (minor) = 25.08 min. **$[\alpha]_D^{20}$** = -28.4 (c = 0.2, $CHCl_3$).

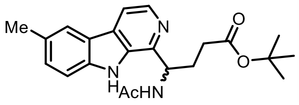

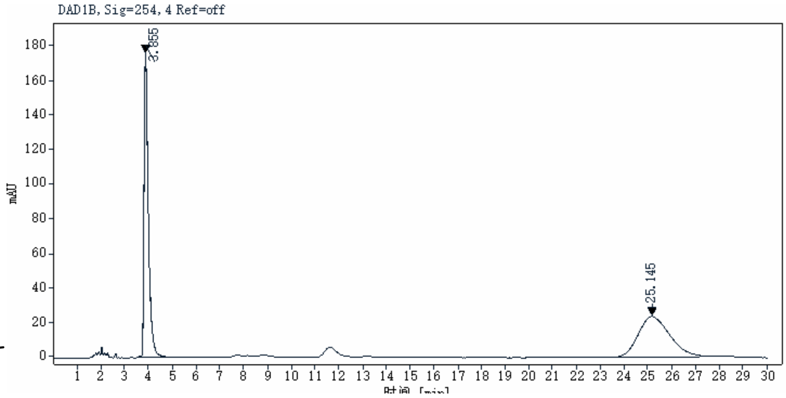


| Entry | Retention Time | Height | Area | Area% |
|---|---|---|---|---|
| 1 | 3.86 | 175.44 | 2197.11 | 50.50 |
| 2 | 25.15 | 23.46 | 2153.33 | 49.50 |

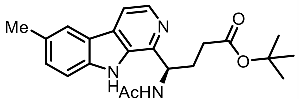

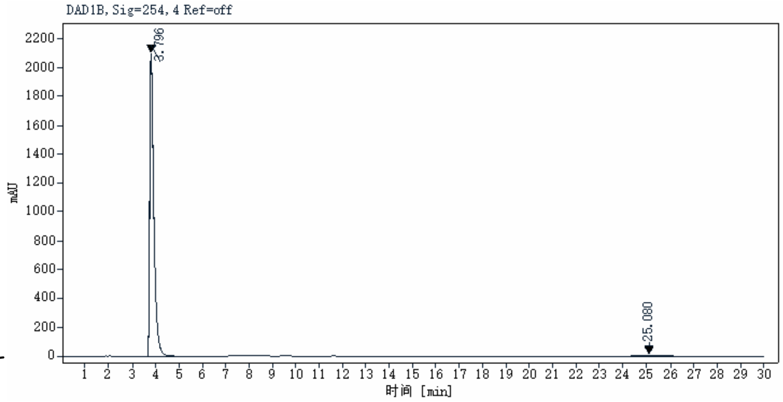


| Entry | Retention Time | Height | Area | Area% |
|---|---|---|---|---|
| 1 | 3.80 | 2099.32 | 25183.91 | 96.44 |
| 2 | 25.08 | 10.02 | 928.07 | 3.56 |

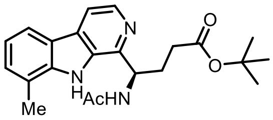

**5d**

($R$)-tert-butyl-4-acetamido-4-(8-methyl-9$H$-pyrido[3,4-$b$]indol-1-yl)butanoate (**5d**) (purification by flash column chromatography: 3% MeOH in $CH_2Cl_2$, 76% yield; 94% ee). **Mp**: 240−242 °C. **$^1$H NMR** (400 MHz, $CDCl_3$) δ 10.72 (s, 1H), 8.30 (d, $J$ = 5.3 Hz, 1H), 7.95 (d, $J$ = 7.9 Hz, 1H), 7.88 (d, $J$ = 5.3 Hz, 1H), 7.41 (d, $J$ = 8.0 Hz, 1H), 7.37 (d, $J$ = 7.1 Hz, 1H), 7.20 (t, $J$ = 7.6 Hz, 1H), 5.76 (td, $J$ = 8.7, 3.3 Hz, 1H), 2.83 – 2.73 (m, 1H), 2.68 (s, 3H), 2.42 – 2.32 (m, 1H), 2.10 (s, 3H), 2.05 – 1.96 (m, 1H), 1.56 (s, 9H). **$^{13}$C NMR** (101 MHz, $CDCl_3$) δ 174.8, 170.7, 143.5, 140.4, 136.9, 133.3, 129.5, 128.7, 121.7, 121.0, 120.0, 119.0, 114.3, 81.5, 49.3, 31.8, 31.4, 28.1, 23.4, 16.7. **FTIR** ($\nu_{max}$, cm$^{-1}$): 3380, 3270, 2919, 2382, 1653, 1542, 1433, 1372, 1288, 1232, 1029, 992, 820, 765, 559. **HRMS** (ESI-TOF) m/z: calcd for $C_{22}H_{28}N_3O_3^+$ [M + H]$^+$, 382.2125; found, 382.2121. **HPLC analysis:** Chiral MX(2) (150 x 4.6 mm, 3 μm, hexane/i-PrOH = 70:30, 1.0 mL/min, 25 °C, 254 nm), $t_r$ (major) = 5.19 min, $t_r$ (minor) = 15.29 min. **[α]$_D^{20}$** = -85.0 (c = 0.2, $CHCl_3$).

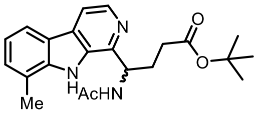

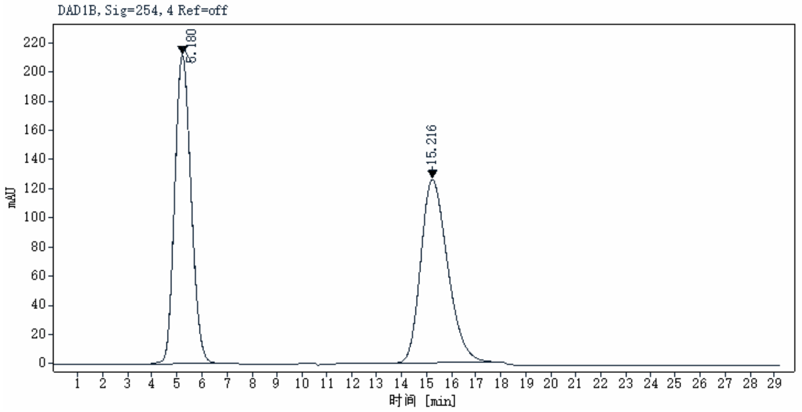


| Entry | Retention Time | Height | Area | Area% |
|---|---|---|---|---|
| 1 | 5.18 | 211.73 | 9652.29 | 50.56 |
| 2 | 15.22 | 125.85 | 9438.18 | 49.44 |

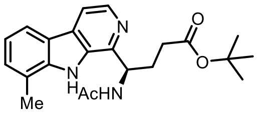

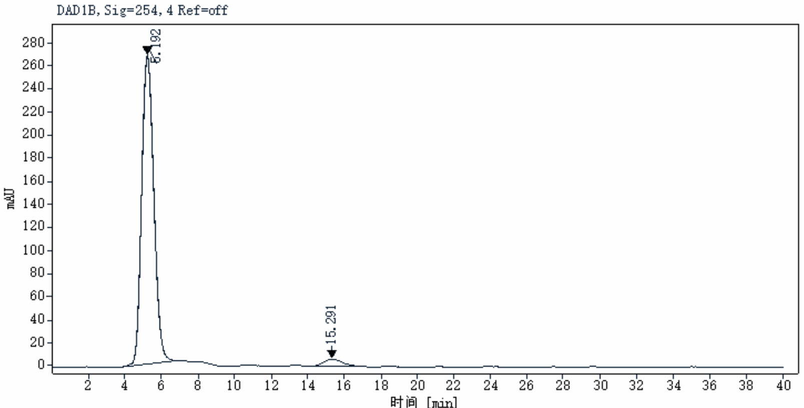


| Entry | Retention Time | Height | Area | Area% |
|---|---|---|---|---|
| 1 | 5.19 | 266.99 | 12322.89 | 97.00 |
| 2 | 15.29 | 5.73 | 381.20 | 3.00 |

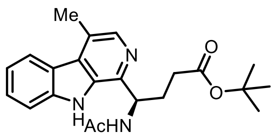

5e

($R$)-tert-butyl-4-acetamido-4-(4-methyl-9$H$-pyrido[3,4-$b$]indol-1-yl)butanoate (**5e**) (purification by flash column chromatography: 3% MeOH in $CH_2Cl_2$, 72% yield; 93% ee). **Mp**: 227−229 °C. **$^1$H NMR** (400 MHz, $CDCl_3$) δ 10.65 (s, 1H), 8.15 (d, $J$ = 7.9 Hz, 1H), 8.07 (s, 1H), 7.58 – 7.50 (m, 2H), 7.45 (d, $J$ = 8.1 Hz, 1H), 7.30 – 7.24 (m, 1H), 5.76 (td, $J$ = 8.3, 4.6 Hz, 1H), 2.80 (s, 3H), 2.62 – 2.51 (m, 1H), 2.43 – 2.30 (m, 2H), 2.24 – 2.15 (m, 1H), 2.08 (s, 3H), 1.48 (s, 9H). **$^{13}$C NMR** (101 MHz, $CDCl_3$) δ 173.7, 170.7, 141.0, 140.7, 137.5, 133.2, 128.0, 127.7, 126.6, 123.3, 122.1, 119.8, 112.0, 81.0, 49.3, 32.0, 30.1, 28.1, 23.4, 17.3. **FTIR** ($\nu_{max}$, $cm^{-1}$): 3298, 3155, 2920, 2879, 2384, 2337, 1653, 1459, 1273, 1108, 731, 520. **HRMS** (ESI-TOF) m/z: calcd for $C_{22}H_{28}N_3O_3^+$ $[M + H]^+$, 382.2125; found, 382.2121. **HPLC analysis:** Chiral MX(2) (150 x 4.6 mm, 3 μm, hexane/i-PrOH = 70:30, 1.0 mL/min, 25 °C, 254 nm), $t_r$ (major) = 3.03 min, $t_r$ (minor) = 13.50 min. **$[\alpha]_D^{20}$** = -23.2 (c = 0.2, $CHCl_3$).

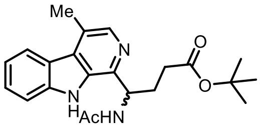

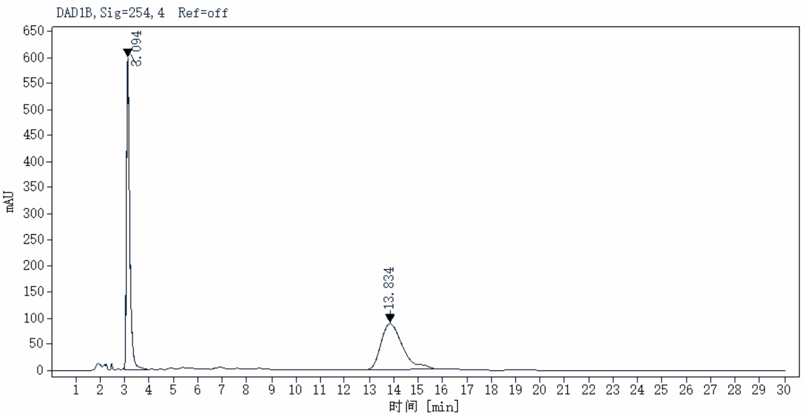


| Entry | Retention Time | Height | Area | Area% |
|---|---|---|---|---|
| 1 | 3.09 | 597.72 | 5486.45 | 50.22 |
| 2 | 13.83 | 88.35 | 5439.32 | 49.78 |

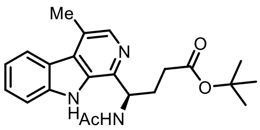

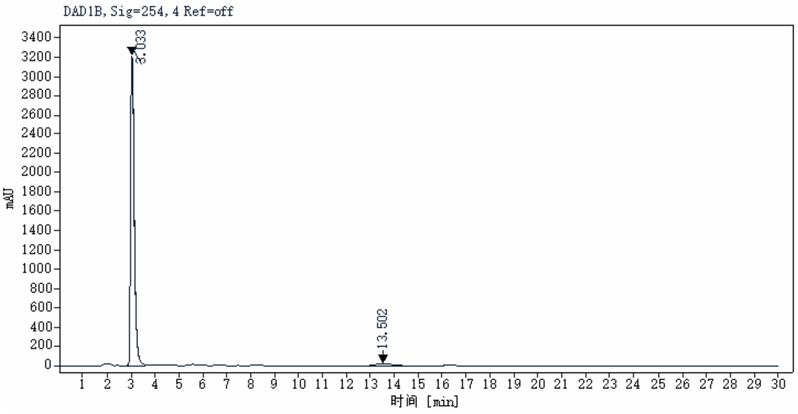


| Entry | Retention Time | Height | Area | Area% |
|---|---|---|---|---|
| 1 | 3.03 | 3212.91 | 34079.39 | 96.46 |
| 2 | 13.50 | 22.48 | 1249.60 | 3.54 |

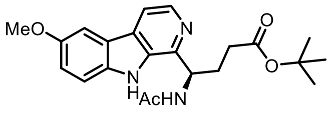

**5f**

(*R*)-tert-butyl-4-acetamido-4-(6-methoxy-9*H*-pyrido[3,4-*b*]indol-1-yl) butanoate(**5f**) (purification by flash column chromatography: 3% MeOH in $CH_2Cl_2$, 76% yield; 93% ee). **Mp**: 190−192 °C. **$^1$H NMR** (400 MHz, $CDCl_3$) δ 10.36 (s, 1H), 8.28 (d, *J* = 5.4 Hz, 1H), 7.84 (d, *J* = 5.4 Hz, 1H), 7.53 (d, *J* = 2.5 Hz, 1H), 7.49 (d, *J* = 8.9 Hz, 1H), 7.29 (s, 1H), 7.21 (dd, *J* = 8.9, 2.5 Hz, 1H), 5.73 (td, *J* = 8.5, 4.3 Hz, 1H), 3.93 (s, 3H), 2.67 – 2.56 (m, 1H), 2.42 – 2.30 (m, 2H), 2.20 – 2.13 (m, 1H), 2.08 (s, 3H), 1.51 (s, 9H). **$^{13}$C NMR** (101 MHz, $CDCl_3$) δ 174.0, 170.7, 154.1, 143.4, 136.5, 135.8, 134.2, 129.1, 121.7, 118.7, 114.1, 113.0, 103.2, 81.2, 56.0, 49.3, 31.9, 30.2, 28.1, 23.4. **FTIR** ($\nu_{max}$, $cm^{-1}$): 3228, 3179, 2925, 2850, 2384, 1641, 1558, 1500, 1437, 1377, 1287, 1290, 1210, 1157, 1024, 804, 625, 557. **HRMS** (ESI-TOF) m/z: calcd for $C_{22}H_{28}N_3O_4^+$ [M + H]$^+$, 398.2074; found, 398.2070. **HPLC analysis:** Chiral MX(2) (150 x 4.6 mm, 3 μm, hexane/i-PrOH = 80:20, 1.0 mL/min, 25 °C, 254 nm), $t_r$ (major) = 8.12 min, $t_r$ (minor) = 25.99 min. **$[\alpha]_D^{20}$** = -16.6 (c = 0.2, $CHCl_3$).

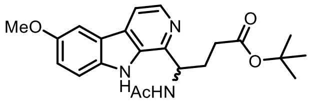

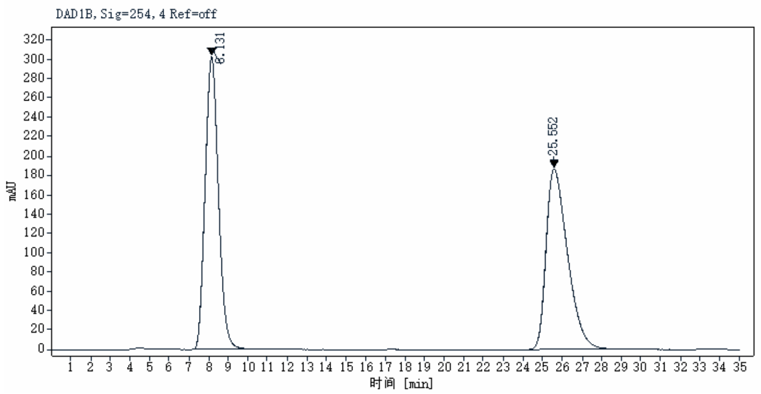


| Entry | Retention Time | Height | Area | Area% |
|---|---|---|---|---|
| 1 | 8.13 | 302.94 | 14205.77 | 50.24 |
| 2 | 25.55 | 186.72 | 14070.49 | 49.76 |

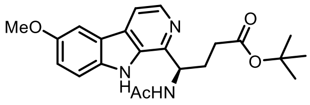

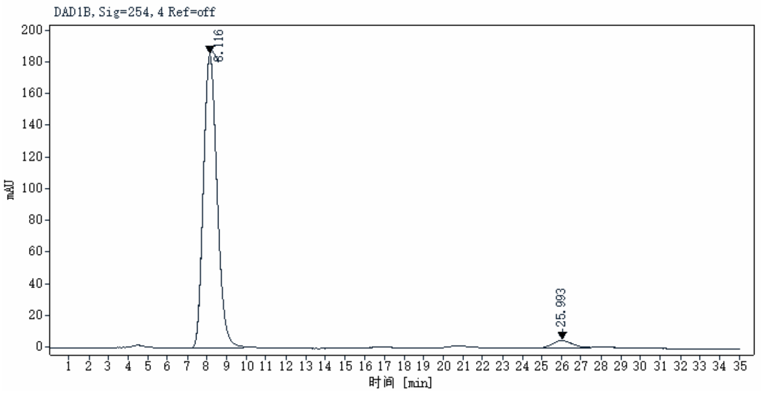


| Entry | Retention Time | Height | Area | Area% |
|---|---|---|---|---|
| 1 | 8.12 | 184.68 | 8884.56 | 96.58 |
| 2 | 25.99 | 4.52 | 314.72 | 3.42 |

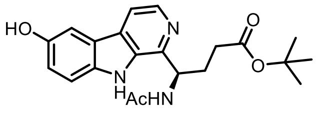

**5g**

(*R*)-tert-butyl-4-acetamido-4-(6-hydroxy-9*H*-pyrido[3,4-*b*]indol-1-yl) butanoate(**5g**) (purification by flash column chromatography: 4% MeOH in $CH_2Cl_2$, 65% yield; 95% ee). **Mp**: 261−263 °C. **$^1$H NMR** (400 MHz, $CDCl_3$) δ 10.12 (s, 1H), 8.14 (d, *J* = 5.4 Hz, 1H), 7.66 (d, *J* = 8.7 Hz, 1H), 7.52 (d, *J* = 5.4 Hz, 1H), 7.25 (d, *J* = 2.4 Hz, 1H), 7.22 (d, *J* = 8.7 Hz, 1H), 7.09 (dd, *J* = 8.7, 2.4 Hz, 1H), 5.70 (td, *J* = 8.8, 4.0 Hz, 1H), 2.59 – 2.49 (m, 1H), 2.38 – 2.26 (m, 2H), 2.11 (s, 3H), 2.09 – 2.02 (m, 1H), 1.49 (s, 9H). **$^{13}$C NMR** (101 MHz, $CDCl_3$) δ 173.8, 171.4, 150.5, 142.9, 135.7, 135.5, 133.9, 129.1, 121.6, 118.9, 114.3, 112.7, 105.9, 81.5, 49.5, 31.7, 30.2, 28.1, 23.4. **FTIR** ($\nu_{max}$, cm$^{-1}$): 2925, 2855, 2381, 1737, 1645, 1464, 1375, 1268, 1025, 820, 759, 723. **HRMS** (ESI-TOF) m/z: calcd for $C_{21}H_{26}N_3O_4^+$ [M + H]$^+$, 384.1918; found, 384.1914. **HPLC analysis:** Chiral MX(2) (150 x 4.6 mm, 3 μm, hexane/i-PrOH = 80:20, 1.0 mL/min, 25 °C, 254 nm), $t_r$ (major) = 12.69 min, $t_r$ (minor)=20.36 min. **[α]$_D^{20}$** = -6.1 (c = 0.2, $CHCl_3$).

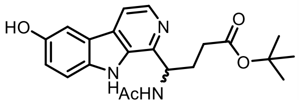

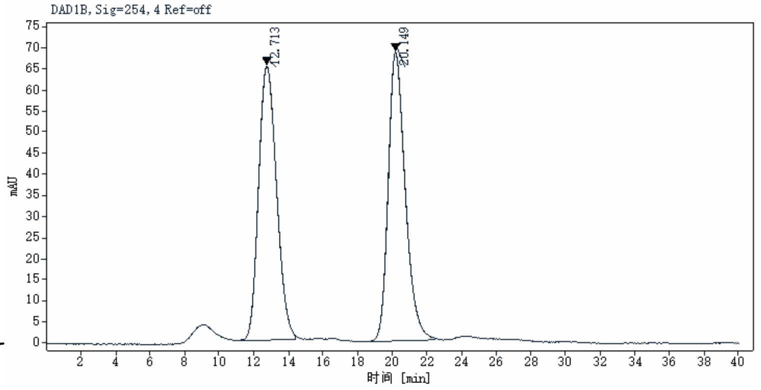


| Entry | Retention Time | Height | Area | Area% |
|---|---|---|---|---|
| 1 | 12.71 | 64.99 | 4695.84 | 50.04 |
| 2 | 20.15 | 68.53 | 4688.64 | 49.96 |

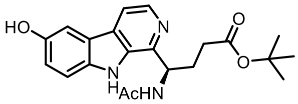

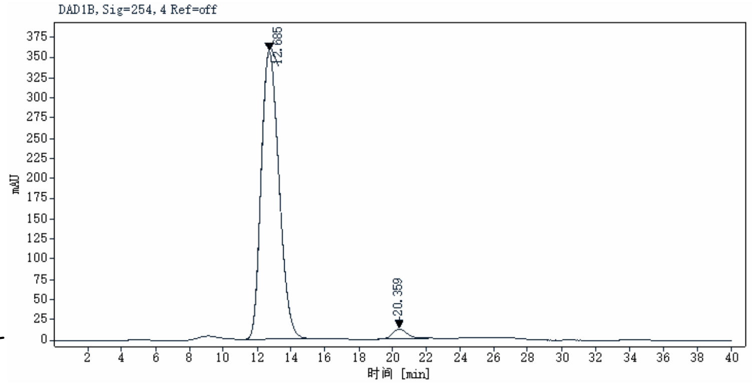


| Entry | Retention Time | Height | Area | Area% |
|---|---|---|---|---|
| 1 | 12.69 | 357.02 | 26158.29 | 97.32 |
| 2 | 20.36 | 12.12 | 719.52 | 2.68 |

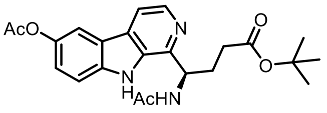

5h

($R$)-tert-butyl-4-acetamido-4-(6-acetoxy-9$H$-pyrido[3,4-$b$]indol-1-yl) butanoate(**5h**) (purification by flash column chromatography: 4% MeOH in $CH_2Cl_2$, 68% yield; 94% ee). **Mp**: 267−270 °C. **$^1$H NMR** (400 MHz, $CDCl_3$) δ 10.71 (s, 1H), 8.28 (d, $J$ = 5.3 Hz, 1H), 7.86 – 7.74 (m, 2H), 7.51 (d, $J$ = 8.8 Hz, 1H), 7.37 (d, $J$ = 8.1 Hz, 1H), 7.25 (dd, $J$ = 8.9, 2.5 Hz, 1H), 5.76 (td, $J$ = 8.3, 4.4 Hz, 1H), 2.65 – 2.53 (m, 1H), 2.43 – 2.29 (m, 5H), 2.22 – 2.12 (m, 1H), 2.09 (s, 3H), 1.49 (s, 9H). **$^{13}$C NMR** (101 MHz, $CDCl_3$) δ 173.9, 170.8, 170.3, 144.0, 143.6, 138.4, 137.2, 134.4, 128.9, 122.5, 121.7, 114.3, 114.0, 112.6, 81.2, 49.5, 31.8, 30.1, 28.1, 23.5, 21.2. **FTIR** ($\nu_{max}$, $cm^{-1}$): 3385, 3289, 2382, 1984, 1650, 1570, 1495, 1379, 1239, 1208, 1024, 992, 820, 648, 582. **HRMS** (ESI-TOF) m/z: calcd for $C_{23}H_{28}N_3O_5^+$ [M + H]$^+$, 426.2023; found, 426.2019. **HPLC analysis:** Chiral MX(2) (150 x 4.6 mm, 3 µm, hexane/i-PrOH = 80:20, 1.0 mL/min, 25 ºC, 254 nm), $t_r$ (major) = 4.18 min, $t_r$ (minor) = 8.95 min. **$[\alpha]_D^{20}$** = -35.5 (c = 0.2, $CHCl_3$).

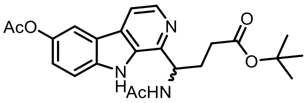

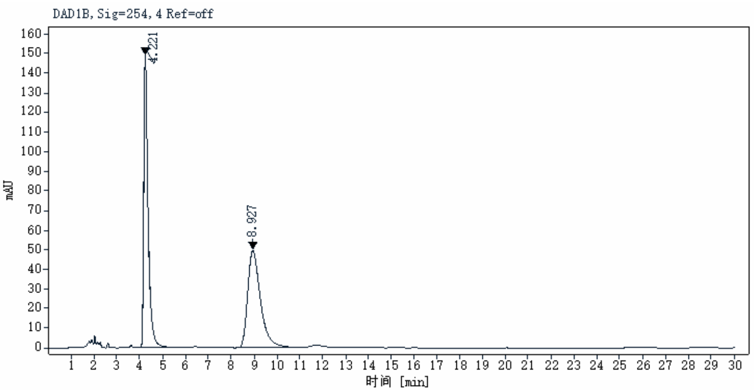


| Entry | Retention Time | Height | Area | Area% |
|---|---|---|---|---|
| 1 | 4.22 | 148.90 | 1935.71 | 50.00 |
| 2 | 8.93 | 49.72 | 1935.65 | 50.00 |

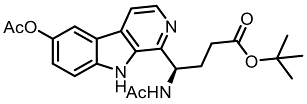

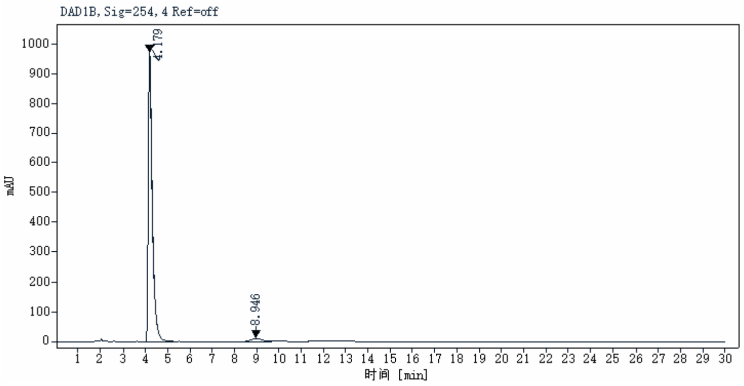


| Entry | Retention Time | Height | Area | Area% |
|---|---|---|---|---|
| 1 | 4.18 | 969.87 | 12279.40 | 96.91 |
| 2 | 8.95 | 10.19 | 391.53 | 3.09 |

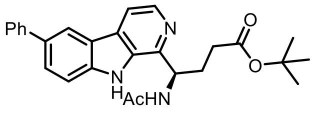

5i

($R$)-tert-butyl-4-acetamido-4-(6-phenyl-9$H$-pyrido[3,4-$b$]indol-1-yl)butanoate (**5i**) (purification by flash column chromatography: 3% MeOH in $CH_2Cl_2$, 76% yield; 92% ee). **Mp**: 181−183 °C. **$^1$H NMR** (400 MHz, $CDCl_3$) δ 10.75 (s, 1H), 8.33 (d, $J$ = 5.3 Hz, 1H), 8.27 (s, 1H), 7.91 (d, $J$ = 5.3 Hz, 1H), 7.78 (d, $J$ = 8.4 Hz, 1H), 7.68 (d, $J$ = 7.6 Hz, 2H), 7.57 (d, $J$ = 8.5 Hz, 1H), 7.48 (t, $J$ = 7.4 Hz, 3H), 7.35 (t, $J$ = 7.3 Hz, 1H), 5.82 (td, $J$ = 8.3, 4.3 Hz, 1H), 2.64 – 2.53 (m, 1H), 2.47 – 2.32 (m, 2H), 2.28 – 2.17 (m, 1H), 2.11 (s, 3H), 1.50 (s, 9H). **$^{13}$C NMR** (101 MHz, $CDCl_3$) δ 173.7, 170.8, 143.5, 141.6, 140.2, 137.3, 134.2, 133.4, 129.4, 128.8, 128.1, 127.3, 126.7, 122.0, 119.9, 114.2, 112.3, 81.1, 49.6, 31.9, 30.1, 28.1, 23.4. **FTIR** ($\nu_{max}$, $cm^{-1}$): 3220, 3177, 2988, 2906, 2384, 2347, 1650, 1558, 1493, 1375, 1239, 1062, 813, 748, 700, 598. **HRMS** (ESI-TOF) m/z: calcd for $C_{27}H_{30}N_3O_3^+$ [M + H]$^+$, 444.2282; found, 444.2277. **HPLC analysis:** Chiral MX(2) (150 x 4.6 mm, 3 μm, hexane/i-PrOH = 80:20, 1.0 mL/min, 25 °C, 254 nm), $t_r$ (major) = 3.49 min, $t_r$ (minor) =13.51 min. **[α]$_D^{20}$** = -25.7 (c = 0.2, $CHCl_3$).

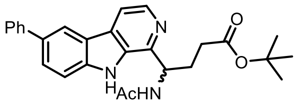

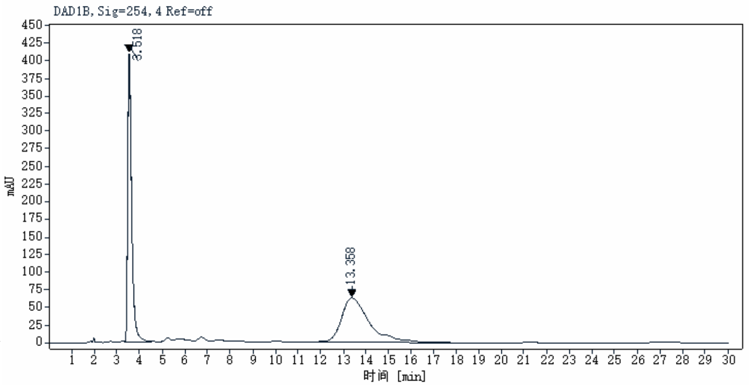


| Entry | Retention Time | Height | Area | Area% |
|---|---|---|---|---|
| 1 | 3.52 | 410.37 | 4968.56 | 47.78 |
| 2 | 13.36 | 63.01 | 5431.28 | 52.22 |

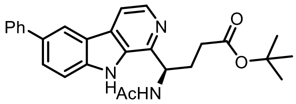

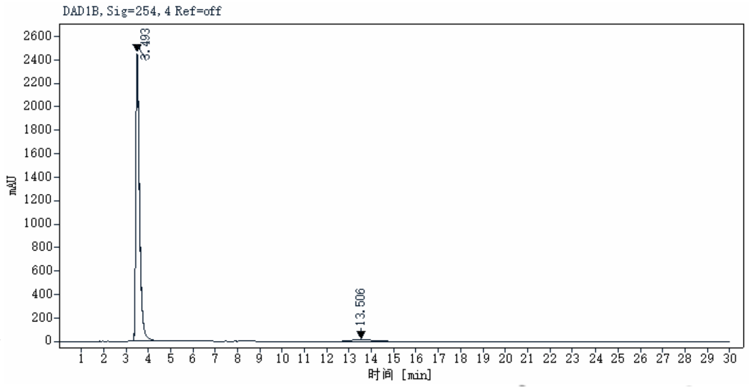


| Entry | Retention Time | Height | Area | Area% |
|---|---|---|---|---|
| 1 | 3.49 | 2453.78 | 28400.08 | 95.83 |
| 2 | 13.51 | 15.38 | 1235.89 | 4.17 |

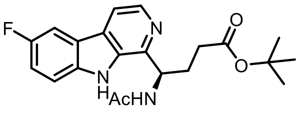


**5j**

(*R*)-tert-butyl-4-acetamido-4-(6-fluoro-9*H*-pyrido[3,4-*b*]indol-1-yl)butanoate (**5j**) (purification by flash column chromatography: 3% MeOH in $CH_2Cl_2$, 72% yield; 87% ee). **Mp**: 220–223 °C. **$^1$H NMR** (400 MHz, $CDCl_3$) δ 10.70 (s, 1H), 8.29 (d, *J* = 5.3 Hz, 1H), 7.81 (d, *J* = 5.3 Hz, 1H), 7.72 (dd, *J* = 8.6, 2.6 Hz, 1H), 7.47 (dd, *J* = 8.9, 4.2 Hz, 1H), 7.41 (d, *J* = 8.3 Hz, 1H), 7.29 (dd, *J* = 9.0, 2.6 Hz, 1H), 5.76 (td, *J* = 8.3, 4.3 Hz, 1H), 2.62 – 2.52 (m, 1H), 2.43 – 2.29 (m, 2H), 2.23 – 2.13 (m, 1H), 2.08 (s, 3H), 1.48 (s, 9H). **$^{13}$C NMR** (101 MHz, $CDCl_3$) δ 173.8, 170.8, 157.4 (d, *J* = 237.3 Hz), 143.8, 137.1, 137.0, 134.6, 128.9 (d, *J* = 4.5 Hz), 121.8 (d, *J* = 9.9 Hz), 116.8 (d, *J* = 25.8 Hz), 114.3, 112.9 (d, *J* = 9.1 Hz), 106.8 (d, *J* = 23.8 Hz), 81.2, 49.5, 31.9, 30.1, 28.1, 23.5. **$^{19}$F NMR** (376 MHz, $CDCl_3$) δ -123.29 (sextet). **FTIR** ($\nu_{max}$, cm$^{-1}$): 3218, 3175, 3102, 3037, 2838, 2357, 1655, 1580, 1505, 1283, 1159, 816, 620. **HRMS** (ESI-TOF) m/z: calcd for $C_{21}H_{25}FN_3O_3^+$ [M + H]$^+$, 386.1874; found, 386.1870. **HPLC analysis:** Chiral MX(2) (150 x 4.6 mm, 3 µm, hexane/i-PrOH = 80:20, 1.0 mL/min, 25 °C, 254 nm), $t_r$ (major) = 3.09 min, $t_r$ (minor) = 10.45 min. **[α]$_D$$^{20}$** = -41.0 (c = 0.2, $CHCl_3$).

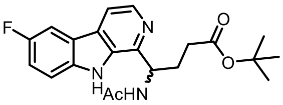


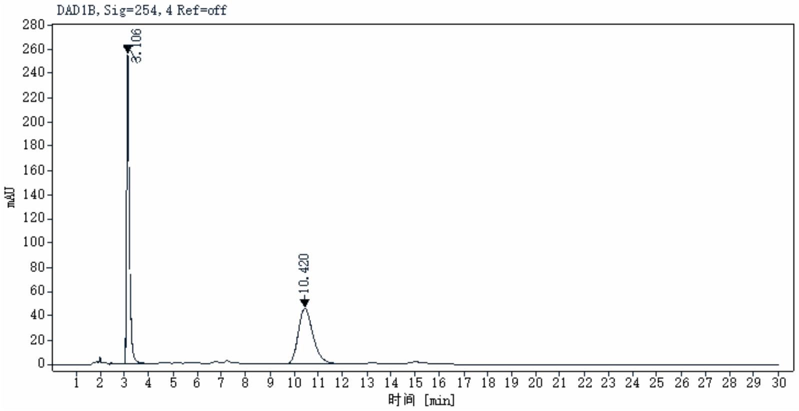


| Entry | Retention Time | Height | Area | Area% |
|---|---|---|---|---|
| 1 | 3.11 | 255.86 | 2024.12 | 50.48 |
| 2 | 10.42 | 46.12 | 1985.77 | 49.52 |

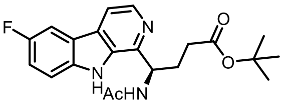


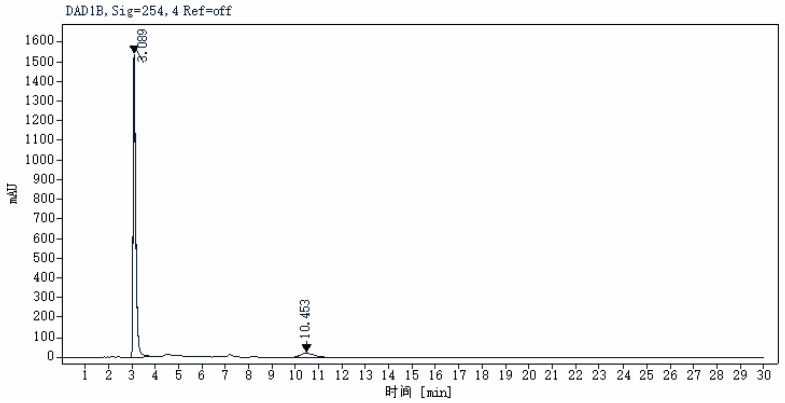


| Entry | Retention Time | Height | Area | Area% |
|---|---|---|---|---|
| 1 | 3.09 | 1536.73 | 11766.60 | 93.26 |
| 2 | 10.45 | 20.19 | 850.37 | 6.74 |

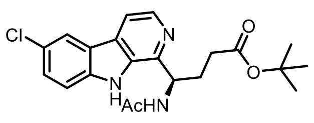

**5k**

(*R*)-tert-butyl-4-acetamido-4-(6-chloro-9*H*-pyrido[3,4-*b*]indol-1-yl)butanoate (**5k**)(purification by flash column chromatography: 3% MeOH in $CH_2Cl_2$, 71% yield; 87% ee). **Mp**: 217−219 °C. **$^1$H NMR** (400 MHz, $CDCl_3$) δ 10.71 (s, 1H), 8.31 (d, *J* = 5.4 Hz, 1H), 8.05 (s, 1H), 7.82 (d, *J* = 5.3 Hz, 1H), 7.48 (s, 2H), 7.29 (d, *J* = 8.6 Hz, 1H), 5.74 (td, *J* = 8.4, 4.3 Hz, 1H), 2.68 – 2.57 (m, 1H), 2.68 – 2.56 (m, 1H), 2.43 – 2.30 (m, 2H), 2.20 – 2.12 (m, 1H), 2.09 (s, 3H), 1.50 (s, 9H). **$^{13}$C NMR** (101 MHz, $CDCl_3$) δ 174.0, 170.8, 143.8, 139.0, 137.4, 134.2, 128.6, 128.3, 125.3, 122.6, 121.2, 114.2, 113.2, 81.3, 49.5, 31.9, 30.1, 28.1, 23.4. **FTIR** ($\nu_{max}$, $cm^{-1}$): 3397, 3279, 2905, 2371, 2287, 2134, 1648, 1387, 1256, 1140, 1021, 985, 782, 576. **HRMS** (ESI-TOF) m/z: calcd for $C_{21}H_{25}ClN_3O_3^+$ [M + H]$^+$, 402.1579; found, 402.1575. **HPLC analysis:** Chiral MX(2) (150 x 4.6 mm, 3 µm, hexane/i-PrOH = 80:20, 1.0 mL/min, 25 °C, 254 nm), $t_r$ (major) = 3.67 min, $t_r$ (minor) = 16.97 min. **$[\alpha]_D^{20}$** = -28.0 (c = 0.2, $CHCl_3$).

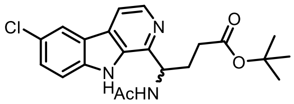

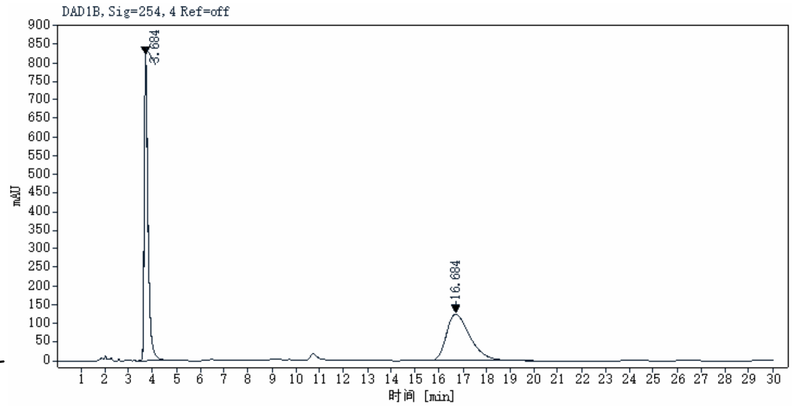


| Entry | Retention Time | Height | Area | Area% |
|---|---|---|---|---|
| 1 | 3.68 | 819.60 | 8939.27 | 51.00 |
| 2 | 16.68 | 125.13 | 8587.45 | 49.00 |

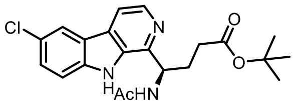

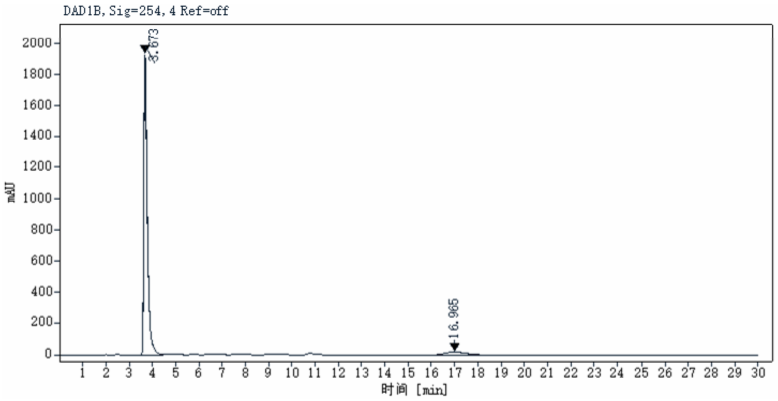


| Entry | Retention Time | Height | Area | Area% |
|---|---|---|---|---|
| 1 | 3.67 | 1934.85 | 20102.95 | 93.59 |
| 2 | 16.97 | 19.44 | 1377.49 | 6.41 |

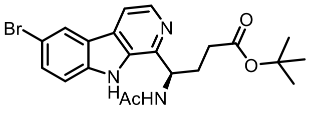

5l

($R$)-tert-butyl-4-acetamido-4-(6-bromo-9$H$-pyrido[3,4-$b$]indol-1-yl)butanoate (**5l**) (purification by flash column chromatography: 3% MeOH in $CH_2Cl_2$, 73% yield; 88% ee). **Mp**: 200−203 °C. **$^1$H NMR** (400 MHz, $CDCl_3$) δ 10.70 (s, 1H), 8.32 (d, $J$ = 5.3 Hz, 1H), 8.21 (d, $J$ = 2.0 Hz, 1H), 7.82 (d, $J$ = 5.3 Hz, 1H), 7.61 (dd, $J$ = 8.7, 1.9 Hz, 1H), 7.44 (d, $J$ = 8.7 Hz, 1H), 7.25 (s, 1H), 5.73 (td, $J$ = 8.5, 4.3 Hz, 1H)., 2.70 – 2.58 (m, 1H), 2.45 – 2.30 (m, 2H), 2.21 – 2.12 (m, 1H), 2.09 (s, 3H), 1.51 (s, 9H). **$^{13}$C NMR** (101 MHz, $CDCl_3$) δ 174.0, 170.8, 143.7, 139.3, 137.4, 134.0, 131.2, 128.2, 124.3, 123.2, 114.2, 113.6, 112.6, 81.3, 49.4, 31.9, 30.2, 28.1, 23.5. **FTIR** ($\nu_{max}$, cm$^{-1}$): 3218, 3165, 2979, 2850, 1653, 1556, 1495, 1375, 1275, 1239, 1070, 818, 801, 610, 582. **HRMS** (ESI-TOF) m/z: calcd for $C_{21}H_{25}BrN_3O_3^+$ [M + H]$^+$, 446.1074; found, 446.1071. **HPLC analysis:** Chiral MX(2) (150 x 4.6 mm, 3 μm, hexane/i-PrOH = 80:20, 1.0 mL/min, 25 ºC, 254 nm), $t_r$ (major) = 2.66 min, $t_r$ (minor) = 6.58 min. **[α]$_D^{20}$** = -42.2 (c = 0.2, $CHCl_3$).

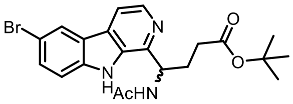

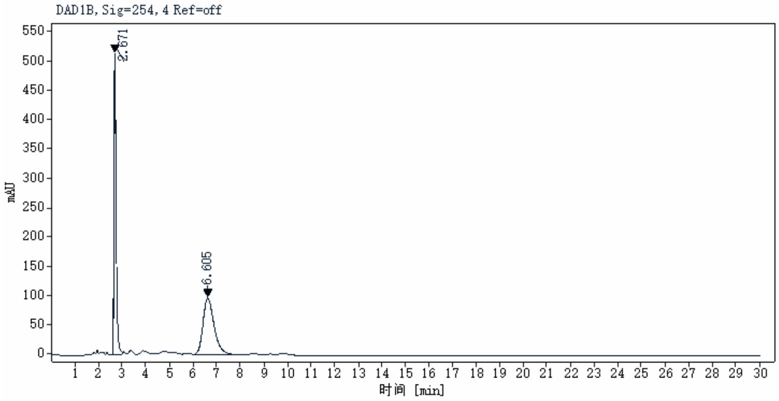


| Entry | Retention Time | Height | Area | Area% |
|---|---|---|---|---|
| 1 | 2.67 | 513.18 | 3217.47 | 50.64 |
| 2 | 6.60 | 96.20 | 3135.61 | 49.36 |

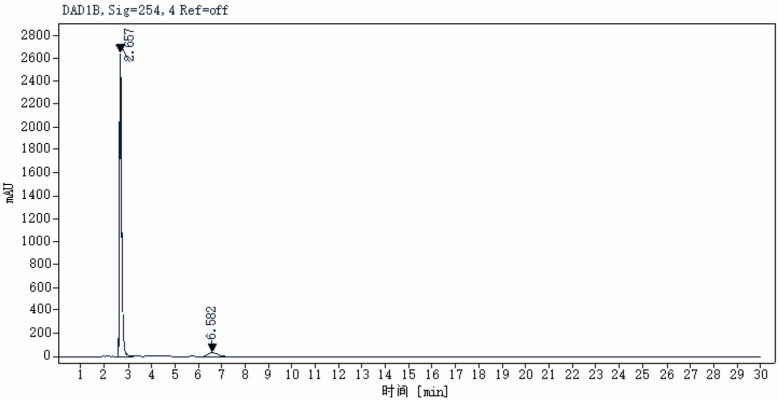


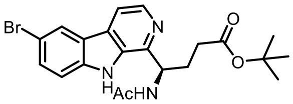

| Entry | Retention Time | Height | Area | Area% |
|---|---|---|---|---|
| 1 | 2.66 | 2643.85 | 16692.26 | 93.83 |
| 2 | 6.58 | 34.68 | 1097.10 | 6.17 |

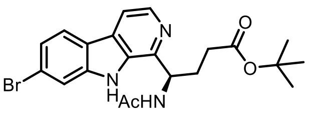

**5m**

($R$)-tert-butyl-4-acetamido-4-(7-bromo-9$H$-pyrido[3,4-$b$]indol-1-yl)butanoate (**5m**) (purification by flash column chromatography: 3% MeOH in $CH_2Cl_2$, 70% yield; 88% ee). **Mp**: 255−257 °C. **$^{1}$H NMR** (400 MHz, $CDCl_3$) δ 10.78 (s, 1H), 8.32 (d, $J$ = 5.3 Hz, 1H), 7.88 (d, $J$ = 8.4 Hz, 1H), 7.80 (d, $J$ = 5.3 Hz, 1H), 7.58 (d, $J$ = 1.6 Hz, 1H), 7.48 (d, $J$ = 8.1 Hz, 1H), 7.33 (dd, $J$ = 8.4, 1.6 Hz, 1H), 5.75 (td, $J$ = 8.5, 4.1 Hz, 1H), 2.65 – 2.54 (m, 1H), 2.42 – 2.30 (m, 2H), 2.22 – 2.13 (m, 1H), 2.11 (s, 3H), 1.49 (s, 9H). **$^{13}$C NMR** (101 MHz, $CDCl_3$) δ 173.9, 170.8, 143.7, 141.4, 137.7, 133.7, 128.7, 123.2, 122.6, 122.0, 120.4, 115.1, 114.0, 81.3, 49.6, 31.9, 30.3, 28.1, 23.5. **FTIR** ($\nu_{max}$, cm$^{-1}$): 3202, 2988, 2974, 2901, 2382, 2342, 1648, 1623, 1560, 1542, 1421, 1375, 1314, 1245, 1078, 1048, 841, 790, 640, 583. **HRMS** (ESI-TOF) m/z: calcd for $C_{21}H_{25}BrN_3O_3^+$ [M + H]$^+$, 446.1074; found, 446.1069. **HPLC analysis:** Chiral MX(2) (150 x 4.6 mm, 3 μm, hexane/i-PrOH = 80:20, 1.0 mL/min, 25 ºC, 254 nm), $t_r$ (major) = 3.30 min, $t_r$ (minor) = 8.34 min. **$[\alpha]_D^{20}$** = -96.0 (c = 0.2, $CHCl_3$).

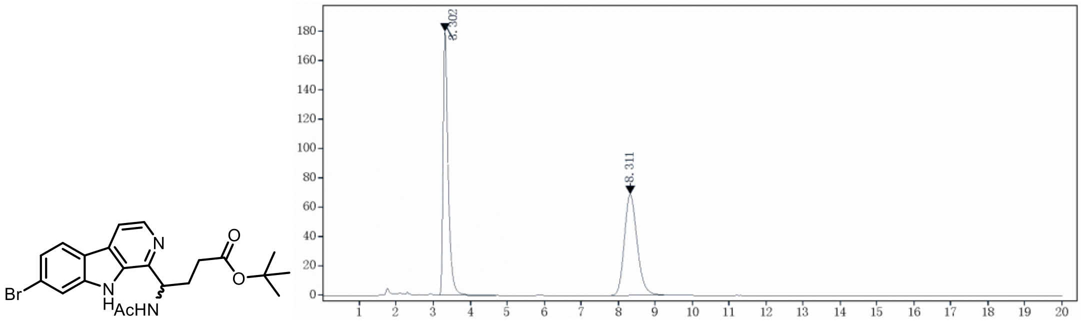

| Entry | Retention Time | Height | Area | Area% |
|---|---|---|---|---|
| 1 | 3.30 | 179.62 | 1681.76 | 50.25 |
| 2 | 8.31 | 68.87 | 1664.97 | 49.75 |

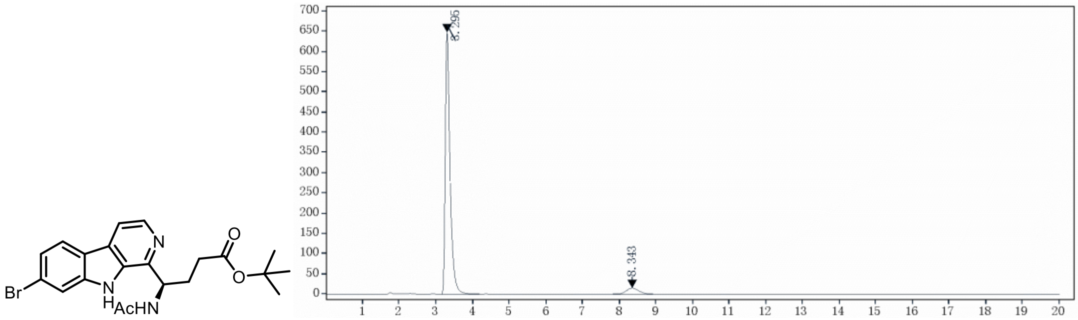

| Entry | Retention Time | Height | Area | Area% |
|---|---|---|---|---|
| 1 | 3.30 | 648.15 | 5832.03 | 93.78 |
| 2 | 8.34 | 15.38 | 386.95 | 6.22 |

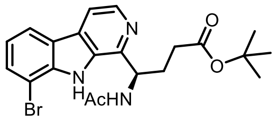


**5n**

($R$)-tert-butyl-4-acetamido-4-(8-bromo-9$H$-pyrido[3,4-$b$]indol-1-yl)butanoate (**5n**) (purification by flash column chromatography: 3% MeOH in $CH_2Cl_2$, 71% yield; 90% ee). **Mp**: 233−235 °C. **$^1$H NMR** (400 MHz, $CDCl_3$) δ 10.85 (s, 1H), 8.34 (d, $J$ = 5.3 Hz, 1H), 8.04 (d, $J$ = 7.8 Hz, 1H), 7.86 (d, $J$ = 5.3 Hz, 1H), 7.72 (d, $J$ = 7.7 Hz, 1H), 7.35 (d, $J$ = 8.3 Hz, 1H), 7.16 (t, $J$ = 7.8 Hz, 1H), 5.79 (td, $J$ = 8.8, 3.3 Hz, 1H), 2.80 – 2.68 (m, 1H), 2.45 – 2.30 (m, 2H), 2.10 (s, 3H), 2.05 – 1.95 (m, 1H), 1.56 (s, 9H). **$^{13}$C NMR** (101 MHz, $CDCl_3$) δ 174.3, 170.8, 144.3, 139.7, 137.7, 133.2, 130.8, 129.5, 122.8, 121.0, 120.6, 114.5, 105.5, 81.5, 49.3, 31.7, 31.1, 28.2, 23.5. **FTIR** ($\nu_{max}$, cm$^{-1}$): 3208, 3157, 2983, 2903, 2383, 2339, 1650, 1542, 1492, 1426, 1370, 1320, 1283, 1133, 835, 769, 675, 600. **HRMS** (ESI-TOF) m/z: calcd for $C_{21}H_{25}BrN_3O_3^+$ [M + H]$^+$, 446.1074; found, 446.1069. **HPLC analysis:** Chiral MX(2) (150 x 4.6 mm, 3 μm, hexane/i-PrOH = 85:15, 1.0 mL/min, 25 °C, 254 nm), $t_r$ (major) = 5.60 min, $t_r$ (minor) = 10.28 min. **[α]$_D^{20}$** = -80.7 (c = 0.2, $CHCl_3$).

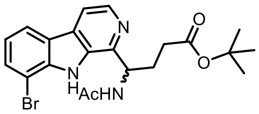


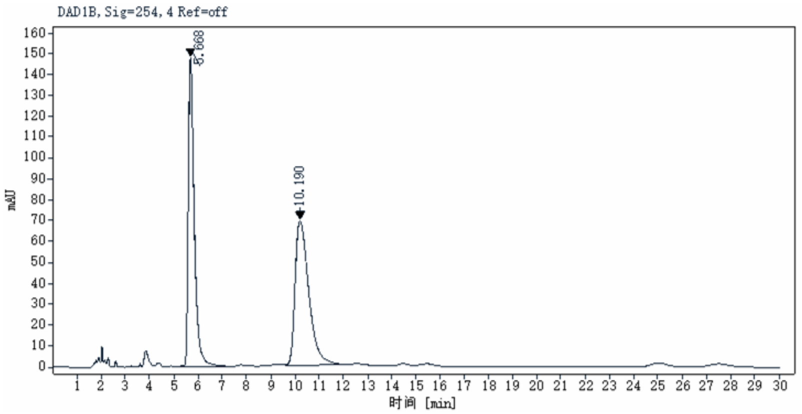


| Entry | Retention Time | Height | Area | Area% |
|---|---|---|---|---|
| 1 | 5.66 | 147.93 | 2506.26 | 47.95 |
| 2 | 10.19 | 69.32 | 2720.33 | 52.05 |

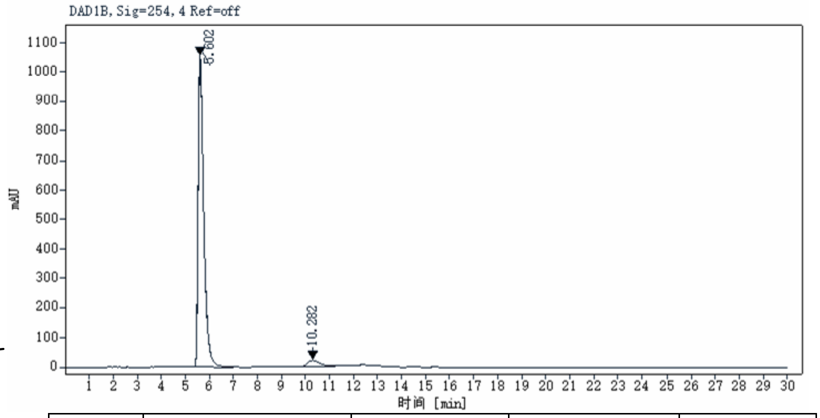


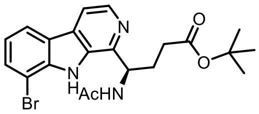


| Entry | Retention Time | Height | Area | Area% |
|---|---|---|---|---|
| 1 | 5.60 | 1052.41 | 17089.63 | 95.10 |
| 2 | 10.28 | 21.98 | 880.62 | 4.90 |

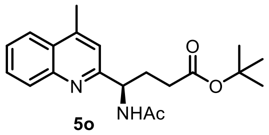

5o

(*R*)-tert-butyl-4-acetamido-4-(4-methylquinolin-2-yl)butanoate (**5o**)[9] (purification by flash column chromatography: 2% MeOH in $CH_2Cl_2$, 82% yield; 93% ee). **$^1$H NMR** (400 MHz, $CDCl_3$) δ 8.04 (d, *J* = 8.4 Hz, 1H), 7.97 (d, *J* = 10.0 Hz, 1H), 7.73 – 7.66 (m, 1H), 7.54 (t, *J* = 7.6 Hz, 1H), 7.34 (d, *J* = 7.5 Hz, 1H), 7.20 (s, 1H), 5.28 – 5.21 (m, 1H), 2.69 (s, 3H), 2.36 – 2.25 (m, 2H), 2.20 – 2.03 (m, 2H), 2.09 (s, 3H), 1.38 (s, 9H). **HPLC analysis:** AD-H (250 x 4.6 mm, 5 µm, hexane/i-PrOH = 90:10, 1.0 mL/min, 25 ºC, 254 nm), $t_r$ (major) = 8.34 min, $t_r$ (minor) = 10.22 min. Ref. 21: **$[\alpha]_D^{20}$** =+10.3 (c = 0.2, $CHCl_3$).

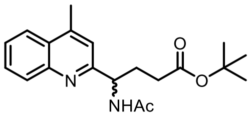

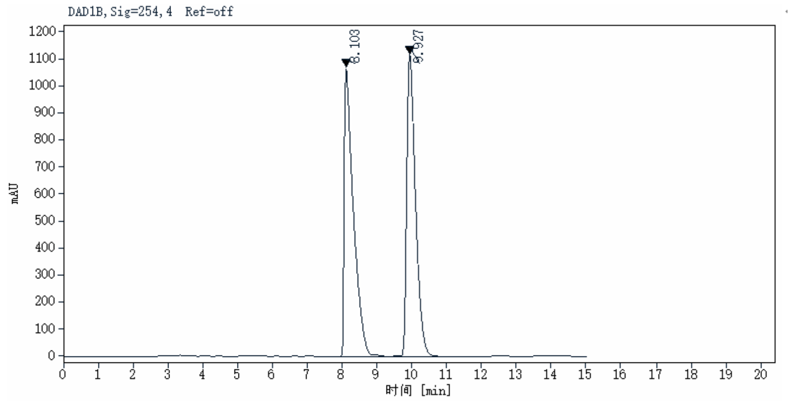


| Entry | Retention Time | Height | Area | Area% |
|---|---|---|---|---|
| 1 | 8.10 | 1065.94 | 19444.67 | 50.05 |
| 2 | 9.93 | 1113.28 | 19404.85 | 49.95 |

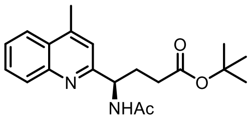

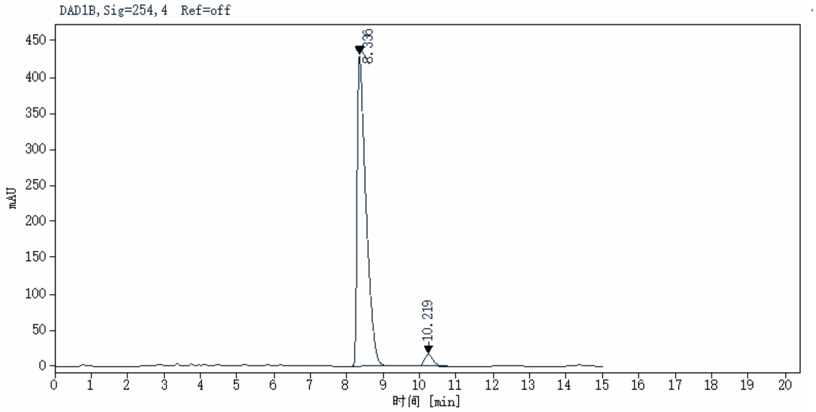


| Entry | Retention Time | Height | Area | Area% |
|---|---|---|---|---|
| 1 | 8.34 | 430.07 | 7231.69 | 96.47 |
| 2 | 10.22 | 15.79 | 264.55 | 3.53 |

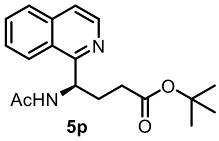

5p

($R$)-tert-butyl-4-acetamido-4-(isoquinolin-1-yl)butanoate (**5p**) (purification by flash column chromatography: 2% MeOH in $CH_2Cl_2$, 76% yield; 88% ee). **$^1$H NMR** (400 MHz, $CDCl_3$) δ 8.42 (d, $J$ = 5.6 Hz, 1H), 8.33 (d, $J$ = 7.6 Hz, 1H), 7.83 (d, $J$ = 9.7 Hz, 1H), 7.72 – 7.62 (m, 2H), 7.58 (d, $J$ = 5.7 Hz, 1H), 7.36 (d, $J$ = 7.9 Hz, 1H), 6.05 (td, $J$ = 7.9, 4.3 Hz, 1H), 2.43 – 2.29 (m, 2H), 2.24 – 2.17 (m, 1H), 2.08 (s, 3H), 2.04 – 1.96 (m, 1H), 1.39 (s, 9H). **$^{13}$C NMR** (101 MHz, $CDCl_3$) δ 172.5, 169.8, 159.1, 141.1, 136.5, 130.5, 127.9, 127.5, 125.6, 124.7, 120.7, 80.4, 48.9, 31.9, 31.5, 28.2, 23.7. **HPLC analysis:** AD-H (250 x 4.6 mm, 5 µm, hexane/i-PrOH = 90:10, 1.0 mL/min, 25 ºC, 254 nm), $t_r$ (major) = 12.16 min, $t_r$ (minor) = 17.81 min. **HRMS** (ESI-TOF) m/z: calcd for $C_{19}H_{25}N_2O_3^+$ $[M + H]^+$, 329.1860; found, 329.1858. **$[\alpha]_D^{20}$** =+17.3 (c = 0.2, $CHCl_3$).

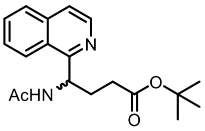


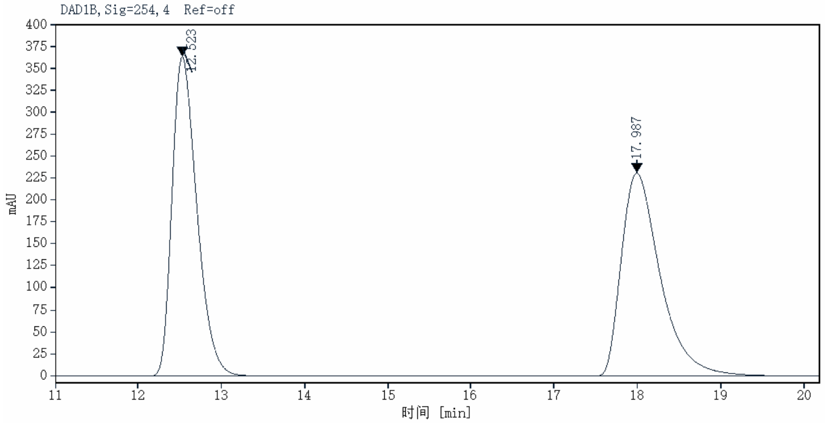


| Entry | Retention Time | Height | Area | Area% |
|---|---|---|---|---|
| 1 | 12.52 | 363.74 | 7548.96 | 50.05 |
| 2 | 17.99 | 231.14 | 7534.02 | 49.95 |

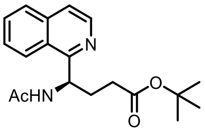


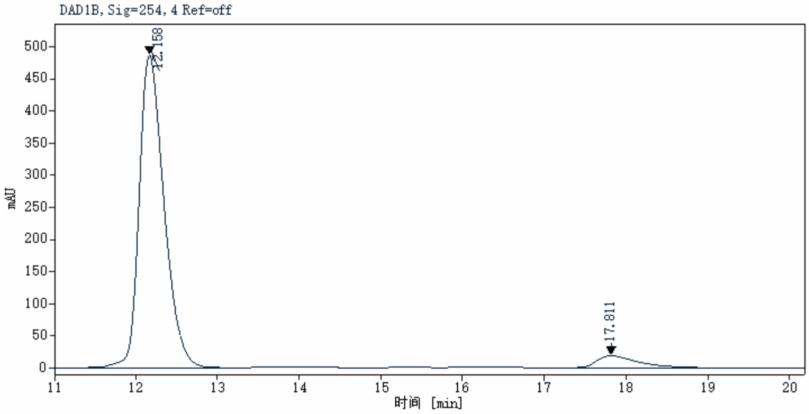


| Entry | Retention Time | Height | Area | Area% |
|---|---|---|---|---|
| 1 | 12.16 | 485.60 | 10256.71 | 93.82 |
| 2 | 17.81 | 18.78 | 676.01 | 6.18 |

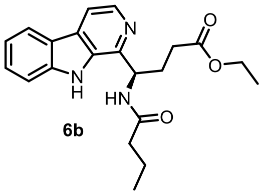

6b

($R$)-ethyl-4-butyramido-4-(9$H$-pyrido[3,4-$b$]indol-1-yl)butanoate (**6b**) (purification by flash column chromatography: 3% MeOH in $CH_2Cl_2$, 75% yield; 89% ee). **Mp**: 155−157 °C. **$^1$H NMR** (400 MHz, $CDCl_3$) δ 10.93 (s, 1H), 8.34 (d, $J$ = 5.2 Hz, 1H), 8.11 (d, $J$ = 7.9 Hz, 1H), 7.90 (d, $J$ = 5.2 Hz, 1H), 7.56 (t, $J$ = 8.5 Hz, 3H), 7.27 (t, $J$ = 7.2 Hz, 1H), 5.87 (td, $J$ = 8.3, 4.4 Hz, 1H), 4.15 – 3.98 (m, 2H), 2.59 – 2.42 (m, 2H), 2.42 – 2.19 (m, 4H), 1.81 – 1.63 (m, 2H), 1.15 (t, $J$ = 7.1 Hz, 3H), 0.93 (t, $J$ = 7.4 Hz, 3H). **$^{13}$C NMR** (101 MHz, $CDCl_3$) δ 173.90, 173.84, 143.30, 140.88, 137.24, 133.84, 129.29, 128.45, 121.57, 121.51, 119.82, 114.28, 112.15, 60.66, 49.37, 38.74, 30.79, 29.79, 19.33, 14.09, 13.75. **FTIR** ($\nu_{max}$, cm$^{-1}$): 3312, 3199, 3061, 2918, 2382, 1752, 1631, 1522, 1389, 1307, 1091, 1050, 716, 646, 537. **HRMS** (ESI-TOF) m/z: calcd for $C_{21}H_{26}N_3O_3^+$ [M + H]$^+$, 368.1969; found, 368.1965. **HPLC analysis:** Chiral MX(2) (150 x 4.6 mm, 3 µm, hexane/i-PrOH = 80:20, 1.0 mL/min, 25 °C, 254 nm), $t_r$ (major) = 5.22 min, $t_r$ (minor) = 17.43 min. **[α]$_D^{20}$** =-28.7 (c = 0.2, $CHCl_3$).

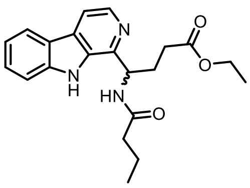

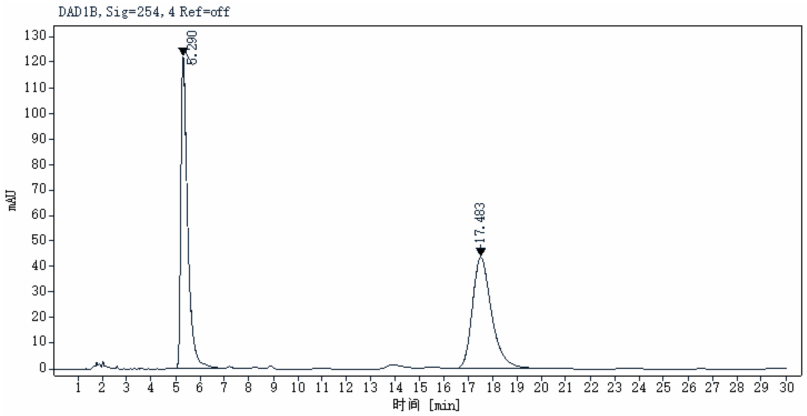


| Entry | Retention Time | Height | Area | Area% |
|---|---|---|---|---|
| 1 | 5.29 | 122.37 | 2385.99 | 50.13 |
| 2 | 17.48 | 43.56 | 2373.87 | 49.87 |

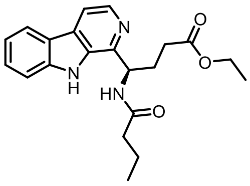

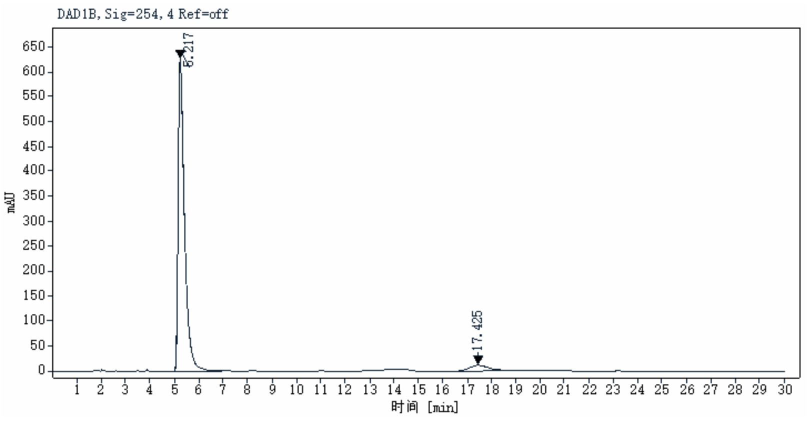


| Entry | Retention Time | Height | Area | Area% |
|---|---|---|---|---|
| 1 | 5.22 | 625.29 | 11425.94 | 94.54 |
| 2 | 17.43 | 11.35 | 659.67 | 5.46 |

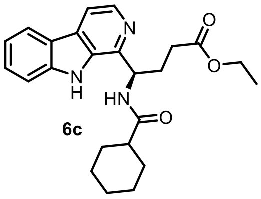

**6c**

(*R*)-ethyl-4-(cyclohexanecarboxamido)-4-(9*H*-pyrido[3,4-*b*]indol-1-yl)butanoate (**6c**) (purification by flash column chromatography: 3% MeOH in $CH_2Cl_2$, 71% yield; 81% ee). **Mp**: 168−170 °C. **$^1$H NMR** (400 MHz, $CDCl_3$) δ 10.82 (s, 1H), 8.34 (d, *J* = 5.3 Hz, 1H), 8.10 (d, *J* = 7.9 Hz, 1H), 7.89 (d, *J* = 5.3 Hz, 1H), 7.63 – 7.48 (m, 2H), 7.41 (d, *J* = 8.3 Hz, 1H), 7.26 (t, *J* = 7.4 Hz, 1H), 5.82 (td, *J* = 8.5, 4.6 Hz, 1H), 4.16 – 3.99 (m, 2H), 2.60 – 2.33 (m, 3H), 2.31 – 2.18 (m, 2H), 1.95 (d, *J* = 12.9 Hz, 1H), 1.88 – 1.71 (m, 3H), 1.67 (d, *J* = 6.2 Hz, 1H), 1.59 – 1.39 (m, 2H), 1.36 – 1.20 (m, 3H), 1.16 (t, *J* = 7.2 Hz, 3H). **$^{13}$C NMR** (101 MHz, $CDCl_3$) δ 177.0, 173.9, 143.4, 140.8, 137.3, 133.8, 129.2, 128.4, 121.6, 121.5, 119.8, 114.2, 112.2, 60.7, 49.1, 45.6, 30.8, 30.0, 29.8, 29.5, 25.8, 25.7, 25.6, 14.1. **FTIR** ($\nu_{max}$, $cm^{-1}$): 2971, 2905, 2359, 1698, 1544, 1505, 1459, 1398, 1165, 1070, 879, 716, 545. **HRMS** (ESI-TOF) m/z: calcd for $C_{24}H_{30}N_3O_3^+$ $[M + H]^+$, 408.2282; found, 408.2278. **HPLC analysis:** Chiral NX(2) (150 x 4.6 mm, 3 μm, hexane/i-PrOH = 80:20, 1.0 mL/min, 25 °C, 254 nm), $t_r$ (major) = 3.71 min, $t_r$ (minor) = 6.76 min. **$[\alpha]_D^{20}$** =-21.5 (c = 0.2, $CHCl_3$).

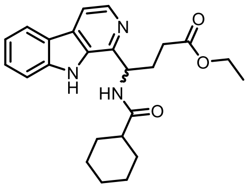

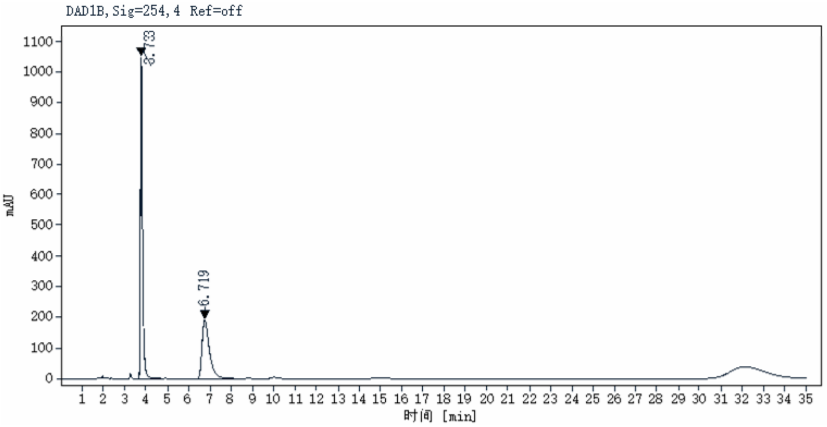


| Entry | Retention Time | Height | Area | Area% |
|---|---|---|---|---|
| 1 | 3.73 | 1050.41 | 6233.40 | 51.48 |
| 2 | 6.72 | 192.13 | 5726.20 | 48.52 |

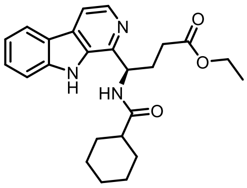

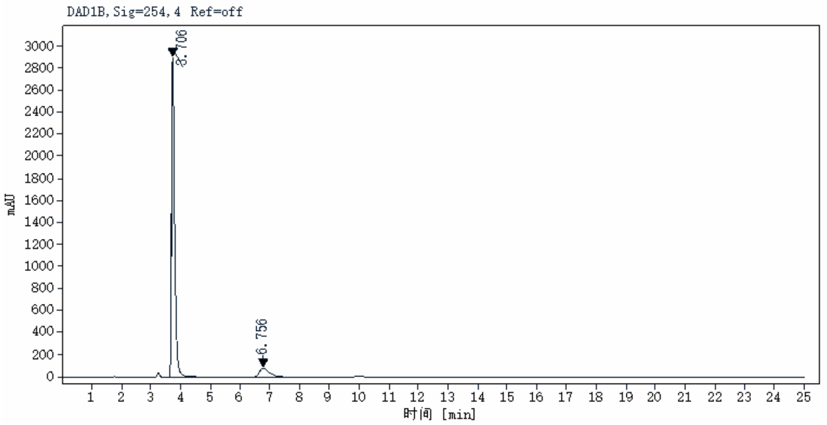


| Entry | Retention Time | Height | Area | Area% |
|---|---|---|---|---|
| 1 | 3.71 | 2900.22 | 21170.83 | 90.67 |
| 2 | 6.76 | 79.87 | 2178.49 | 9.33 |

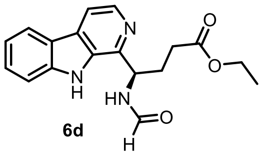

6d

(*R*)-ethyl-4-formamido-4-(9*H*-pyrido[3,4-*b*]indol-1-yl)butanoate (**6d**) (purification by flash column chromatography: 3% MeOH in $CH_2Cl_2$, 61% yield; 35% ee). **Mp**: 138−140 °C. **$^1$H NMR** (400 MHz, $CDCl_3$) δ 10.52 (s, 1H), 8.36 (s, 1H), 8.31 (d, *J* = 5.3 Hz, 1H), 8.10 (d, *J* = 7.9 Hz, 1H), 7.90 (d, *J* = 5.3 Hz, 1H), 7.76 (d, *J* = 8.4 Hz, 1H), 7.62 – 7.51 (m, 2H), 7.29 (d, *J* = 7.9 Hz, 1H), 5.89 (td, *J* = 8.6, 4.0 Hz, 1H), 4.17 (q, *J* = 7.1 Hz, 2H), 2.72 – 2.59 (m, 1H), 2.52 – 2.33 (m, 2H), 2.23 – 2.11 (m, 1H), 1.25 (t, *J* = 7.2 Hz, 3H). **$^{13}$C NMR** (101 MHz, $CDCl_3$) δ 174.3, 161.7, 142.4, 140.9, 137.0, 133.4, 129.6, 128.7, 121.6, 121.4, 120.1, 114.4, 112.2, 61.0, 48.0, 30.6, 30.4, 14.2. **FTIR** ($\nu_{max}$, cm$^{-1}$): 2990, 2896, 2359, 1754, 1672, 1587, 1543, 1505, 1459, 1243, 1052, 730, 619, 571. **HRMS** (ESI-TOF) m/z: calcd for $C_{18}H_{20}N_3O_3^+$ [M + H]$^+$, 326.1499; found, 326.1495. **HPLC analysis:** Chiral MX(2) (150 x 4.6 mm, 3 µm, hexane/i-PrOH = 80:20, 1.0 mL/min, 25 °C, 254 nm), $t_r$ (major) = 12.54 min, $t_r$ (minor) = 18.02 min. **$[\alpha]_D^{20}$** =-31.0 (c = 0.2, $CHCl_3$).

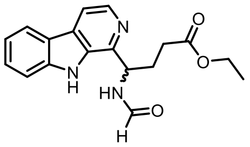

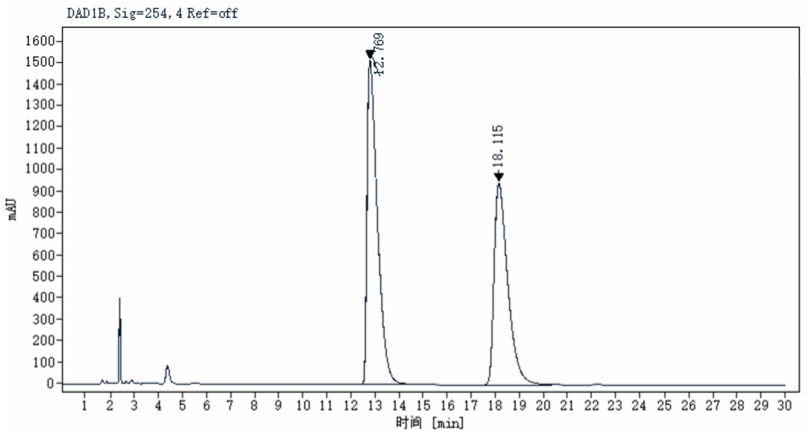


| Entry | Retention Time | Height | Area | Area% |
|---|---|---|---|---|
| 1 | 12.77 | 1517.35 | 45251.51 | 50.13 |
| 2 | 18.12 | 943.98 | 45021.64 | 49.87 |

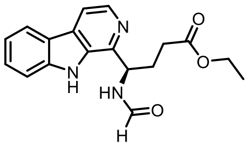

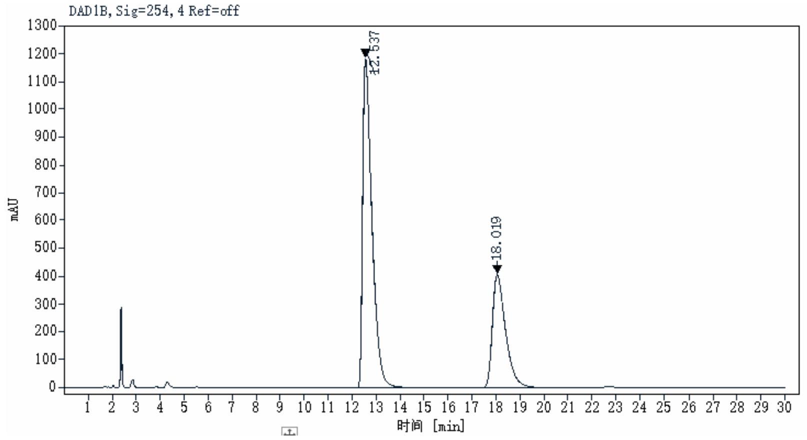


| Entry | Retention Time | Height | Area | Area% |
|---|---|---|---|---|
| 1 | 12.54 | 1183.95 | 32081.57 | 67.30 |
| 2 | 18.02 | 406.86 | 15586.31 | 32.70 |

## 14 References


[1] M.-P. Luo, Y.-J. Gu, S.-G. Wang, *Chem. Sci*. **2023**, *14*, 251-256.

[2] J. T. Brogan, S. L. Stoops, B. C. Crews, L. J. Marnett, C. W. Lindsley, *ACS Chem. Neurosci.* **2011**, *2*, 633-639.

[3] G. Dong, S. Wang, Z. Miao, J. Yao, Y. Zhang, Z. Guo, W. Zhang, C. Sheng, *J. Med. Chem.* **2012**, *55*, 7593-7613.

[4] Y. Yang, C. Zhu, M. Zhang, S. Huang, J. Lin, X. Pan, W. Su, *Chem. Commun.* **2016**, *52*, 12869-12872.

[5] T. Toma, J. Shimokawa, T. Fukuyama, *Org. Lett.* **2007**, *9*, 3195-3197.

[6] T. Ghosh, S. Jana, J. Dash, *Org. Lett*. **2019**, *21*, 6690-6694.

[7] G. La Regina, V. Famiglini, S. Passacantilli, S. Pelliccia, P. Punzi, R. Silvestri, *Synthesis-Stuttgart* **2014**, *46*, 2093-2097.

[8] M. J. Thompson, J. C. Louth, S. M. Little, M. P. Jackson, Y. Boursereau, B. Chen, I. Coldham, *ChemMedChem* **2012**, *7*, 578-586.

[9] D.-Q. Zheng, A. Studer, *Angew. Chem., Int. Ed.* **2019**, *58*, 15803-15807.

[10] J. McNulty, I. W. J. Still, *J. Chem. Soc., Perkin Trans. 1*. **1994**, 1329-1337.

[11] A. S. Kende, H.-Q. Dong, A. W. Mazur, F. H. Ebetino, *Tetrahedron Lett.* **2001**, *42*, 6015-6018.

[12] P. Melnyk, P. Ducrot, L. Demuynck, C. Thal, *Tetrahedron Lett.* **1993**, *34*, 5085-5088.

[13] S. Mahboobi, S. Dove, P. J. Bednarski, S. Kuhr, *J. Nat. Prod.* **1997**, *60*, 587-591.

[14] Y.-L. Su, G.-X. Liu, L. D. Angelis, R. He, A. Al-Sayyed, K. S. Schanze, W.-H. Hu, H. Qiu, M. P. Doyle, *ACS Catal.* **2022**, *12*, 1357-1363.

[15] H. Zhang, R. C. Larock, *J. Org. Chem.* **2002**, *67*, 7048-7056.

[16] G.-X. Liu, X.-T. Jie, X.-l. Li, L.-S. Yang, H. Qiu, W.-H. Hu, *ACS Catal.* **2023**, *13*, 5307-5314.

[17] D. M. Hodgson, D. Angrish, *Chem.-Eur. J.* **2007**, *13*, 3470-3479.

[18] Y.-L. Su, K. Dong, H. Zheng, M. P. Doyle, *Angew. Chem. Int. Ed.* **2021**, *60*, 18484

[19] L. Candish, D. W. Lupton, *J. Am. Chem. Soc.* **2013**, *135*, 58-61.

[20] H. Shimomoto, T. Kudo, S. Tsunematsu, T. Itoh, E. Ihara, *Macromolecules* **2018**, *51*, 328-335.

[21] S. K. Ray, M. M. Sadhu, R. G. Biswas, R. A. Unhale, V. K. Singh, *Org. Lett.* **2019**, *21*, 417-422.

[22] D. Dar'in, G. Kantin, M. Krasavin, *Synthesis* **2019**, *51*, 4284-4290.

[23] S. E. Cleary, X. Li, L.-C. Yang, K. N. Houk, X. Hong, M. Brewer, *J. Am. Chem. Soc.* **2019**, *141*, 3558-3565.

## 15 Copies of NMR spectra

[1]H NMR (400 MHz, DMSO-$d_6$) 5-methyl-9*H*-pyrido[3,4-*b*]indole (**1b**)

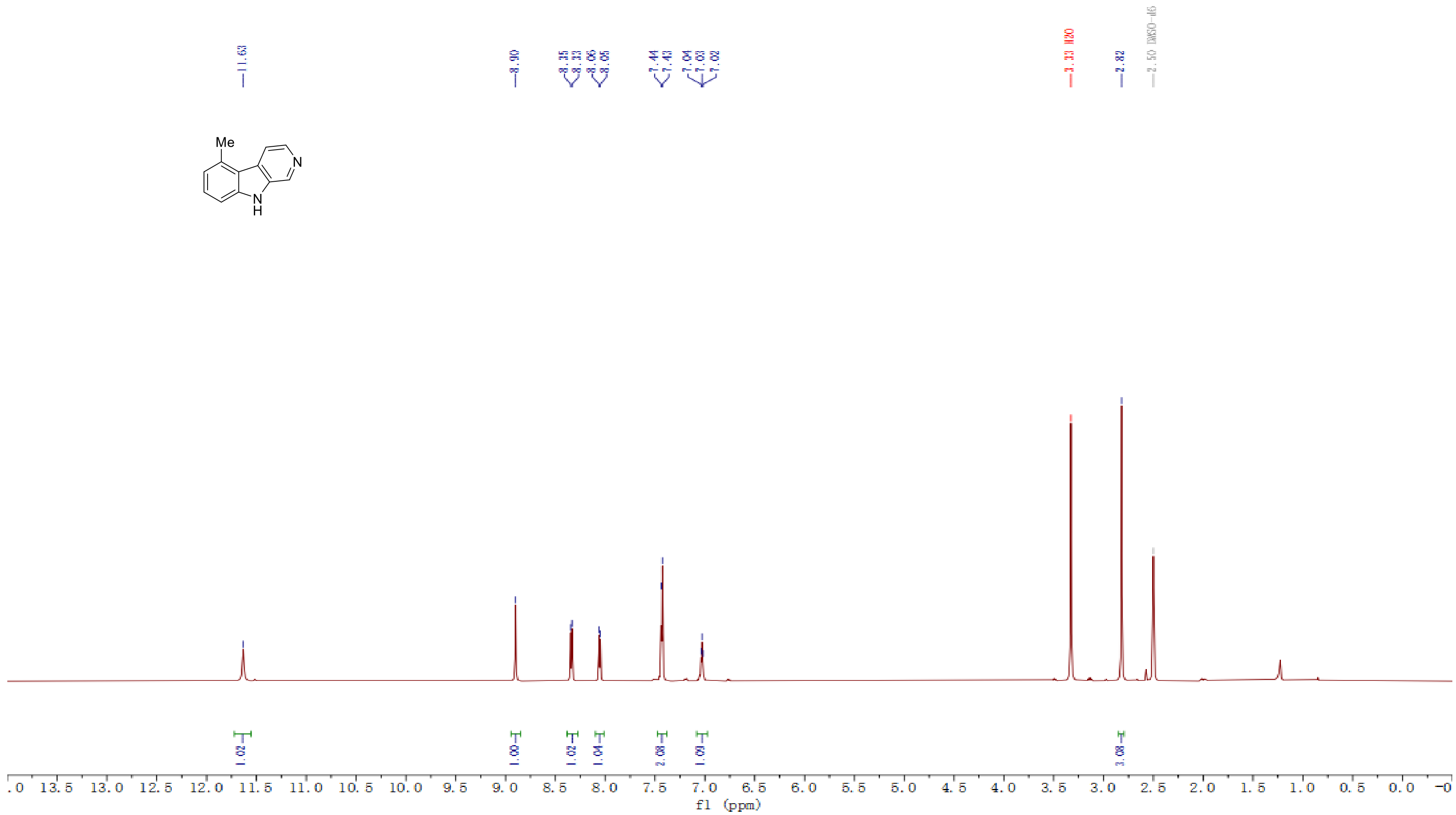

[1]H NMR (400 MHz, DMSO-$d_6$) 6-methyl-9$H$-pyrido[3,4-$b$]indole (**1c**)

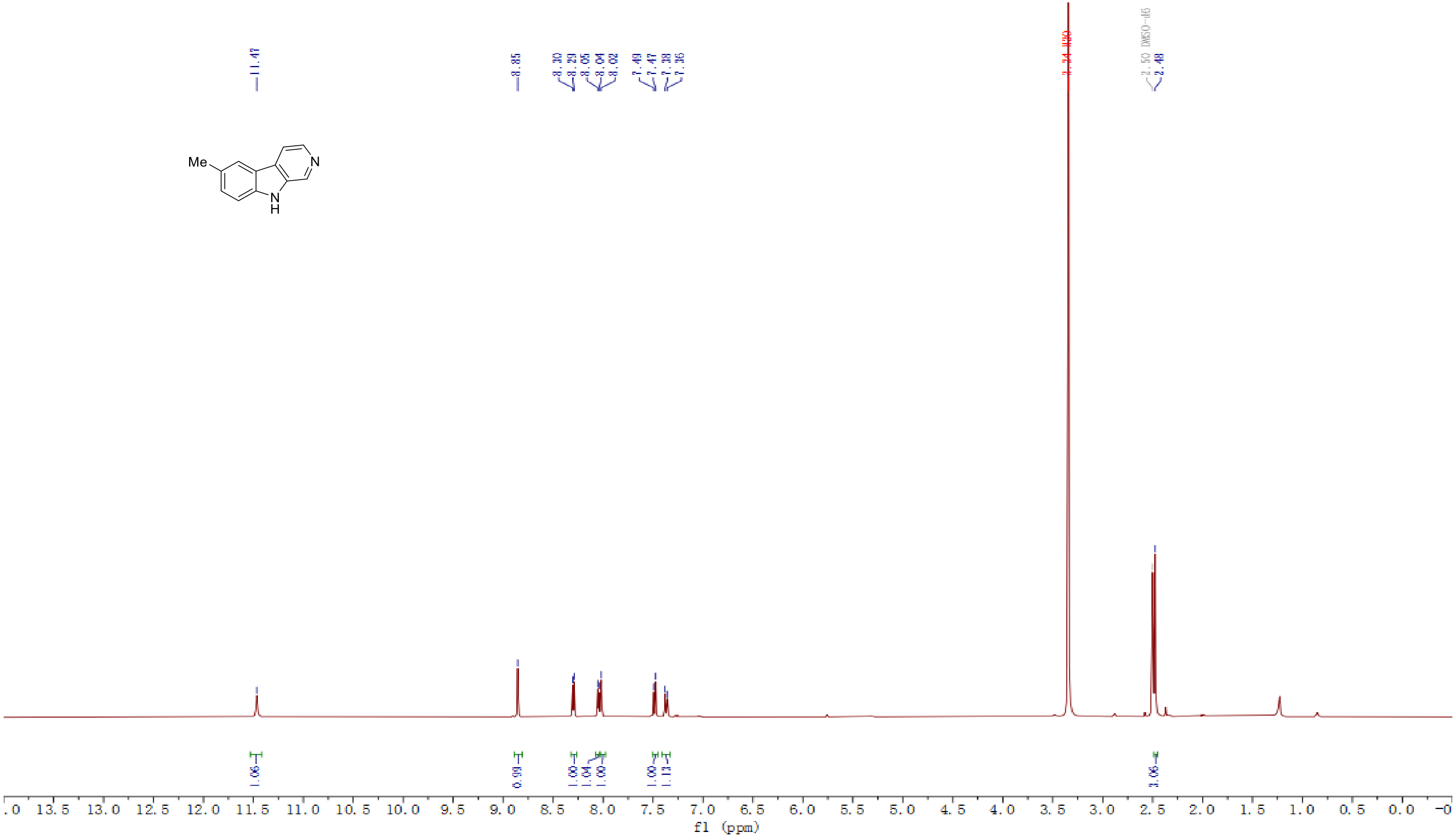

$^{1}$H NMR (400 MHz, DMSO-$d_6$) 8-methyl-9*H*-pyrido[3,4-*b*]indole (**1d**)

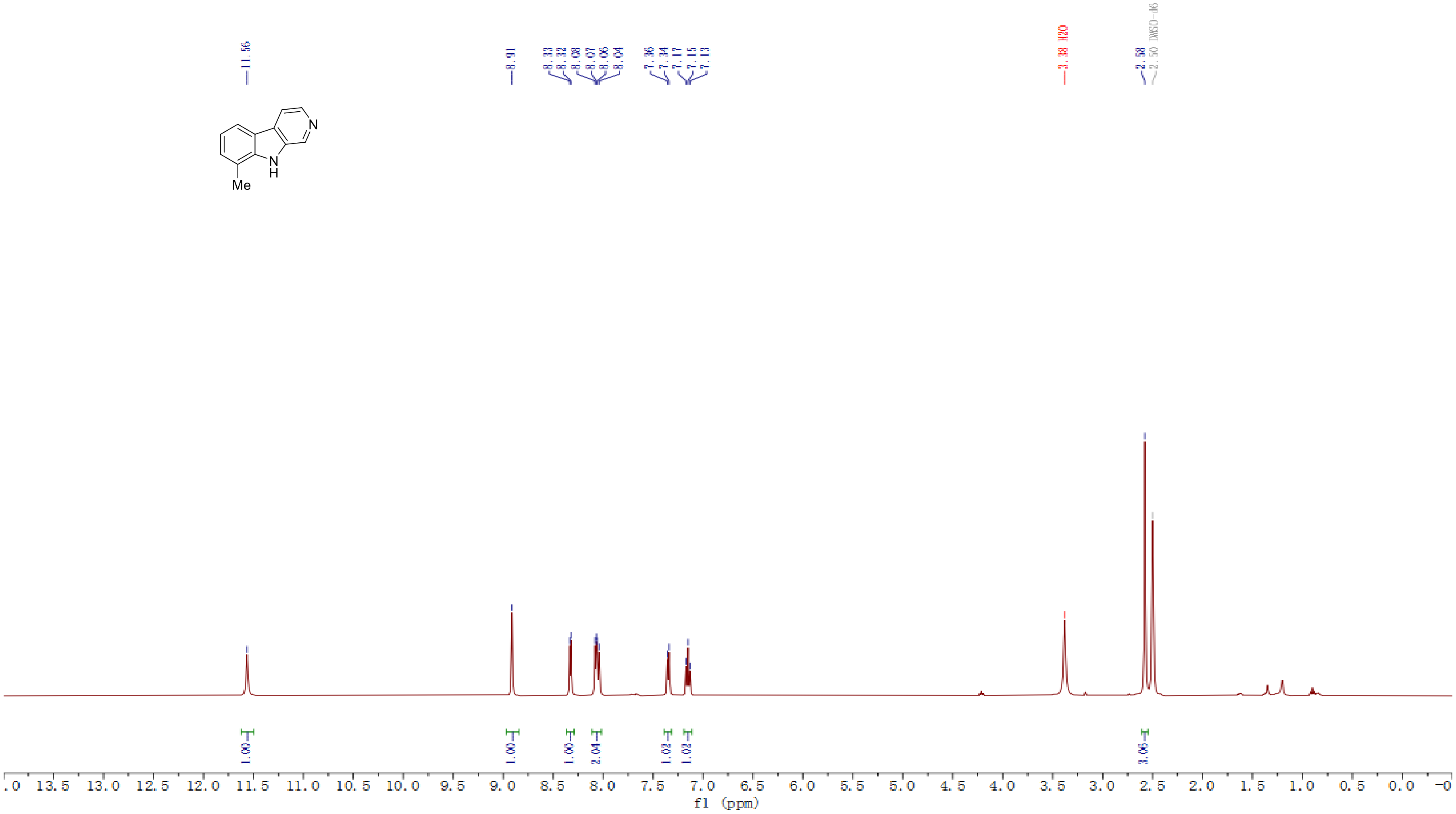

[1]H NMR (400 MHz, DMSO-$d_6$) 4-methyl-9*H*-pyrido[3,4-*b*]indole (**1e**)

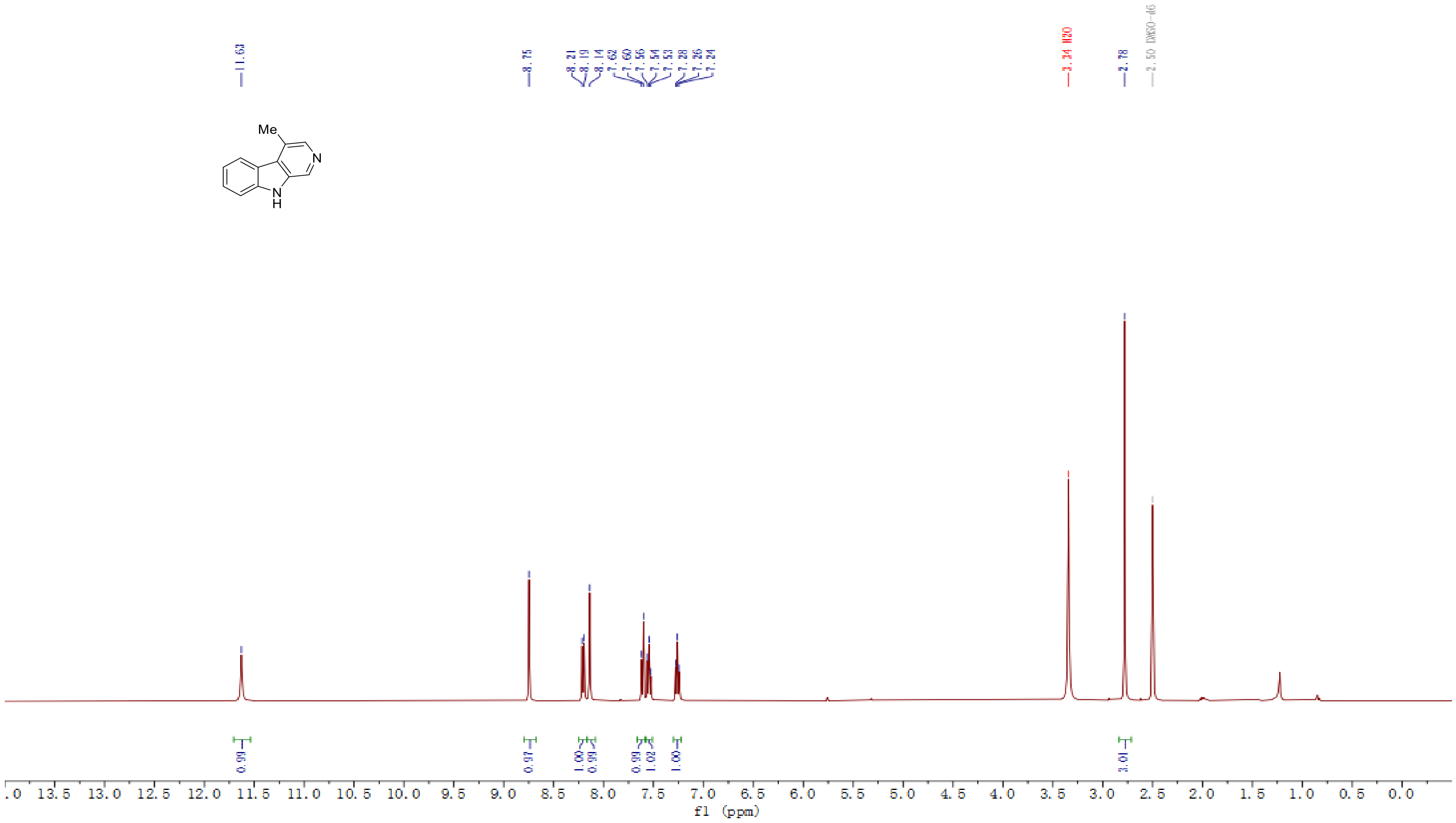

[1]H NMR (400 MHz, DMSO-$d_6$) 6-methoxy-9*H*-pyrido[3,4-*b*]indole (**1f**)

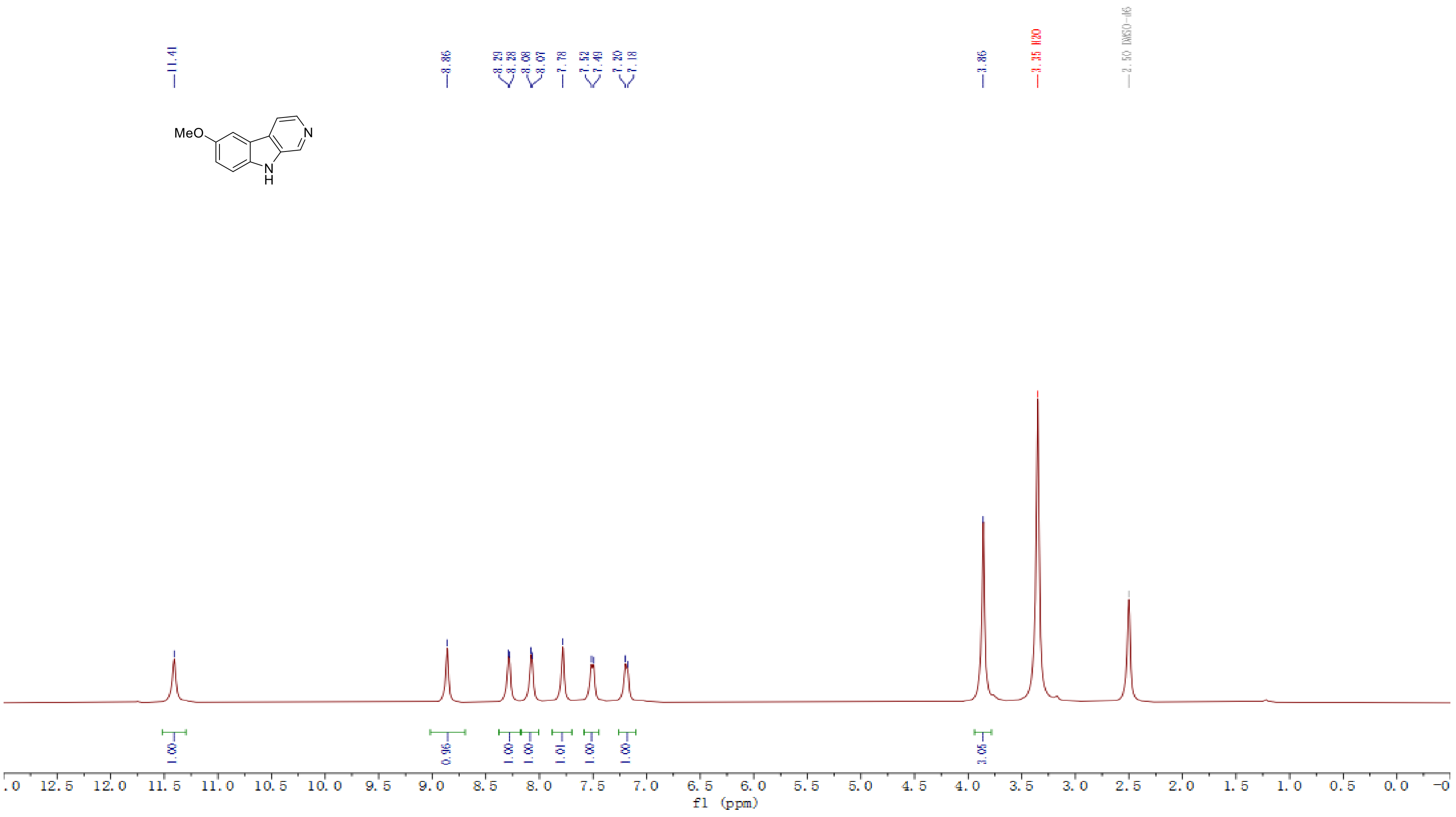

[1]H NMR (400 MHz, DMSO-$d_6$) 9*H*-pyrido[3,4-*b*]indol-6-ol (**1g**)

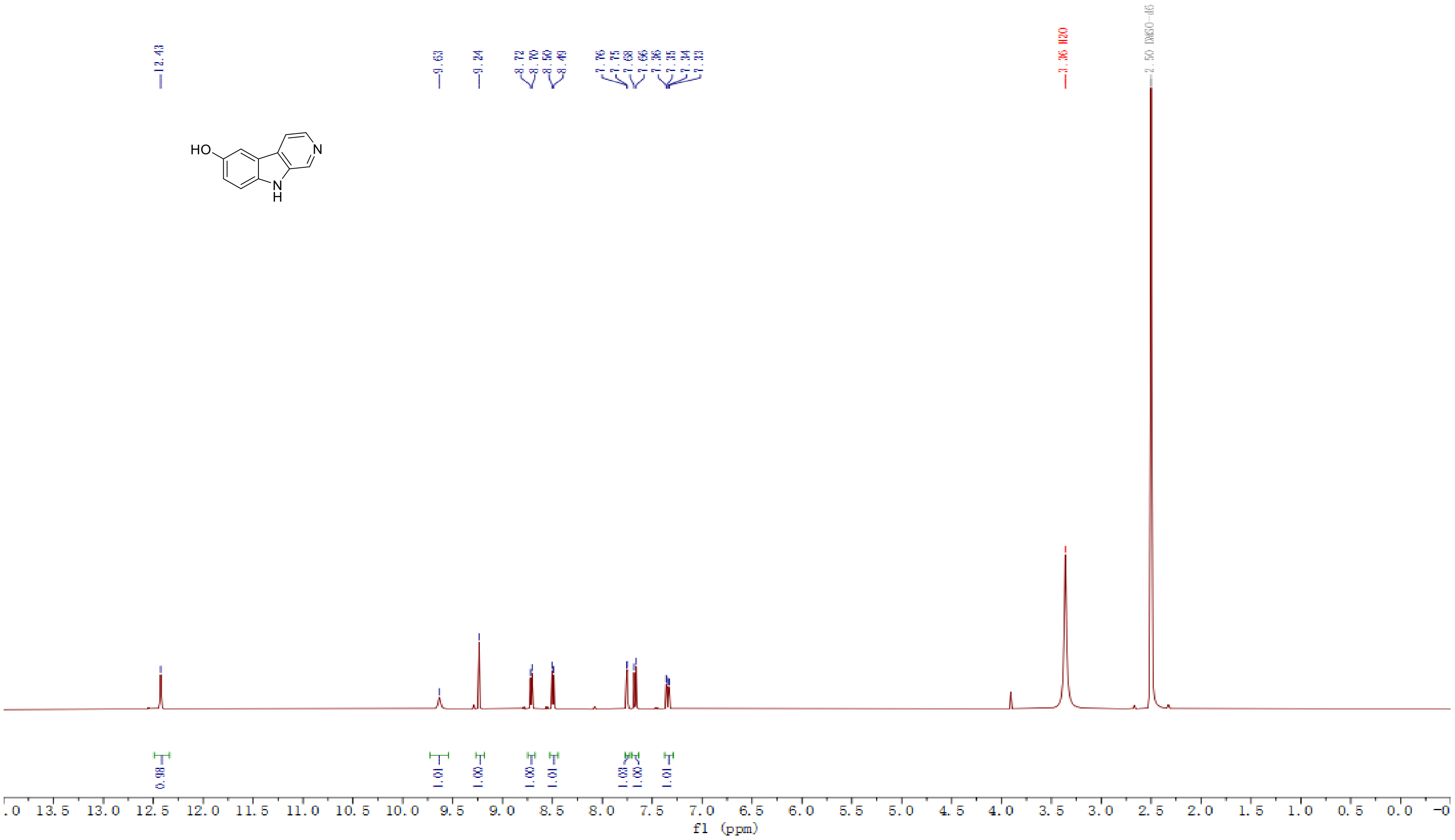

<sup></sup>

$^{1}$H NMR (400 MHz, DMSO-$d_6$) 9*H*-pyrido[3,4-*b*]indol-6-yl acetate (**1h**)

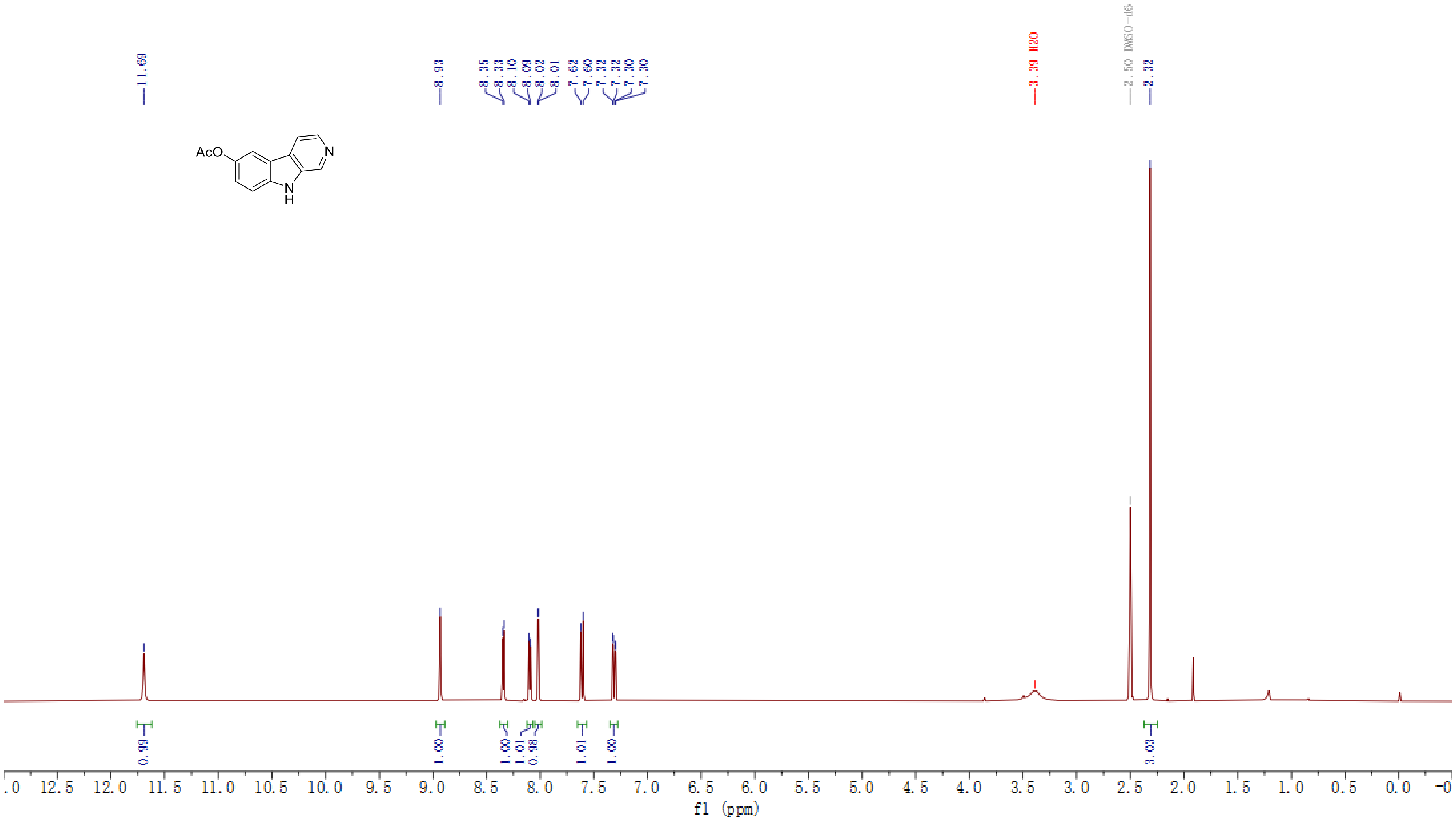

$^{13}$C NMR (400 MHz, DMSO-$d_6$) 9*H*-pyrido[3,4-*b*]indol-6-yl acetate (**1h**)

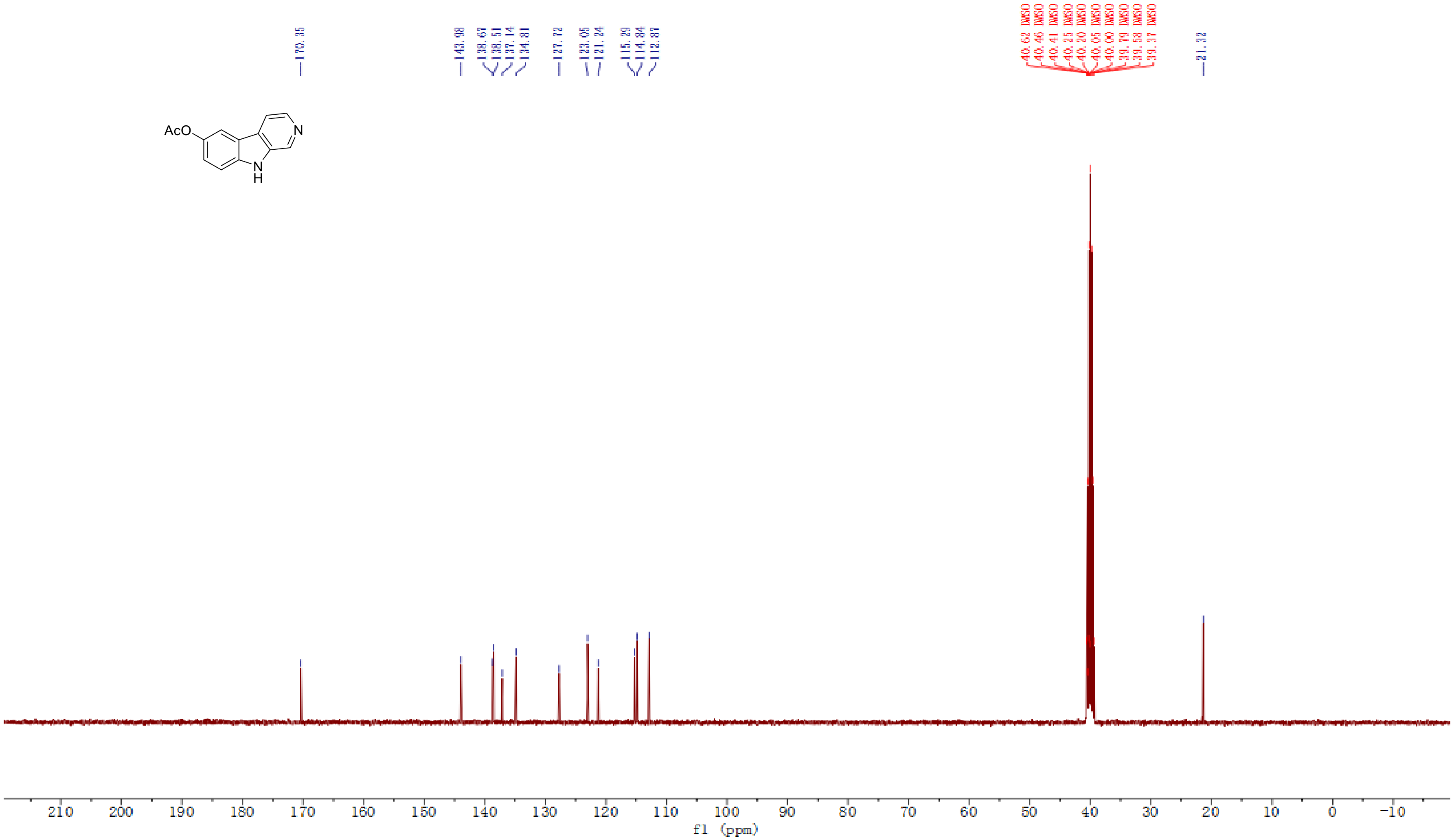

[1]H NMR (400 MHz, DMSO-$d_6$) 6-phenyl-9*H*-pyrido[3,4-*b*]indole (**1i**)

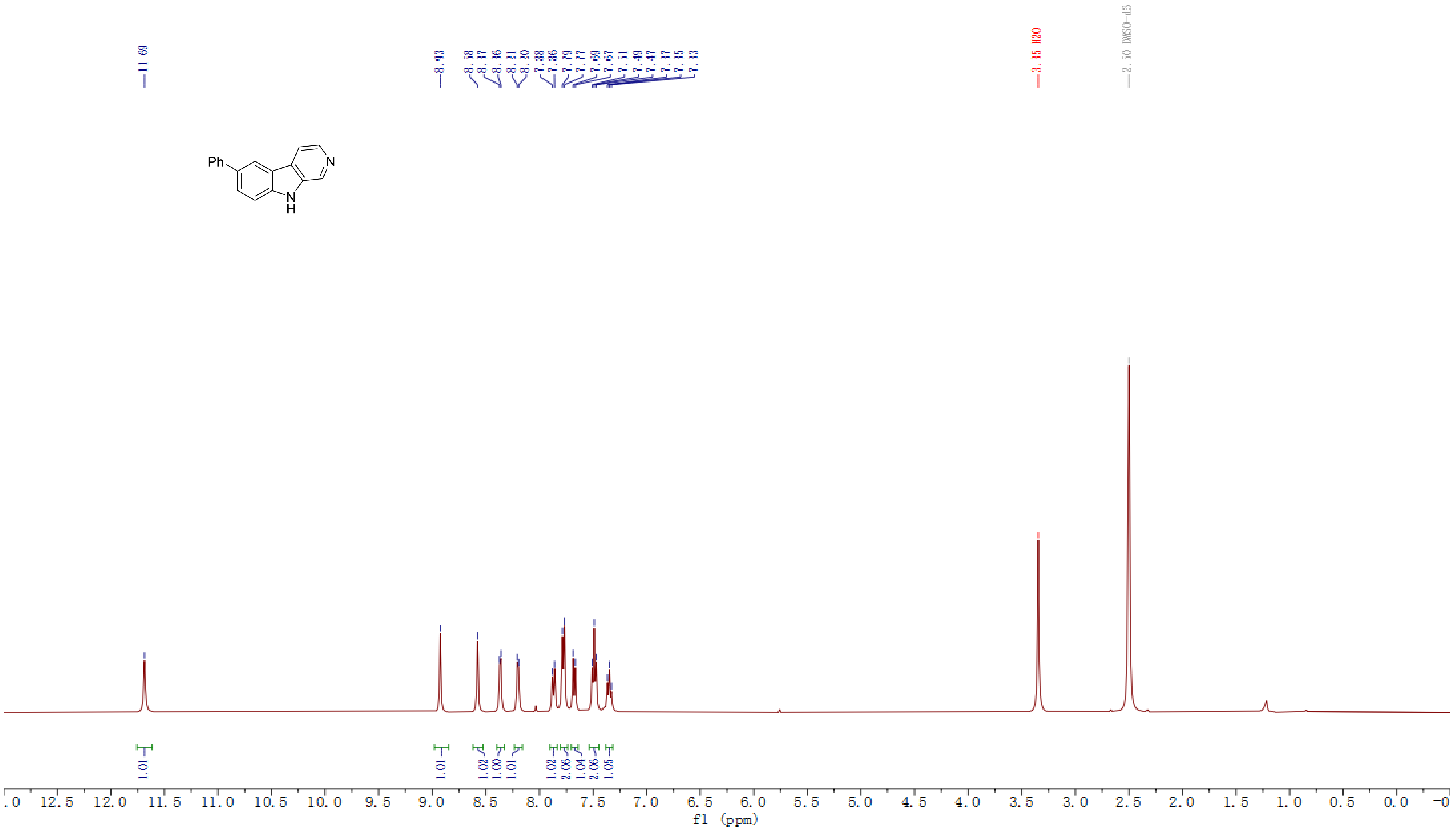

$^{1}$H NMR (400 MHz, DMSO-$d_6$) 6-fluoro-9*H*-pyrido[3,4-*b*]indole (**1j**)

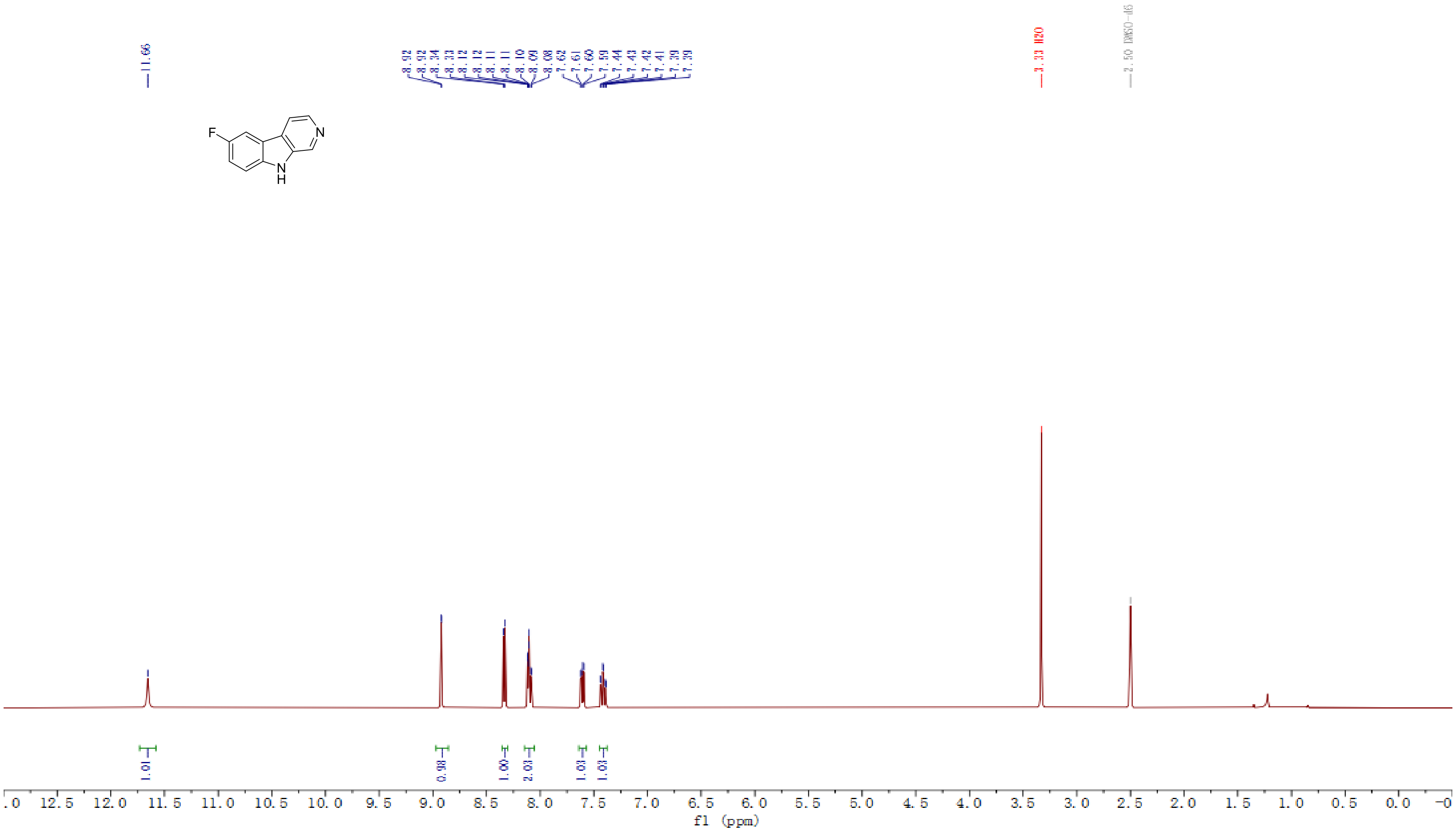

$^1$H NMR (400 MHz, DMSO-$d_6$) 6-chloro-9*H*-pyrido[3,4-*b*]indole (**1k)**

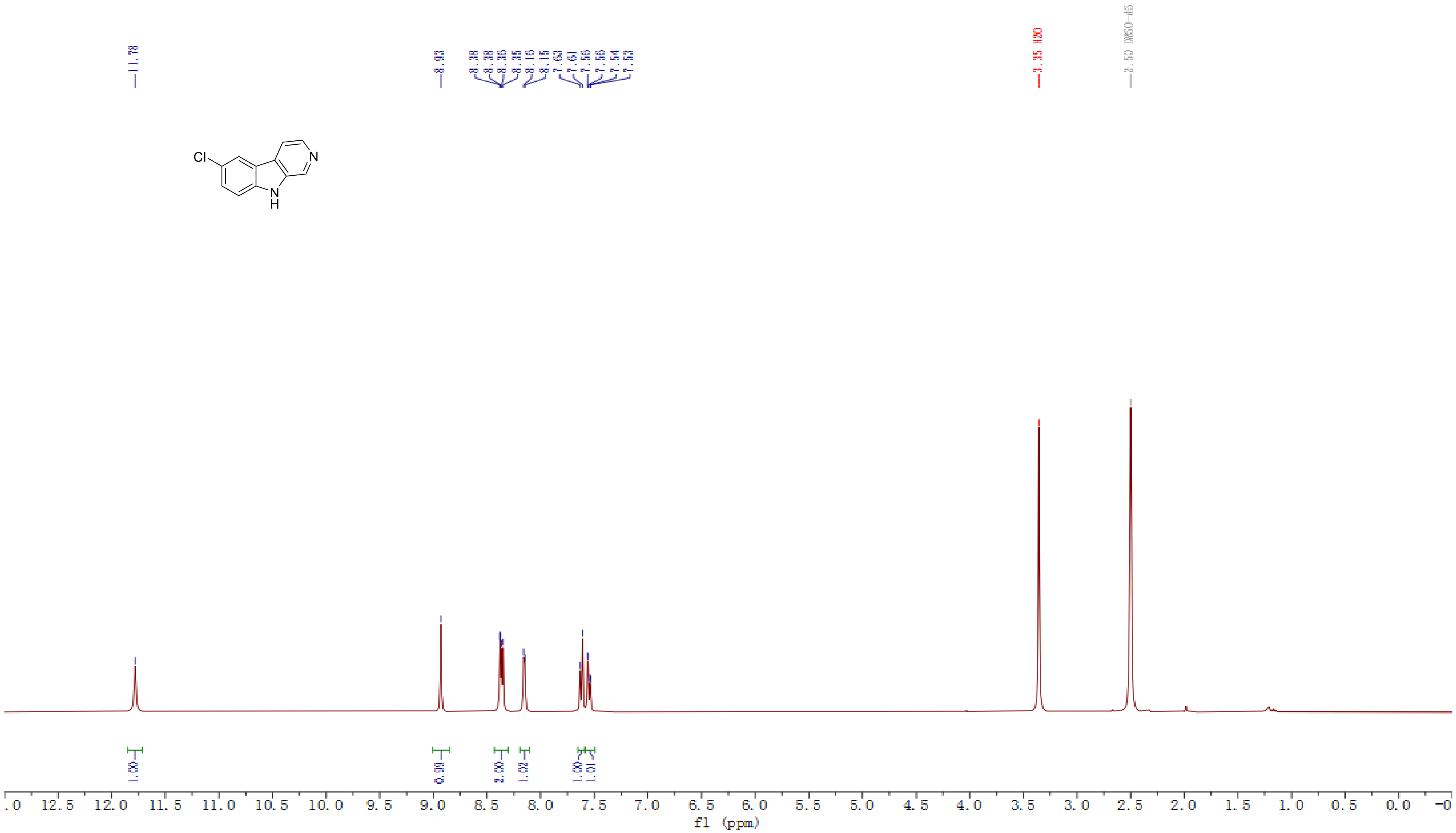

[1]H NMR (400 MHz, DMSO-$d_6$) 6-bromo-9*H*-pyrido[3,4-*b*]indole (**1l**)

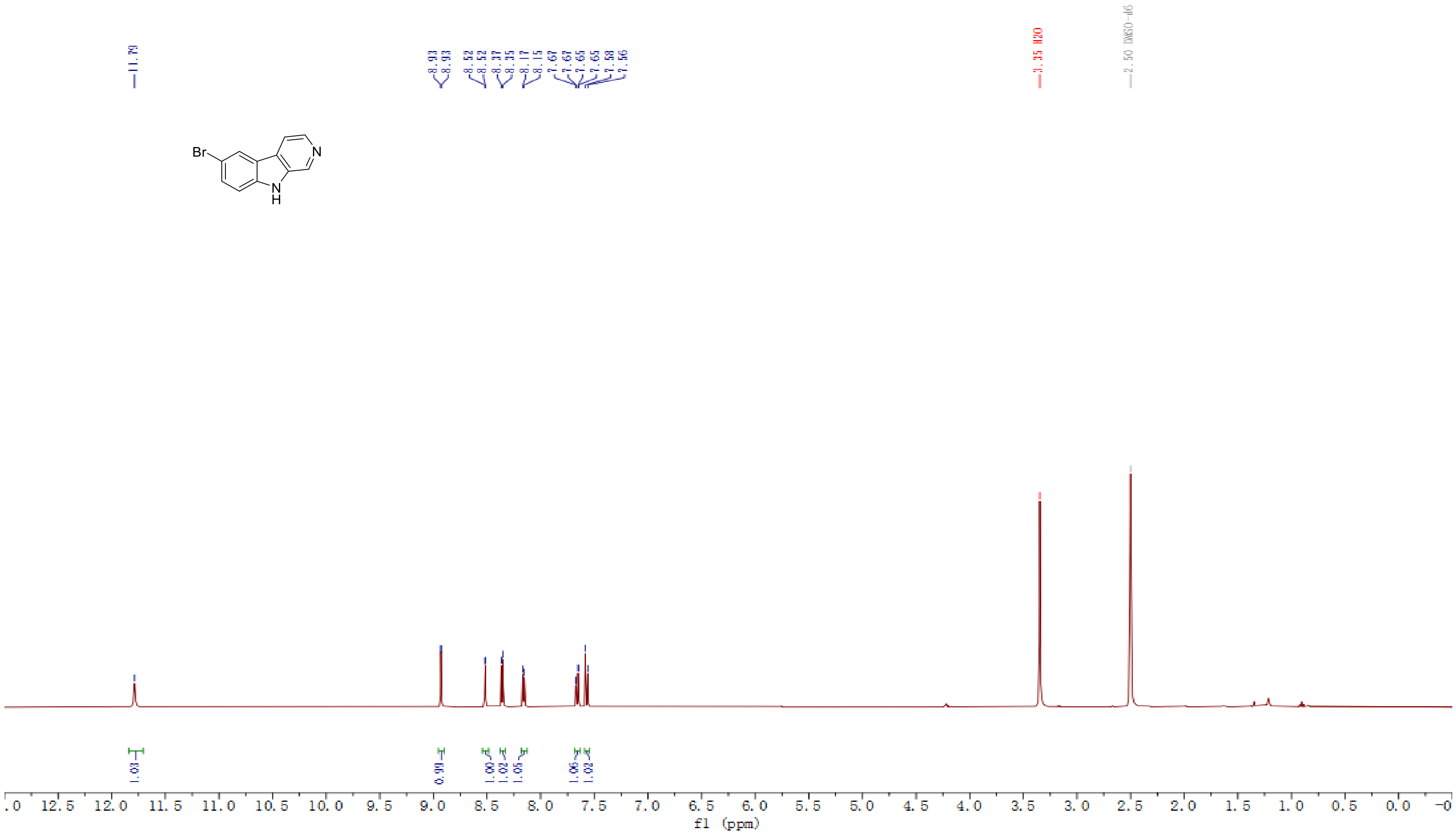

<sup></sup>

[1]H NMR (400 MHz, DMSO-$d_6$) 7-bromo-9*H*-pyrido[3,4-*b*]indole (**1m**)

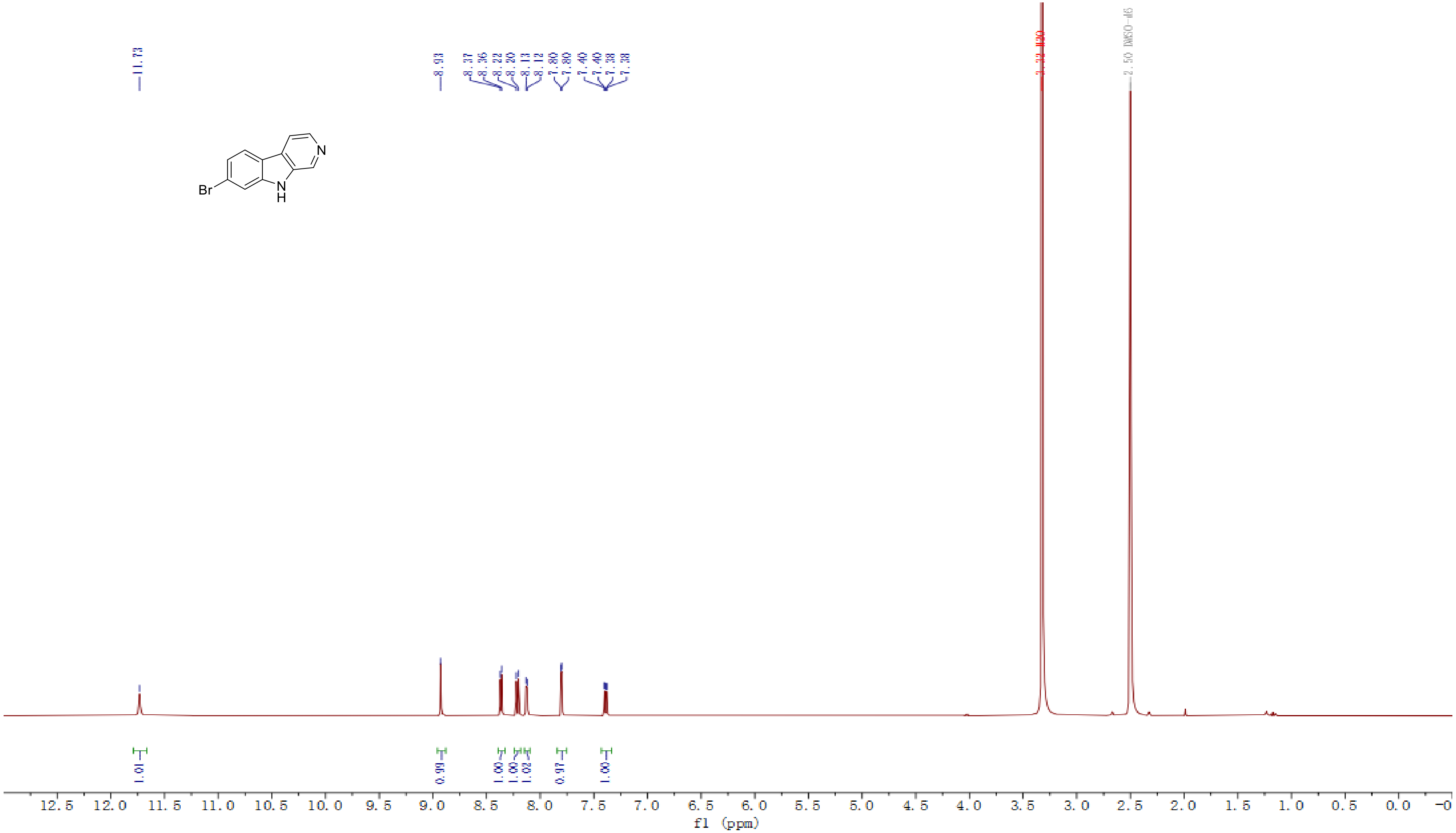

$^1$H NMR (400 MHz, DMSO-$d_6$) 8-bromo-9*H*-pyrido[3,4-*b*]indole (**1n**)

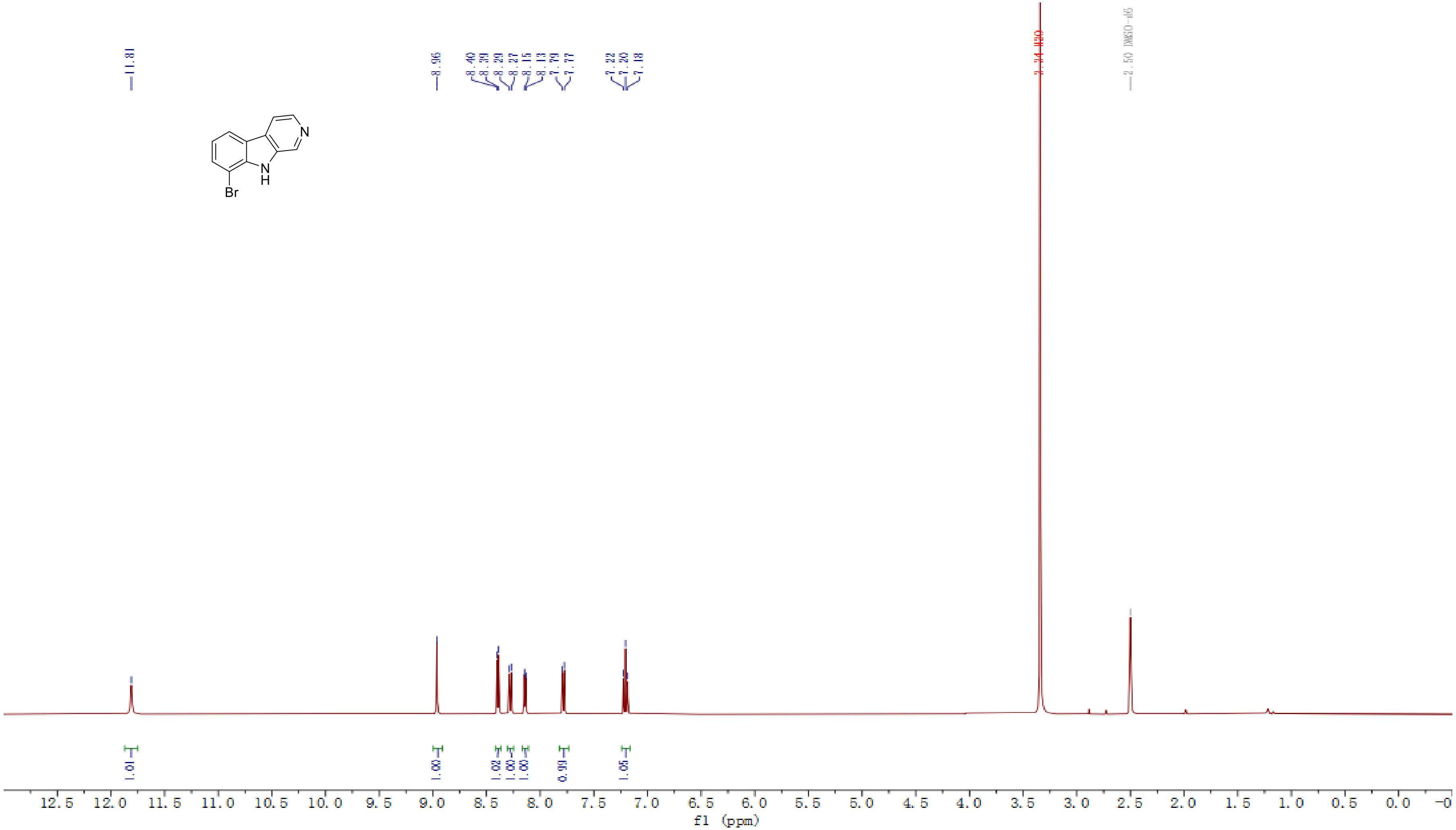

$^{1}H$ NMR (400 MHz, DMSO-$d_6$) 9-methyl-9H-pyrido[3,4-b]indole (**1o**)

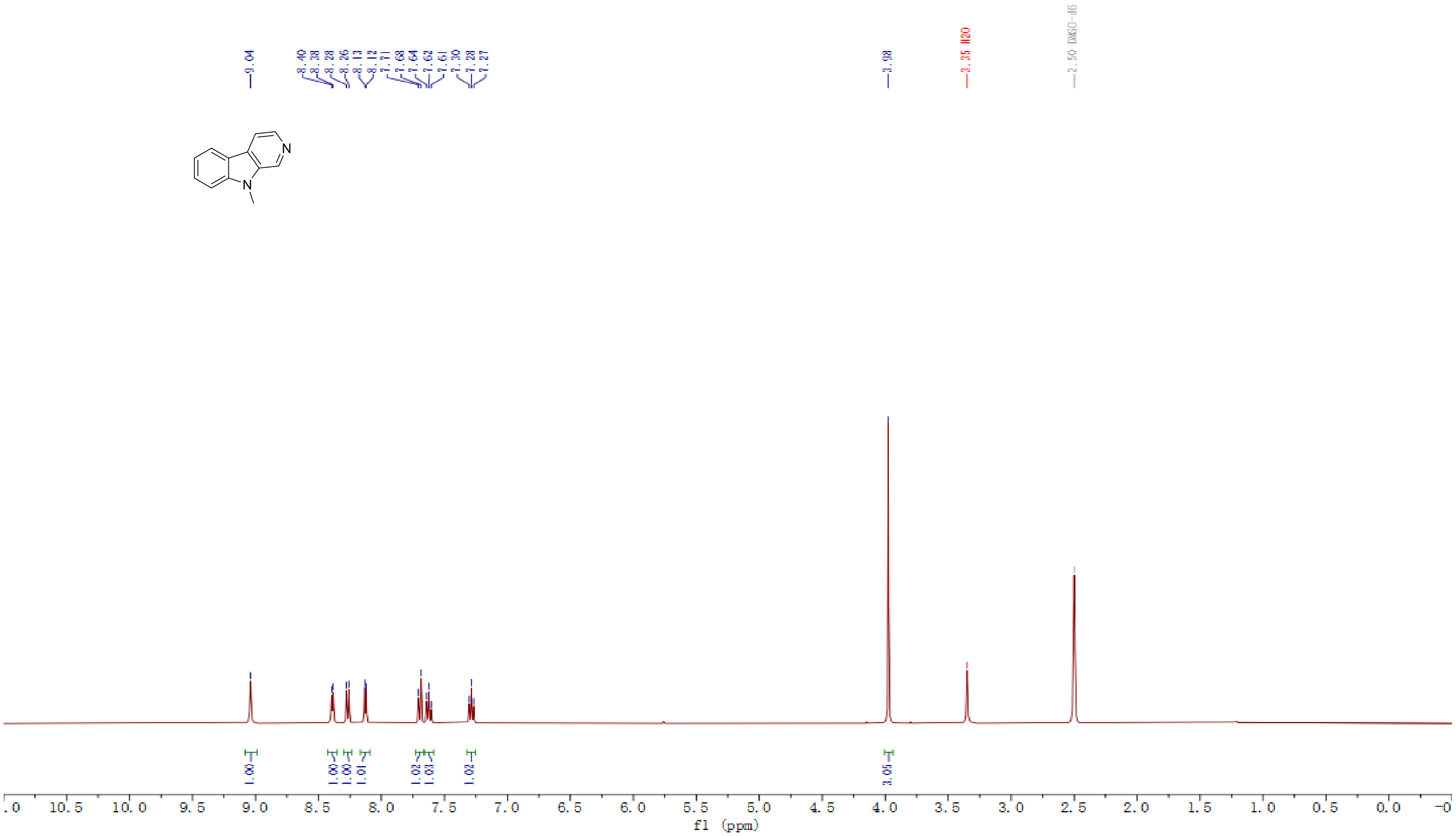

$^{1}H$ NMR (400 MHz, DMSO-$d_6$) 1-deuterated β-carboline

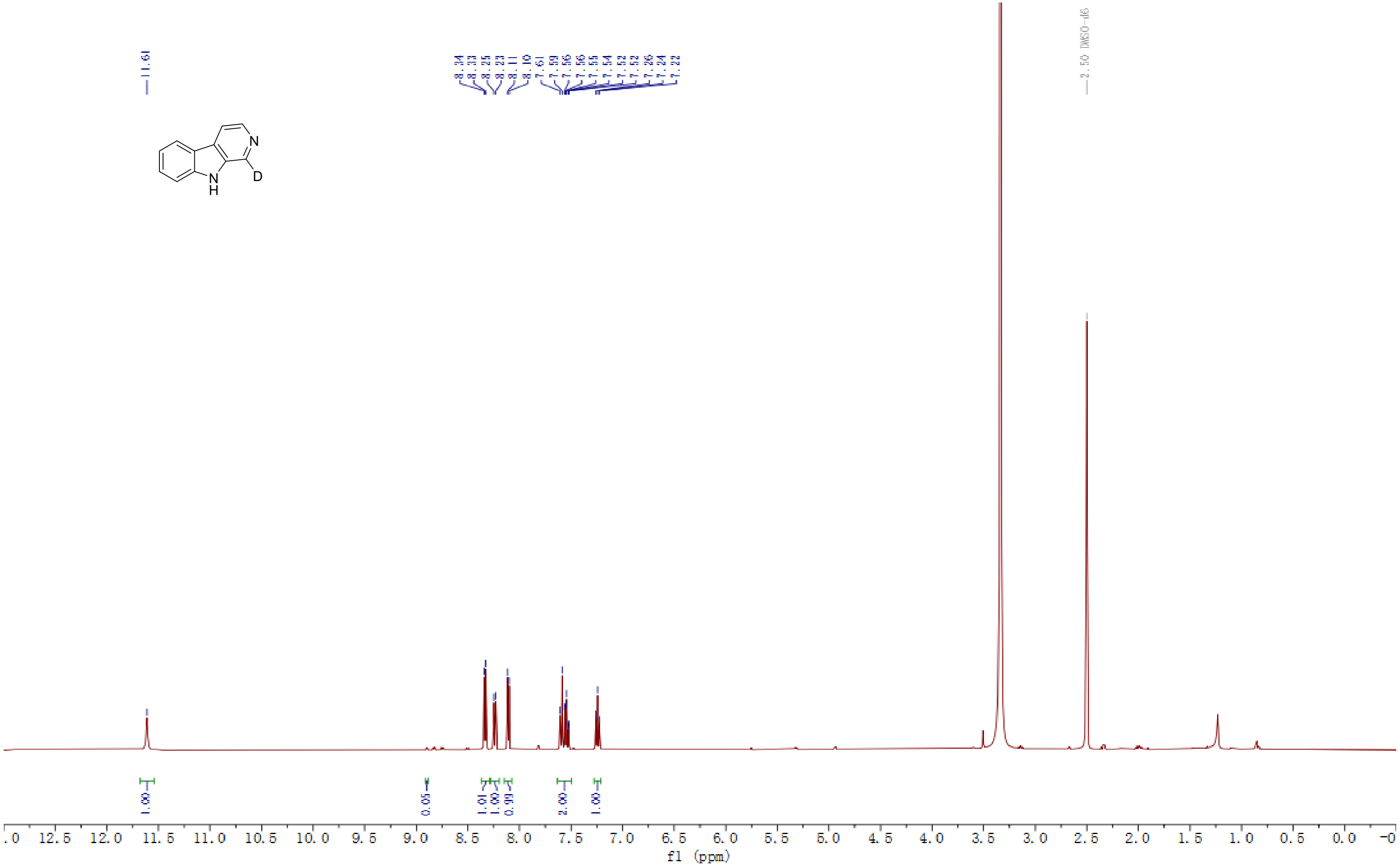

[1]H NMR (400 MHz, $CDCl_3$) *N*-(prop-1-en-1-yl)acetamide (**2f**) (*E*:*Z*=10:1)

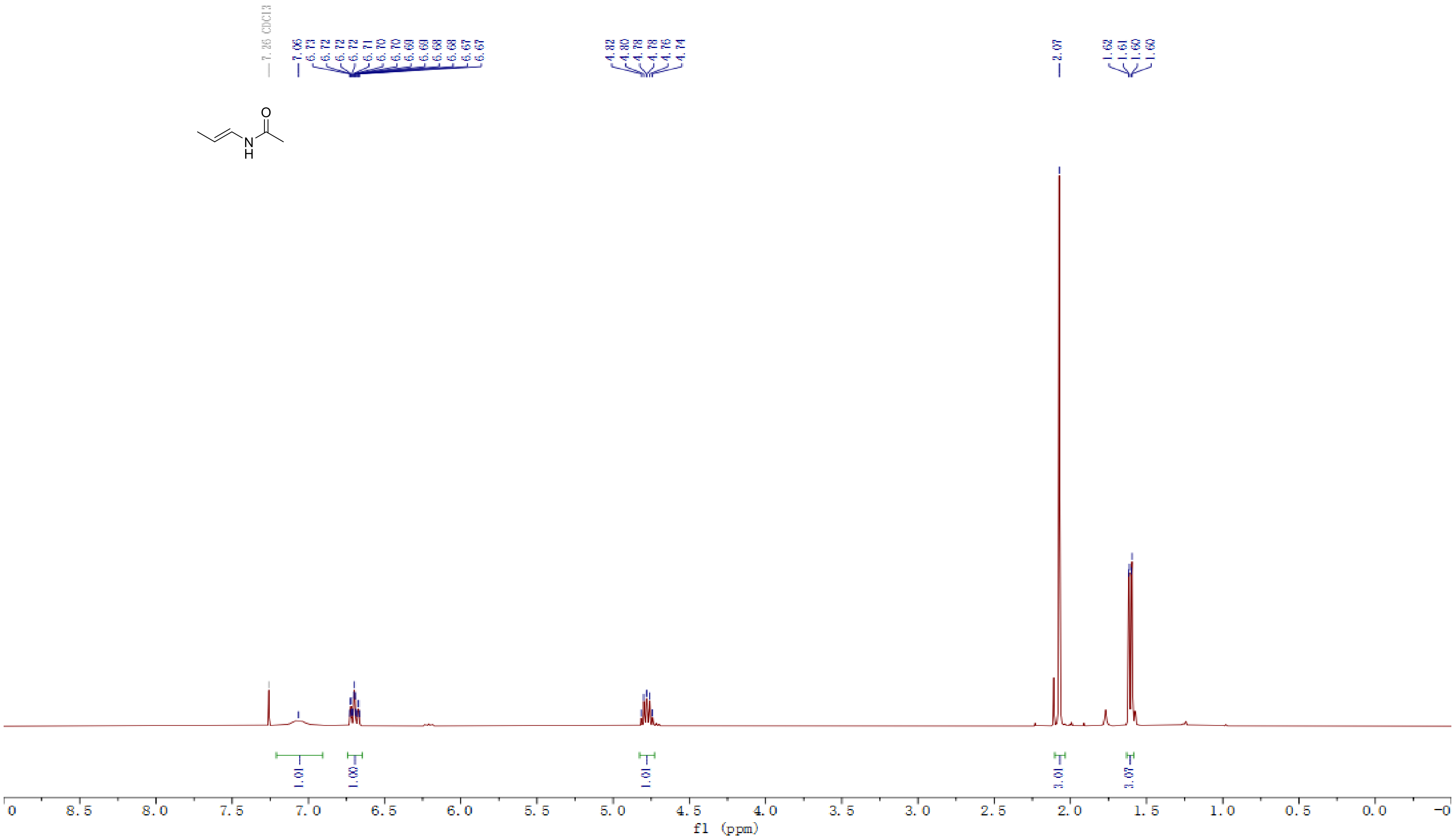

[1]H NMR (400 MHz, $CDCl_3$) pentyl 2-diazoacetate (**3b**)

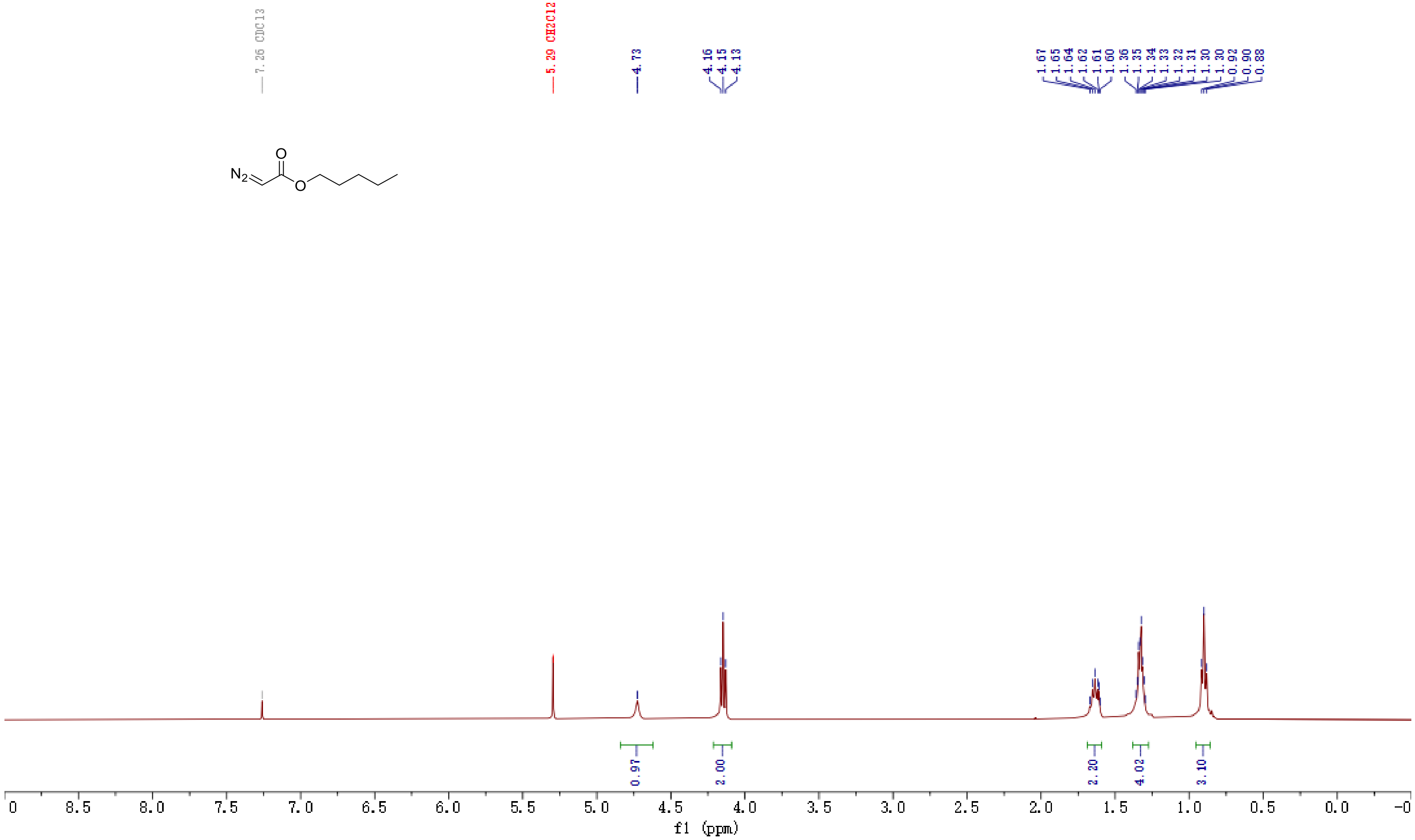

[1]H NMR (400 MHz, $CDCl_3$) isopropyl 2-diazoacetate (**3c**)

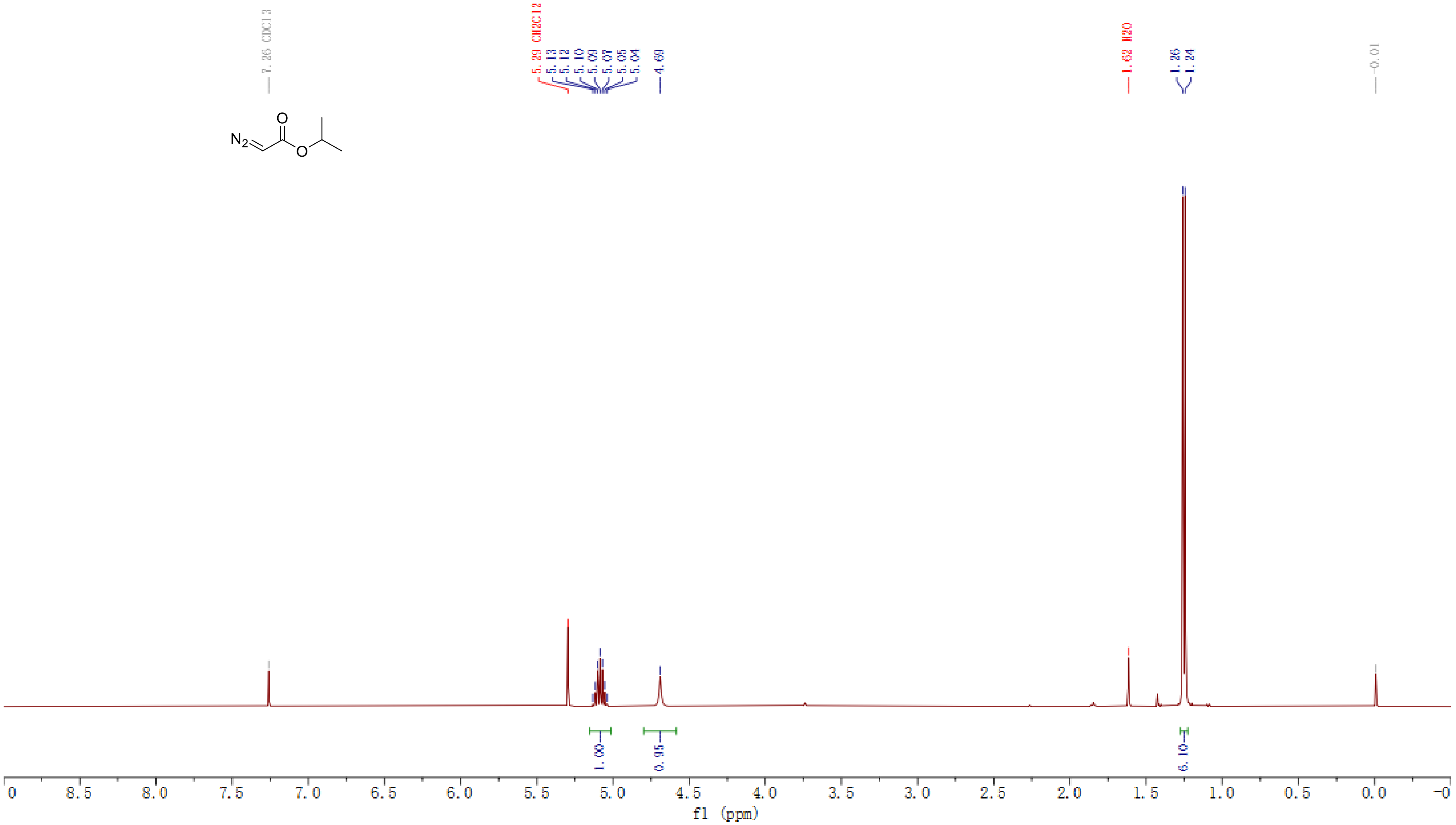

$^{1}H$ NMR (400 MHz, DMSO-$d_6$) pent-4-en-1-yl 2-diazoacetate (**3e**)

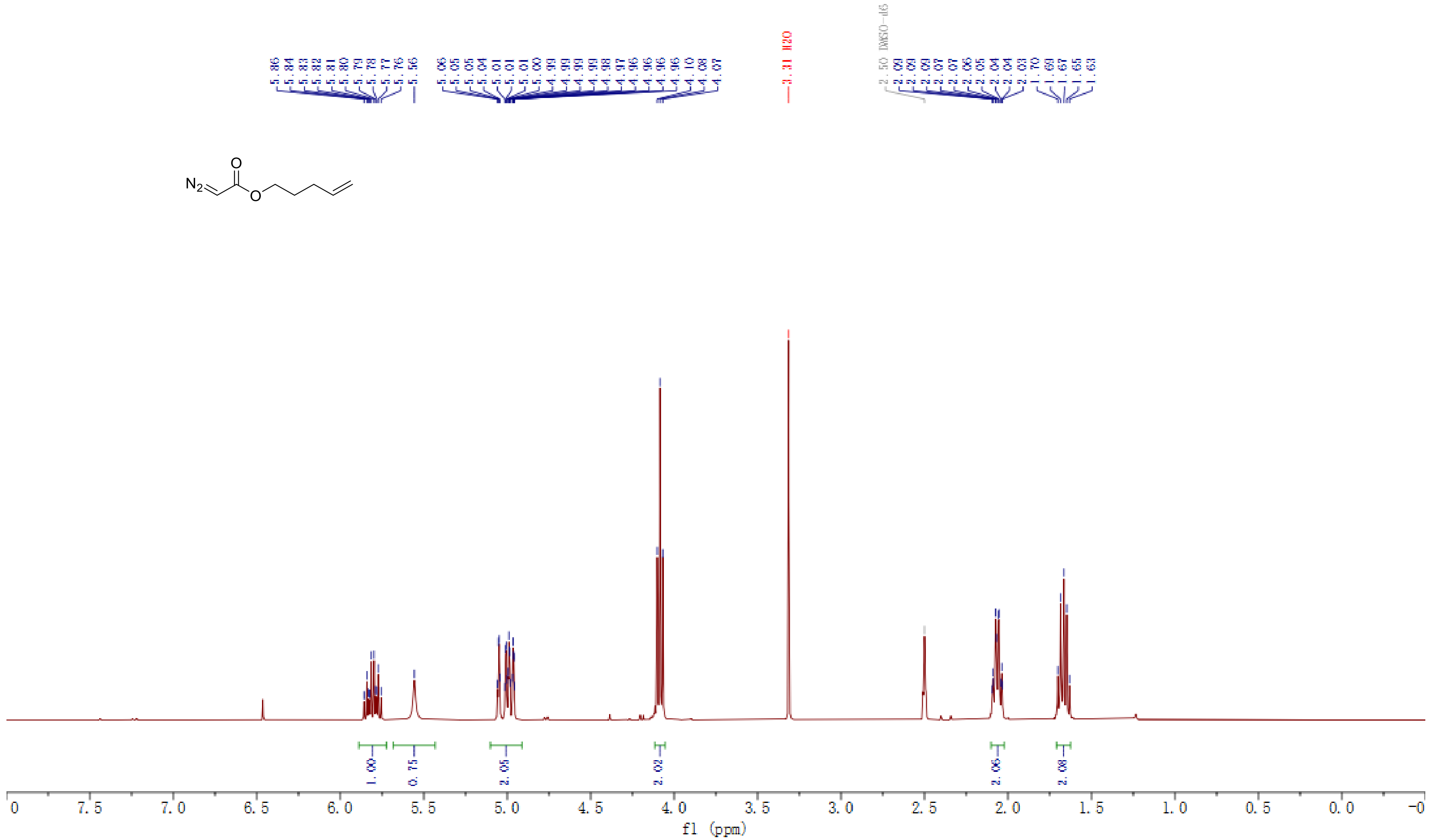

[1]H NMR (400 MHz, DMSO-$d_6$) pent-4-yn-1-yl 2-diazoacetate (**3f**)

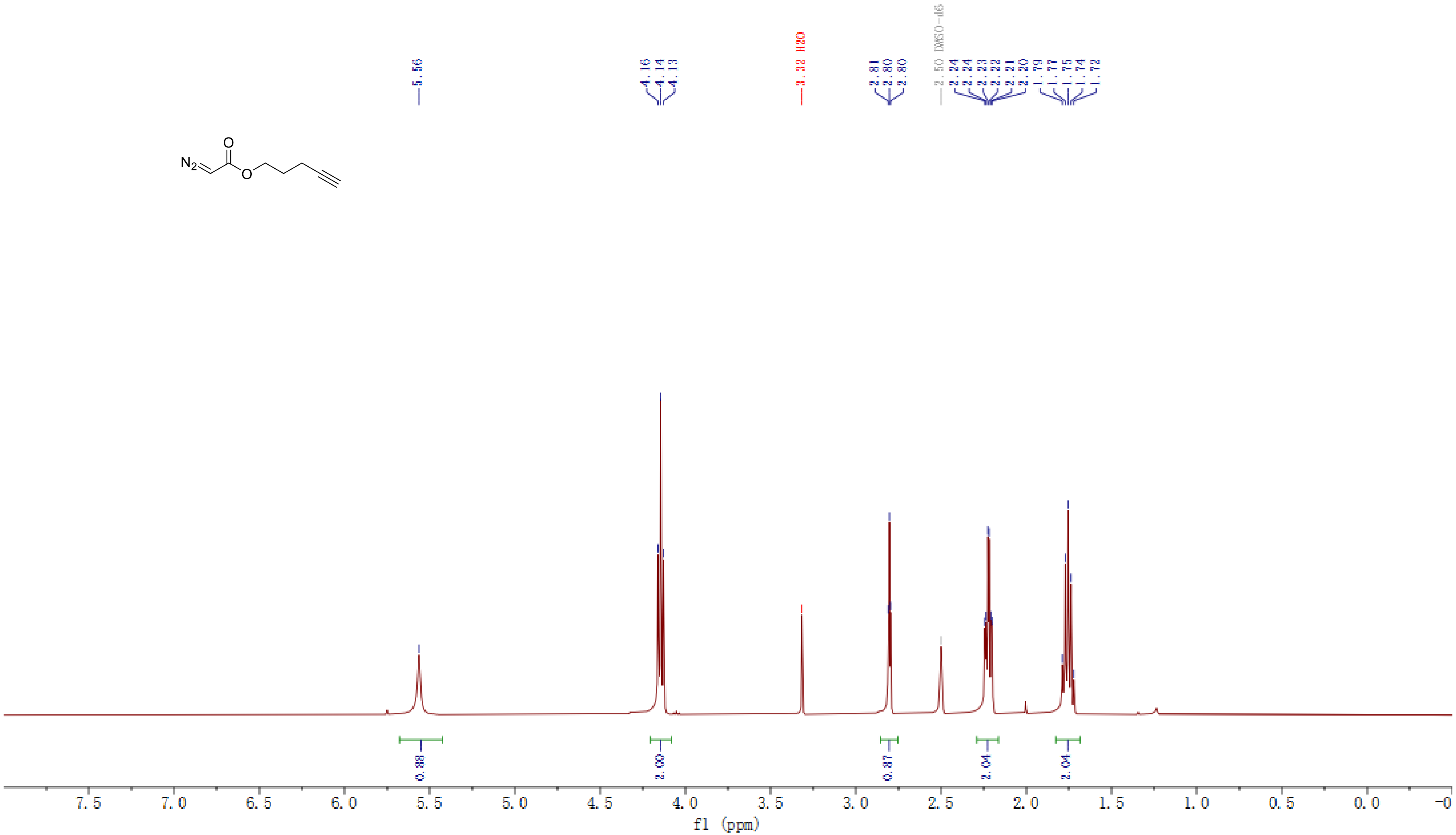

<sup></sup>

[1]H NMR (400 MHz, $CDCl_3$) phenyl 2-diazoacetate (**3g**)

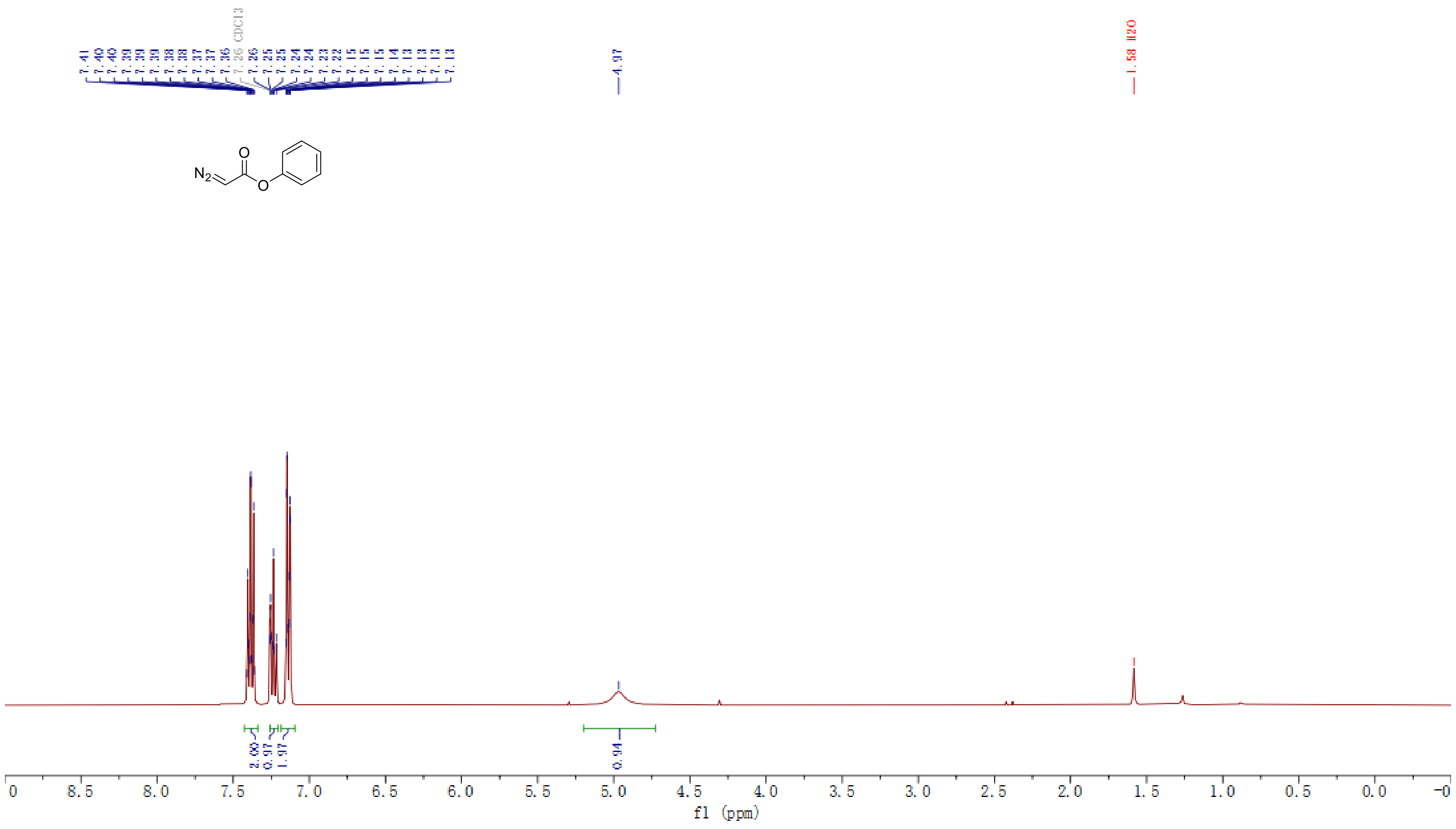

[1]H NMR (400 MHz, $CDCl_3$) 4-methoxyphenyl 2-diazoacetate **(3h)**

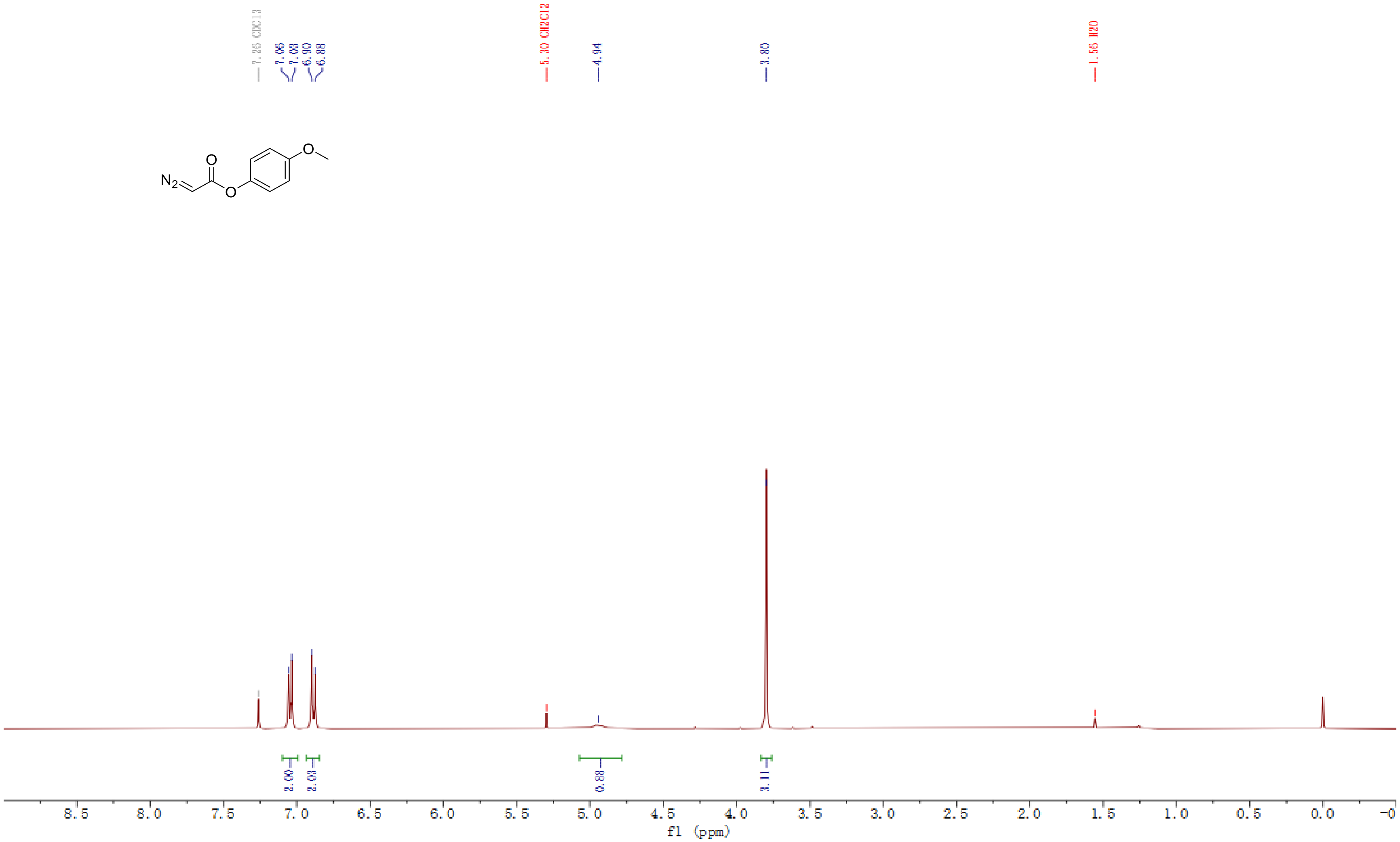

$^{1}H$ NMR (400 MHz, $CDCl_3$) 4-fluorophenyl 2-diazoacetate (**3i**)

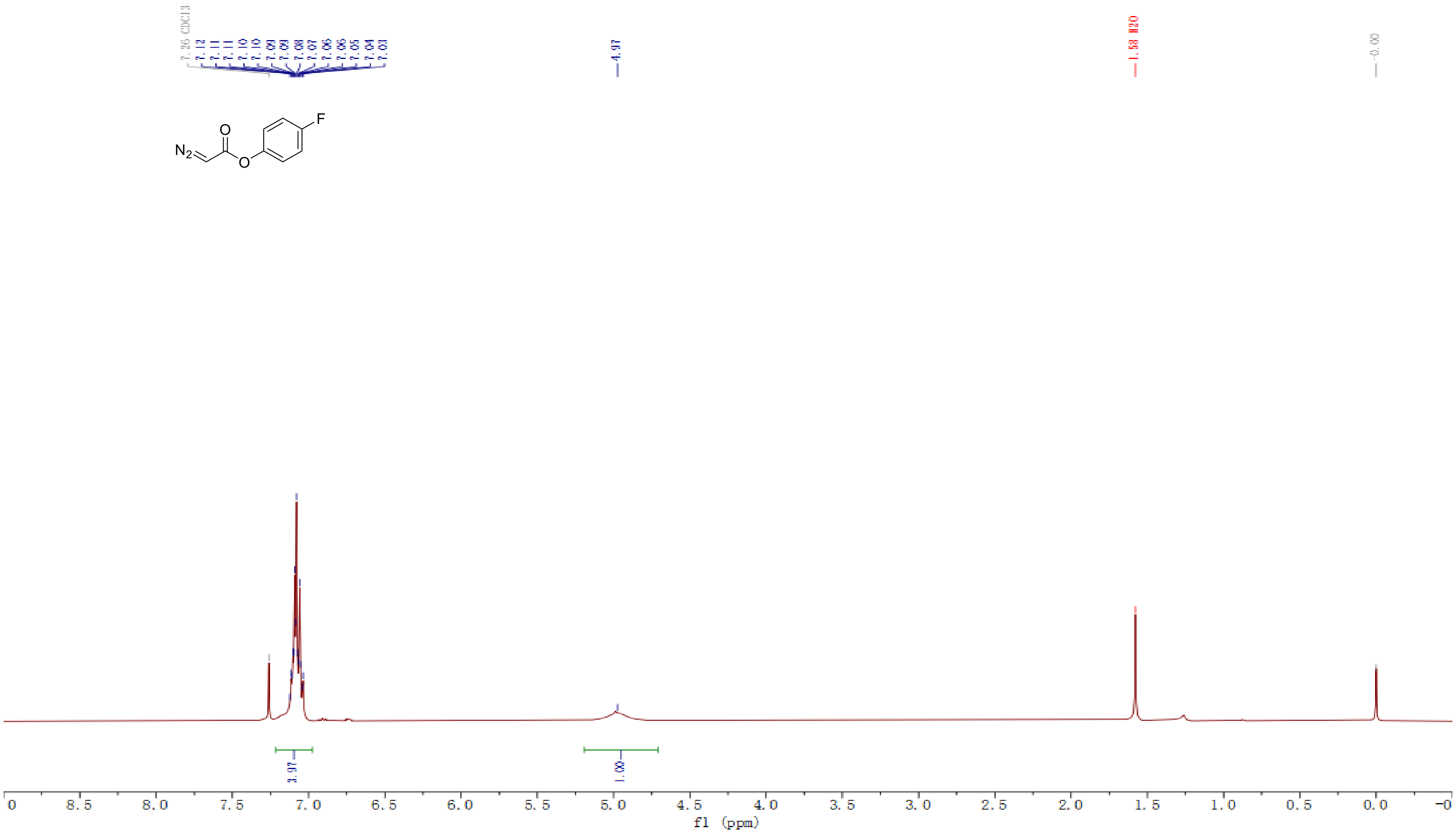

$^{1}H$ NMR (400 MHz, $CDCl_3$) benzyl 2-diazoacetate (**3j**)

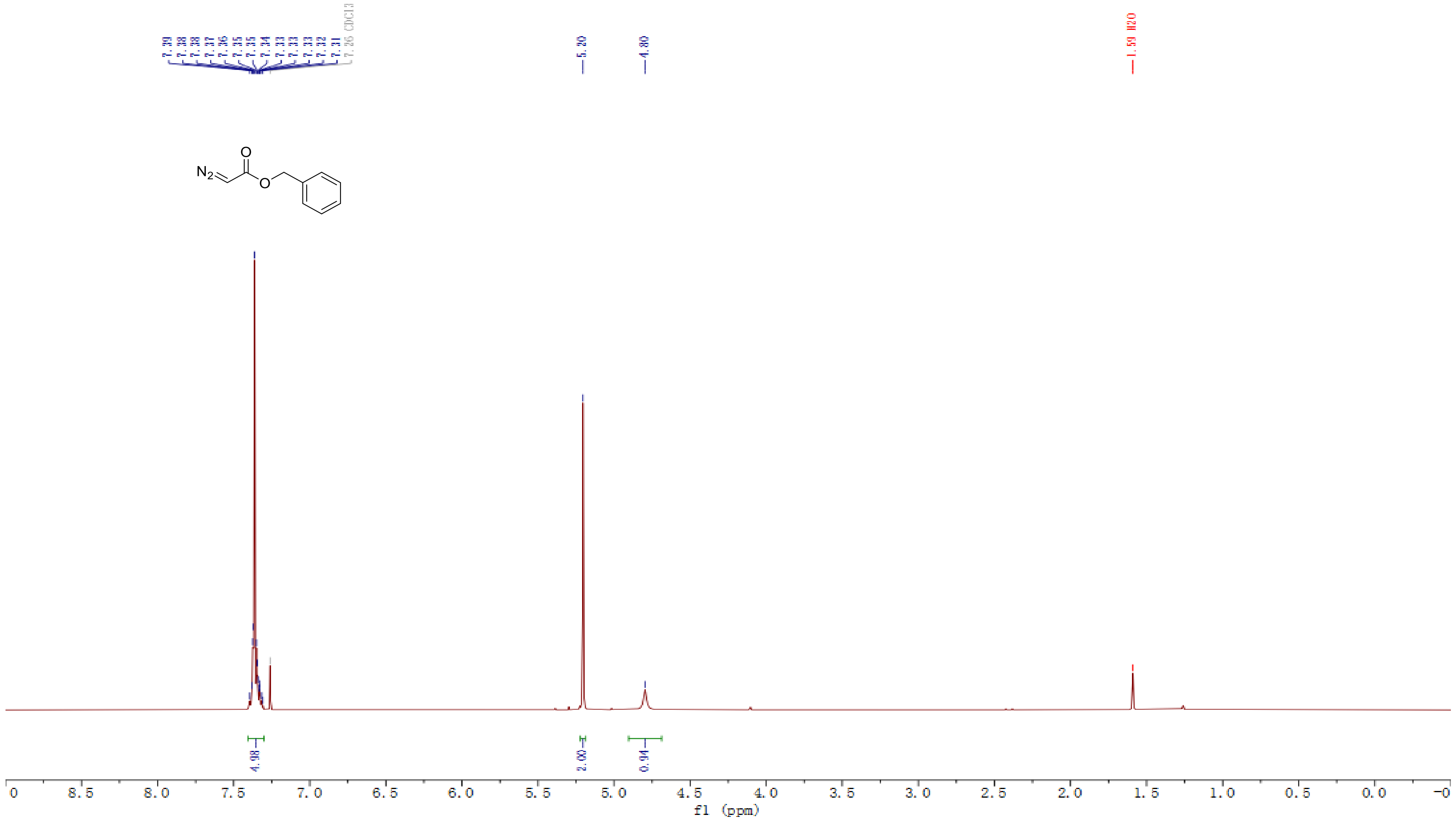

[1]H NMR (400 MHz, DMSO-$d_6$) 4-methoxybenzyl 2-diazoacetate (**3k**)

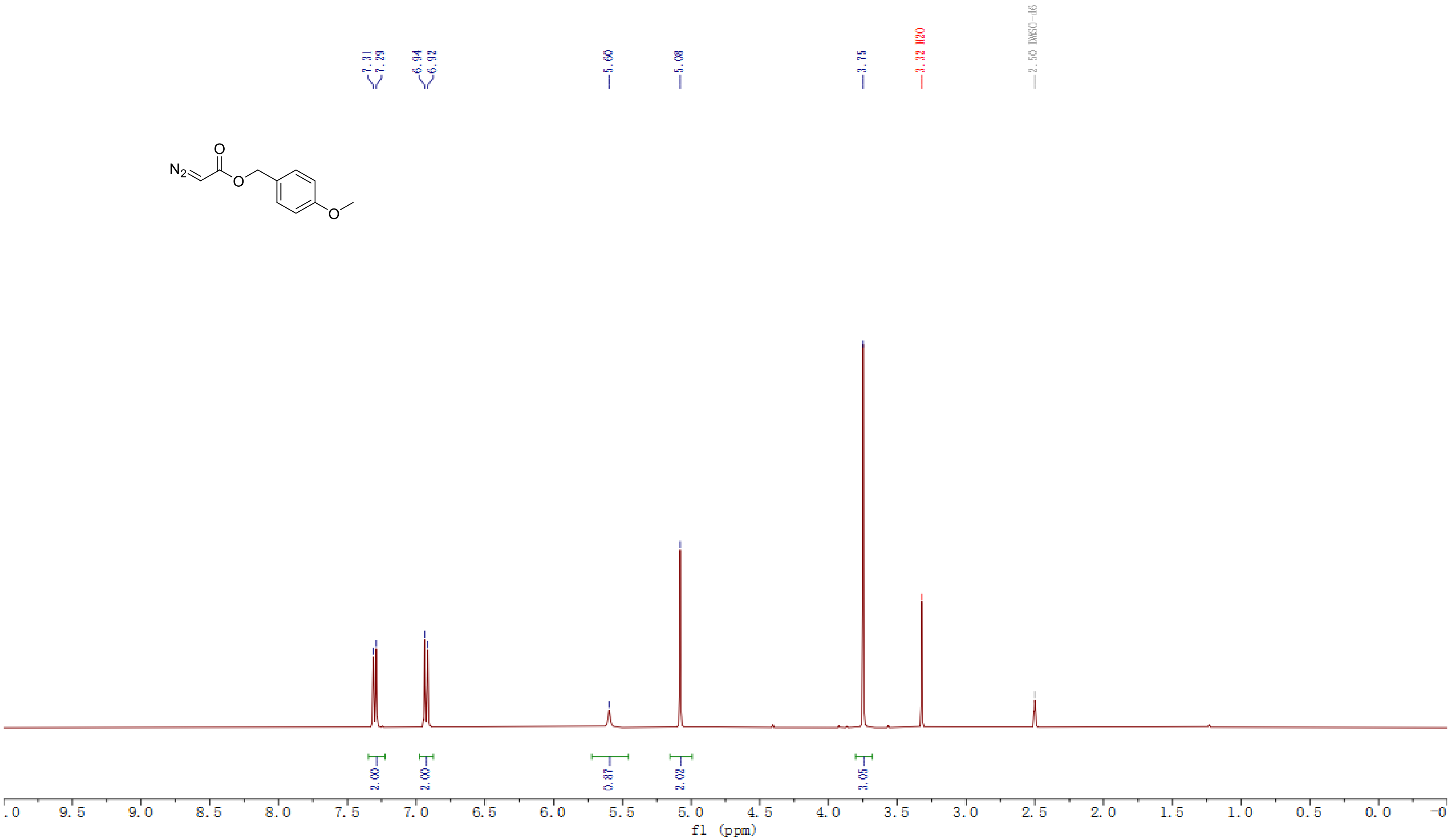

[1]H NMR (400 MHz, DMSO-$d_6$) 4-fluorobenzyl 2-diazoacetate (**3l**)

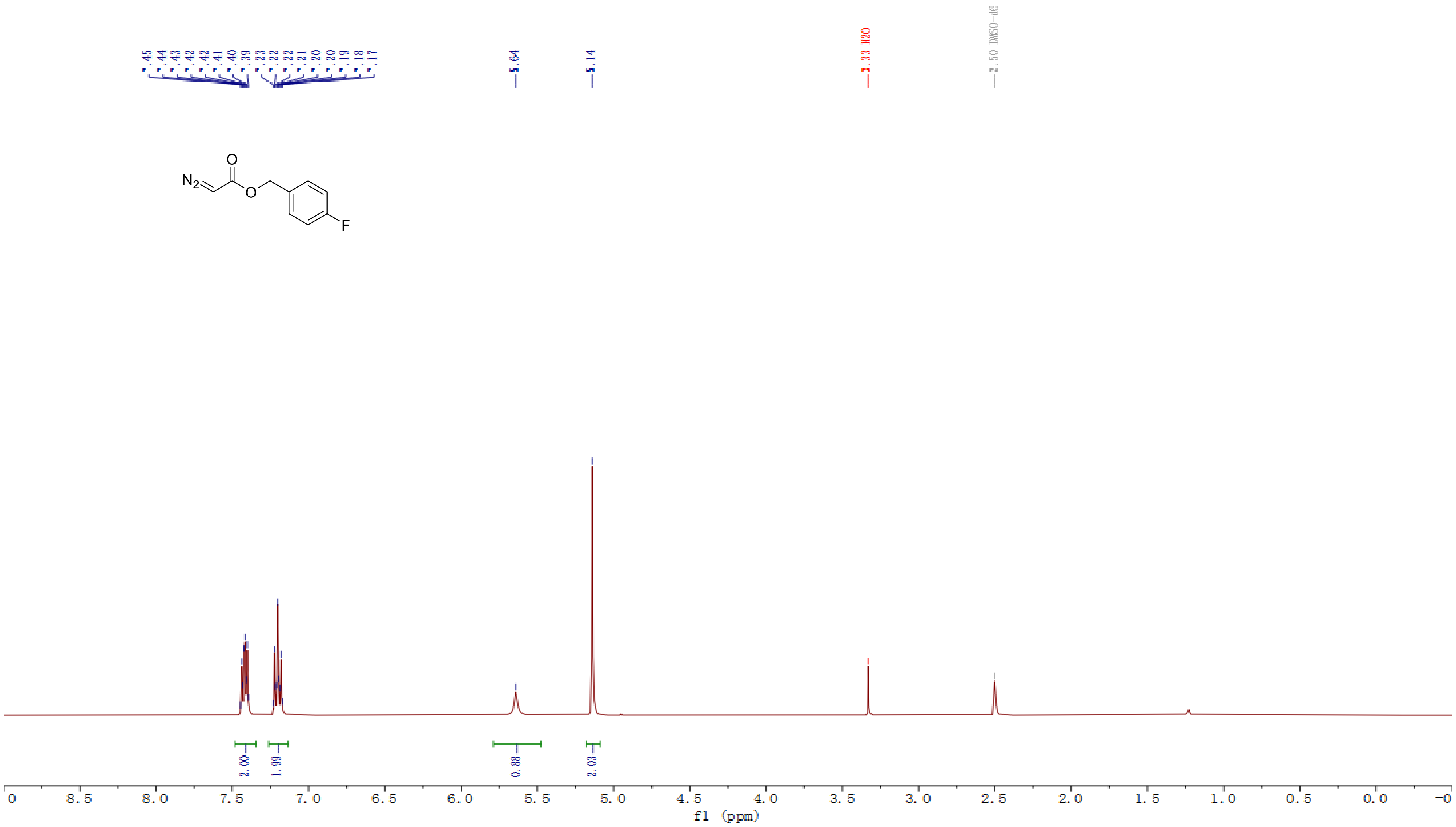

$^{1}H$ NMR (400 MHz, $CDCl_3$) 1-diazo-3,3-dimethylbutan-2-one (**3m**)

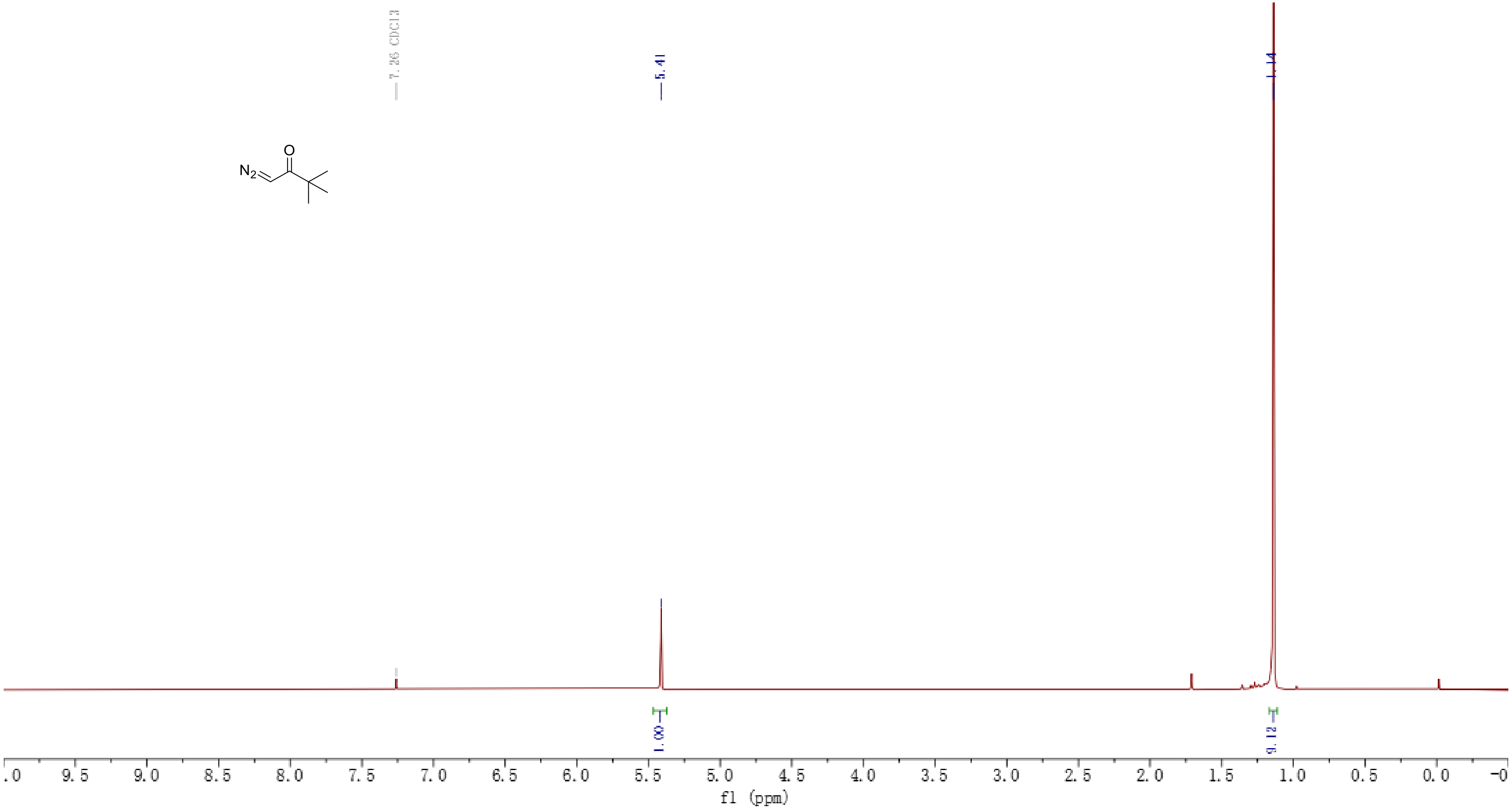

[1]H NMR (400 MHz, DMSO-$d_6$) 2-diazo-1-phenylethan-1-one (**3n**)

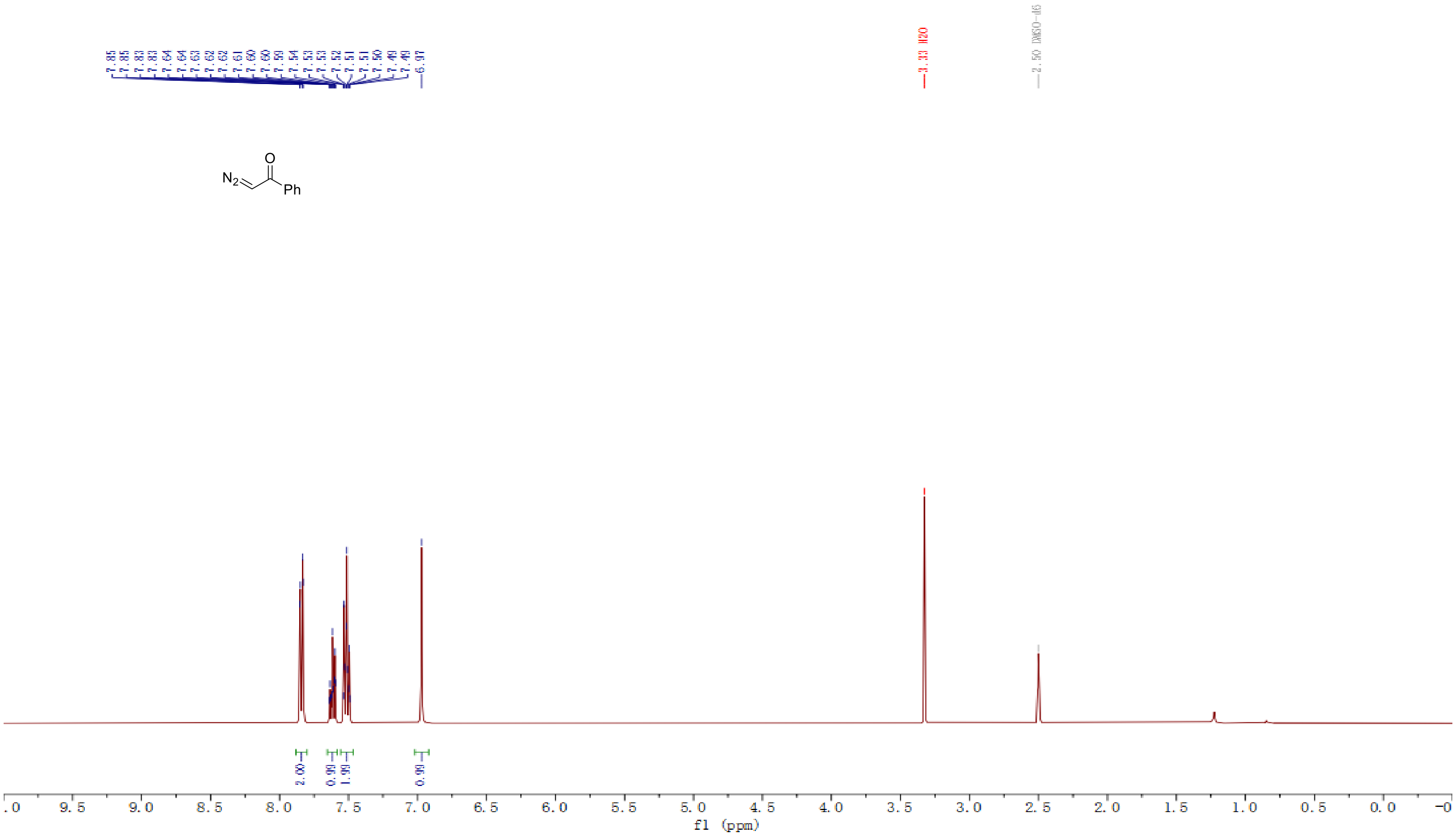

<sup></sup>

$^{1}$H NMR (400 MHz, $CDCl_3$) 2-diazo-*N,N*-dimethylacetamide (**3o**)

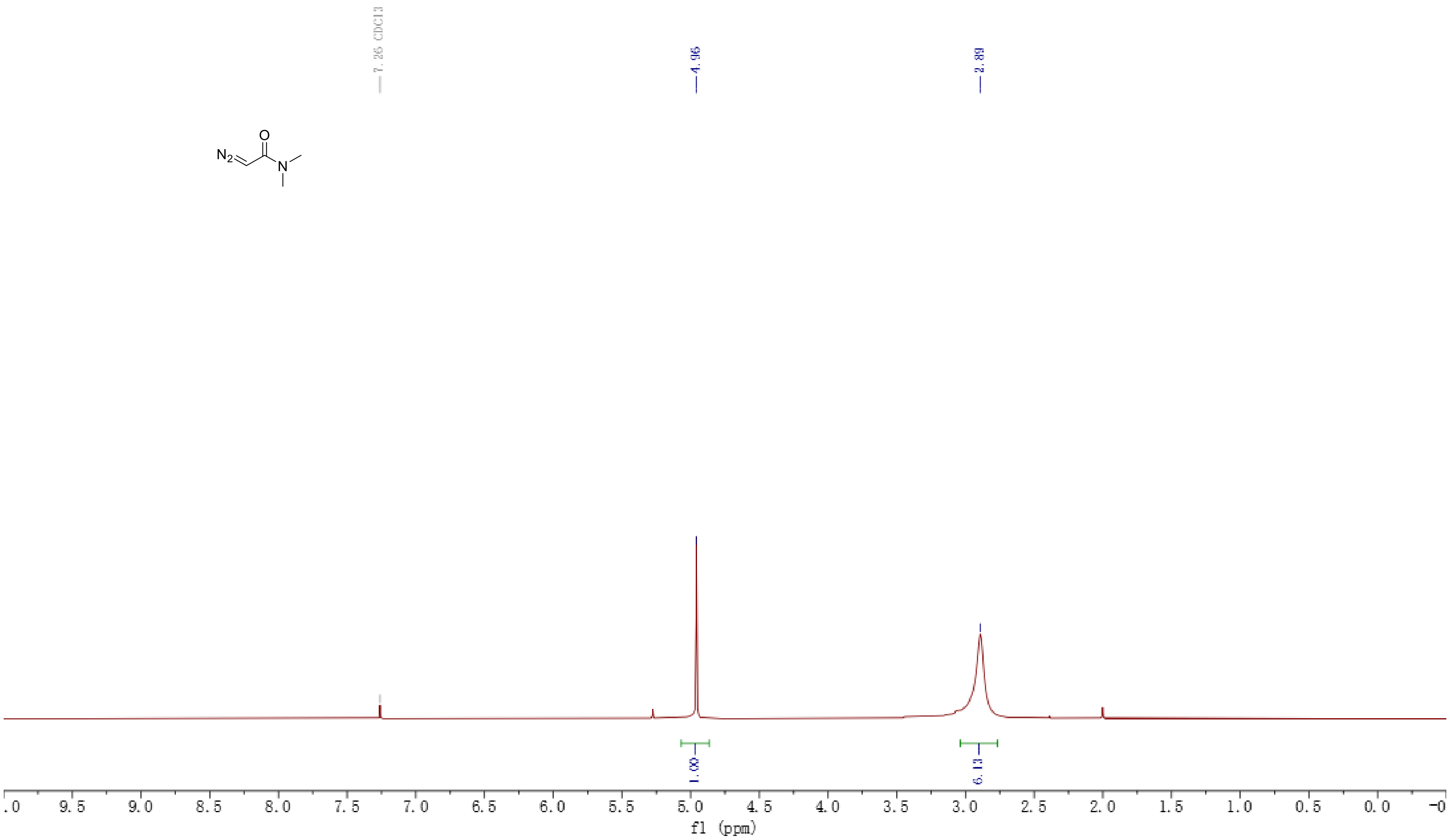

[1]H NMR (400 MHz, $CDCl_3$) (*R*)-ethyl-4-acetamido-4-(9*H*-pyrido[3,4-*b*]indol-1-yl)butanoate (**4a**)

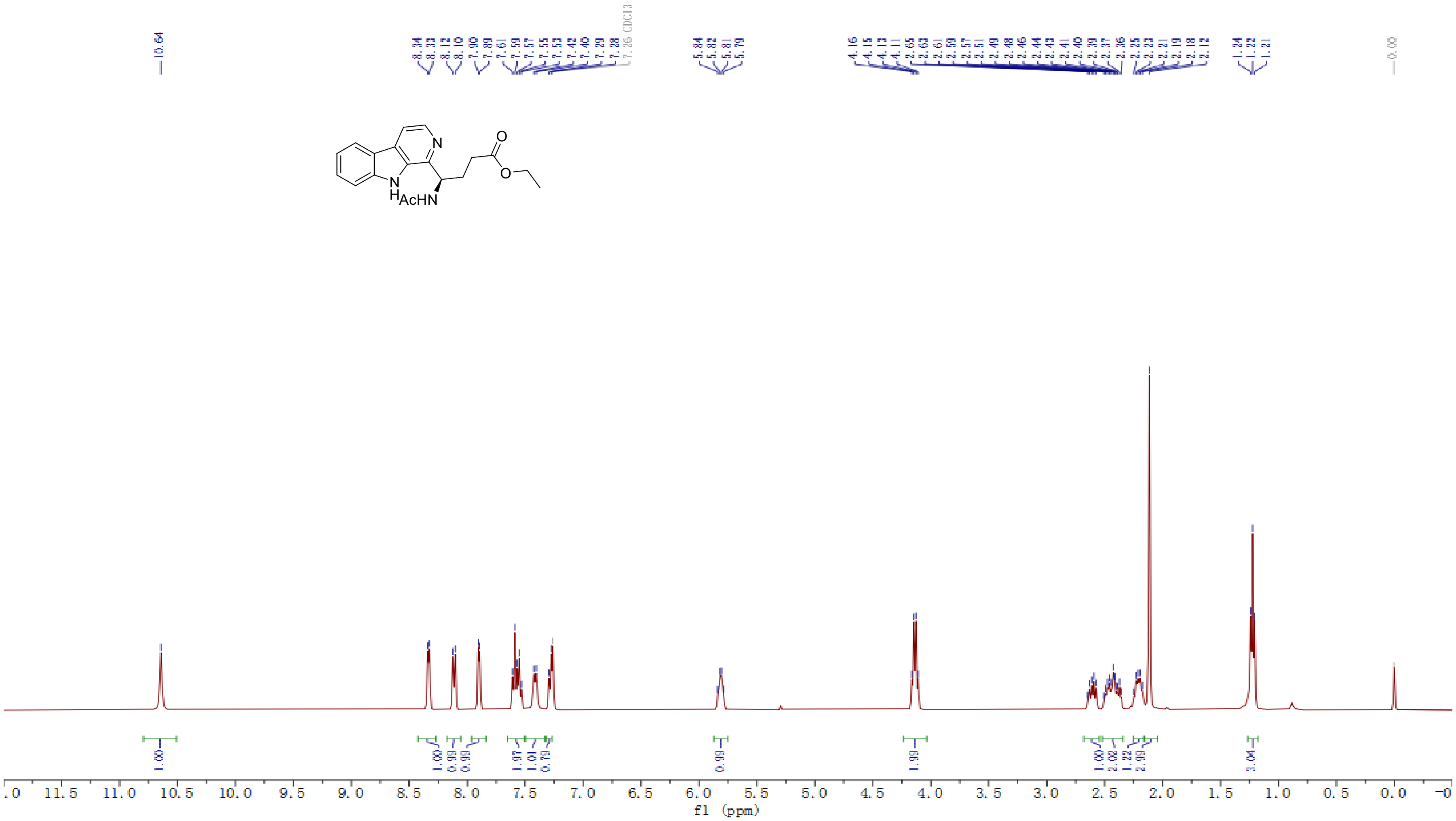

$^{13}C$ NMR (101 MHz, $CDCl_3$) (R)-ethyl -4-acetamido-4-(9H-pyrido[3,4-*b*]indol-1-yl)butanoate (**4a**)

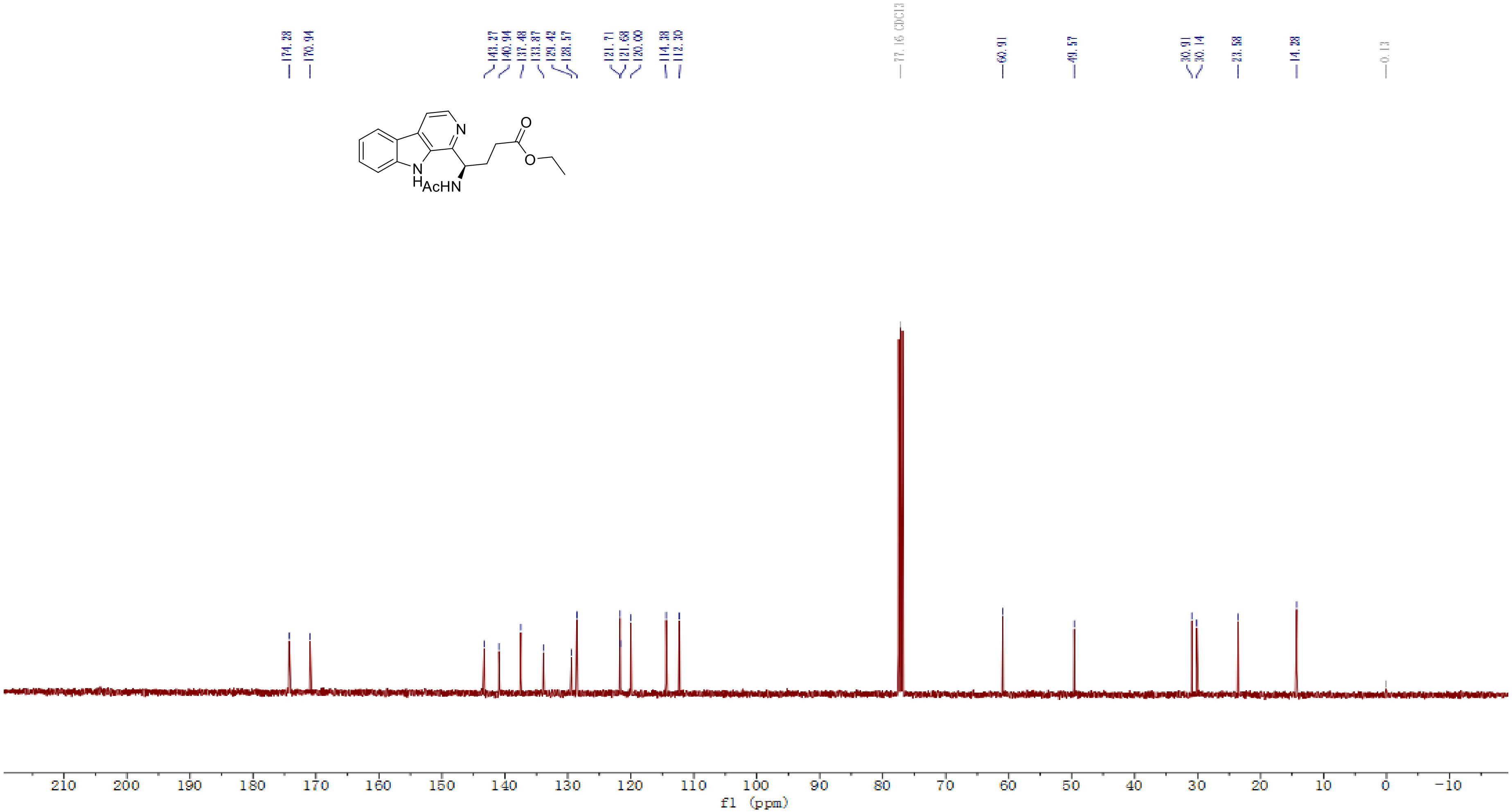

[1]H NMR (400 MHz, $CDCl_3$) (*R*)-pentyl-4-acetamido-4-(9*H*-pyrido[3,4-*b*]indol-1-yl)butanoate (**4b**)

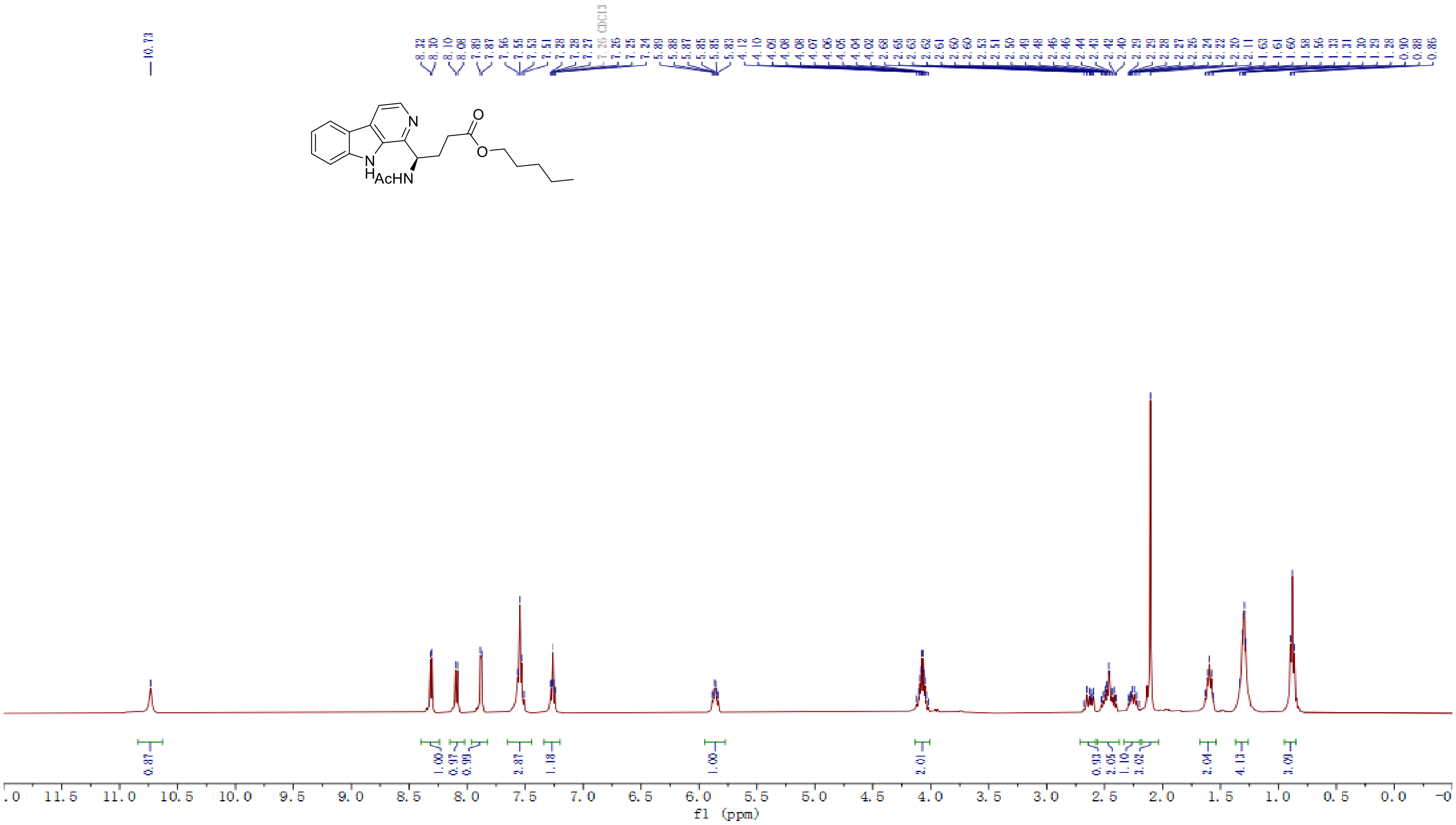

$^{13}C$ NMR (101 MHz, $CDCl_3$) (*R*)-pentyl-4-acetamido-4-(9*H*-pyrido[3,4-*b*]indol-1-yl)butanoate (**4b**)

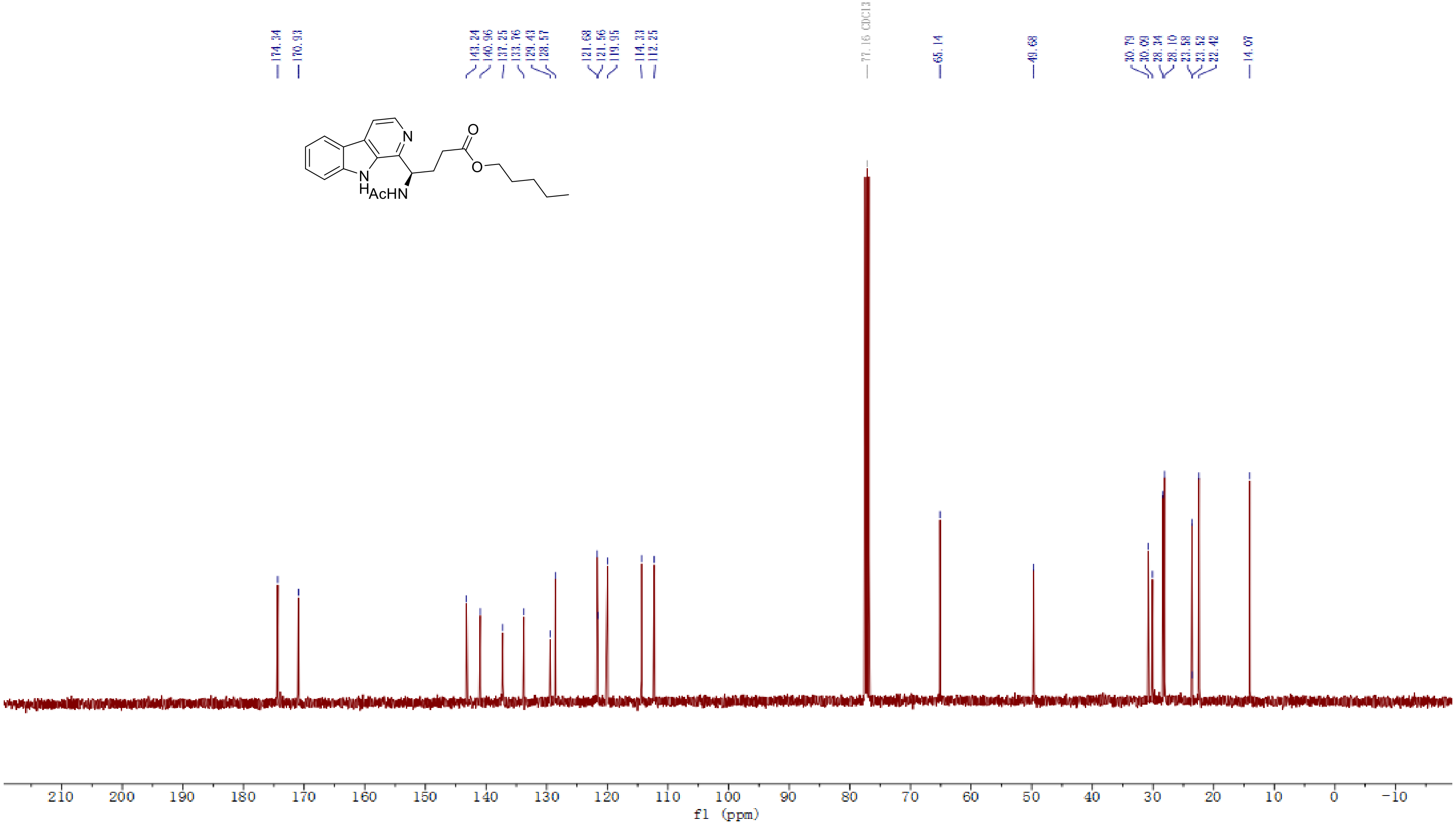

[1]H NMR (400 MHz, $CDCl_3$) (*R*)-isopropyl-4-acetamido-4-(9*H*-pyrido[3,4-*b*]indol-1-yl)butanoate (**4c**)

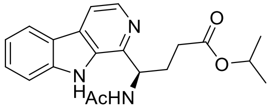

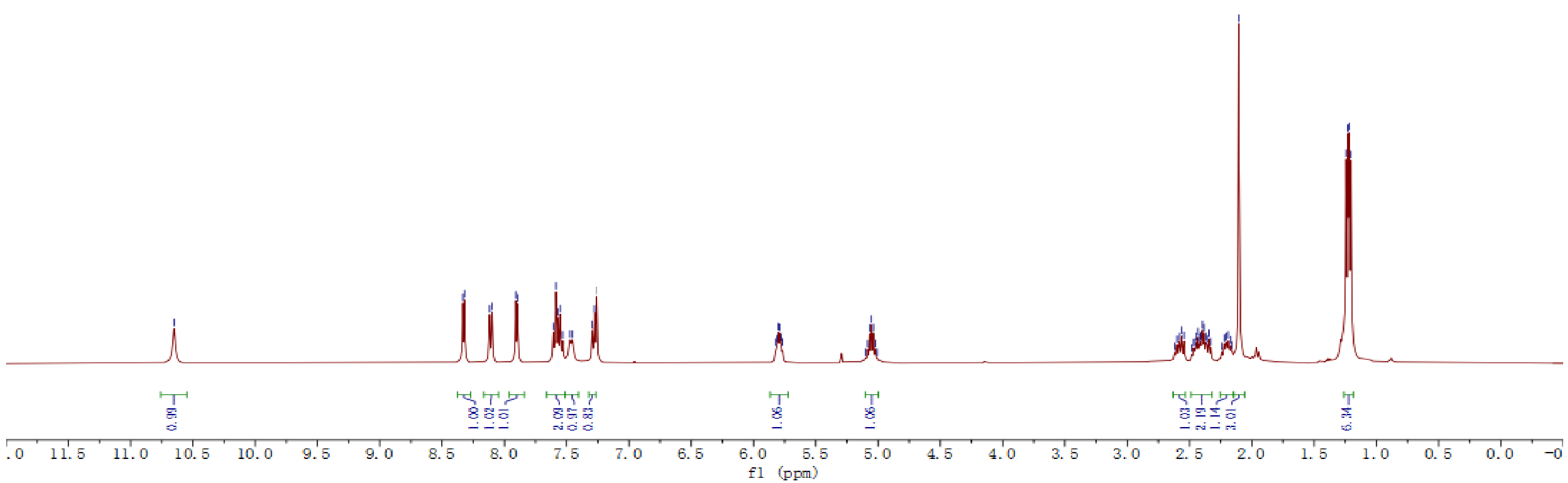

$^{13}C$ NMR (101 MHz, $CDCl_3$) (*R*)-isopropyl-4-acetamido-4-(9*H*-pyrido[3,4-*b*]indol-1-yl)butanoate (**4c**)

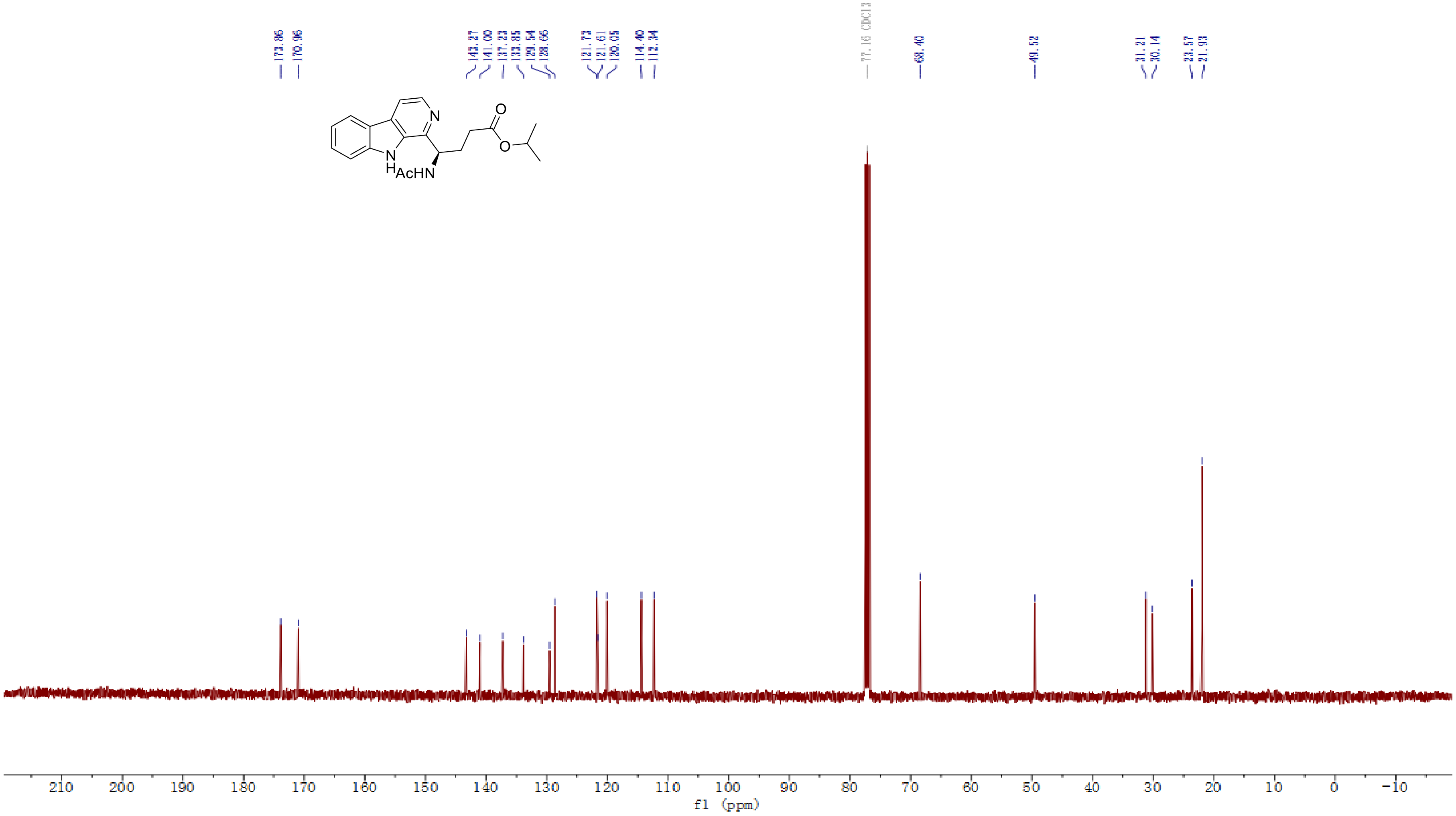

$^{1}H$ NMR (400 MHz, $CDCl_3$) (*R*)-tert-butyl-4-acetamido-4-(9*H*-pyrido[3,4-*b*]indol-1-yl)butanoate (**4d**)

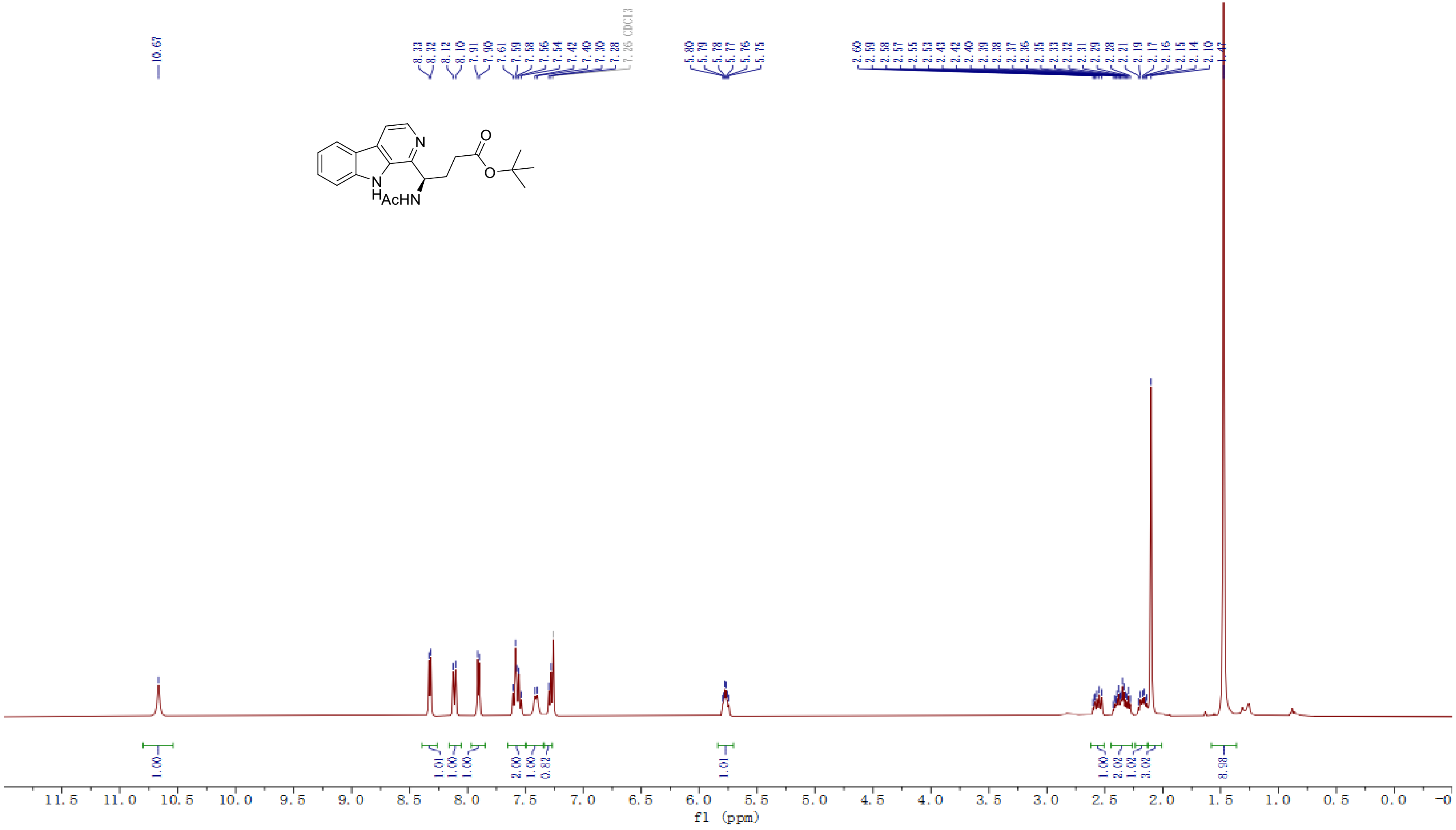

[13]C NMR (101 MHz, CDCl$_3$) (*R*)-tert-butyl-4-acetamido-4-(9*H*-pyrido[3,4-*b*]indol-1-yl)butanoate (**4d**)

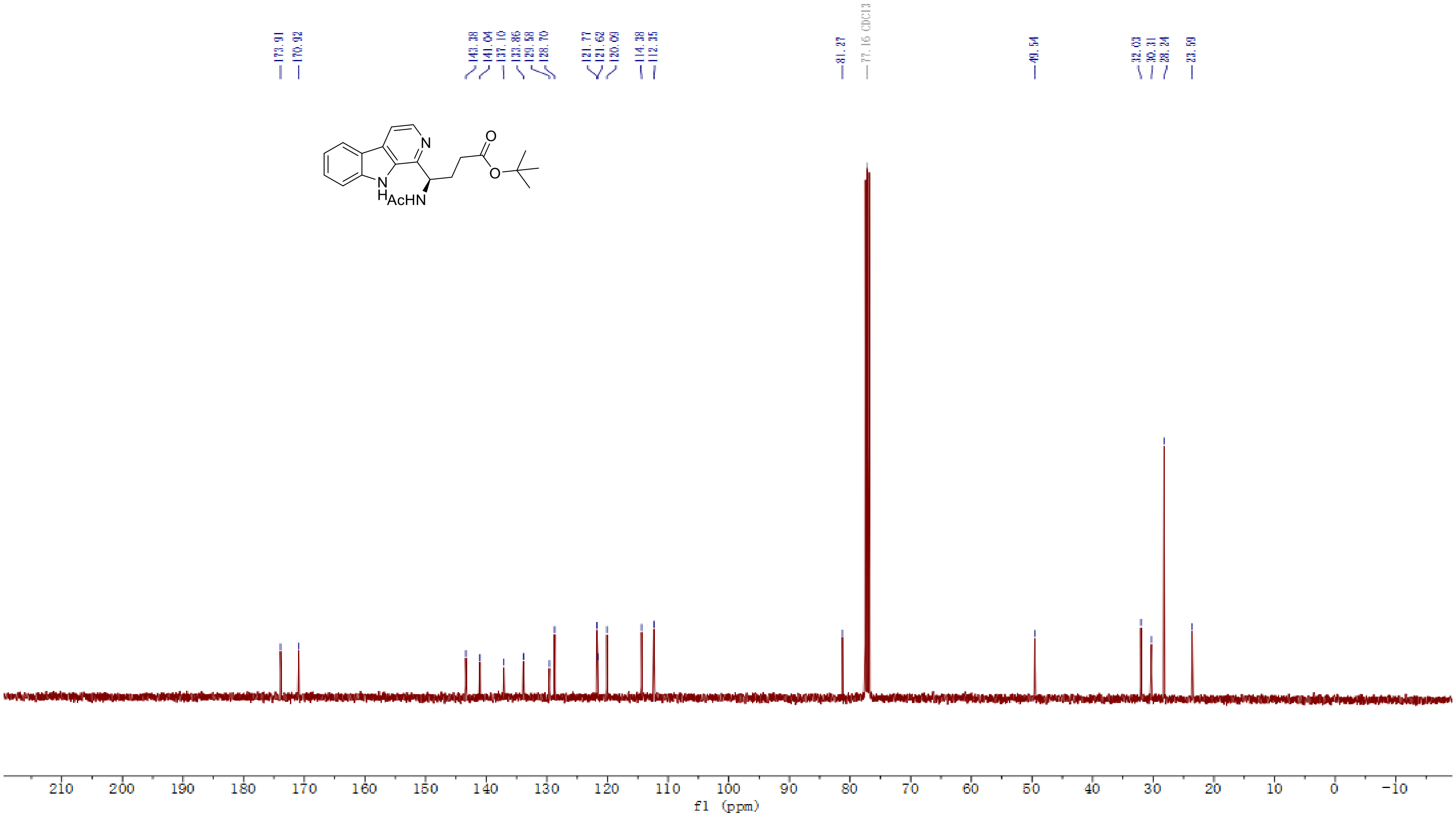

[1]H NMR (400 MHz, $CDCl_3$) (*R*)-pent-4-en-1-yl-4-acetamido-4-(9*H*-pyrido[3,4-*b*]indol-1-yl)butanoate (**4e**)

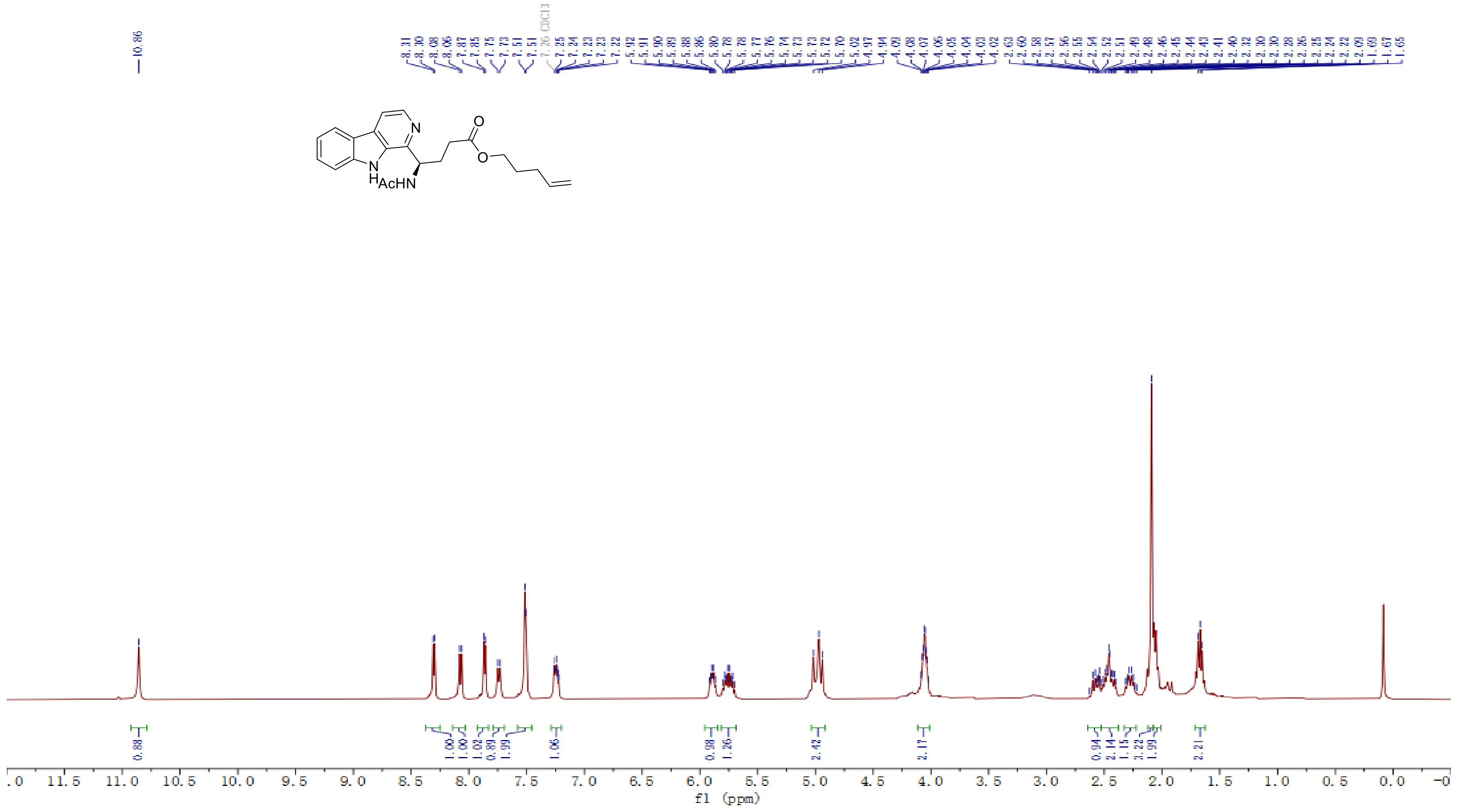

$^{13}C$ NMR (101 MHz, $CDCl_3$) (*R*)-pent-4-en-1-yl-4-acetamido-4-(9*H*-pyrido[3,4-*b*]indol-1-yl)butanoate (**4e**)

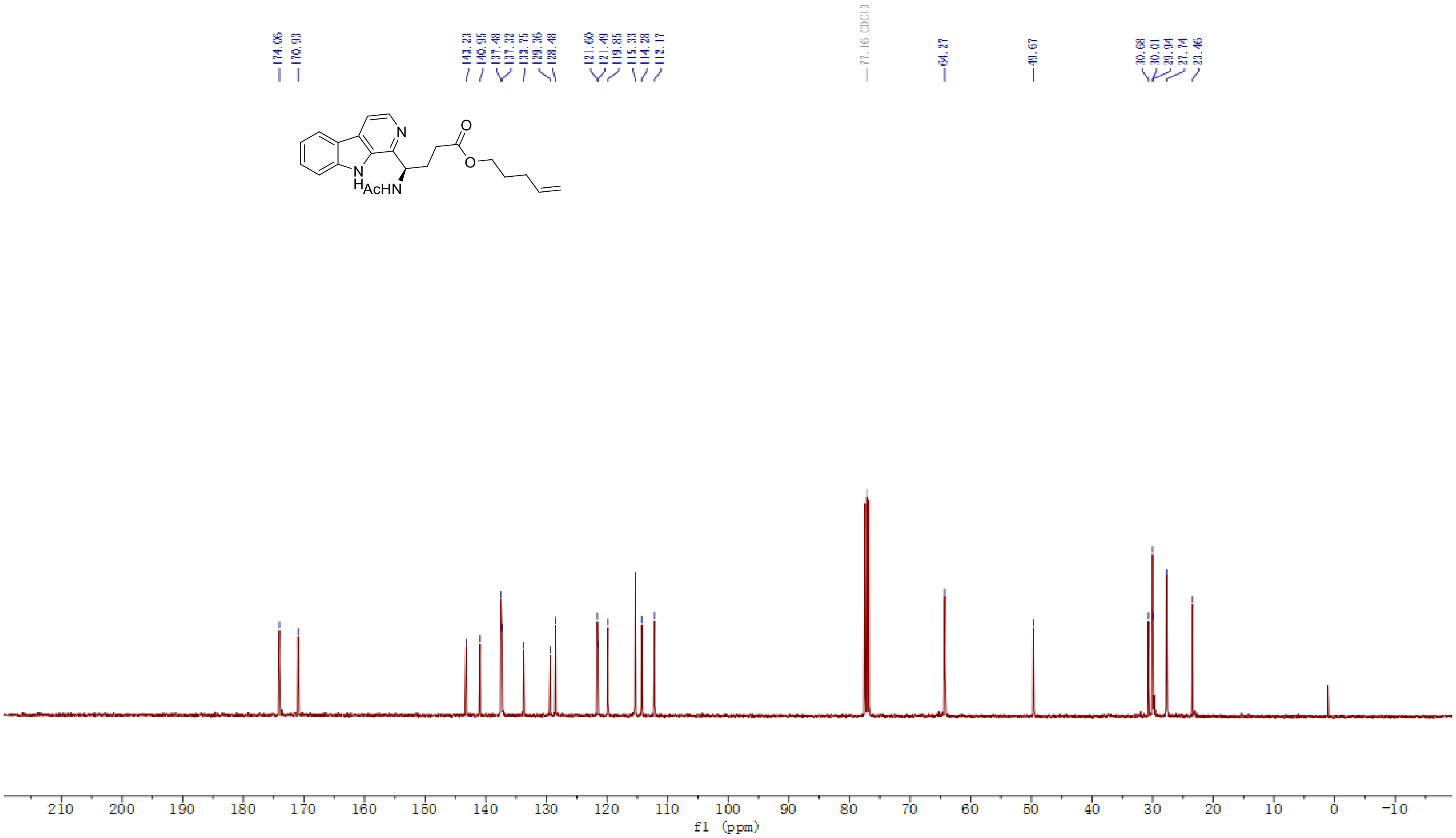

$^1$H NMR (400 MHz, DMSO-$d_6$) (*R*)-pent-4-yn-1-yl-4-acetamido-4-(9*H*-pyrido[3,4-*b*]indol-1-yl)butanoate (**4f**)

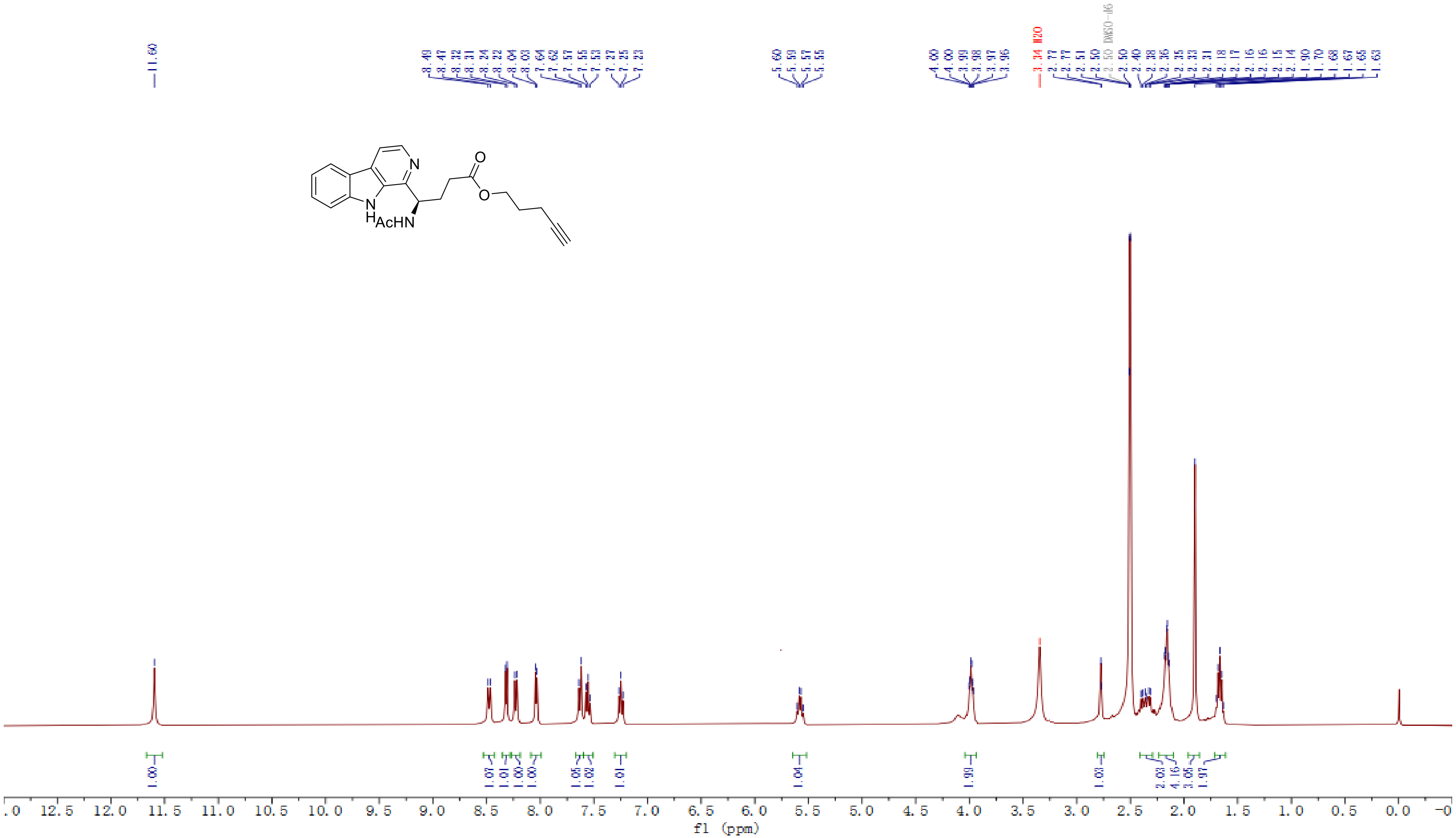

$^{13}C$ NMR (101 MHz, $CDCl_3$) (*R*)-pent-4-yn-1-yl-4-acetamido-4-(9*H*-pyrido[3,4-*b*]indol-1-yl)butanoate (**4f**)

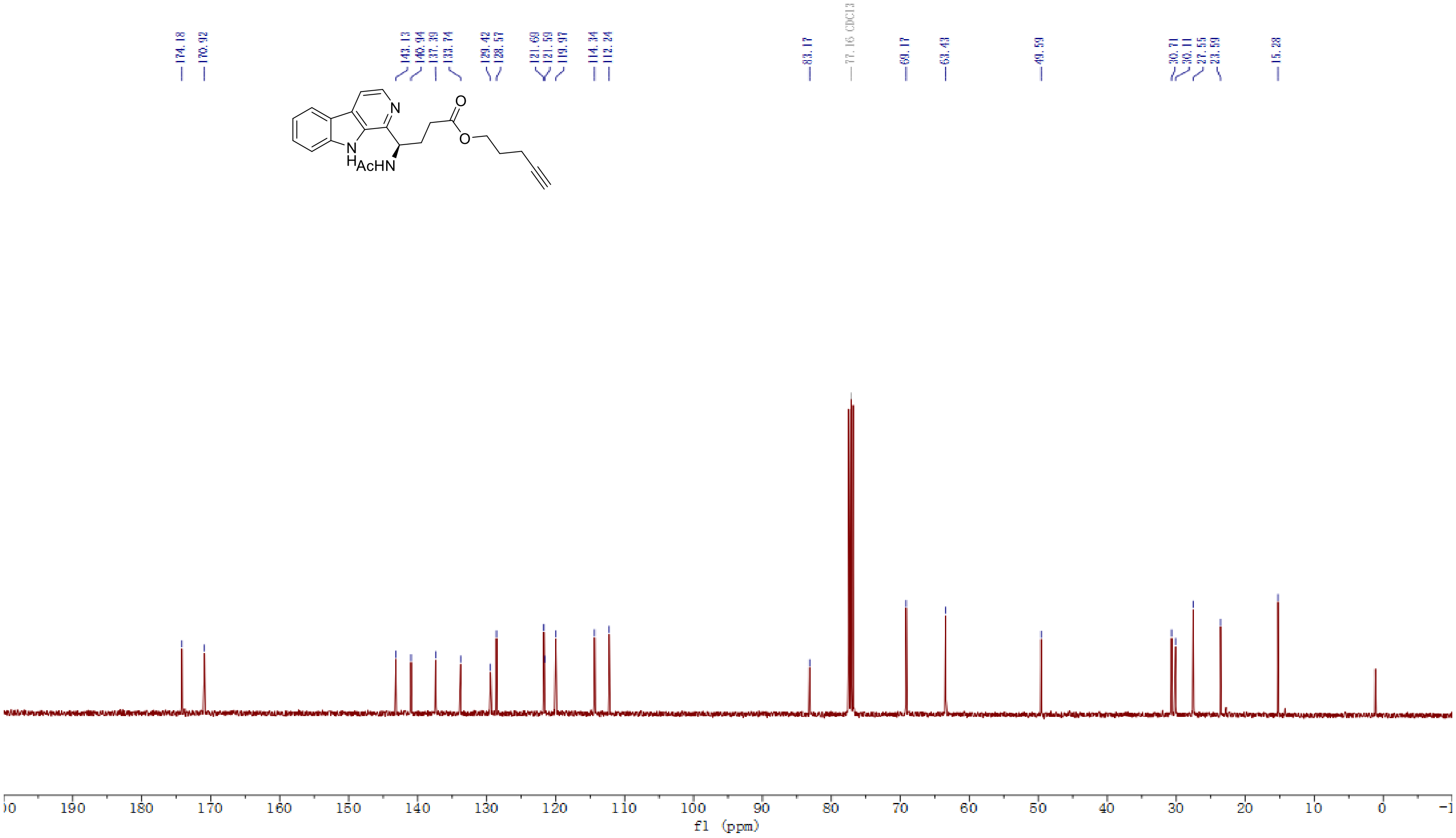

[1]H NMR (400 MHz, DMSO-$d_6$) (*R*)-phenyl-4-acetamido-4-(9*H*-pyrido[3,4-*b*]indol-1-yl)butanoate (**4g**)

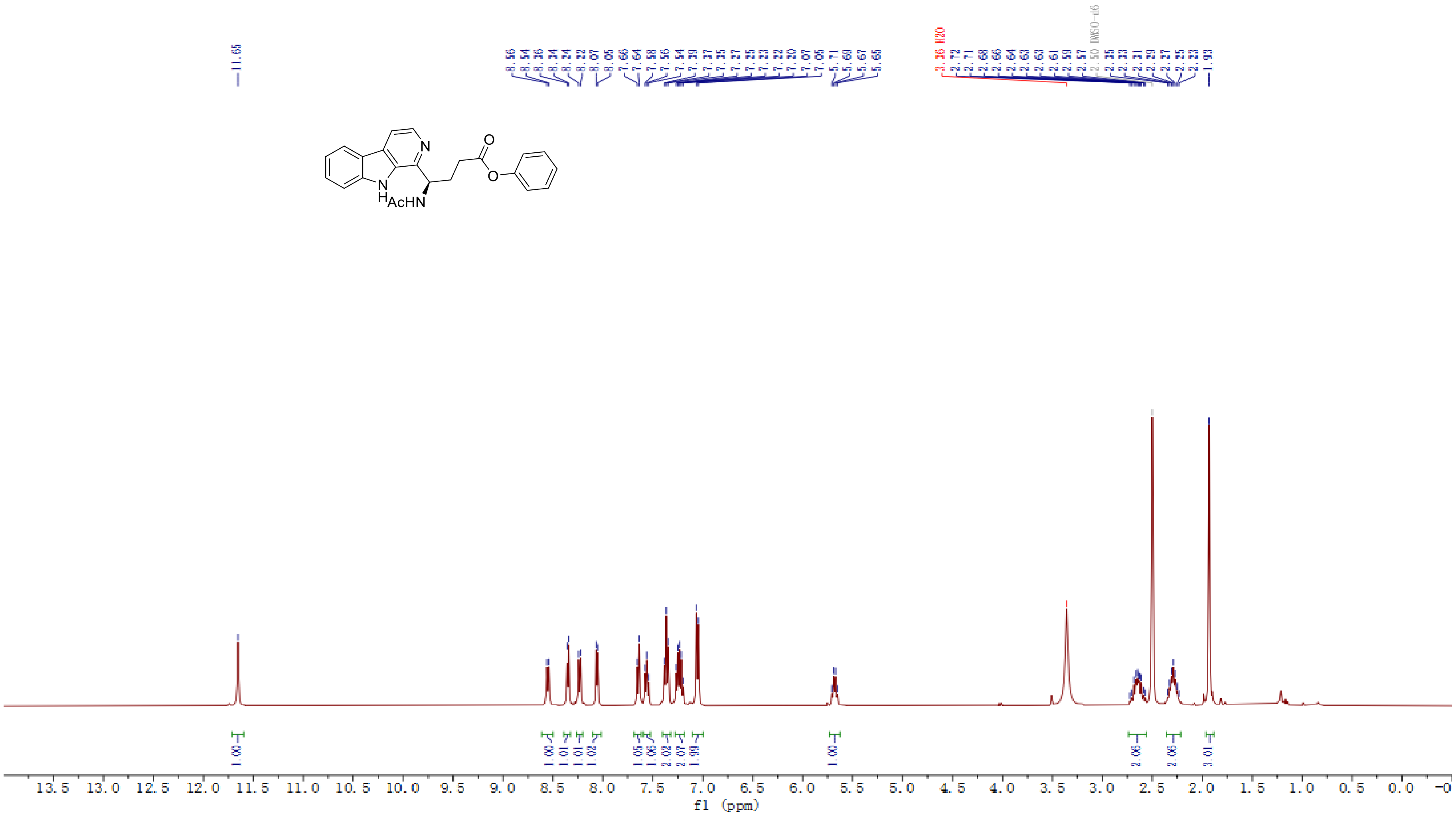

[13]C NMR (101 MHz, DMSO-$d_6$) (*R)*-phenyl-4-acetamido-4-(9*H*-pyrido[3,4-*b*]indol-1-yl)butanoate (**4g**)

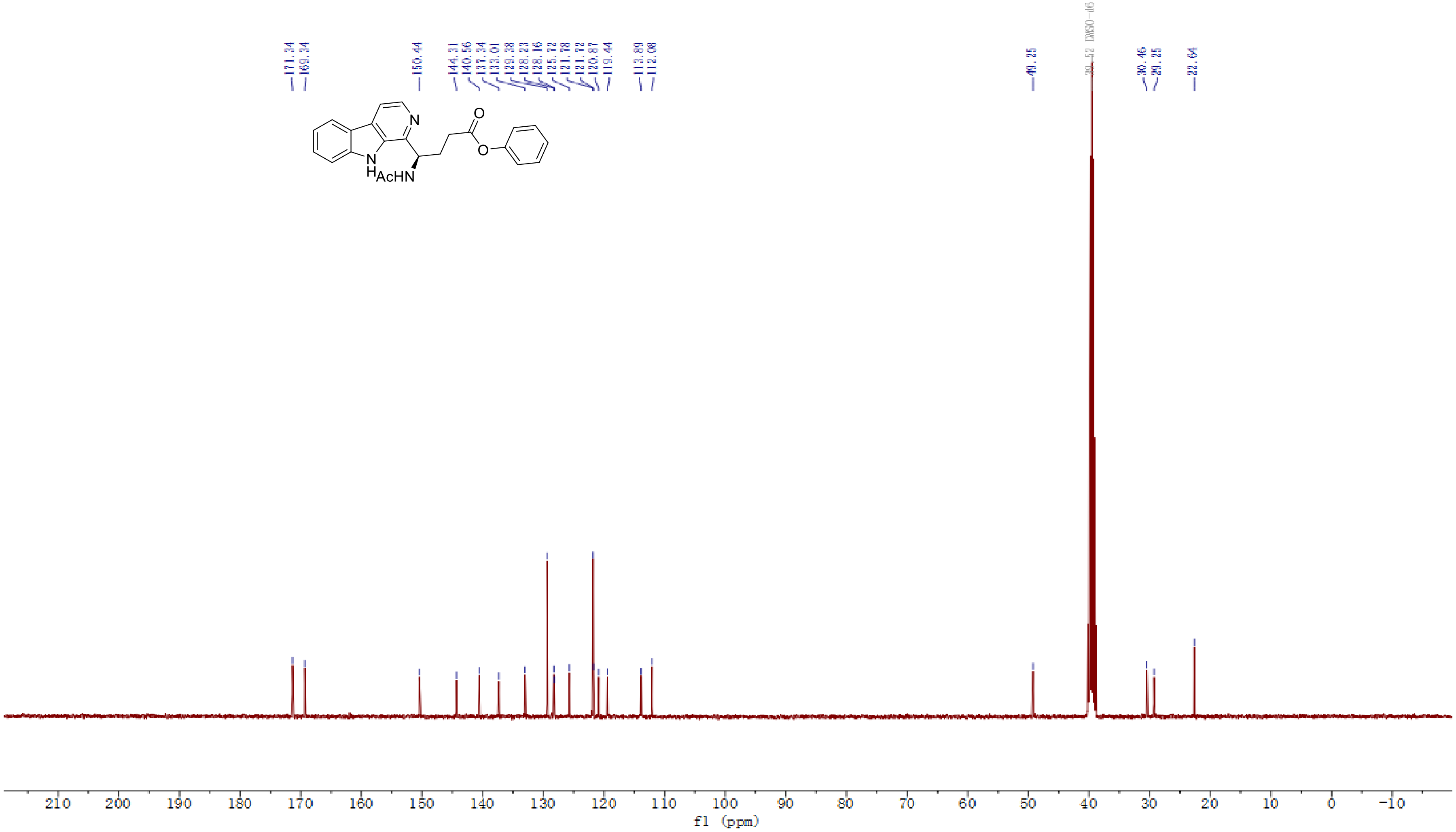

$^{1}H$ NMR (400 MHz, $CDCl_3$) (*R*)-4-methoxyphenyl-4-acetamido-4-(9*H*-pyrido[3,4-*b*]indol-1-yl) butanoate (**4h**)

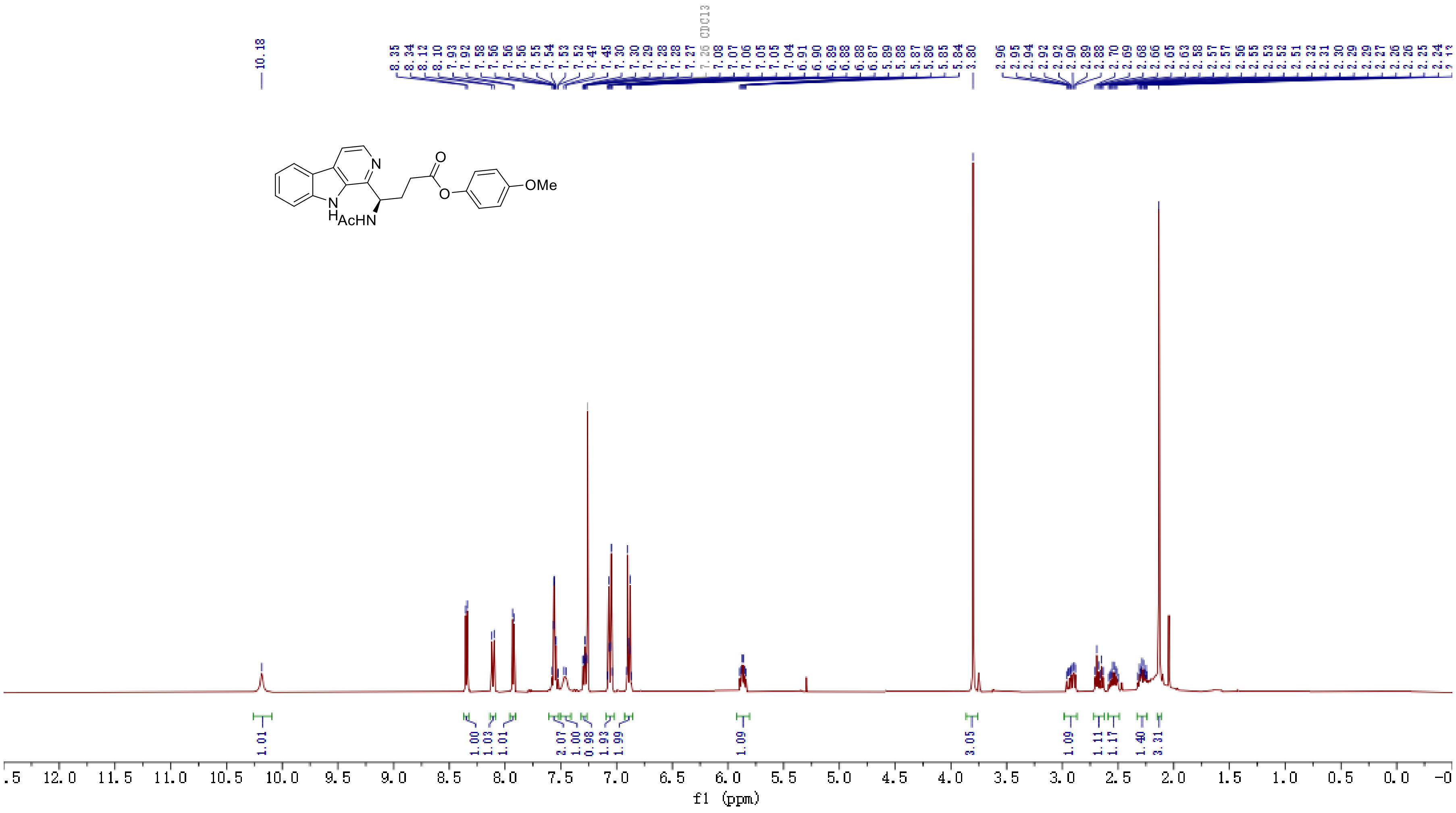

$^{13}C$ NMR (101 MHz, $CDCl_3$) (*R*)-4-methoxyphenyl-4-acetamido-4-(9*H*-pyrido[3,4-*b*]indol-1-yl) butanoate (**4h**)

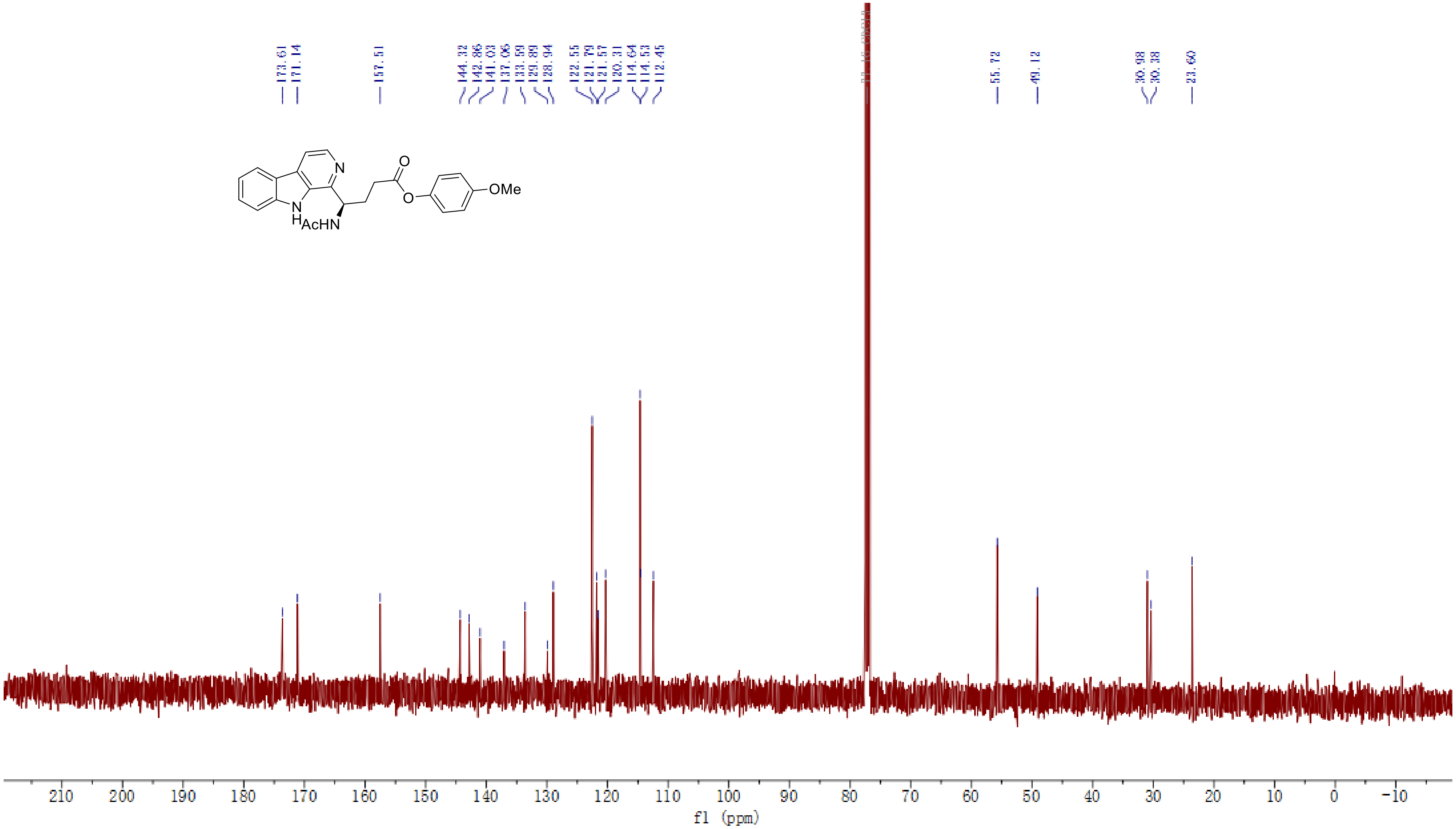

[1]H NMR (400 MHz, $CDCl_3$) (*R*)-4-fluorophenyl-4-acetamido-4-(9*H*-pyrido[3,4-*b*]indol-1-yl)butanoate (**4i**)

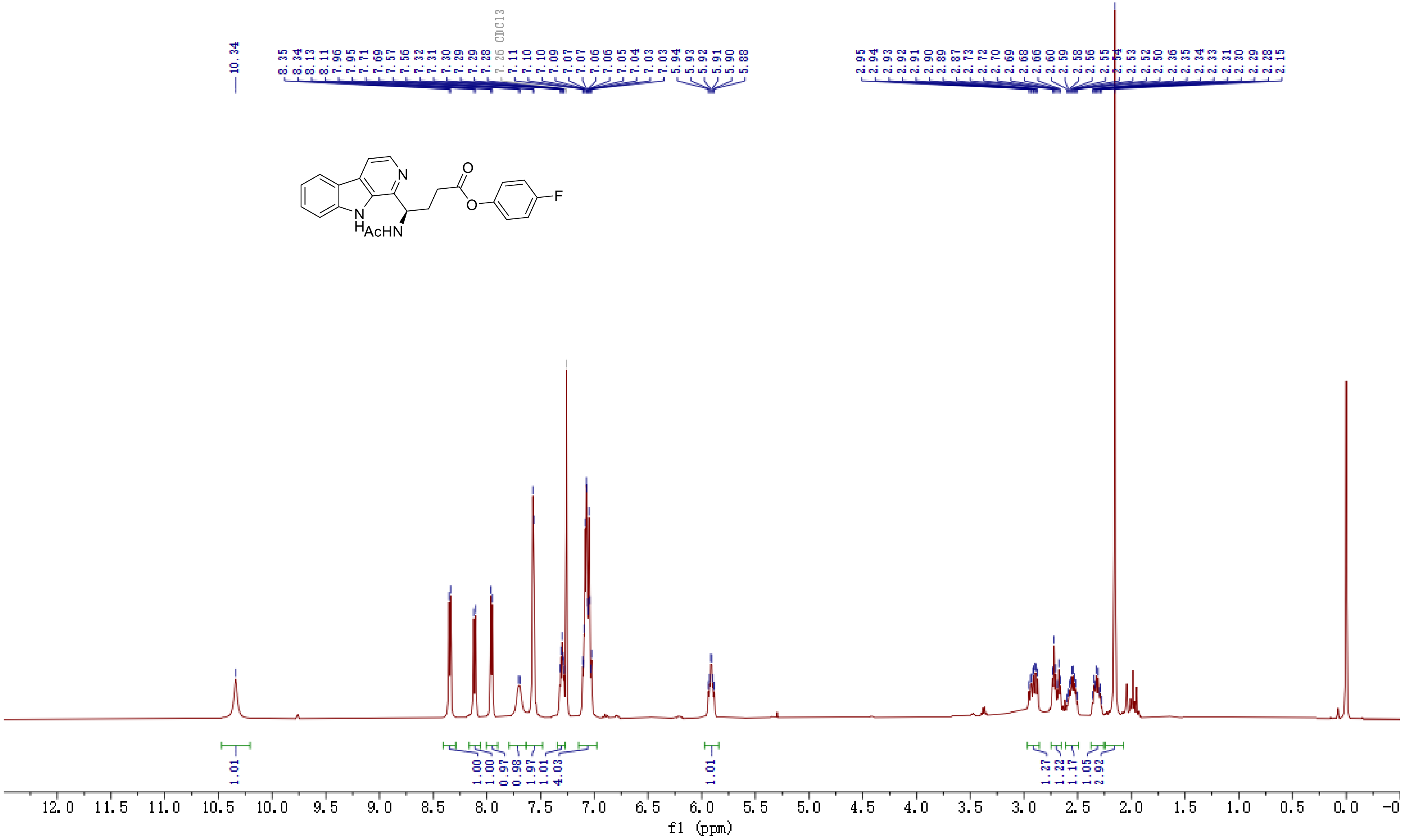

$^{13}$C NMR (101 MHz, $CDCl_3$) (*R*)-4-fluorophenyl-4-acetamido-4-(9*H*-pyrido[3,4-*b*]indol-1-yl)butan-oate (**4i**)

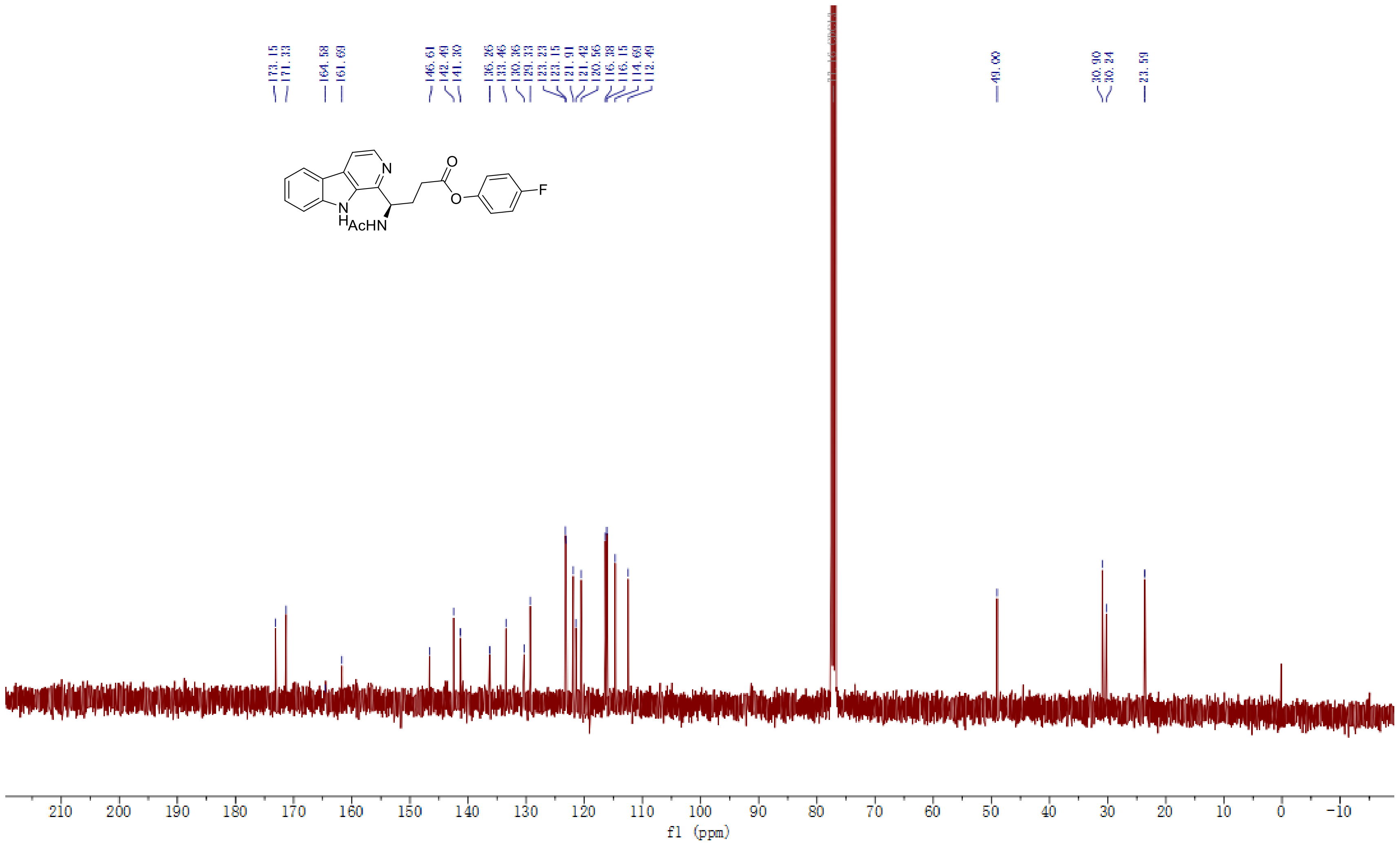

$^{19}$F NMR (376 MHz, $CDCl_3$) (*R*)-4-fluorophenyl-4-acetamido-4-(9*H*-pyrido[3,4-*b*]indol-1-yl)butan-oate (**4i**)

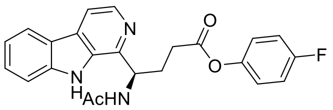

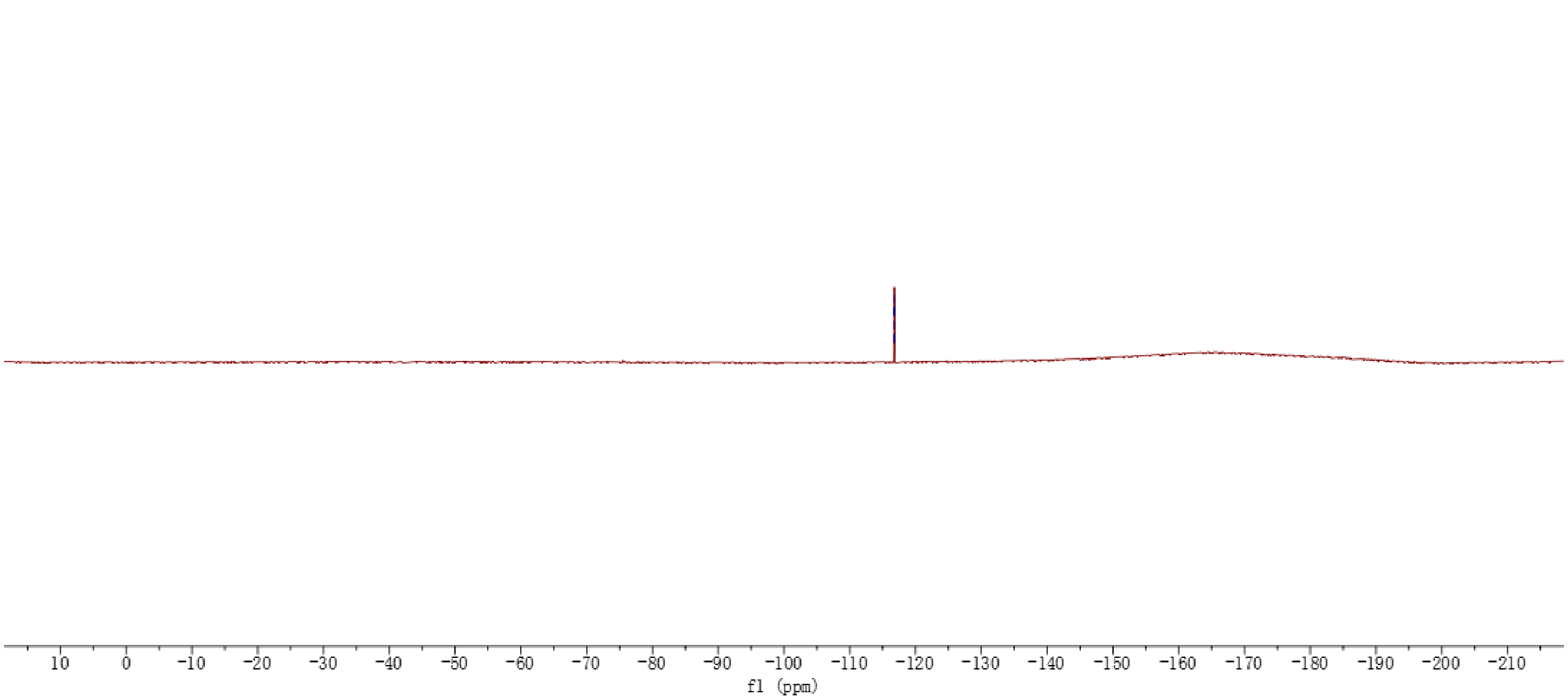

$^{1}H$ NMR (400 MHz, $CDCl_3$) (*R*)-benzyl-4-acetamido-4-(9*H*-pyrido[3,4-*b*]indol-1-yl)butanoate(**4j**)

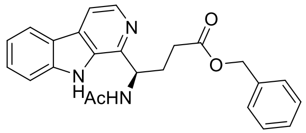


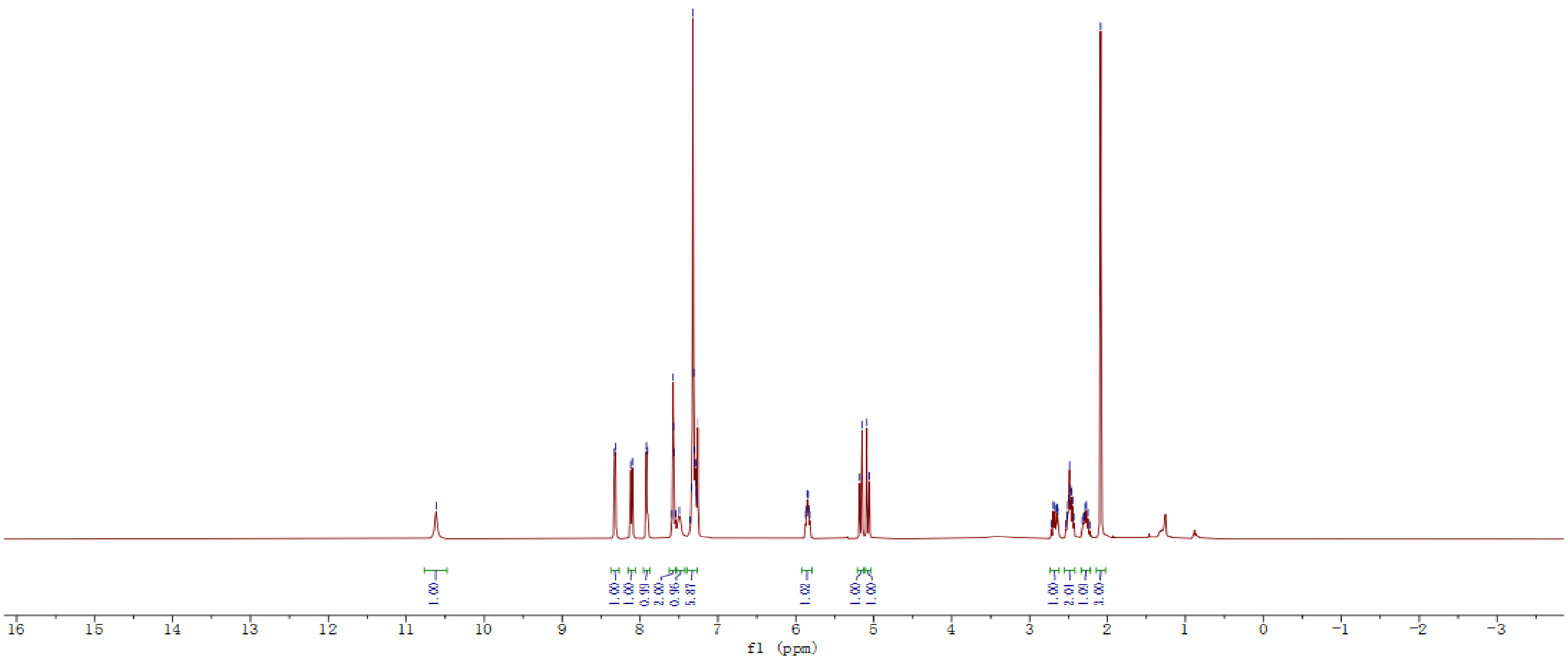

$^{13}$C NMR (101 MHz, $CDCl_3$) (*R*)-benzyl-4-acetamido-4-(9*H*-pyrido[3,4-*b*]indol-1-yl)butanoate(**4j**)

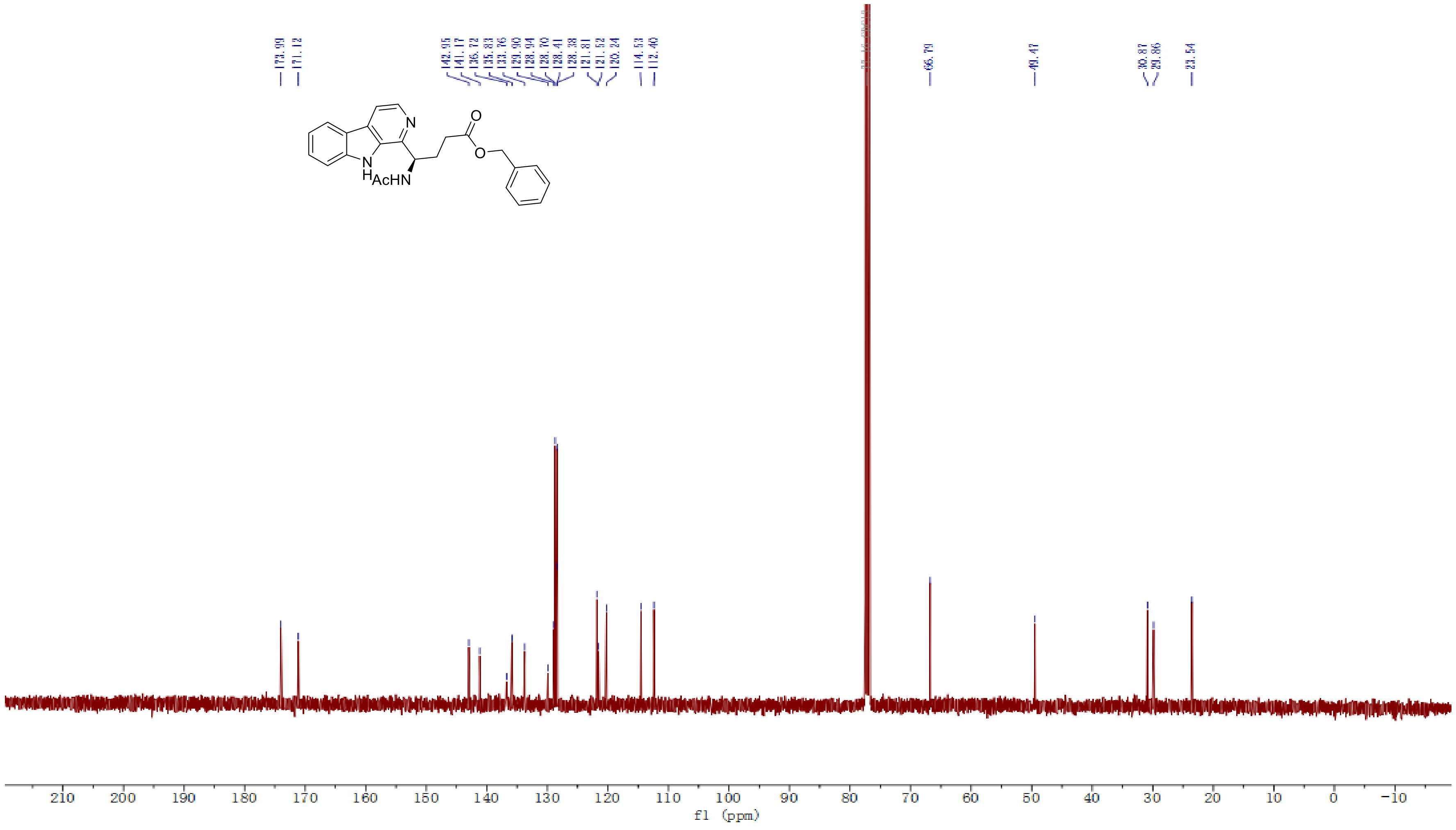

[1]H NMR (400 MHz, $CDCl_3$) (*R*)-4-methoxybenzyl-4-acetamido-4-(9*H*-pyrido[3,4-*b*]indol-1-yl)butanoate(**4k**)

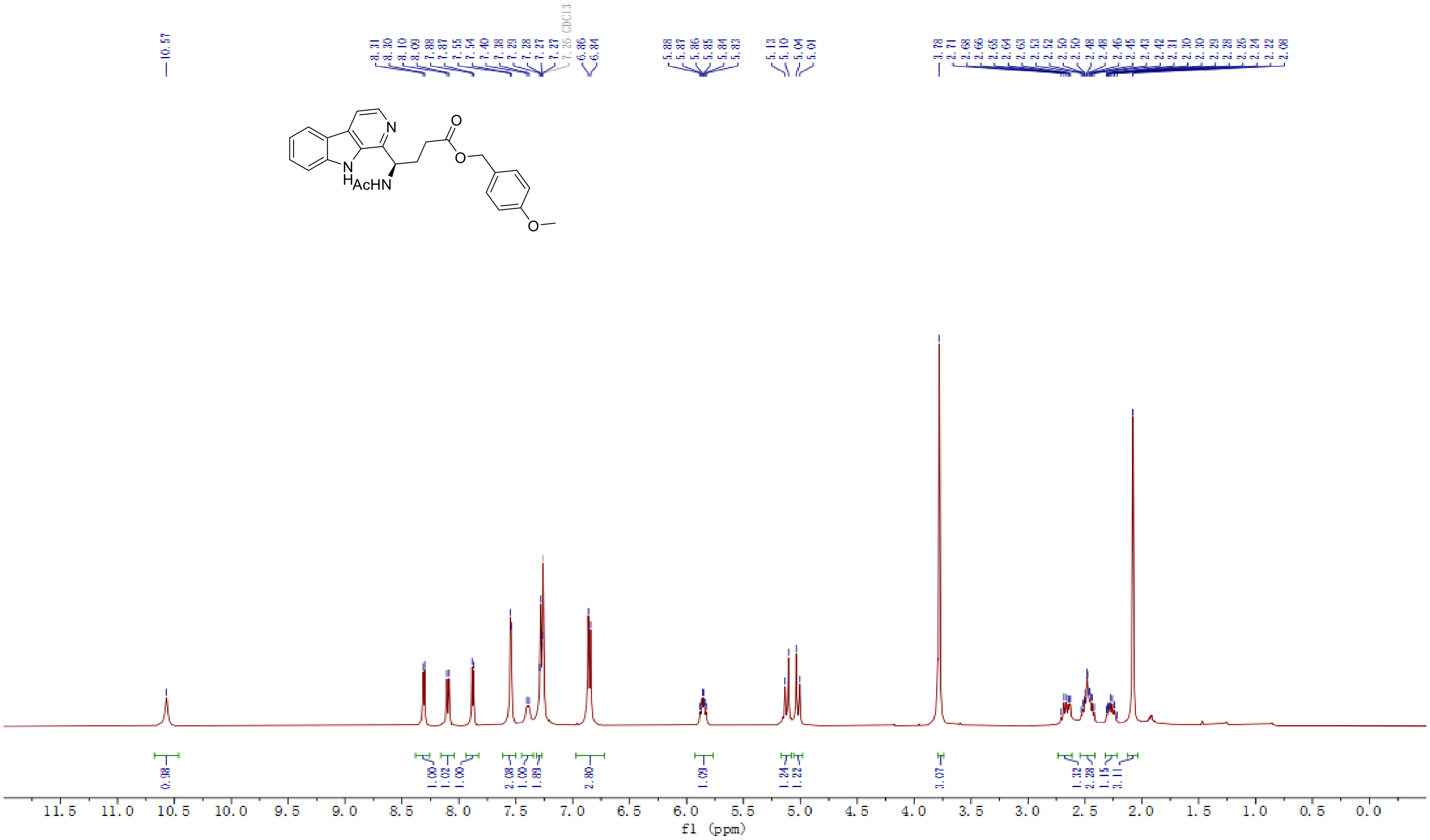

$^{13}C$ NMR (101 MHz, $CDCl_3$) (R)-4-methoxybenzyl-4-acetamido-4-(9H-pyrido[3,4-*b*]indol-1-yl)butanoate(**4k**)

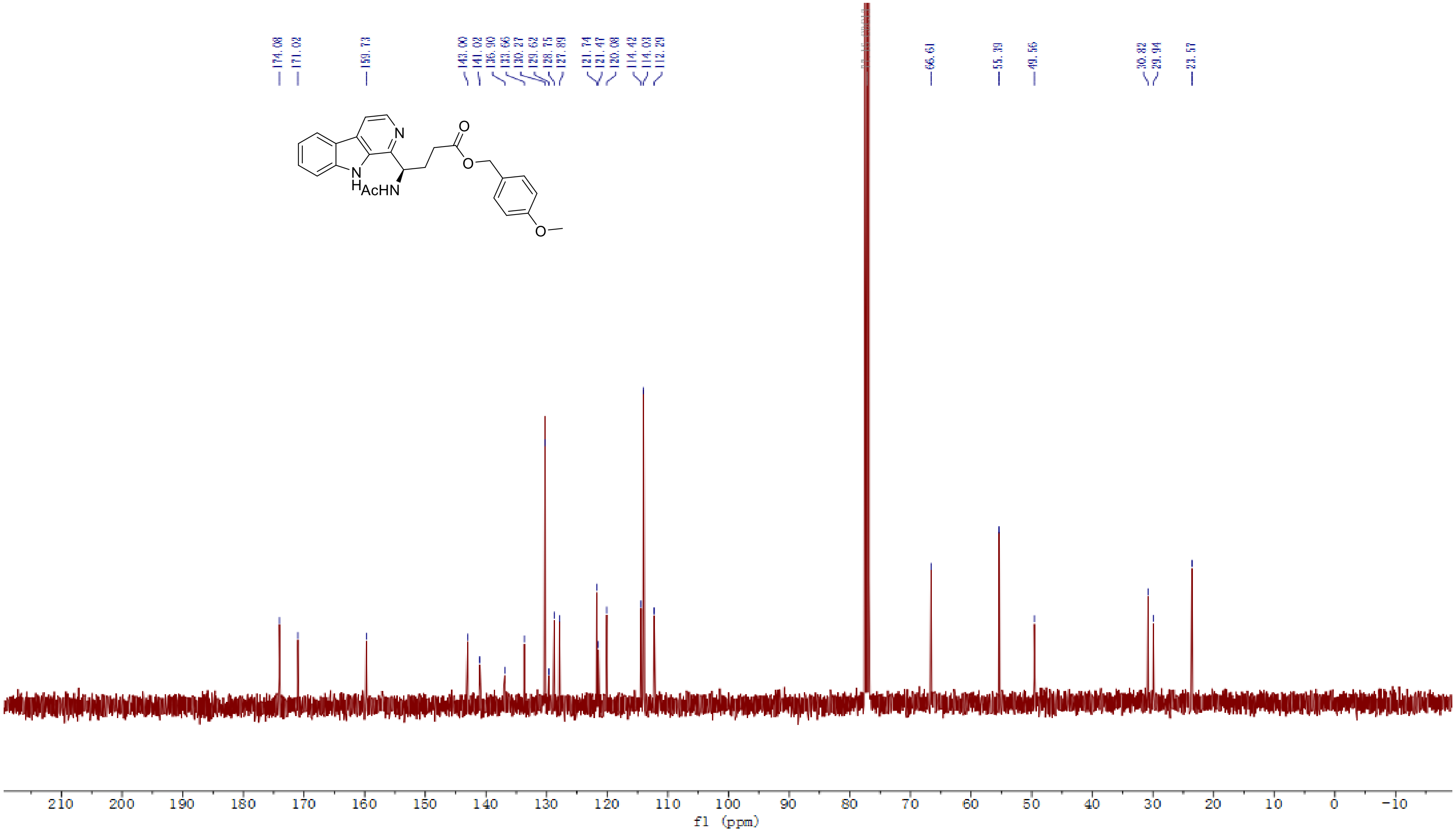

[1]H NMR (400 MHz, $CDCl_3$) (*R*)-4-fluorobenzyl-4-acetamido-4-(9*H*-pyrido[3,4-*b*]indol-1-yl)butanoate (**4l**)

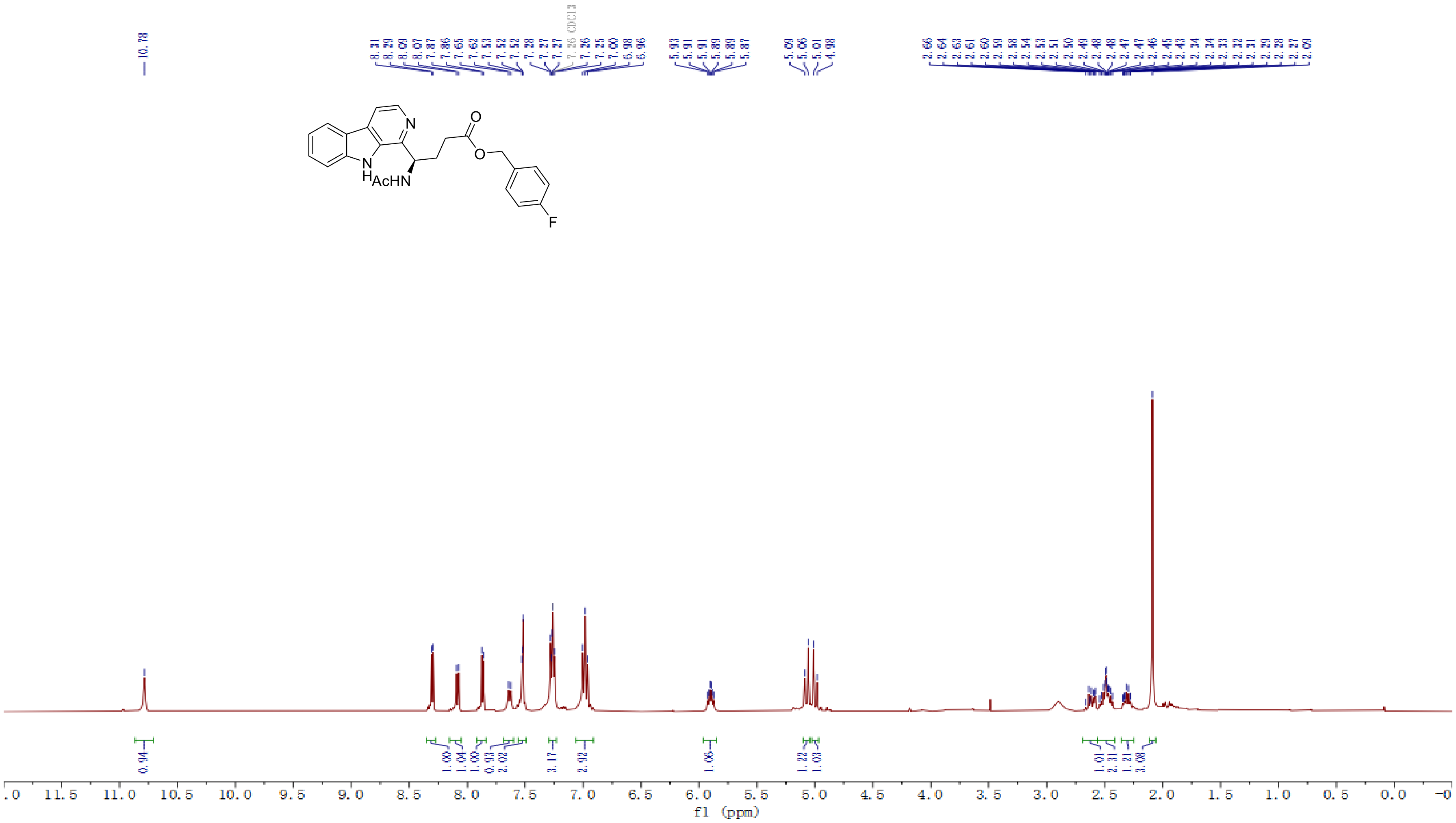

$^{13}$C NMR (101 MHz, $CDCl_3$) (*R*)-4-fluorobenzyl-4-acetamido-4-(9*H*-pyrido[3,4-*b*]indol-1-yl)butanoate (**4l**)

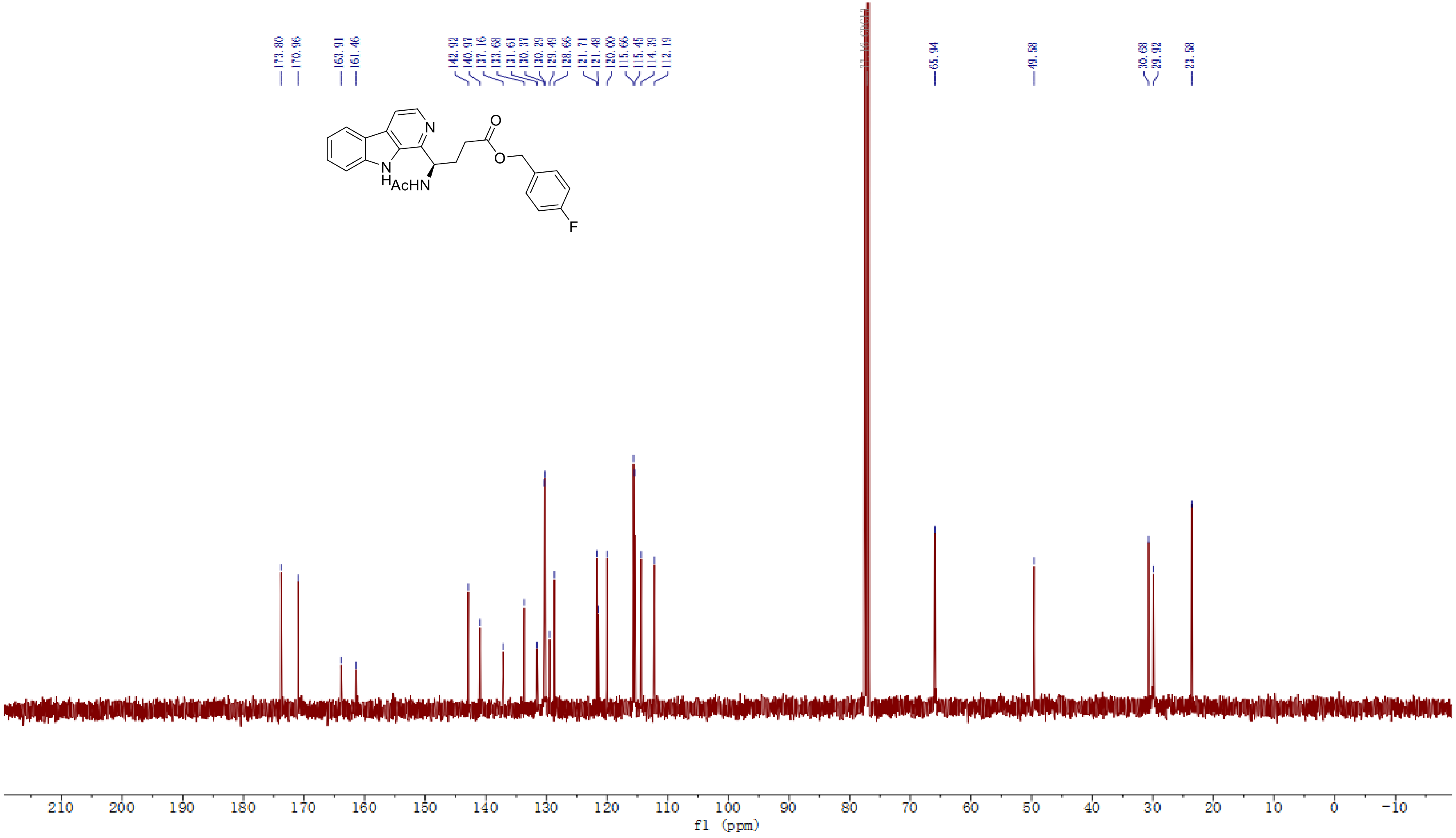

[19]F NMR (376 MHz, $CDCl_3$) (*R*)-4-fluorobenzyl-4-acetamido-4-(9*H*-pyrido[3,4-*b*]indol-1-yl)butanoate (**4l**)

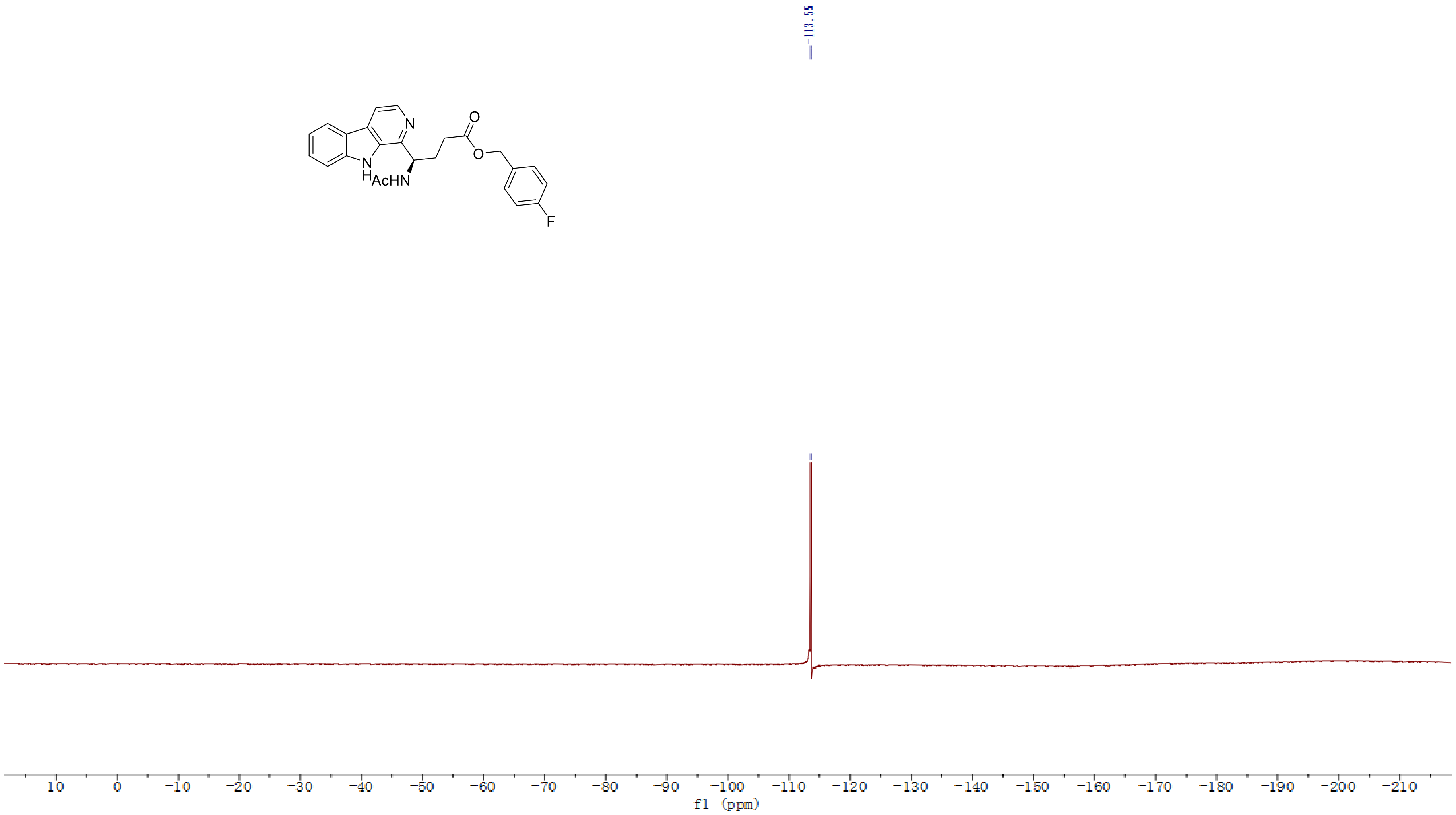

$^1$H NMR (400 MHz, $CDCl_3$) (*R*)-*N*-(5,5-dimethyl-4-oxo-1-(9*H*-pyrido[3,4-*b*]indol-1-yl)hexyl)acetamide (**4m**)

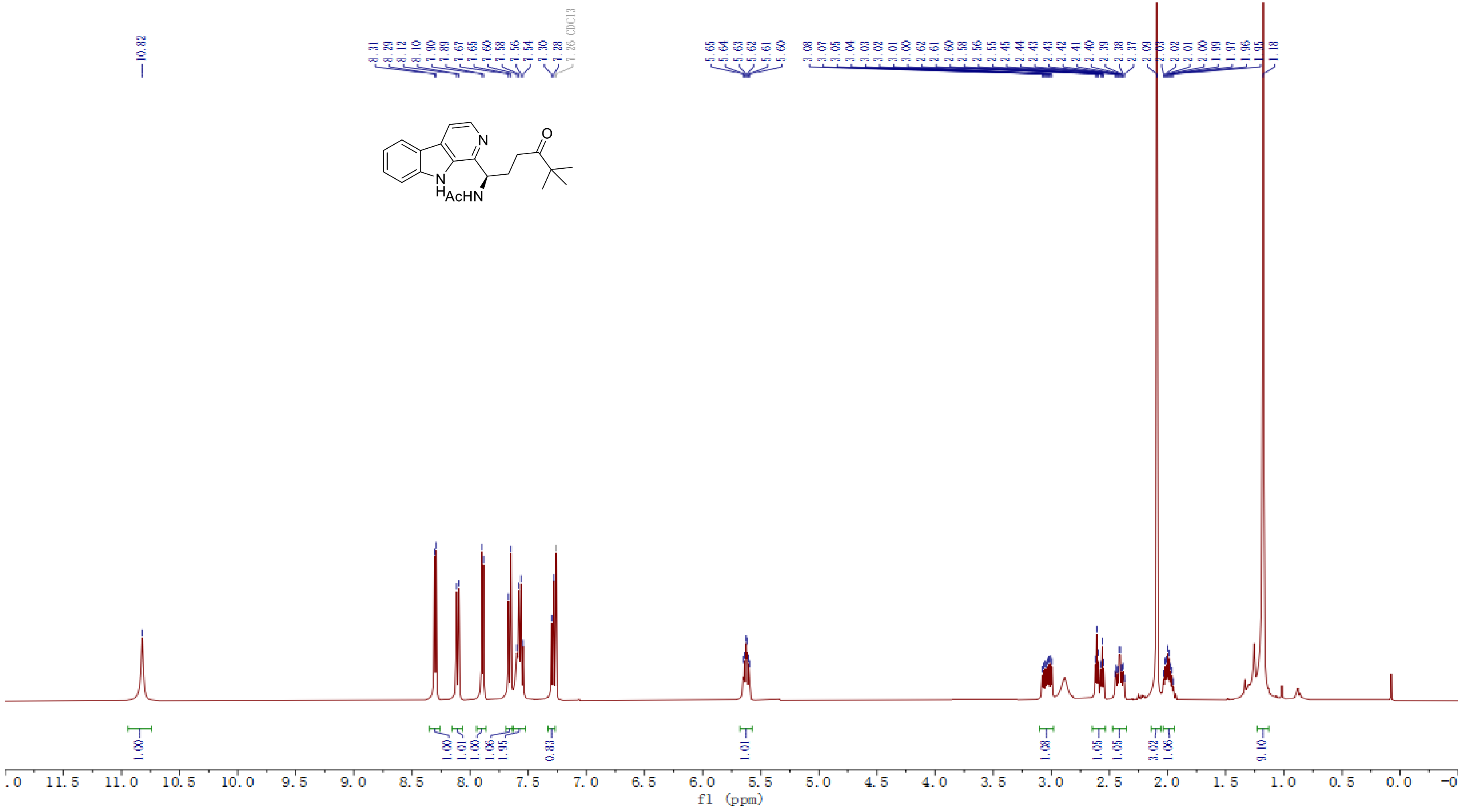

$^{13}C$ NMR (101 MHz, $CDCl_3$) (*R*)-*N*-(5,5-dimethyl-4-oxo-1-(9*H*-pyrido[3,4-*b*]indol-1-yl)hexyl)acetamide (**4m**)

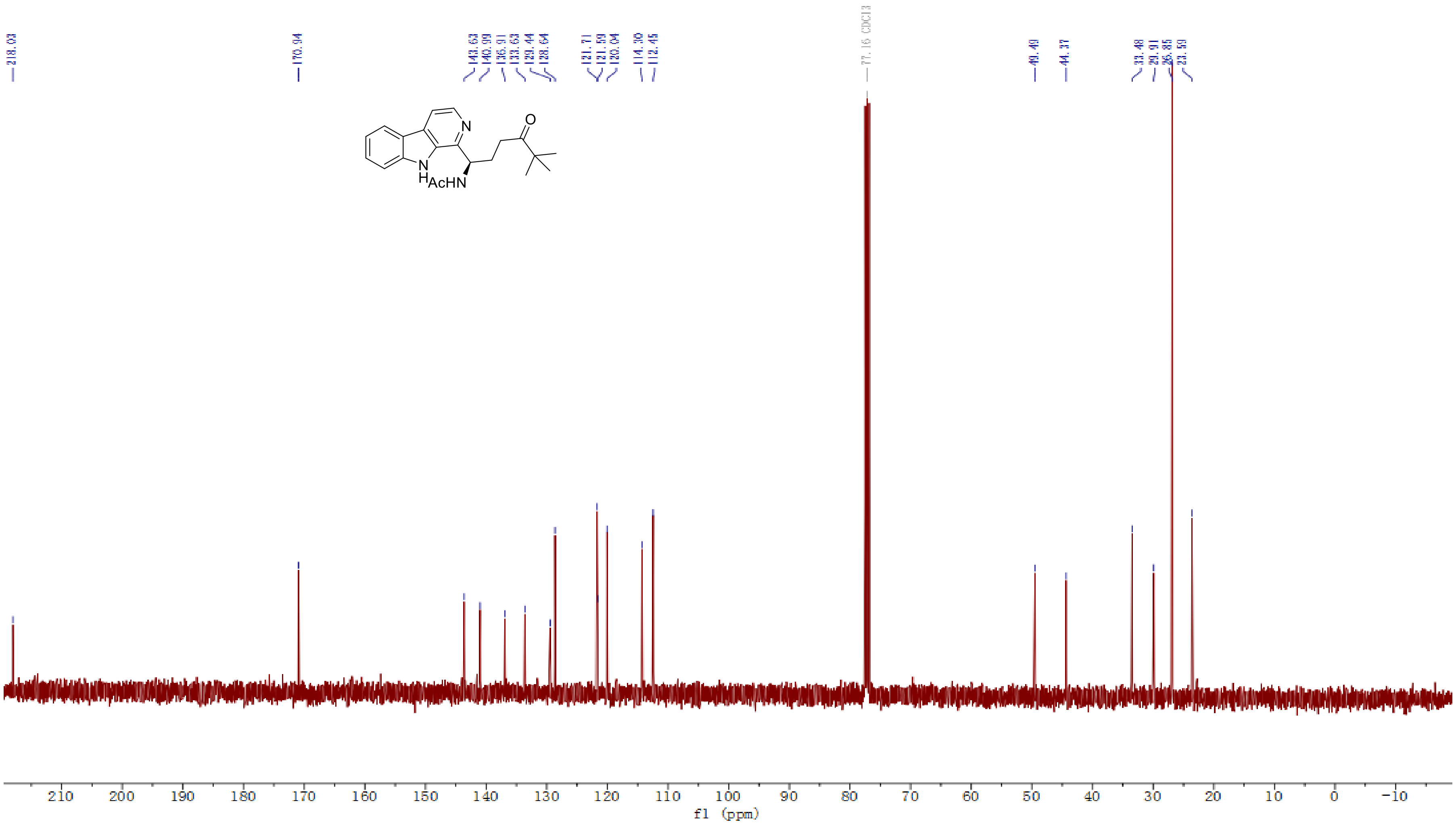

[1]H NMR (400 MHz, CDCl3) (*R*)-*N*-(4-oxo-4-phenyl-1-(9*H*-pyrido[3,4-*b*]indol-1-yl)butyl)acetamide (**4n**)

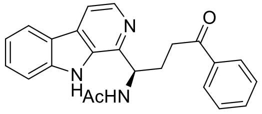


4an

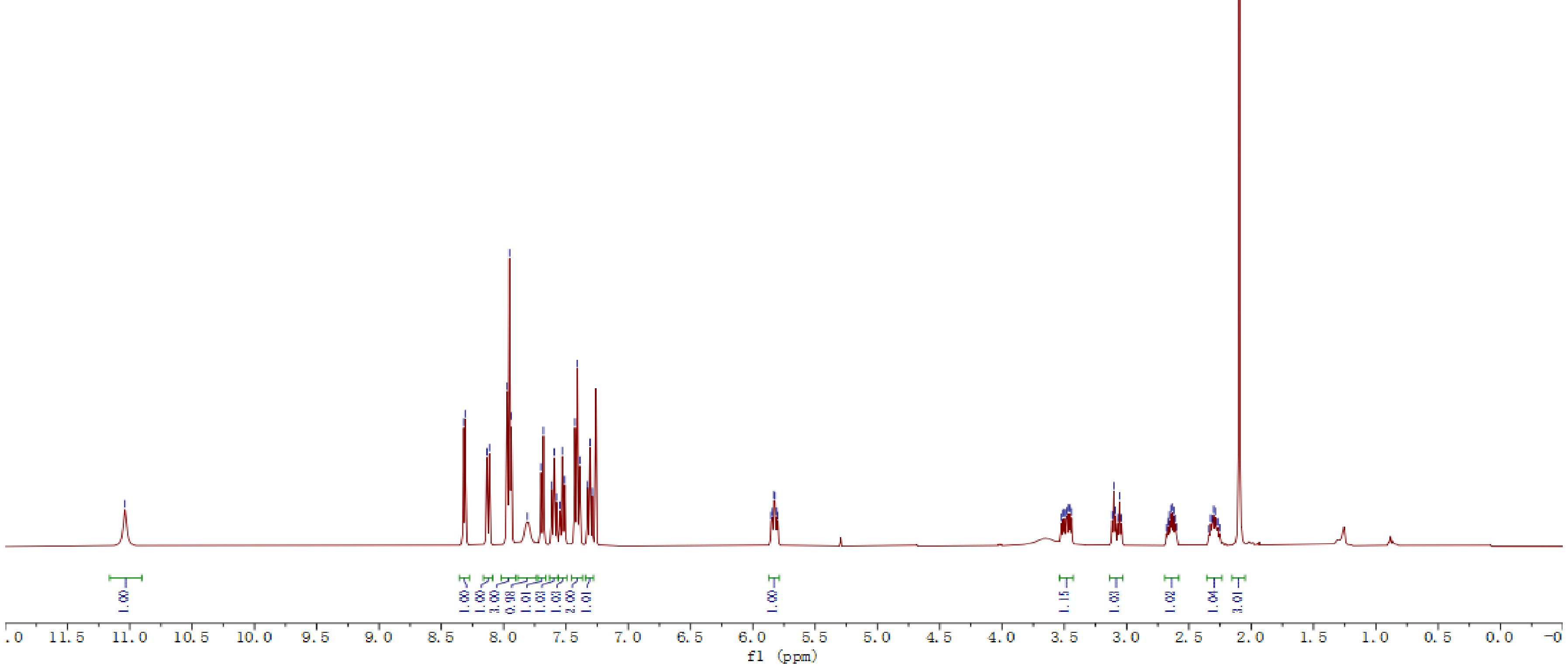

$^{13}C$ NMR (101 MHz, $CDCl_3$) (*R*)-*N*-(4-oxo-4-phenyl-1-(9*H*-pyrido[3,4-*b*]indol-1-yl)butyl)acetamide (**4n**)

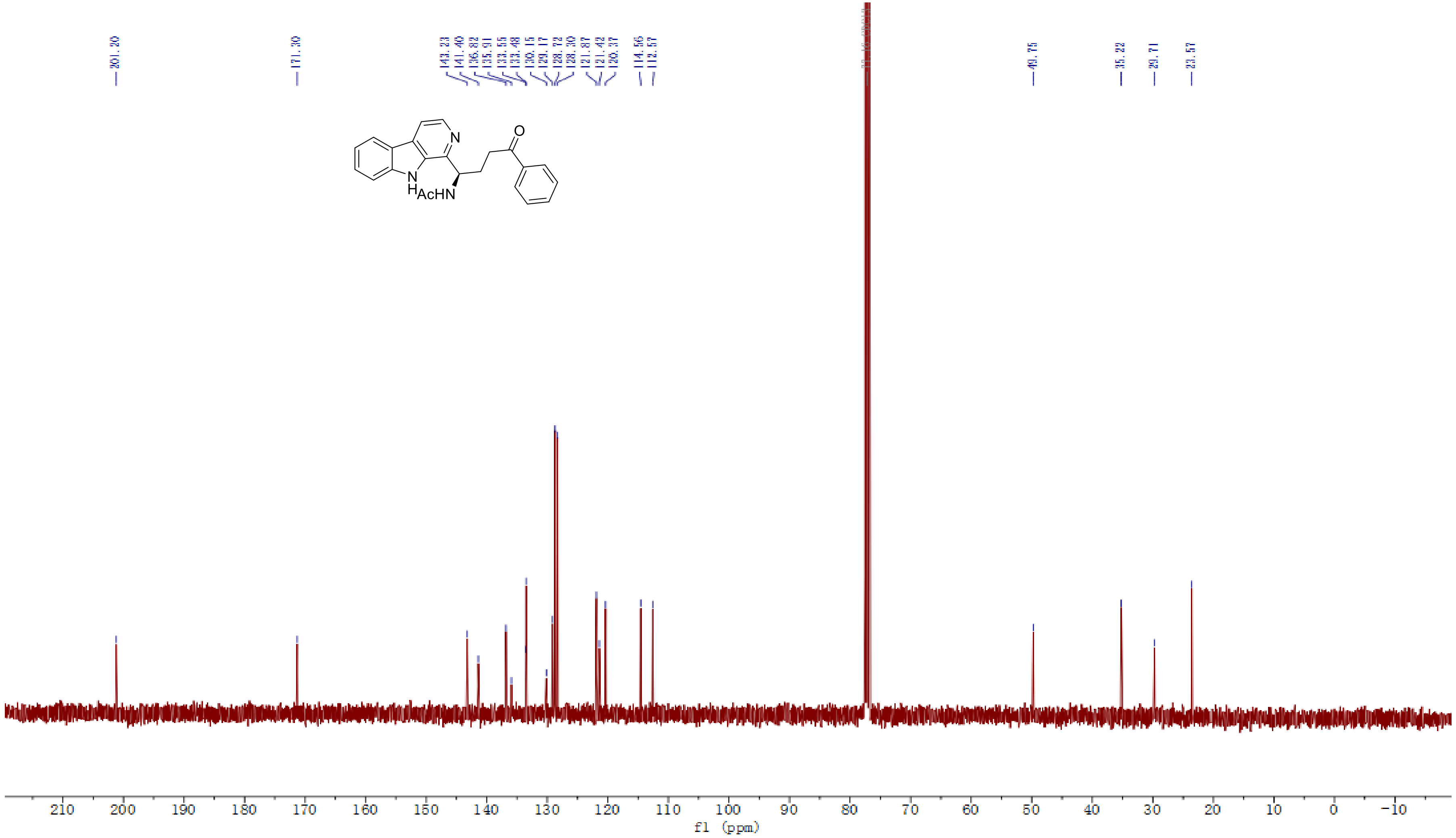

[1]H NMR (400 MHz, $CDCl_3$) (*R*)-4-acetamido-*N*,*N*-dimethyl-4-(9*H*-pyrido[3,4-*b*]indol-1-yl)butanamide (**4o**)

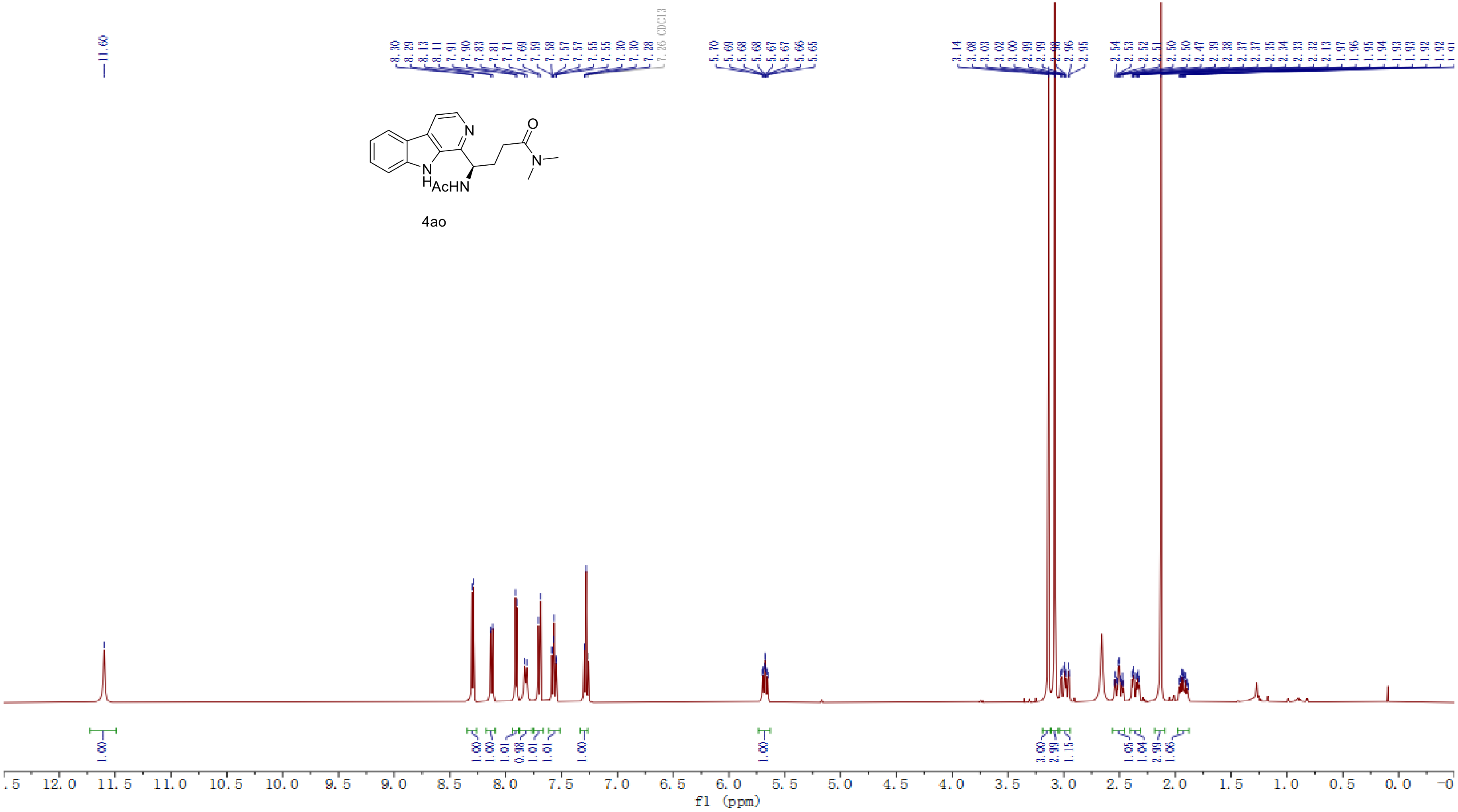

[13]C NMR (101 MHz, $CDCl_3$) (*R*)-4-acetamido-*N,N*-dimethyl-4-(9*H*-pyrido[3,4-*b*]indol-1-yl)butanamide (**4o**)

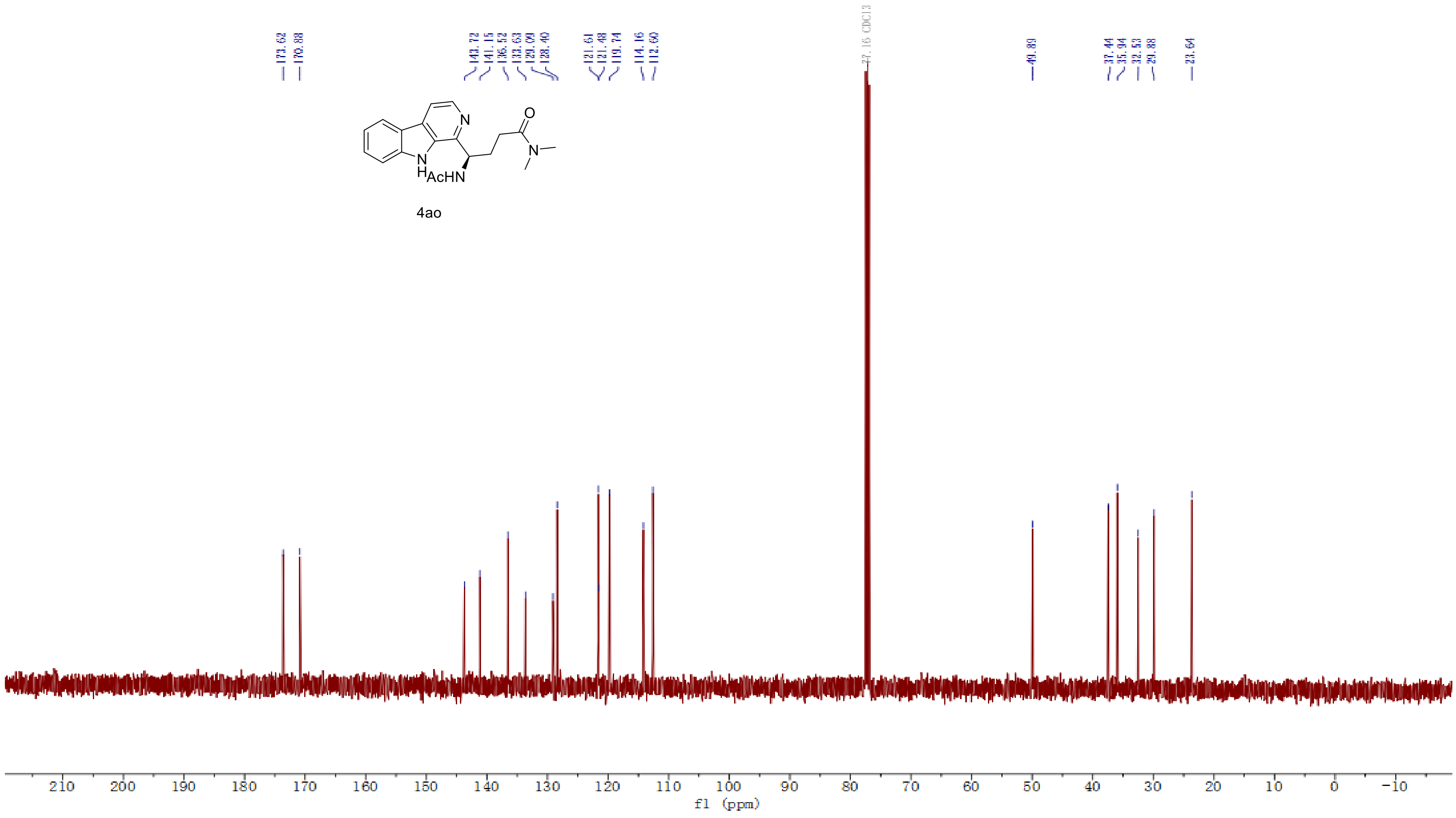

$^1$H NMR (400 MHz, $CDCl_3$) (*R*)-dimethyl-2-(2-acetamido-2-(9*H*-pyrido[3,4-*b*]indol-1-yl)ethyl)malonate (**4p**)

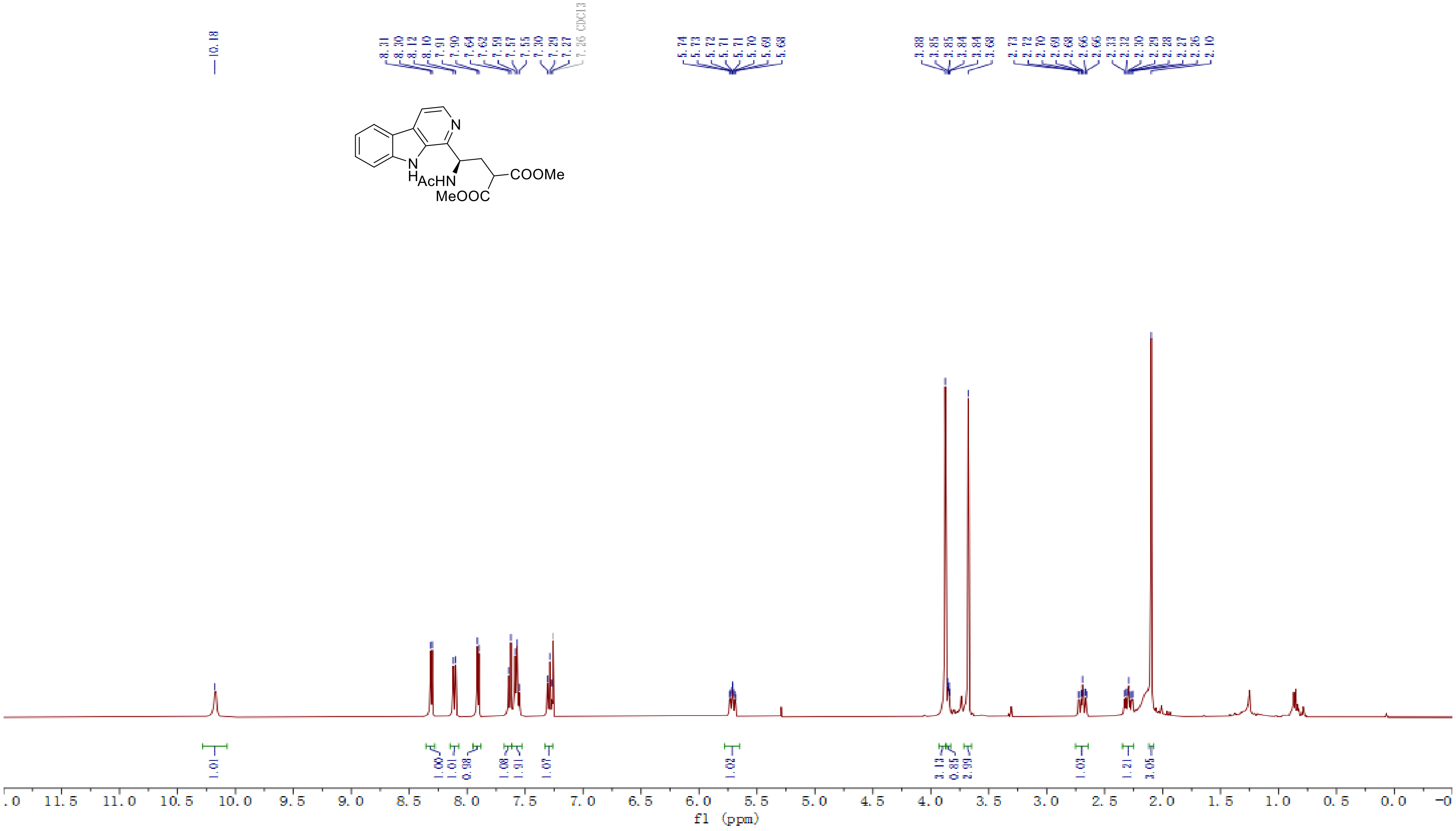

$^{13}C$ NMR (101 MHz, $CDCl_3$) (*R*)-dimethyl-2-(2-acetamido-2-(9*H*-pyrido[3,4-*b*]indol-1-yl)ethyl)malonate (**4p**)

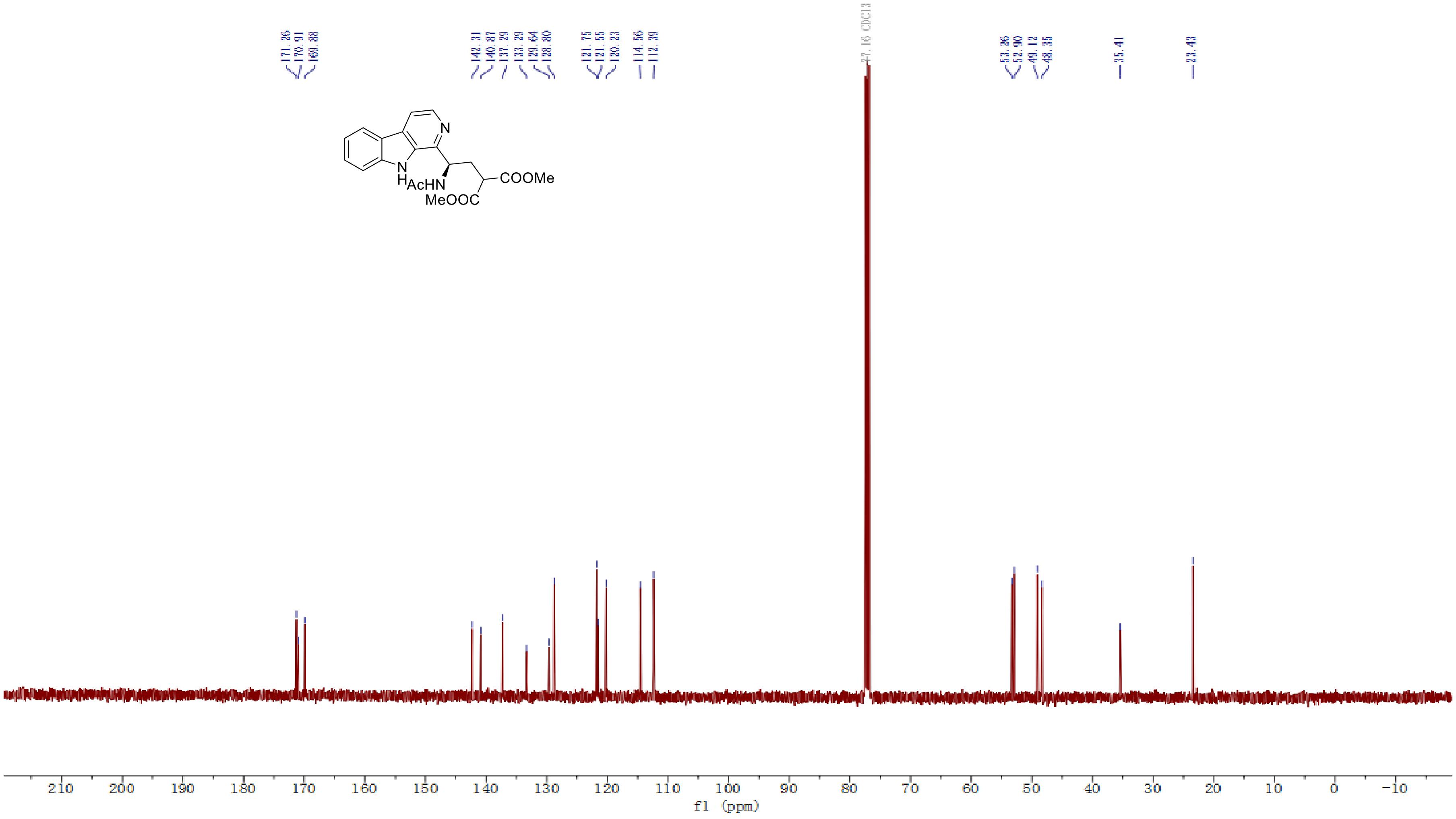

$^1$H NMR(400 MHz, $CDCl_3$) (*R*)-tert-butyl-4-acetamido-4-(5-methyl-9*H*-pyrido[3,4-*b*]indol-1-yl)butanoate (**5b**)

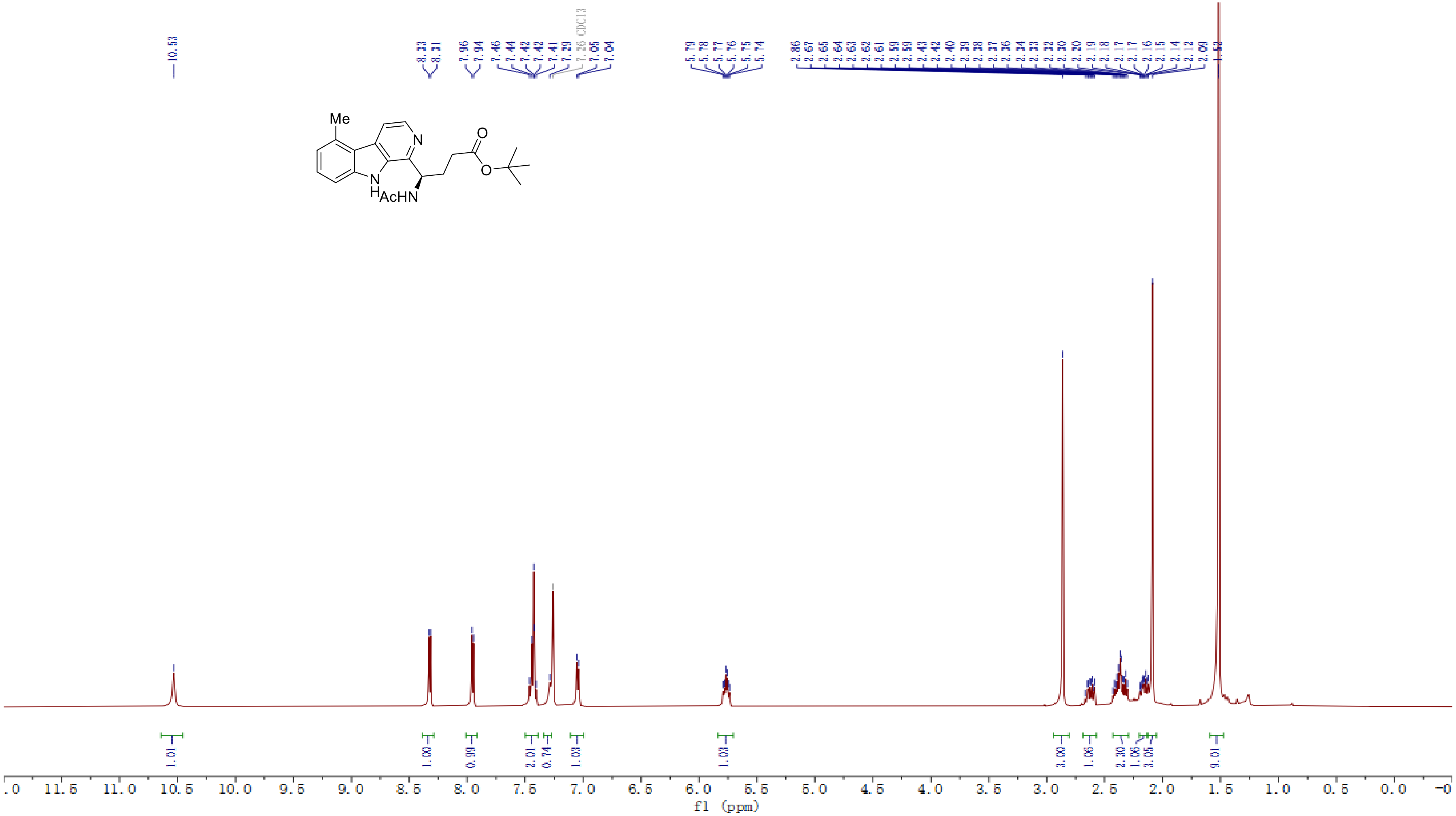

$^{13}C$ NMR(101 MHz, $CDCl_3$) (*R*)-tert-butyl-4-acetamido-4-(5-methyl-9*H*-pyrido[3,4-*b*]indol-1-yl)butanoate (**5b**)

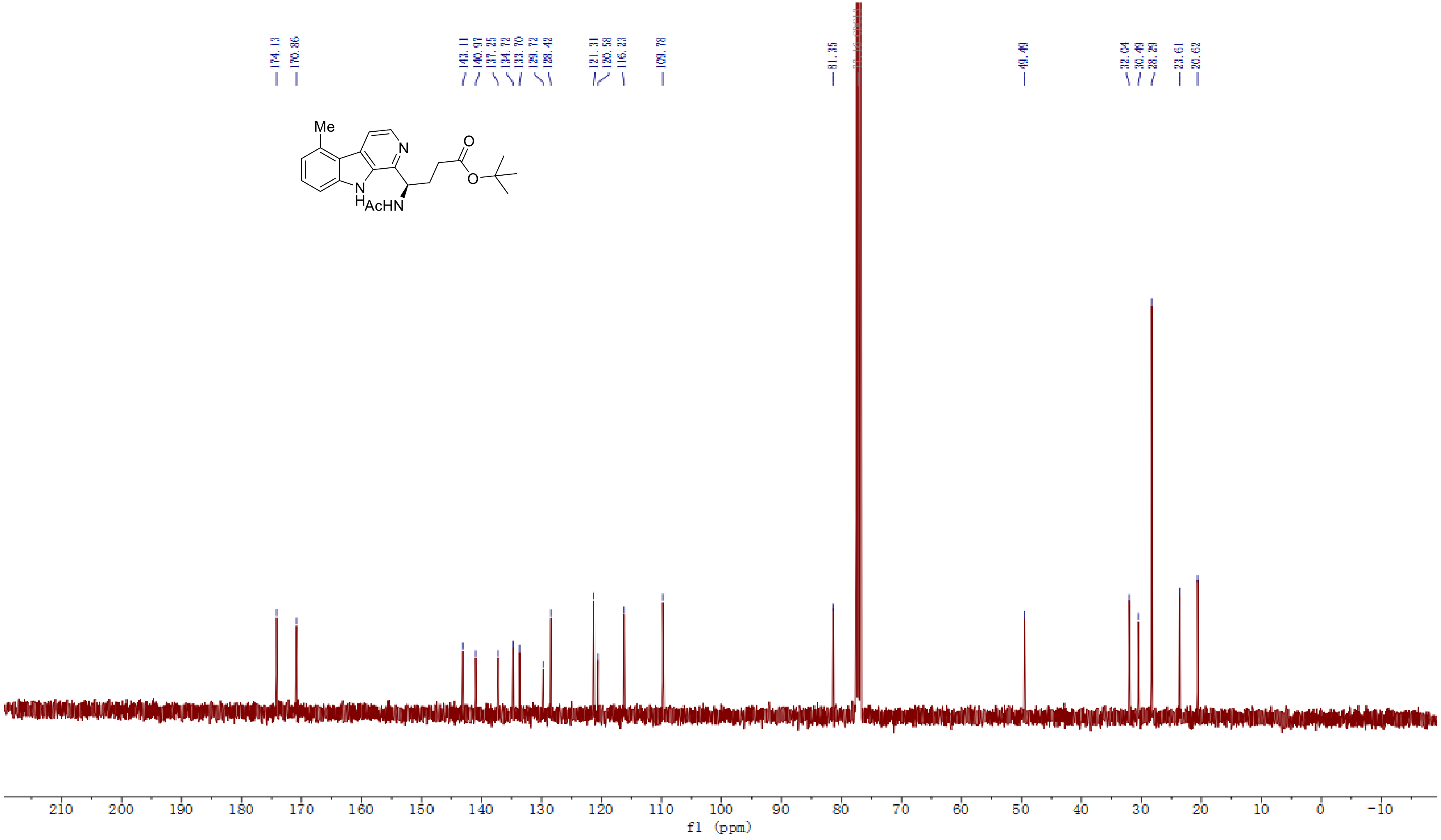

[1]H NMR (400 MHz, CDCl3) (*R*)-tert-butyl-4-acetamido-4-(6-methyl-9*H*-pyrido[3,4-*b*]indol-1-yl)butanoat (**5c**)

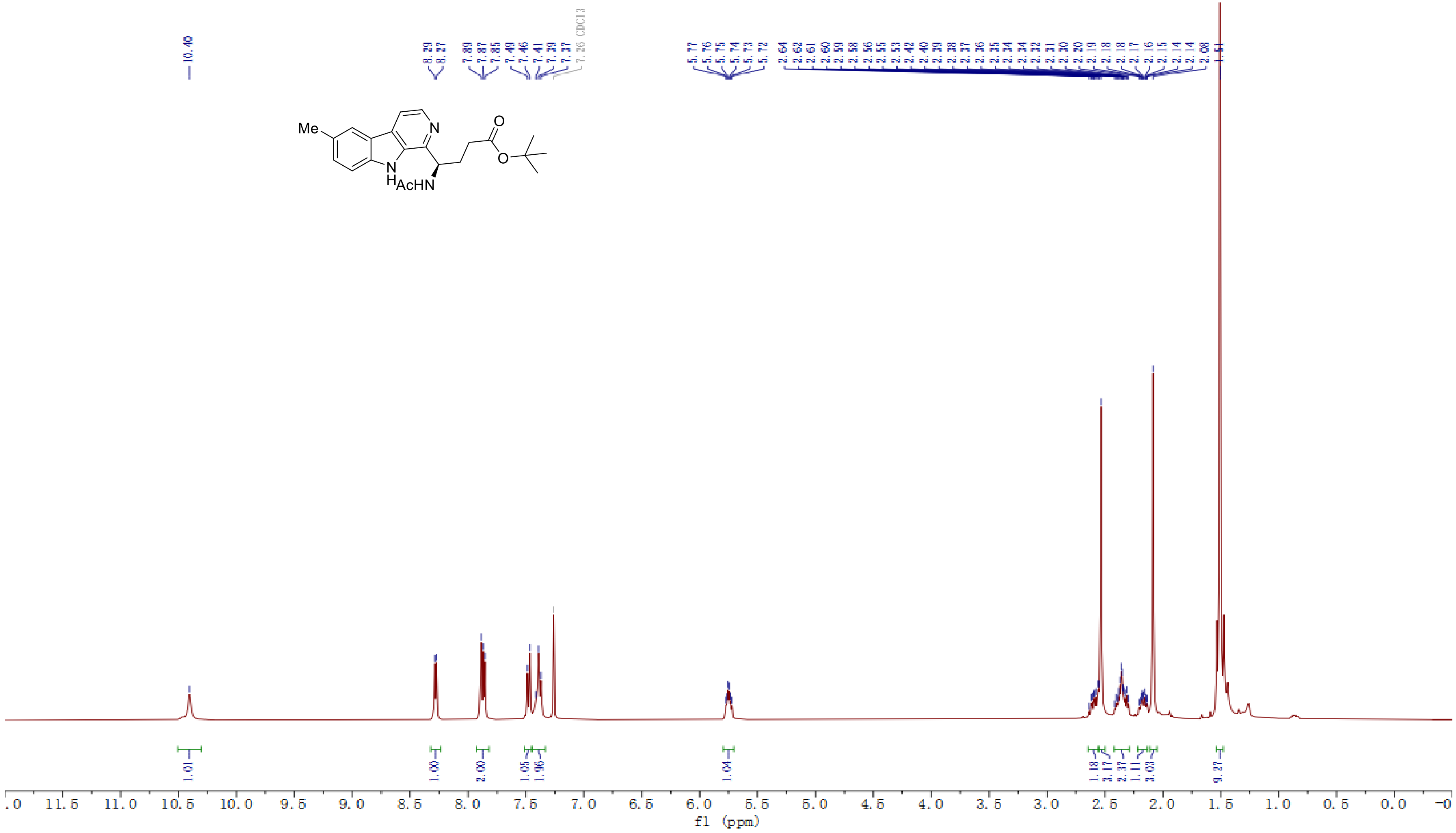

$^{13}$C NMR (101 MHz, $CDCl_3$) (*R*)-tert-butyl-4-acetamido-4-(6-methyl-9*H*-pyrido[3,4-*b*]indol-1-yl)butanoat (**5c**)

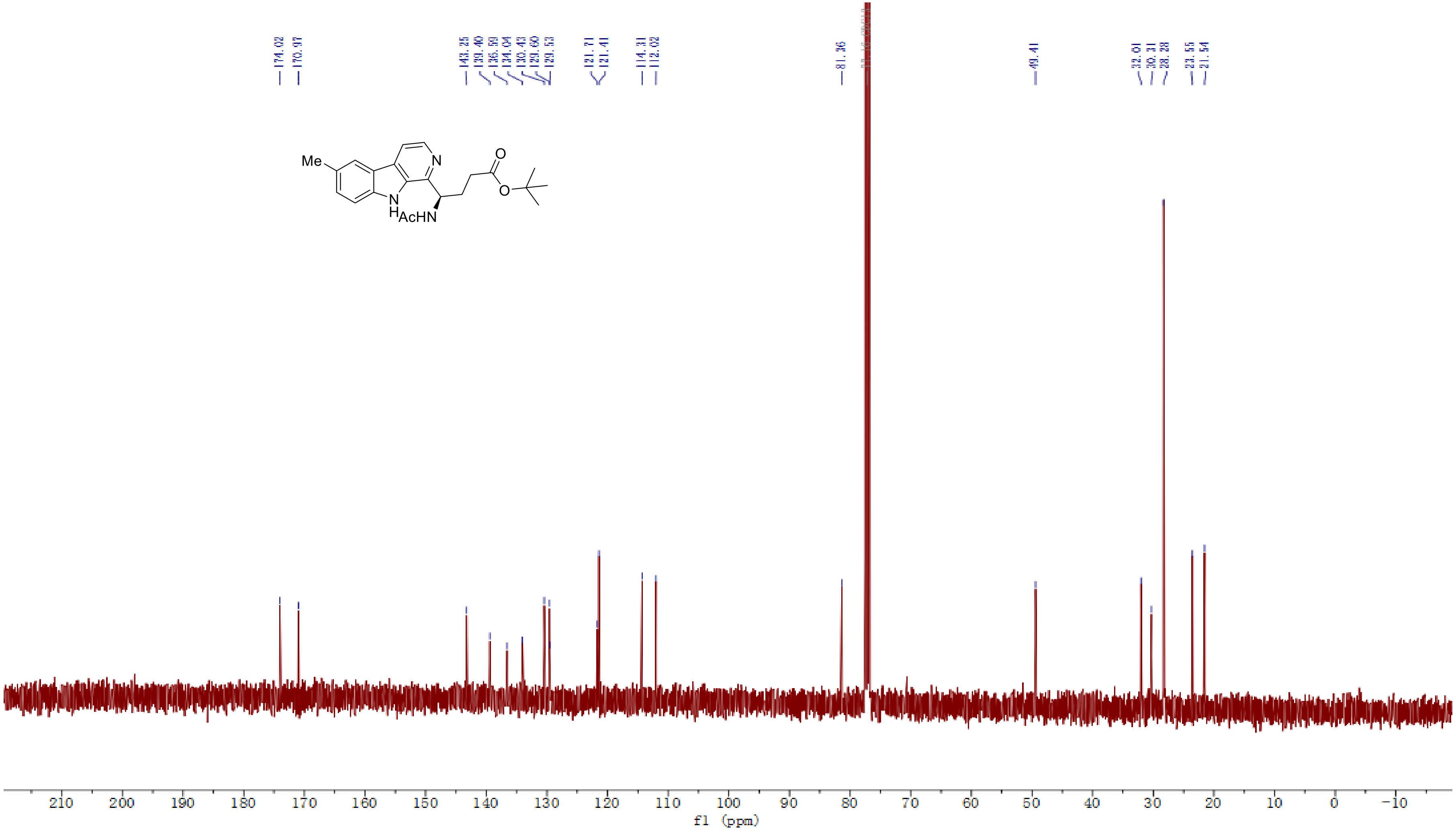

$^1$H NMR(400 MHz, $CDCl_3$) (*R*)-tert-butyl-4-acetamido-4-(8-methyl-9*H*-pyrido[3,4-*b*]indol-1-yl)butanoate (**5d**)

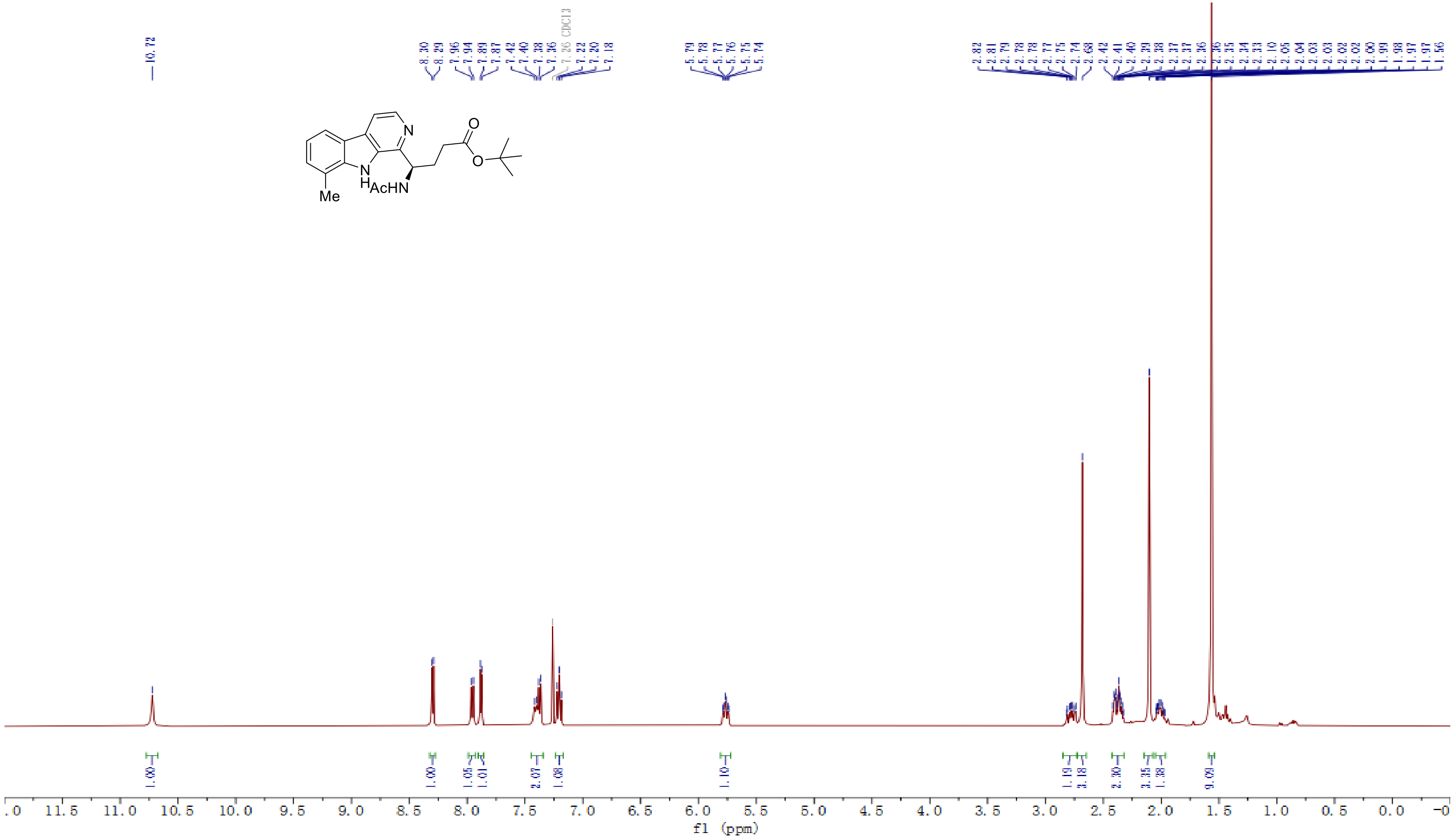

$^{13}C$ NMR(101 MHz, $CDCl_3$)(R)-tert-butyl-4-acetamido-4-(8-methyl-9H-pyrido[3,4-*b*]indol-1-yl)butanoate (**5d**)

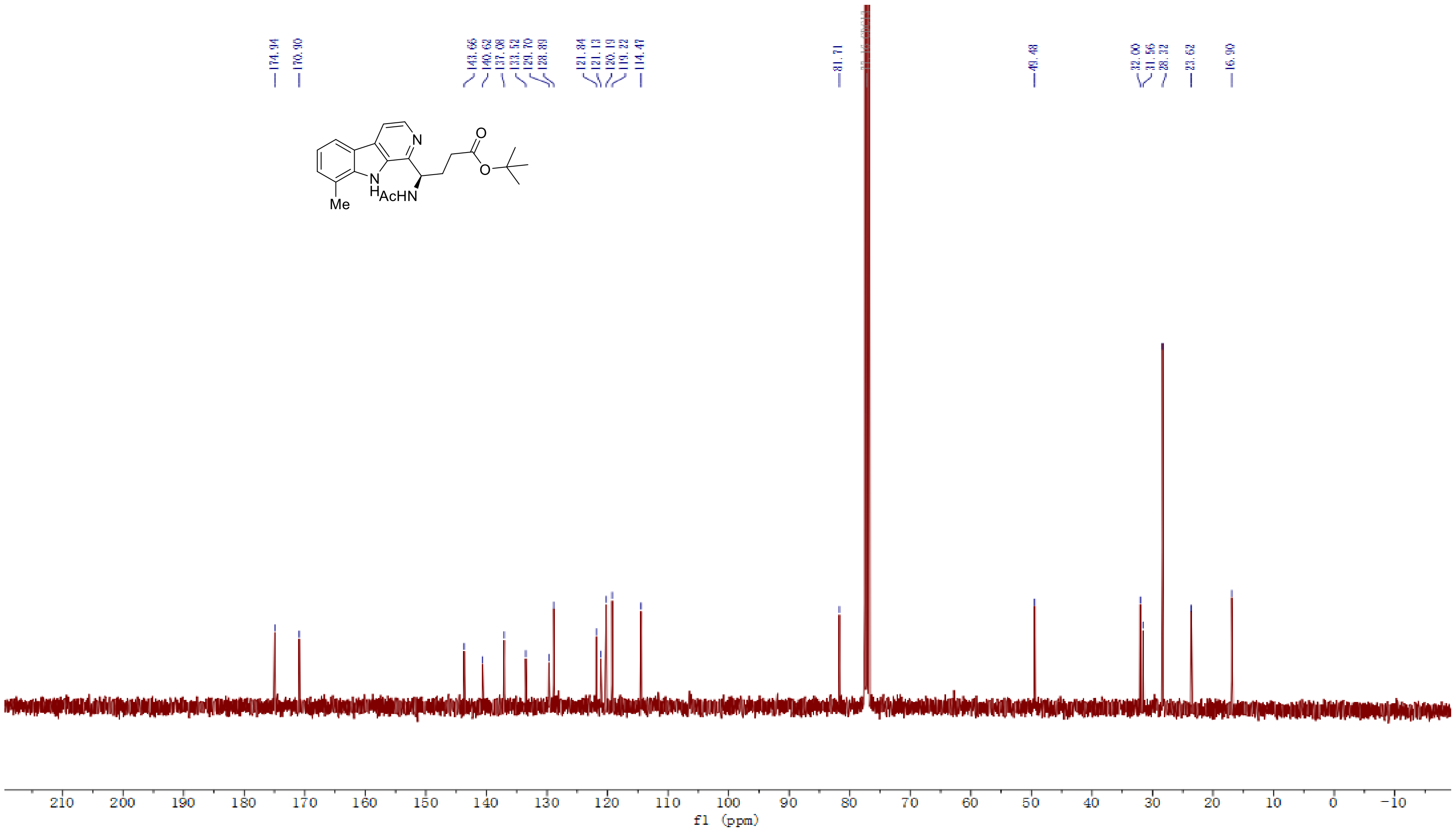

[1]H NMR(400 MHz, CDCl$_3$) (*R*)-tert-butyl-4-acetamido-4-(4-methyl-9*H*-pyrido[3,4-*b*]indol-1-yl)butanoate (**5e**)

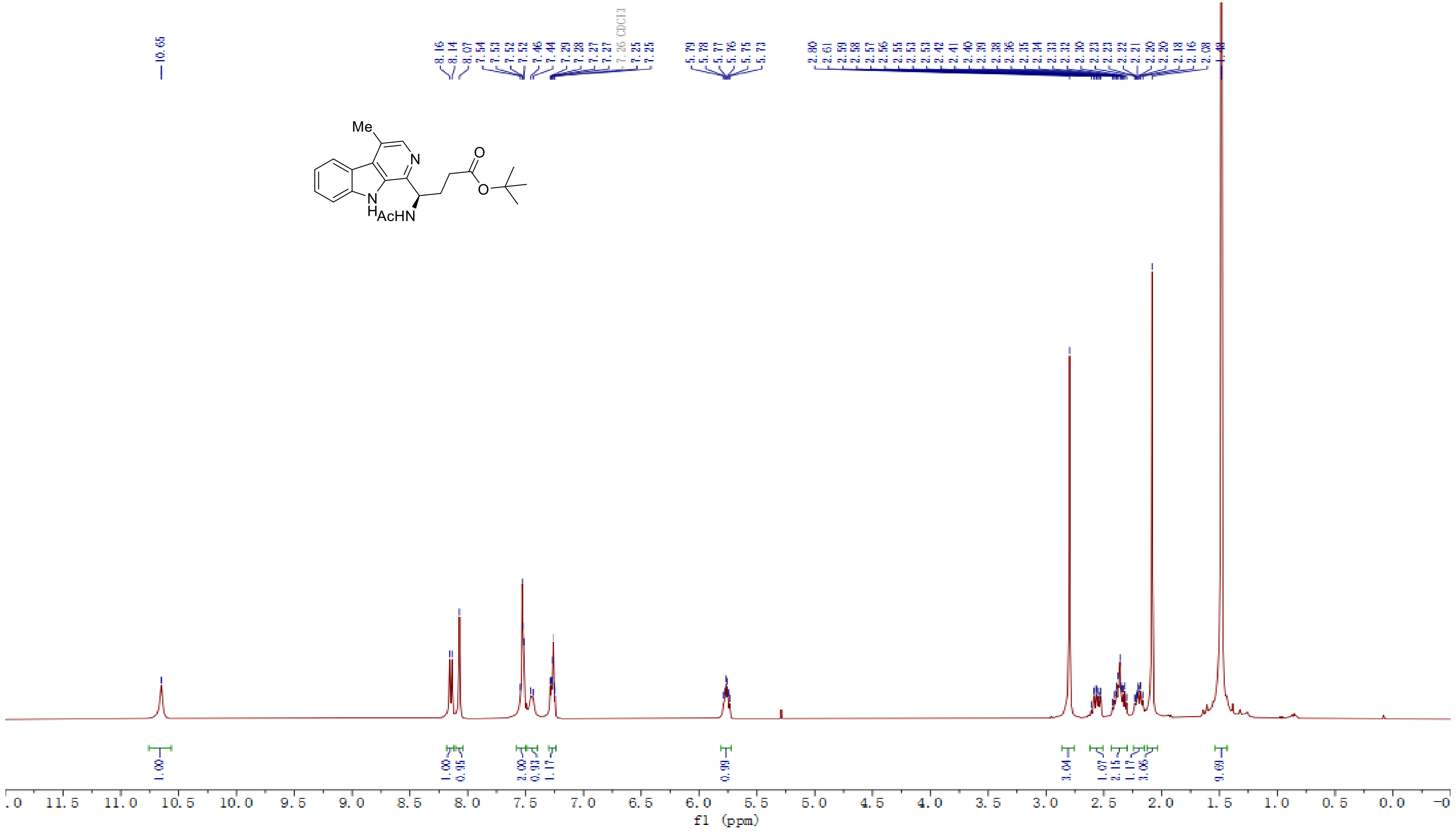

$^{13}C$ NMR(101 MHz, $CDCl_3$) (*R*)-tert-butyl-4-acetamido-4-(4-methyl-9*H*-pyrido[3,4-*b*]indol-1-yl)butanoate (**5e**)

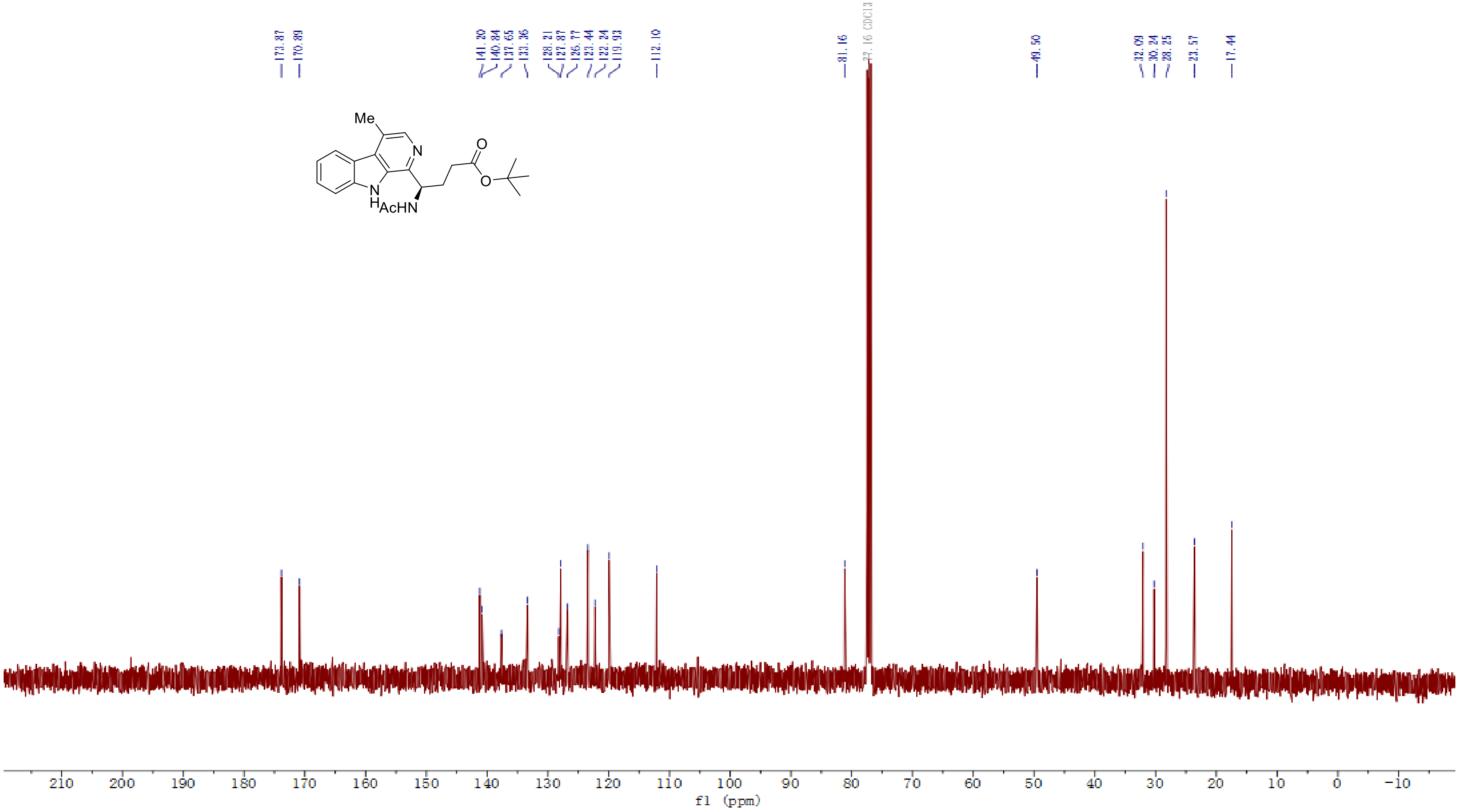

$^{1}H$ NMR (400 MHz, $CDCl_3$) (*R*)-tert-butyl-4-acetamido-4-(6-methoxy-9*H*-pyrido[3,4-*b*]indol-1-yl)butanoate (**5f**)

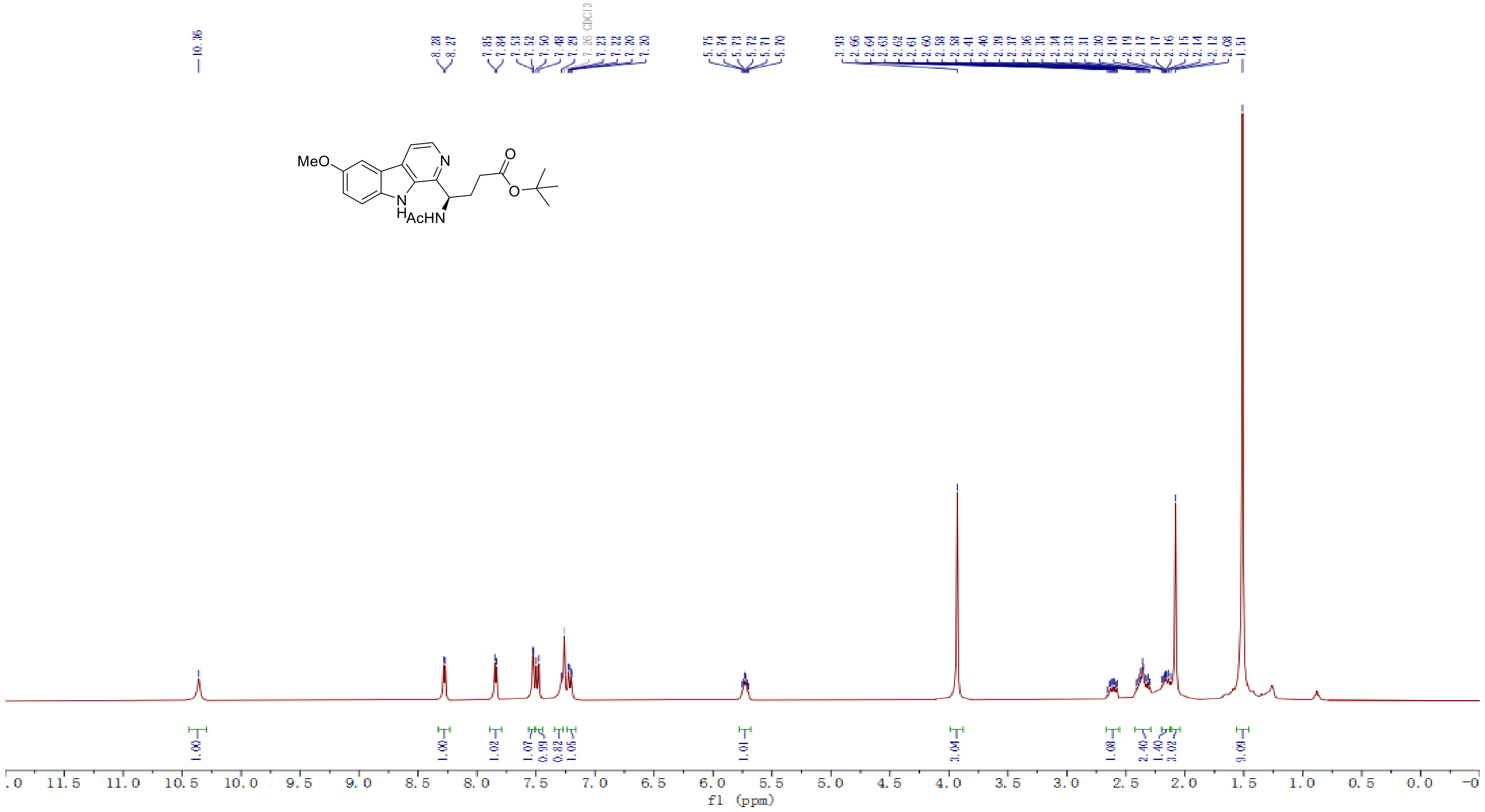

$^{13}$C NMR (101 MHz, $CDCl_3$)(R)-tert-butyl-4-acetamido-4-(6-methoxy-9H-pyrido[3,4-*b*]indol-1-yl)butanoate (**5f**)

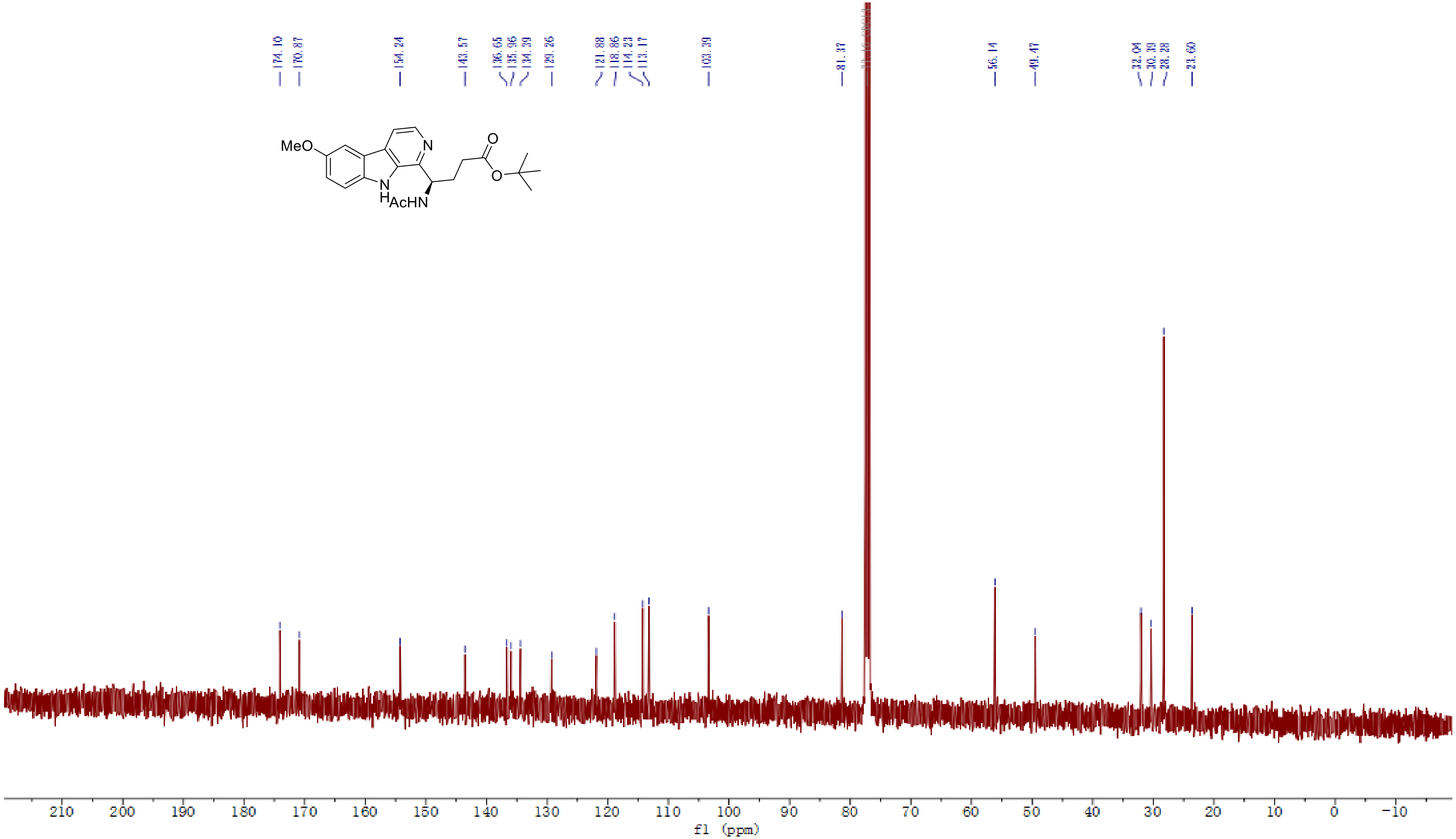

[1]H NMR (400 MHz, $CDCl_3$) (R)-tert-butyl-4-acetamido-4-(6-hydroxy-9H-pyrido[3,4-*b*]indol-1-yl)butanoate (**5g**)

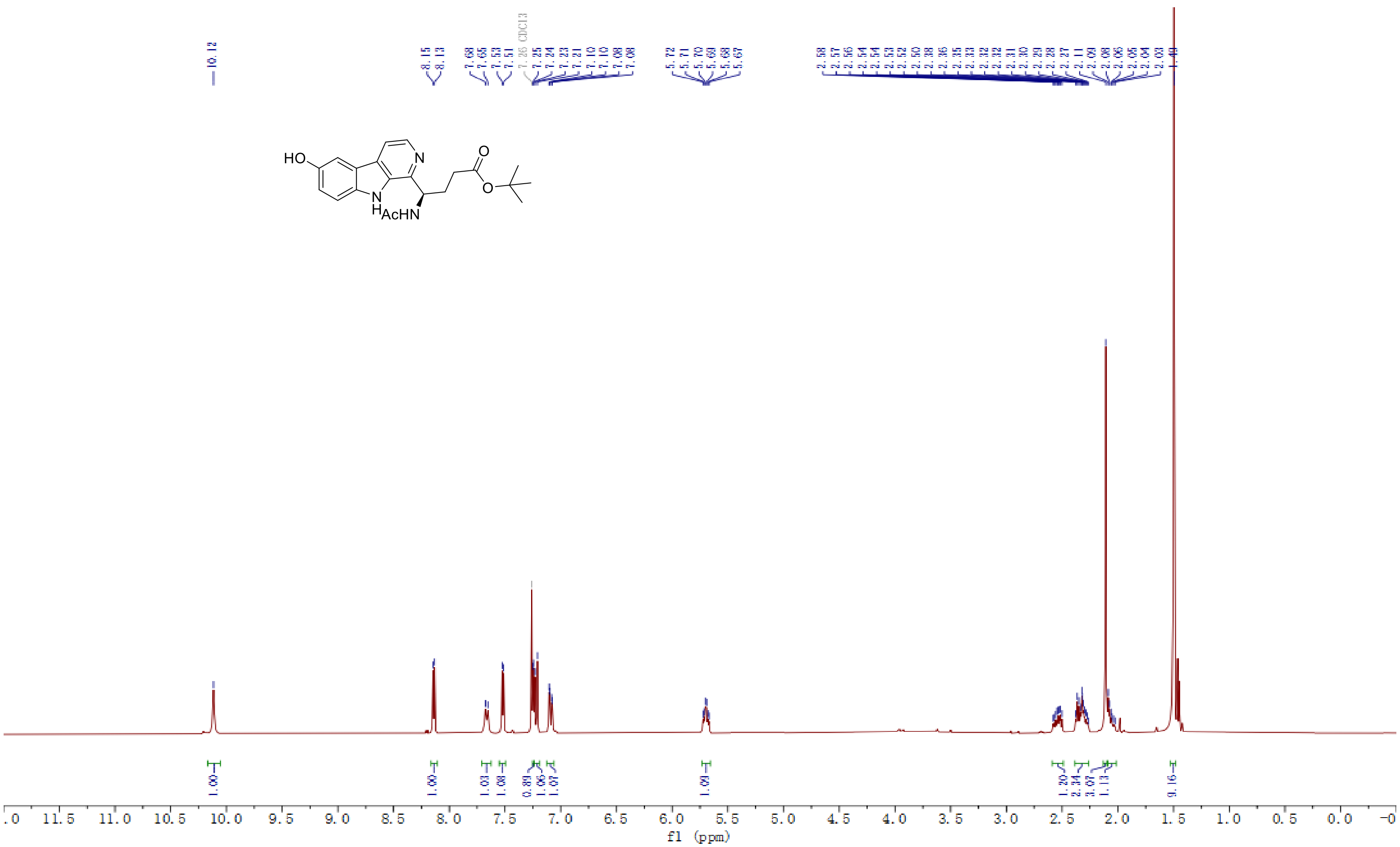

$^{13}C$ NMR (101 MHz, $CDCl_3$) (*R*)-tert-butyl-4-acetamido-4-(6-hydroxy-9*H*-pyrido[3,4-*b*]indol-1-yl)butanoate (**5g**)

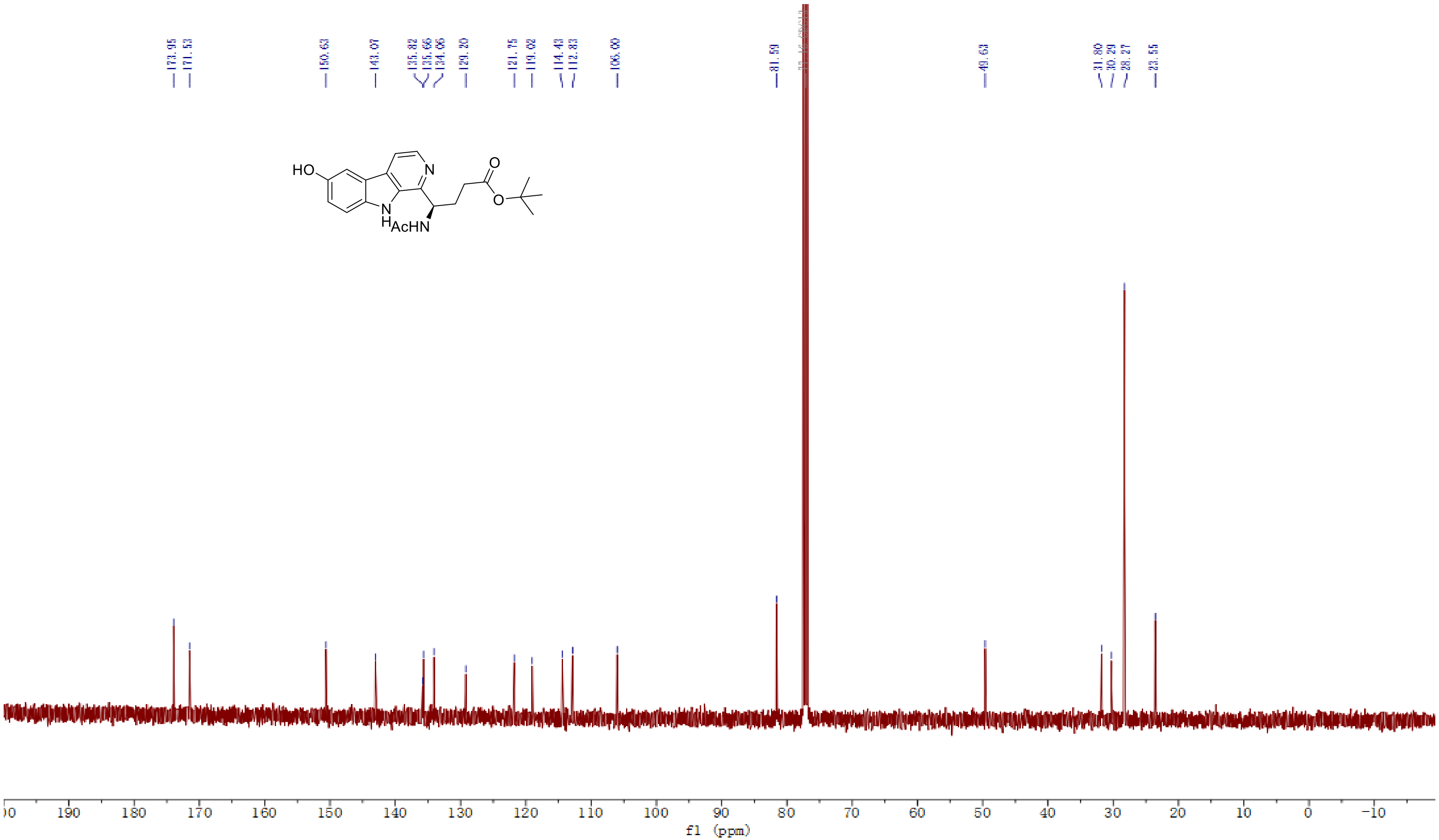

[1]H NMR (400 MHz, $CDCl_3$) (*R*)-tert-butyl-4-acetamido-4-(6-acetoxy-9*H*-pyrido[3,4-*b*]indol-1-yl)butanoate (**5h**)

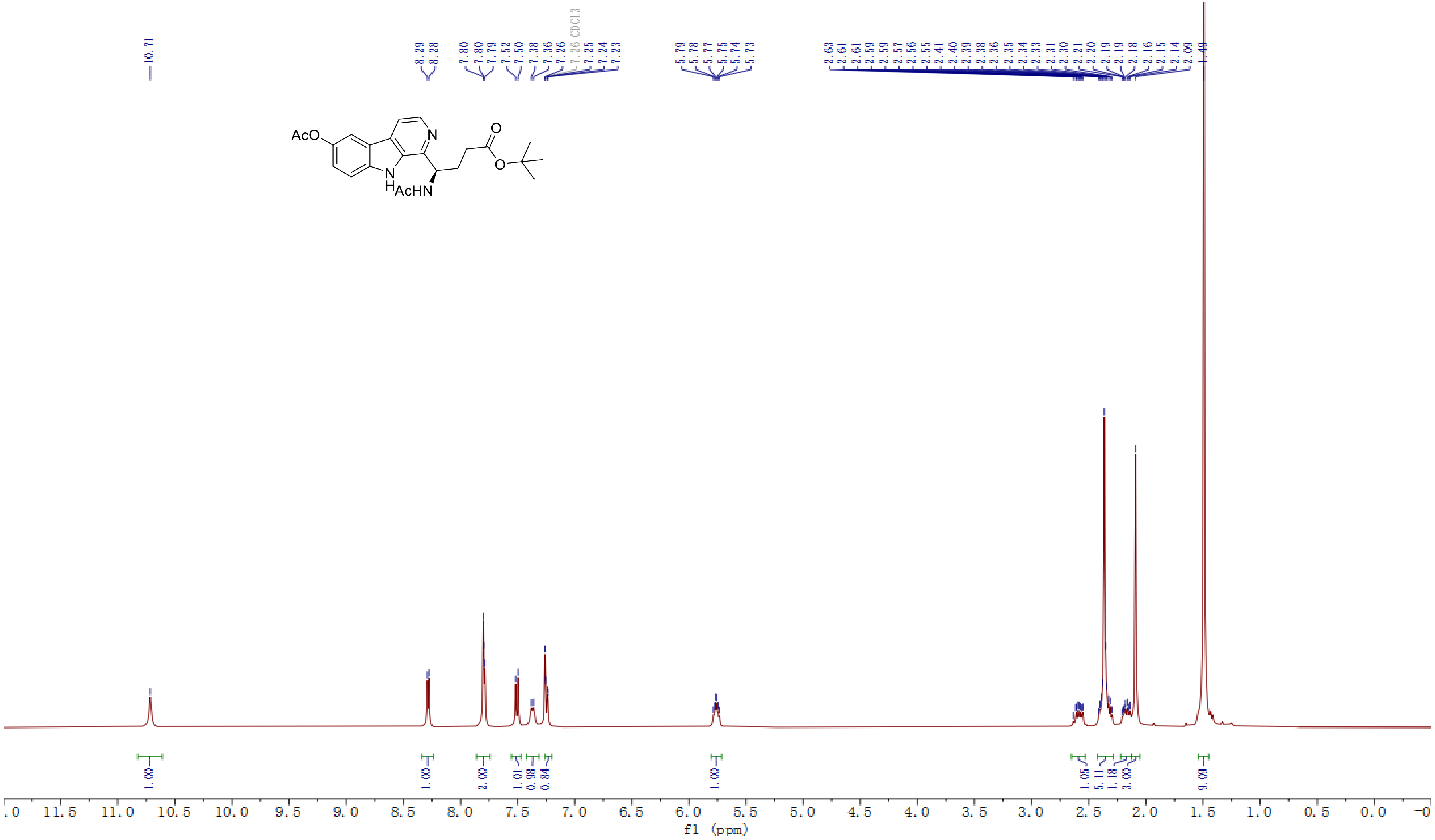

$^{13}C$ NMR (101 MHz, $CDCl_3$) (*R*)-tert-butyl-4-acetamido-4-(6-acetoxy-9*H*-pyrido[3,4-*b*]indol-1-yl)butanoate (**5h**)

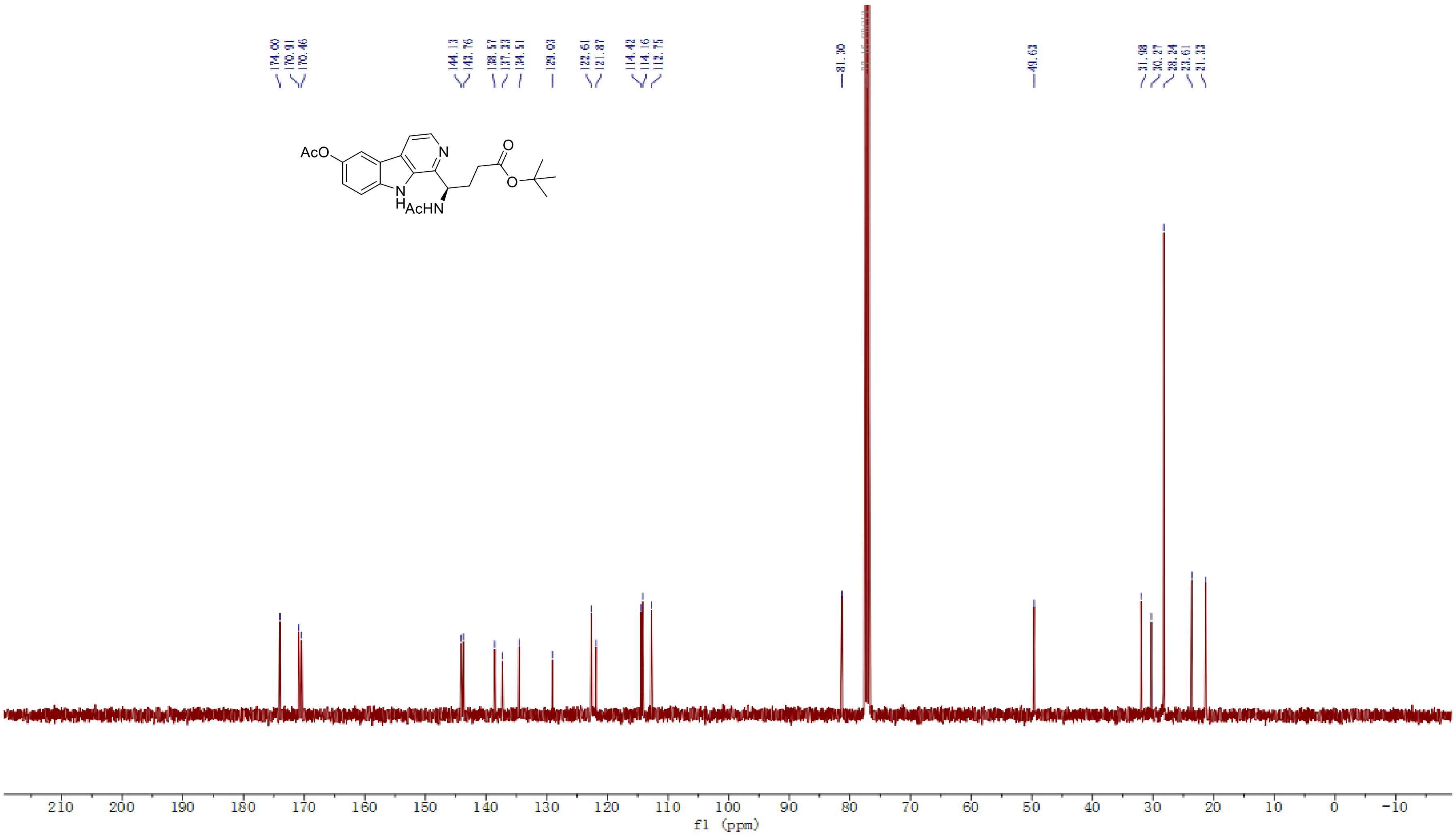

[1]H NMR(400 MHz, $CDCl_3$) (*R*)-tert-butyl-4-acetamido-4-(6-phenyl-9*H*-pyrido[3,4-*b*]indol-1-yl)butanoate (**5i**)

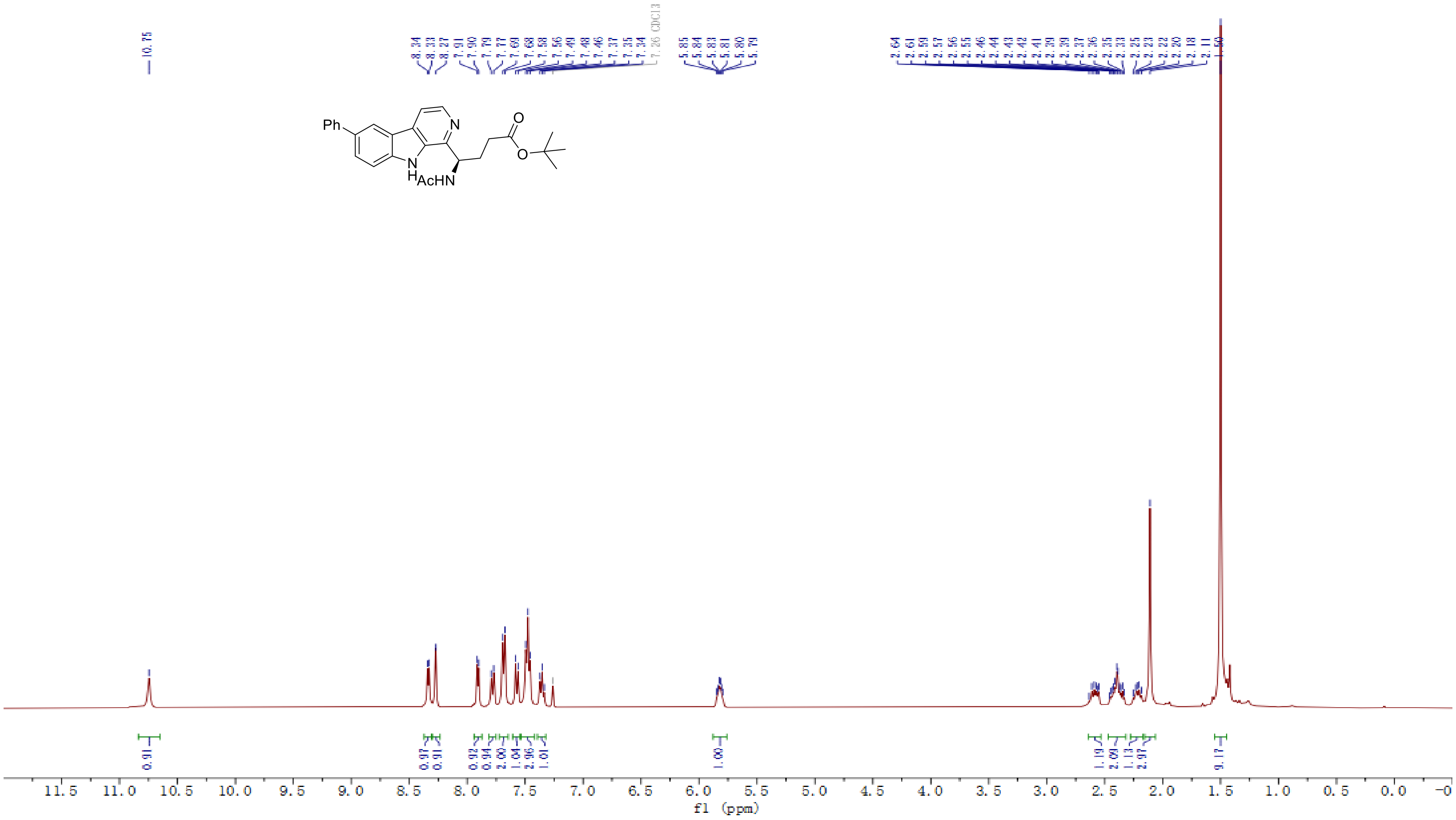

$^{13}C$ NMR(101 MHz, $CDCl_3$) (*R*)-tert-butyl-4-acetamido-4-(6-phenyl-9*H*-pyrido[3,4-*b*]indol-1-yl)butanoate (**5i**)

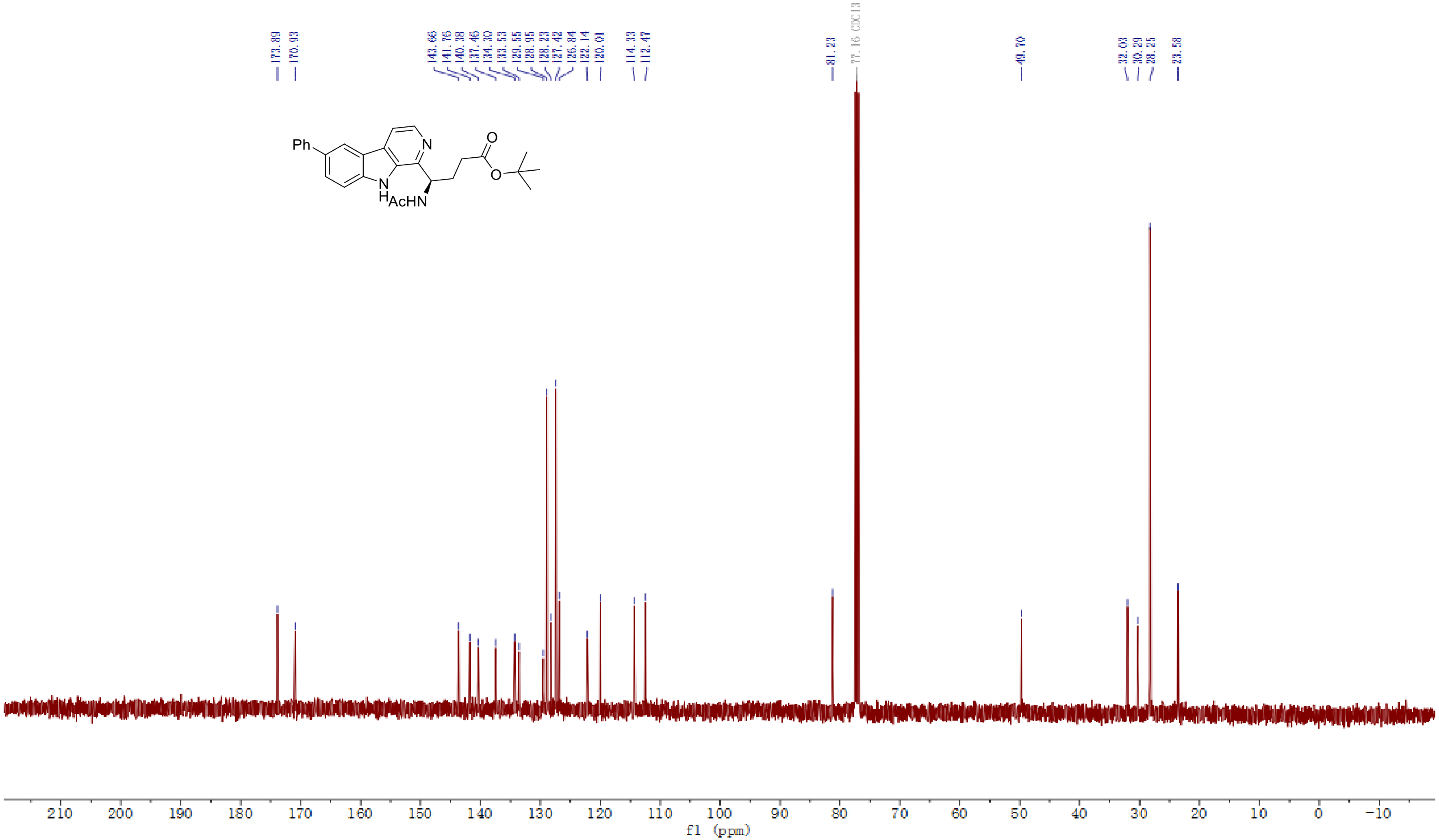

$^{1}$H NMR(400 MHz, CDCl$_3$) (*R*)-tert-butyl-4-acetamido-4-(6-fluoro-9*H*-pyrido[3,4-*b*]indol-1-yl)butanoate (**5j**)

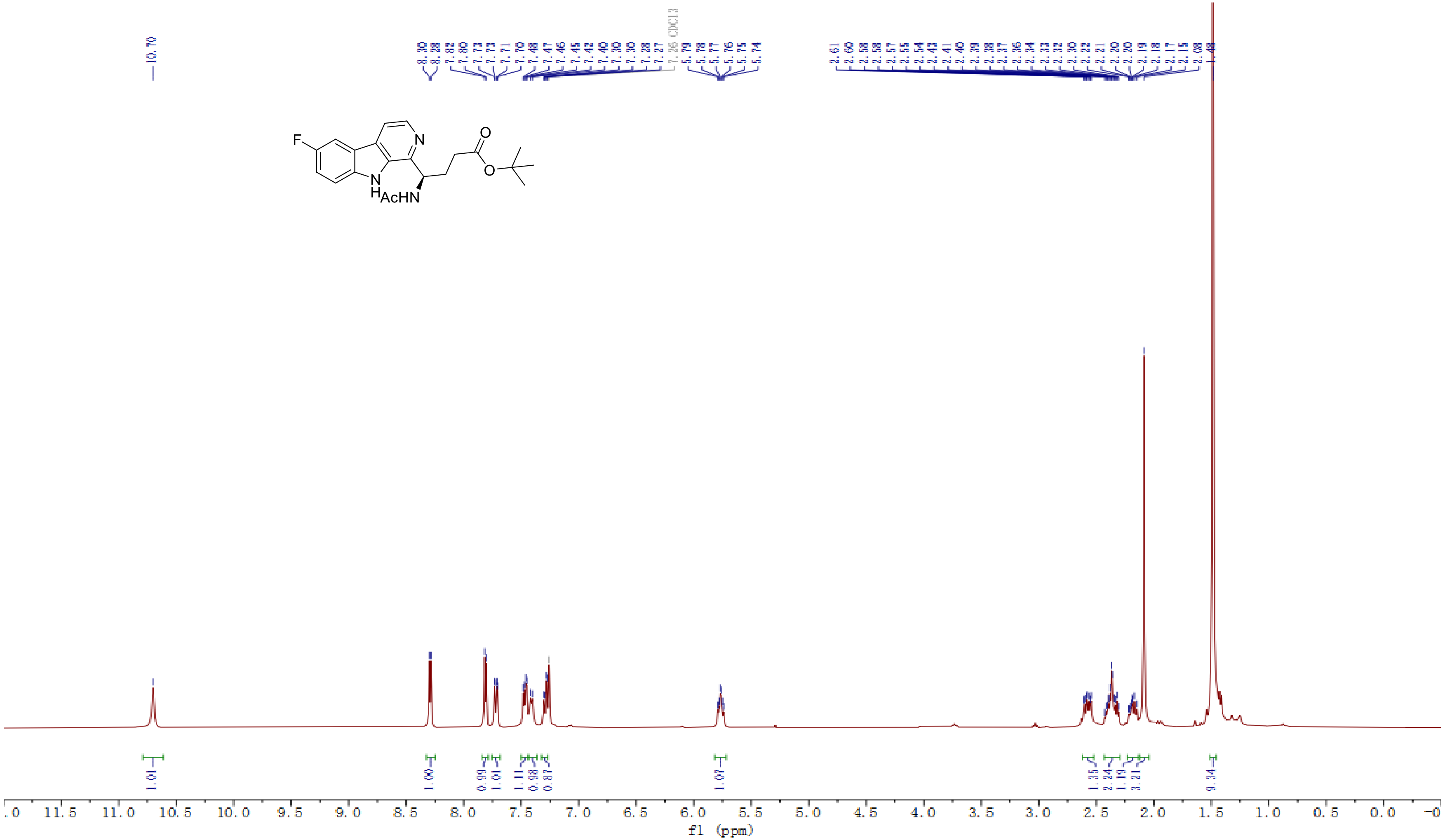

$^{13}C$ NMR(101 MHz, $CDCl_3$) (*R*)-tert-butyl-4-acetamido-4-(6-fluoro-9*H*-pyrido[3,4-*b*]indol-1-yl)butanoate (**5j**)

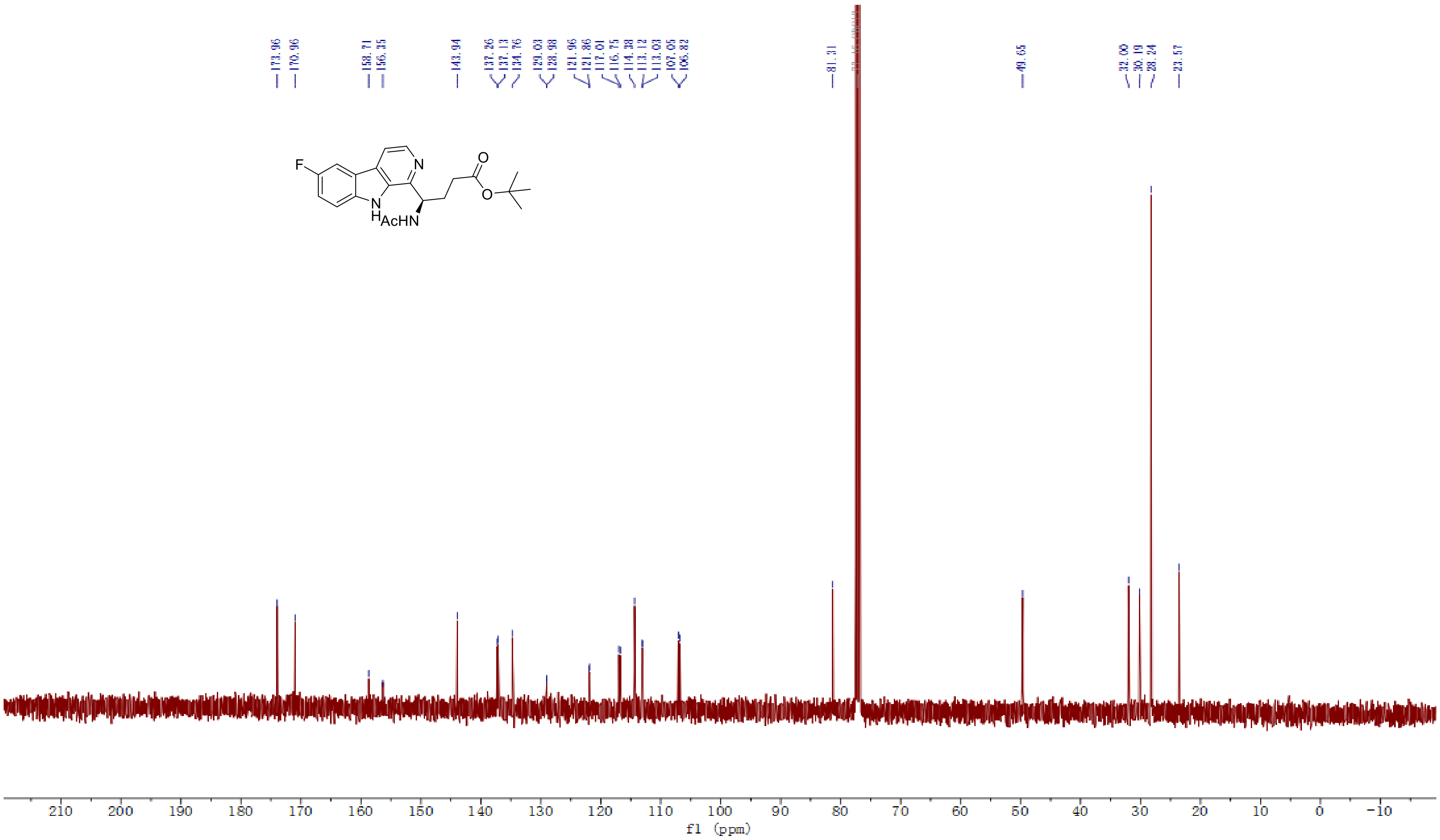

$^{19}$F NMR (376 MHz, CDCl$_3$) (*R*)-tert-butyl-4-acetamido-4-(6-fluoro-9*H*-pyrido[3,4-*b*]indol-1-yl)butanoate (**5j**)

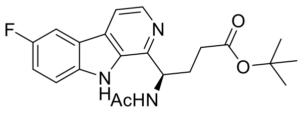


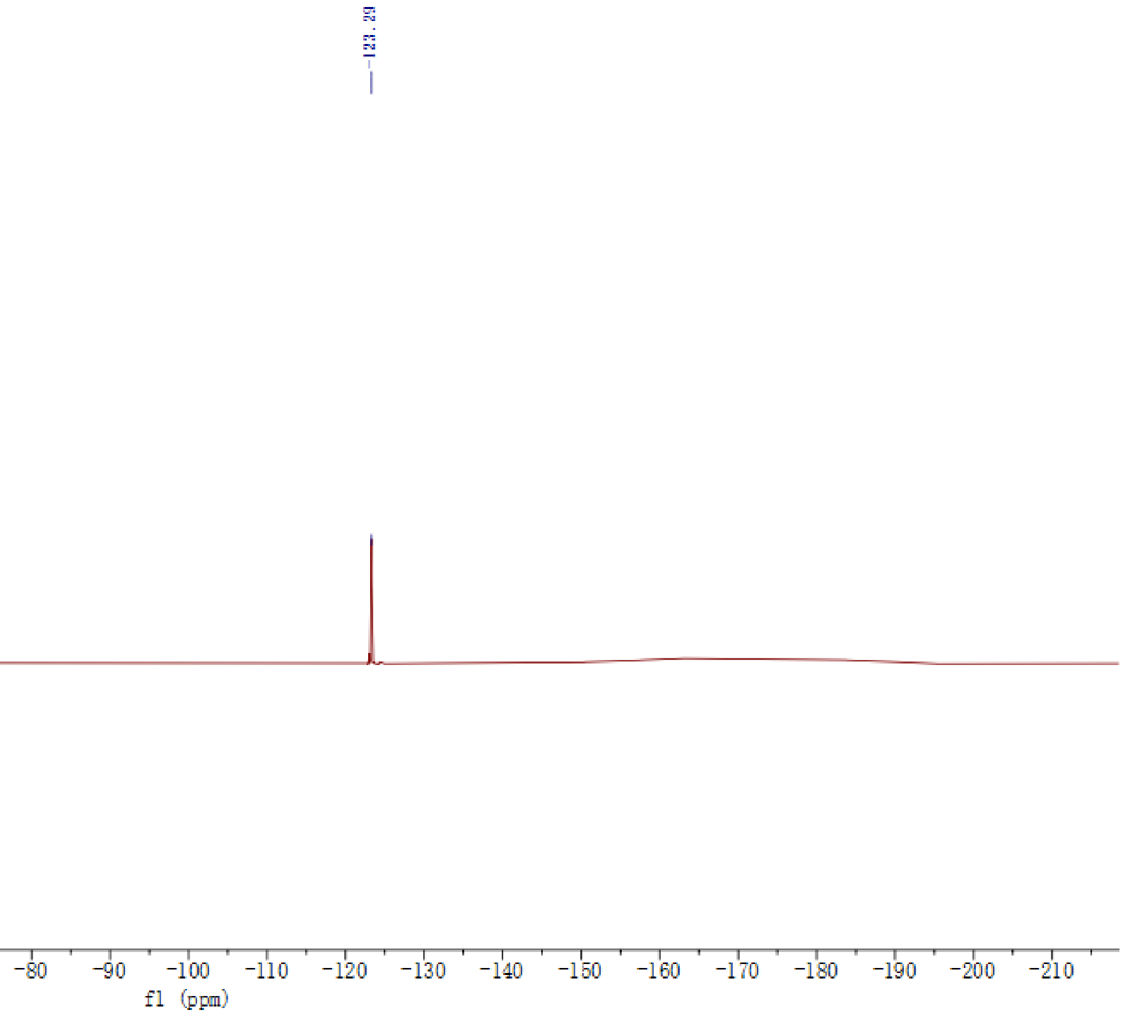

[1]H NMR(400 MHz, $CDCl_3$) (*R*)-tert-butyl-4-acetamido-4-(6-chloro-9*H*-pyrido[3,4-*b*]indol-1-yl)butanoate (**5k**)

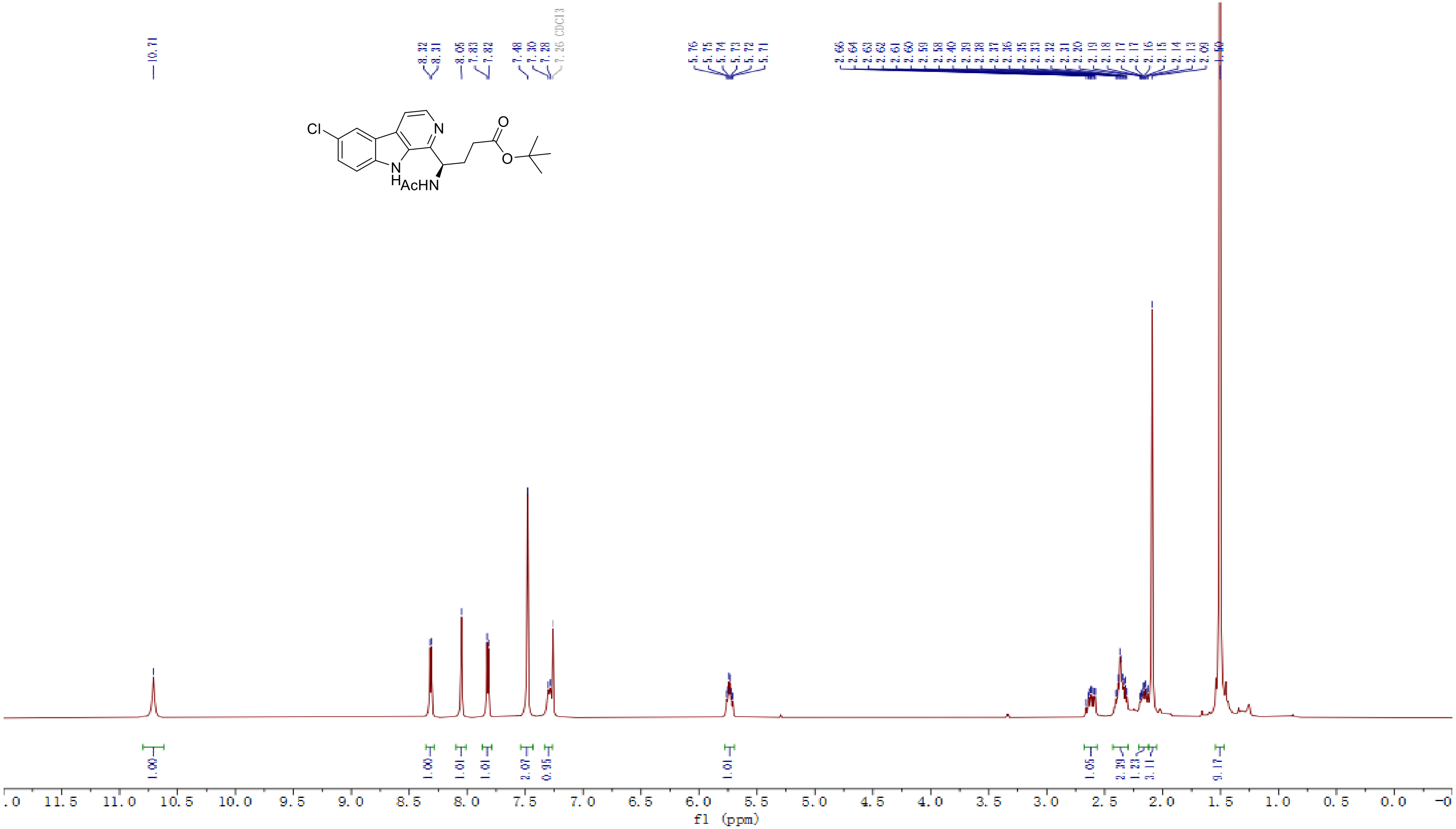

$^{13}C$ NMR(101 MHz, $CDCl_3$) (*R*)-tert-butyl-4-acetamido-4-(6-chloro-9*H*-pyrido[3,4-*b*]indol-1-yl)butanoate (**5k**)

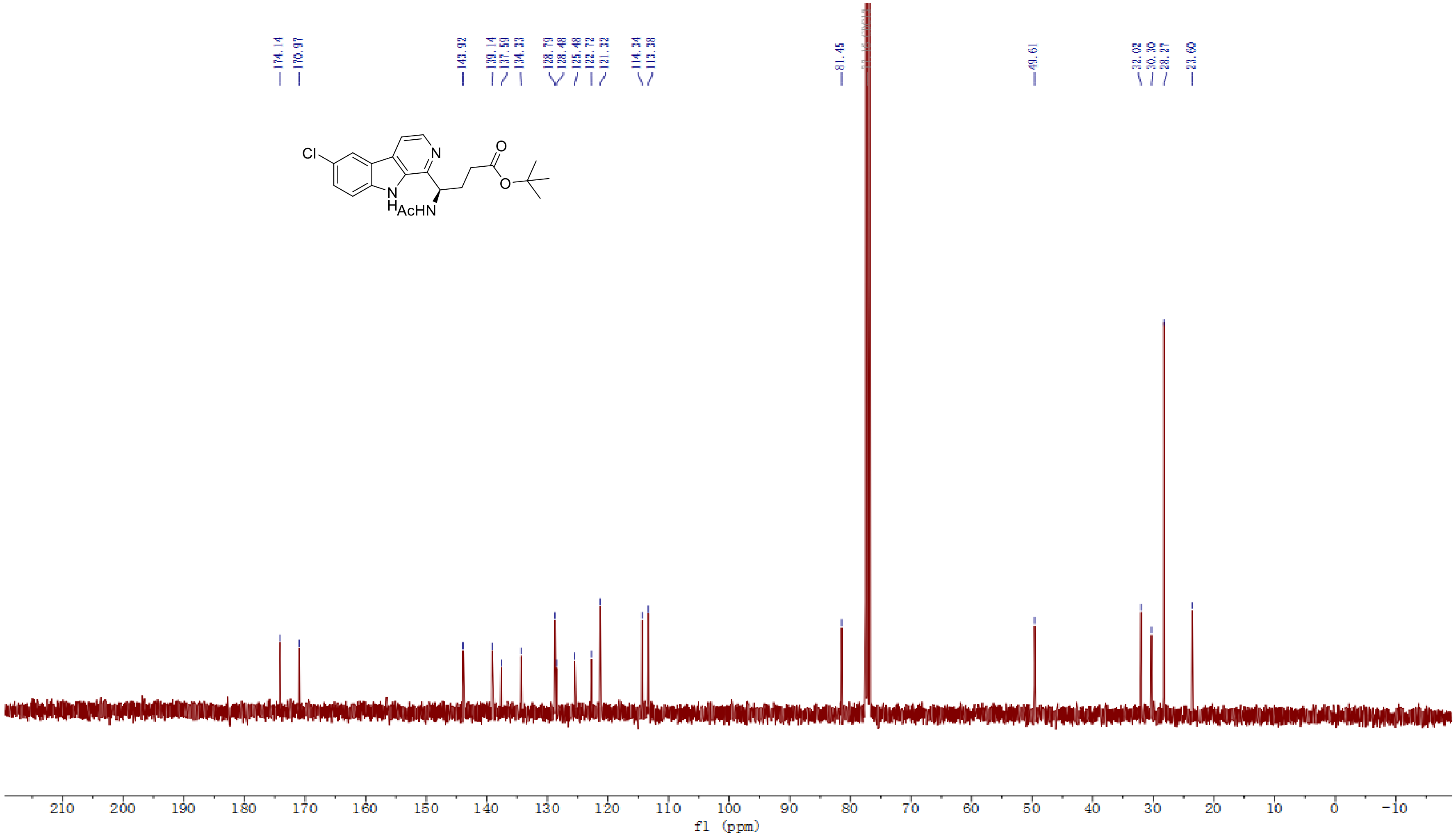

[1]H NMR(400 MHz, CDCl3) (*R*)-tert-butyl-4-acetamido-4-(6-bromo-9*H*-pyrido[3,4-*b*]indol-1-yl)butanoate (**5l**)

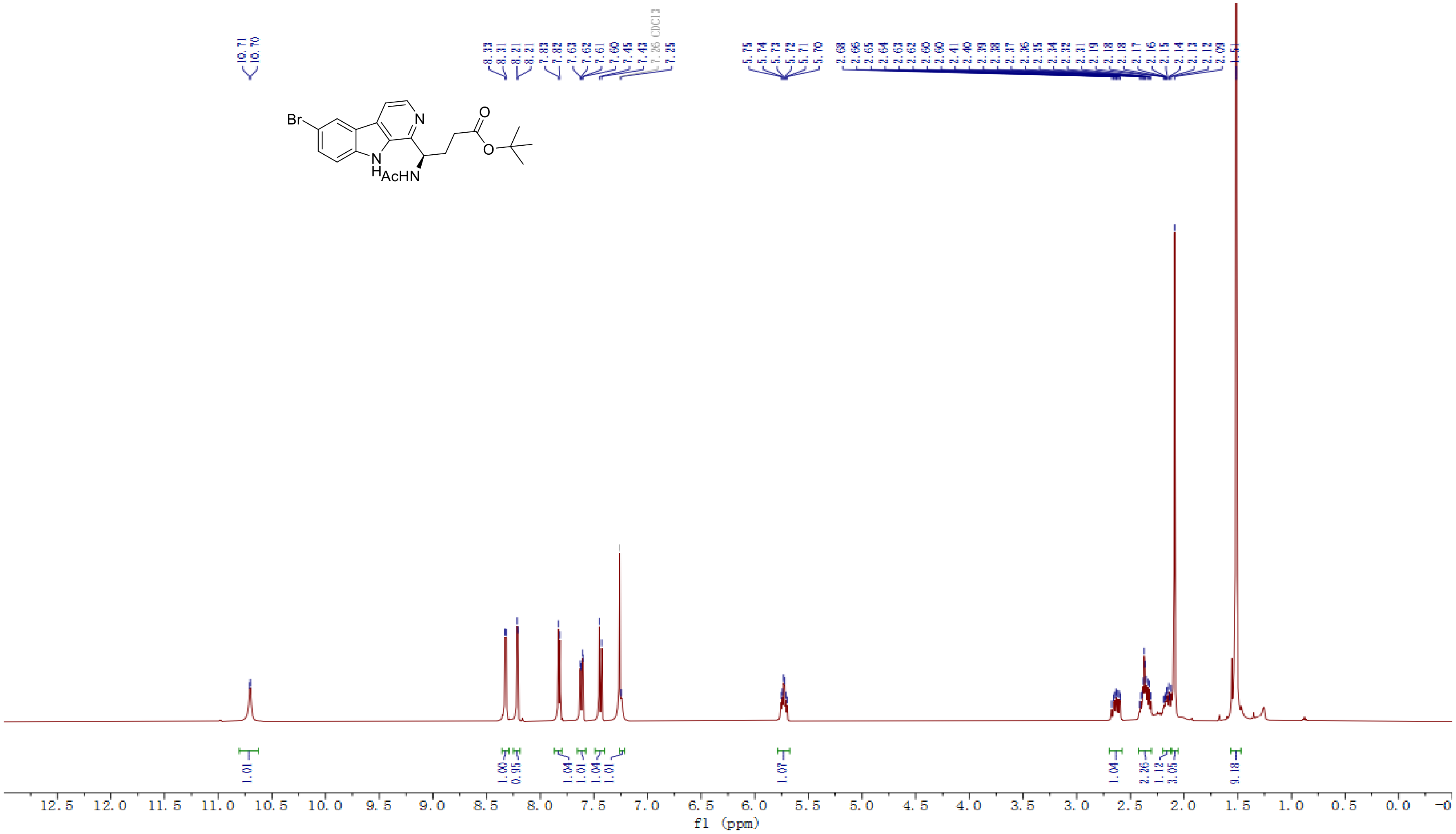

$^{13}C$ NMR(101 MHz, $CDCl_3$) (R)-tert-butyl-4-acetamido-4-(6-bromo-9*H*-pyrido[3,4-*b*]indol-1-yl)butanoate (**5l**)

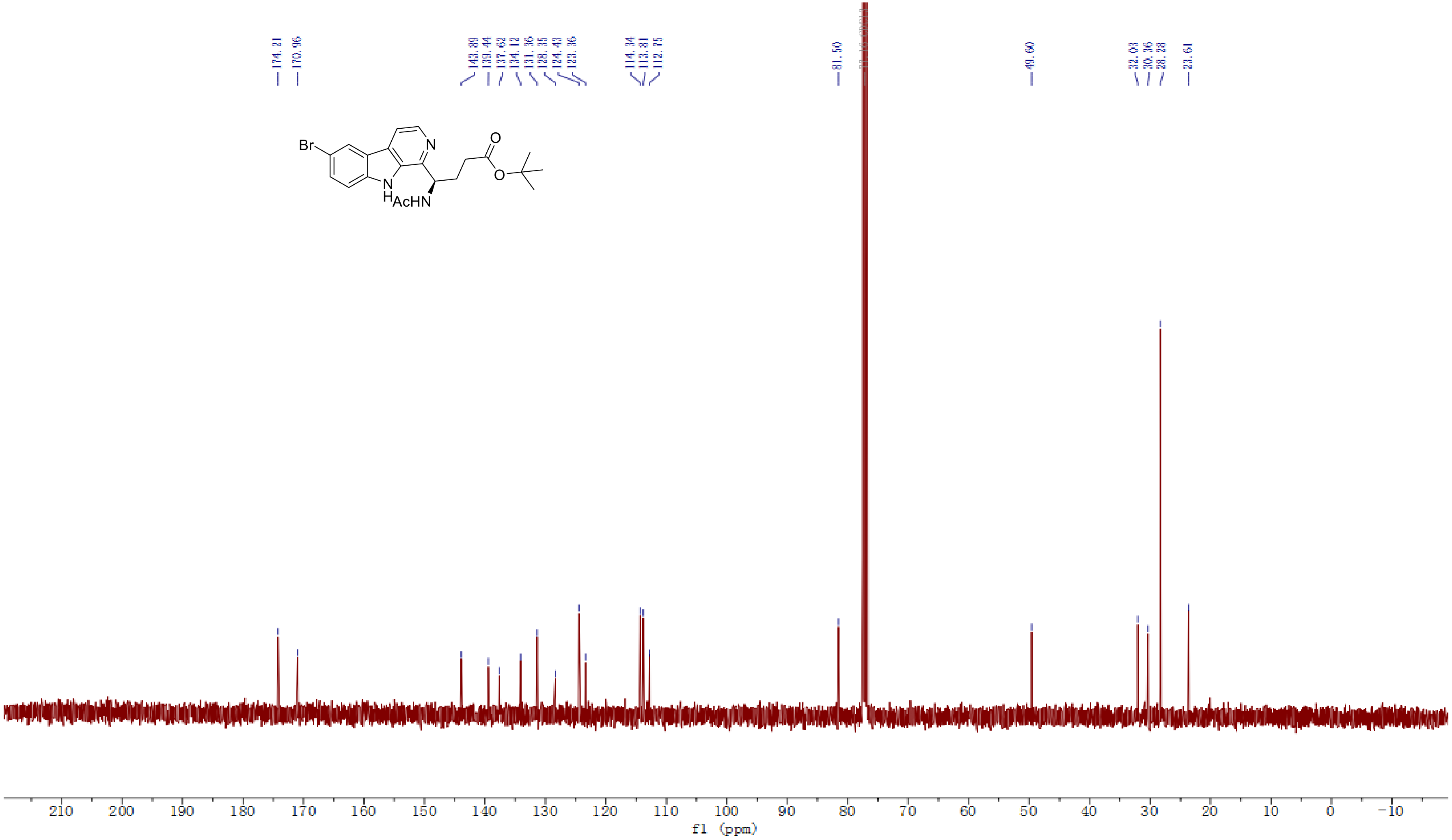

[1]H NMR(400 MHz, CDCl$_3$) (R)-tert-butyl -4-acetamido-4-(7-bromo-9*H*-pyrido[3,4-*b*]indol-1-yl)butanoate (**5m**)

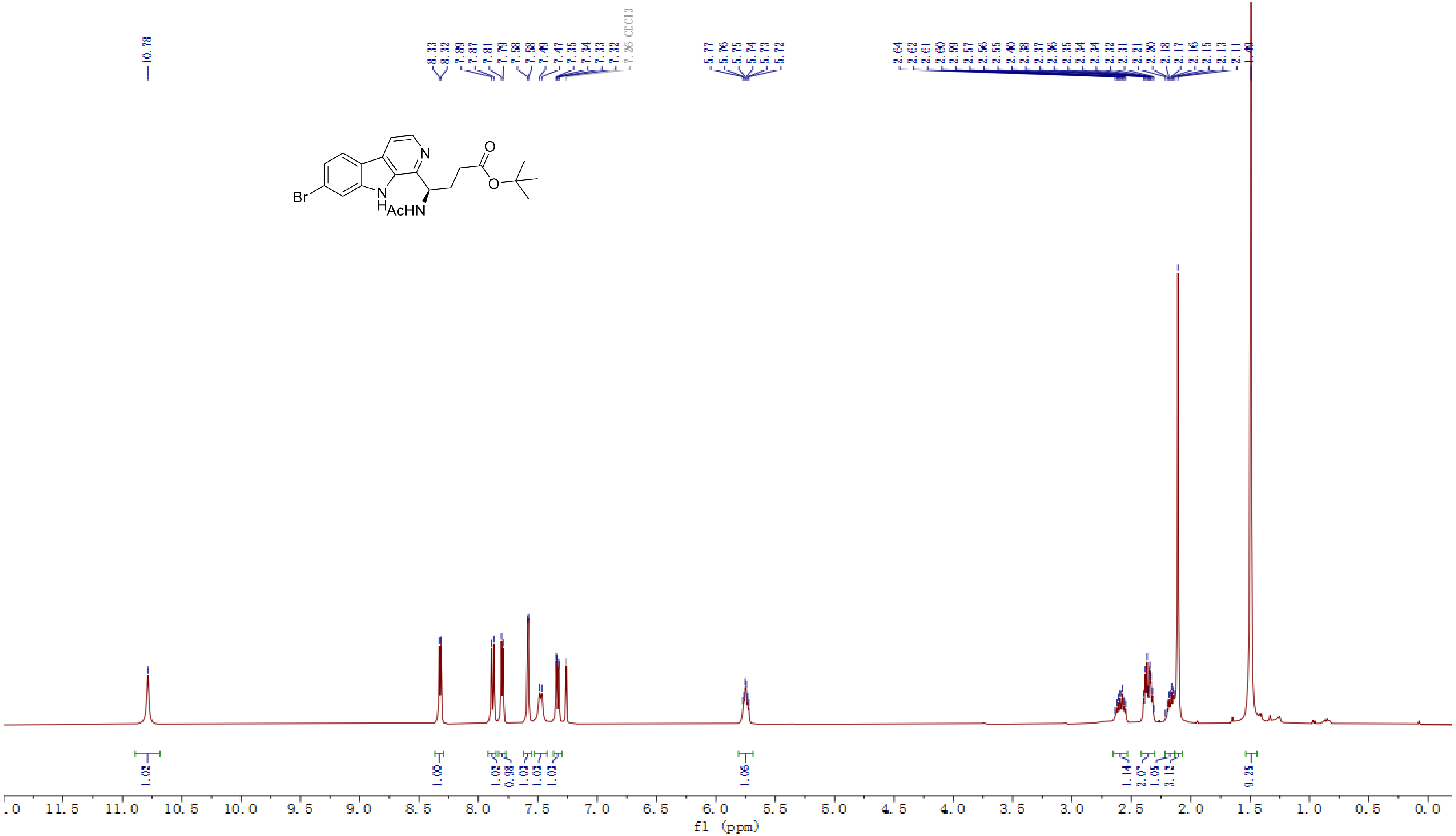

[13]C NMR(101 MHz, $CDCl_3$)(*R*)-tert-butyl-4-acetamido-4-(7-bromo-9*H*-pyrido[3,4-b]indol-1-yl)butanoate (**5m**)

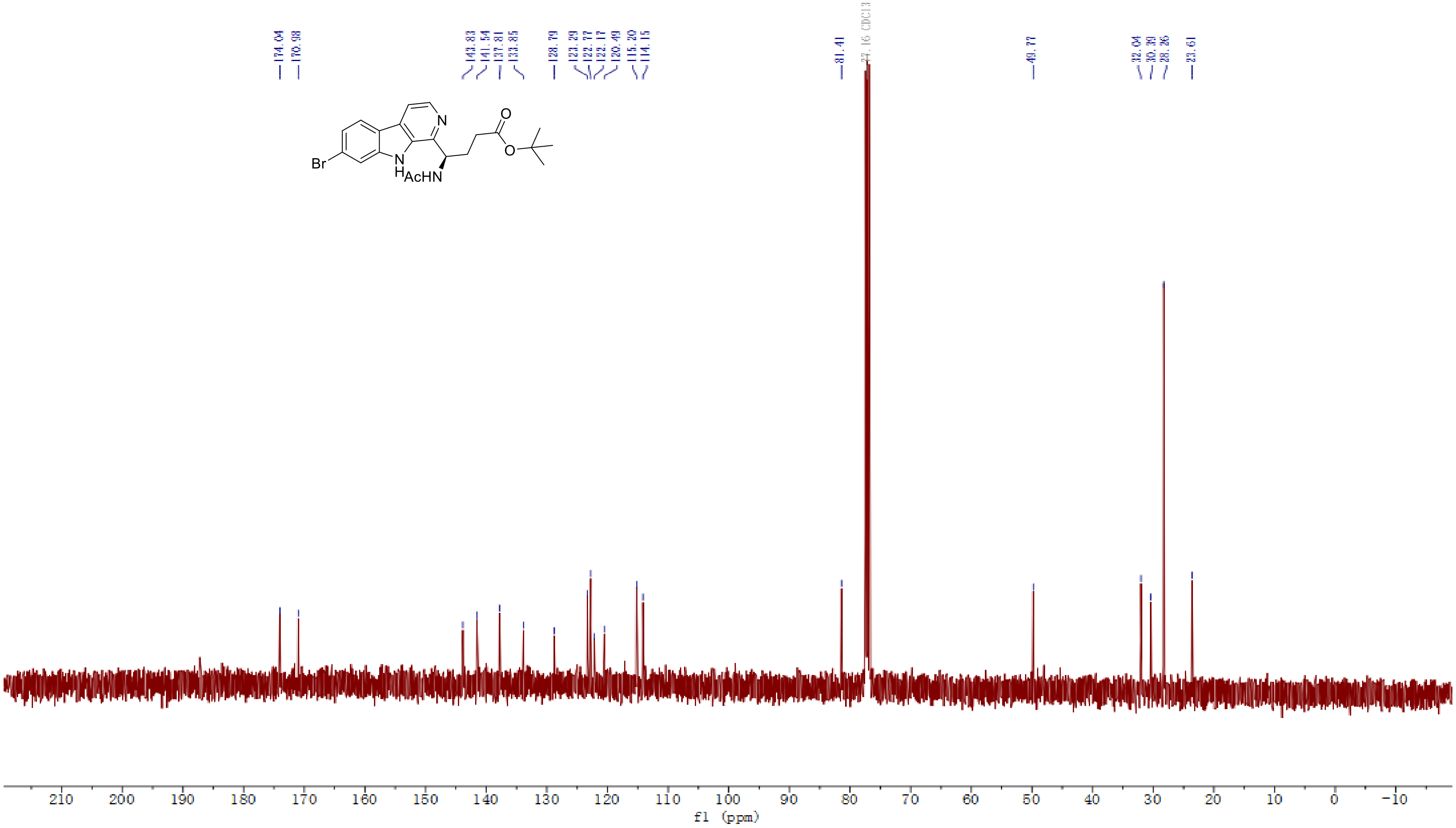

$^1$H NMR (400 MHz, CDCl$_3$) (*R*)-tert-butyl-4-acetamido-4-(8-bromo-9*H*-pyrido[3,4-b]indol-1-yl)butanoate (**5n**)

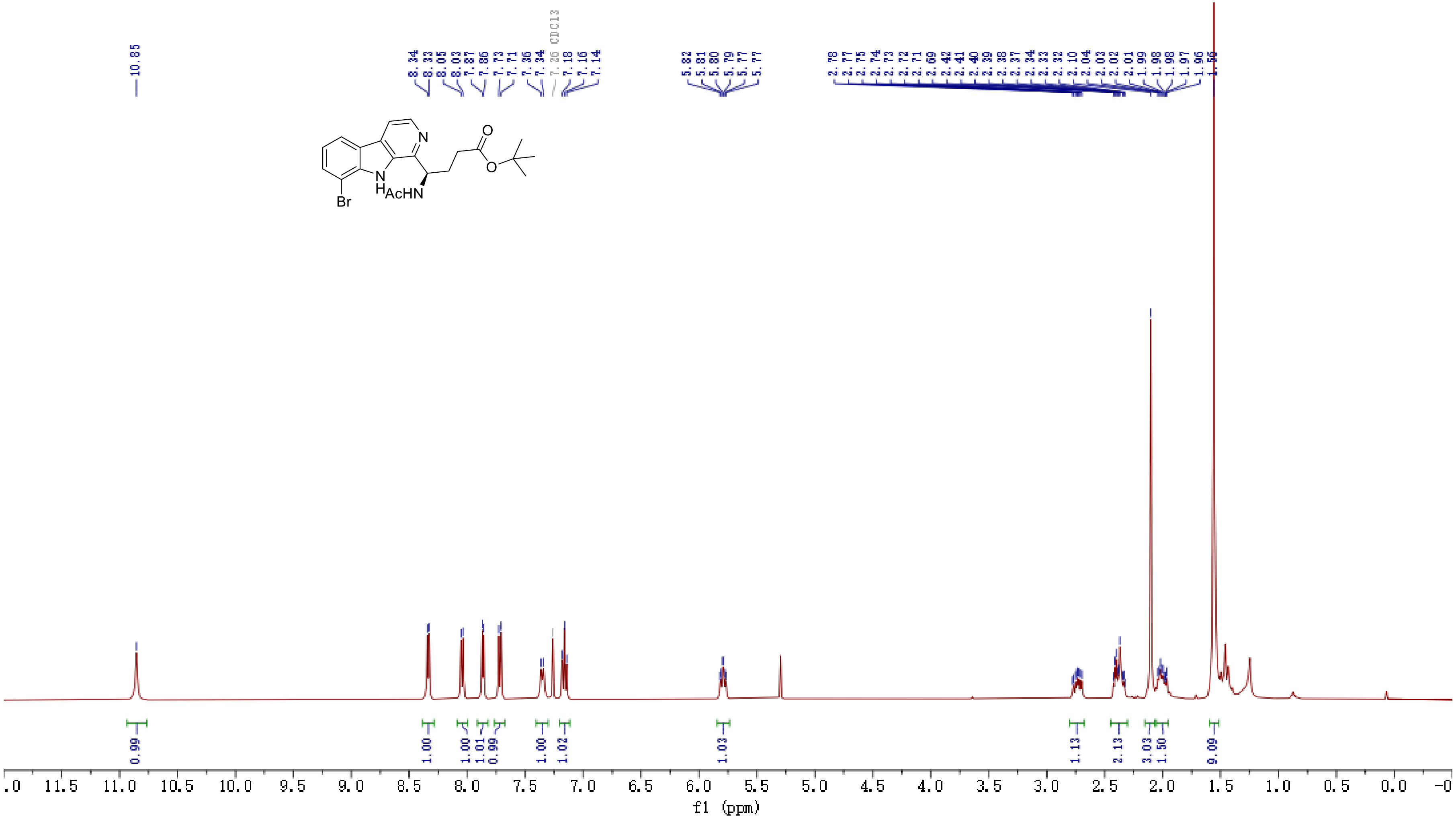

$^{13}C$ NMR(101 MHz, $CDCl_3$) (*R*)-tert-butyl-4-acetamido-4-(8-bromo-9*H*-pyrido[3,4-b]indol-1-yl)butanoate (**5n**)

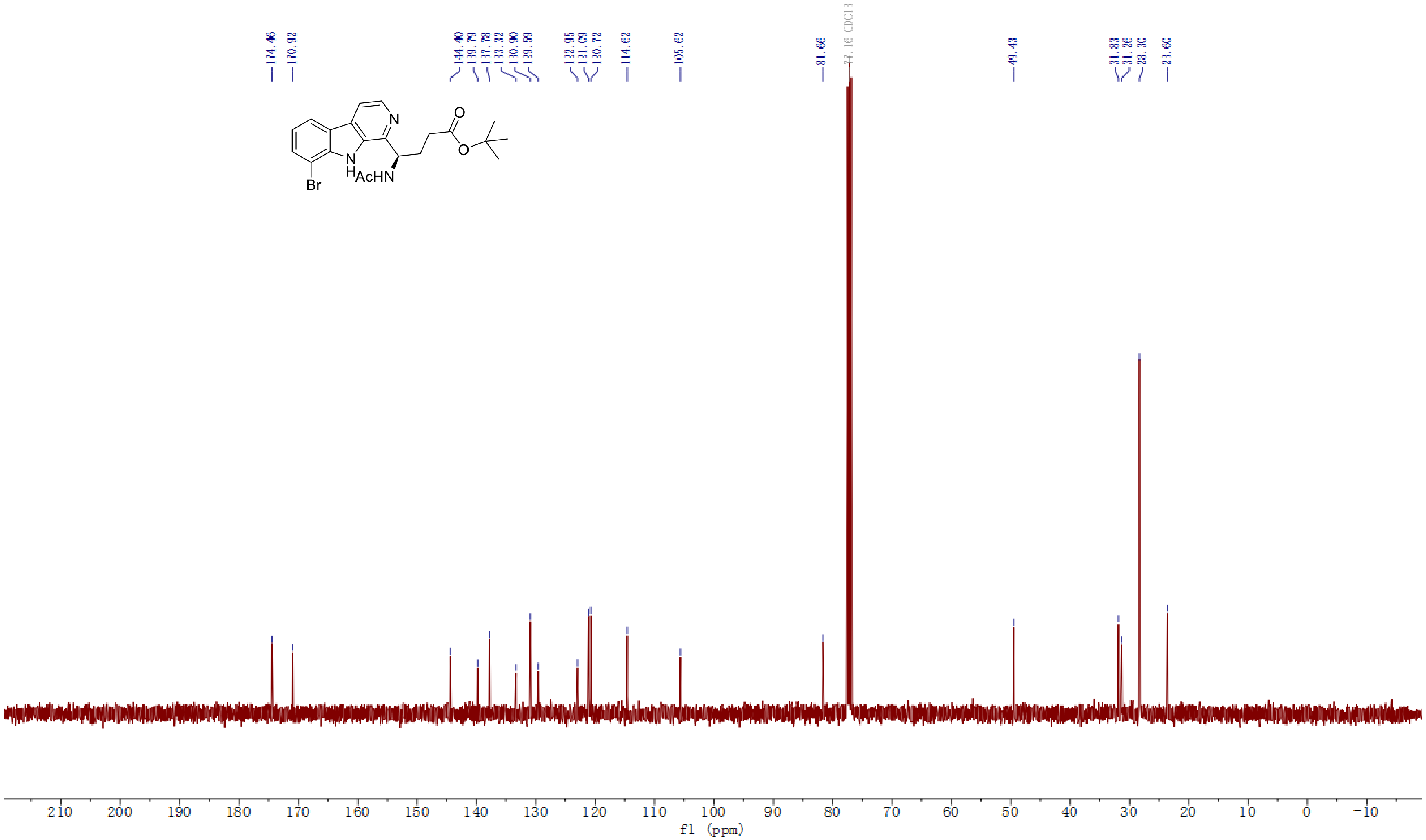

$^{1}H$ NMR (400 MHz, $CDCl_3$) (*R*)-tert-butyl-4-acetamido-4-(4-methylquinolin-2-yl)butanoate (**5o**)

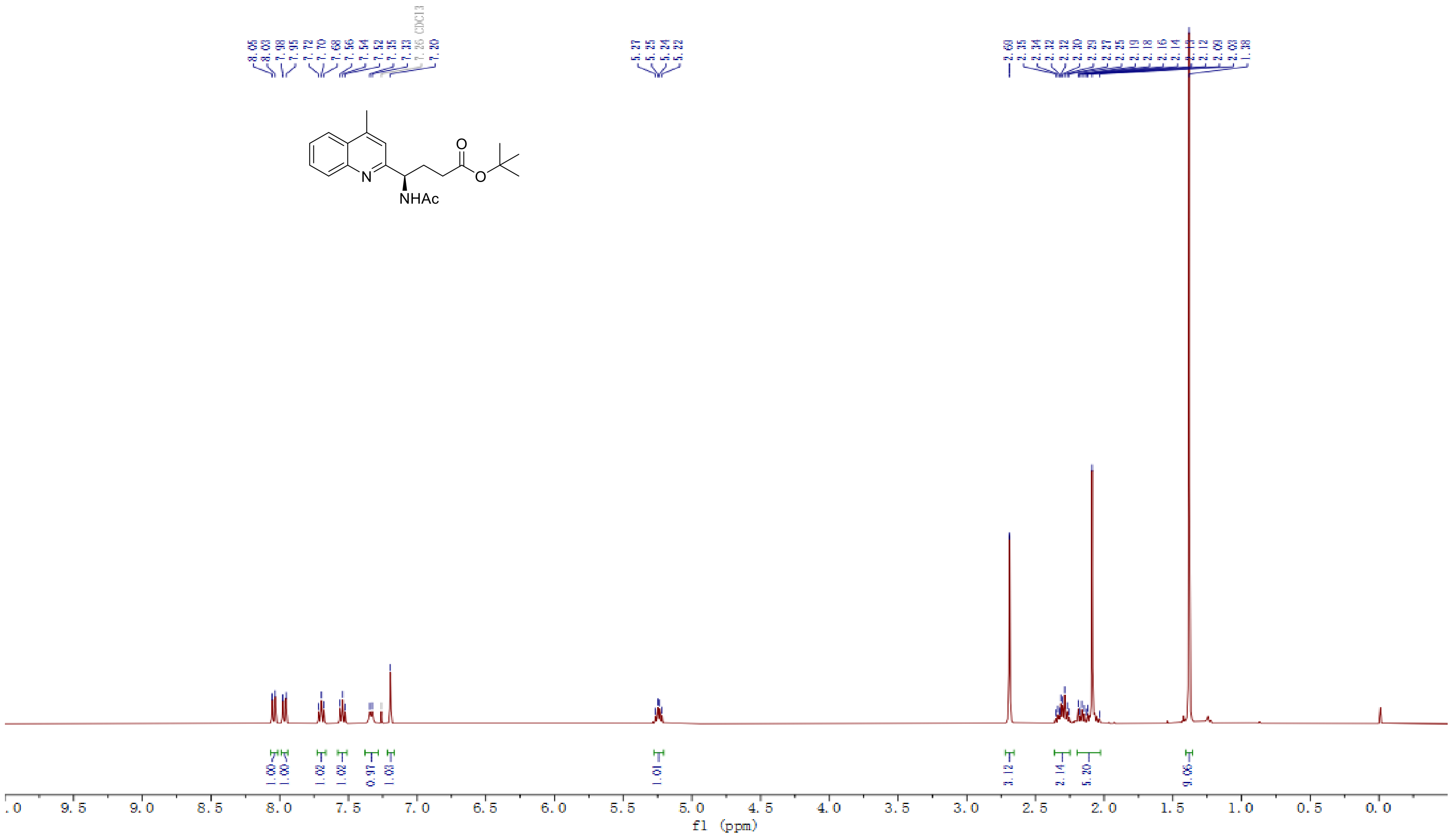

$^{1}$H NMR (400 MHz, CDCl$_3$) (*R*)-tert-butyl-4-acetamido-4-(isoquinolin-1-yl)butanoate (**5p**)

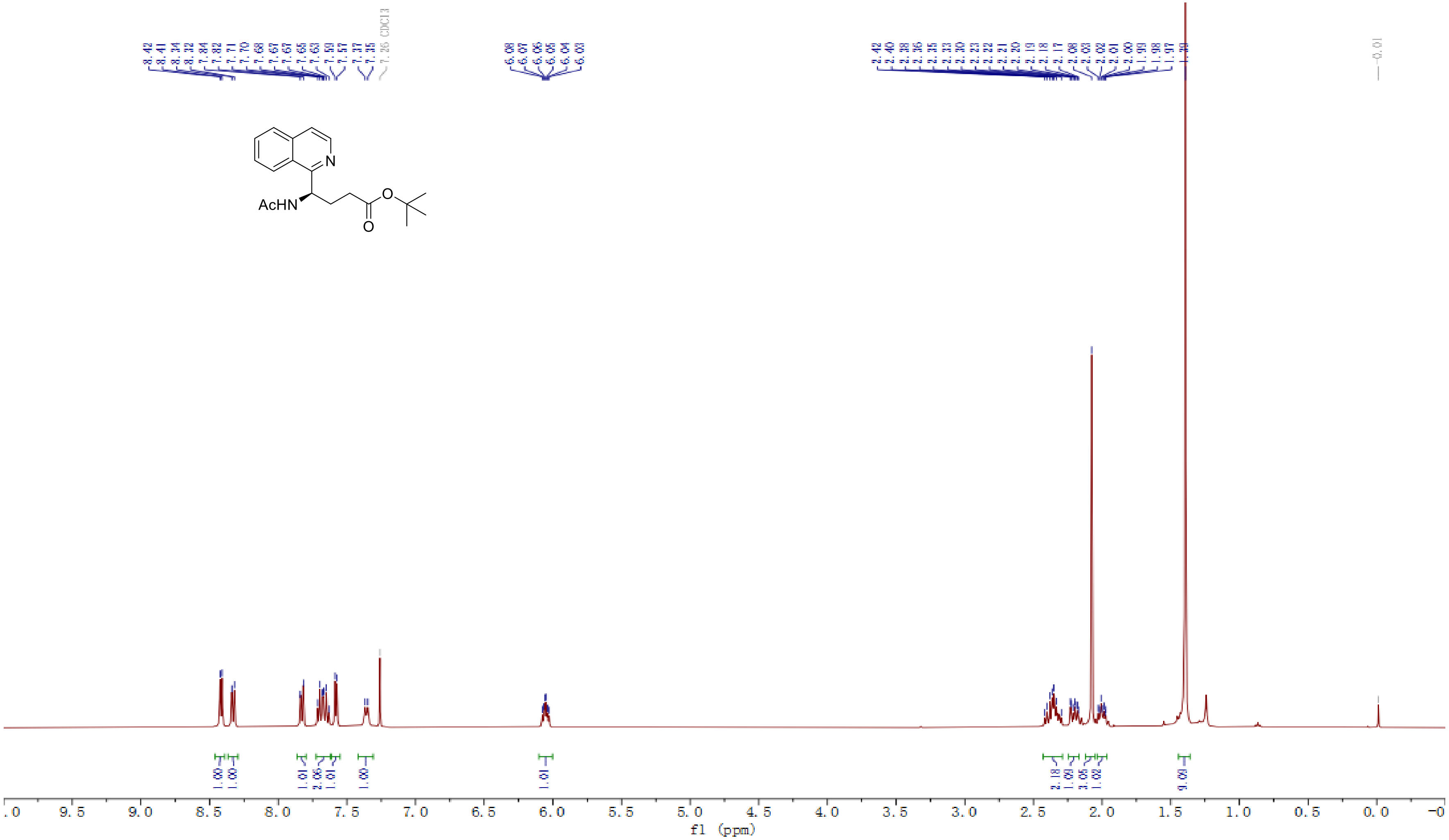

$^{1}$H NMR (400 MHz, $CDCl_3$) (*R*)-tert-butyl-4-acetamido-4-(isoquinolin-1-yl)butanoate (**5p**)

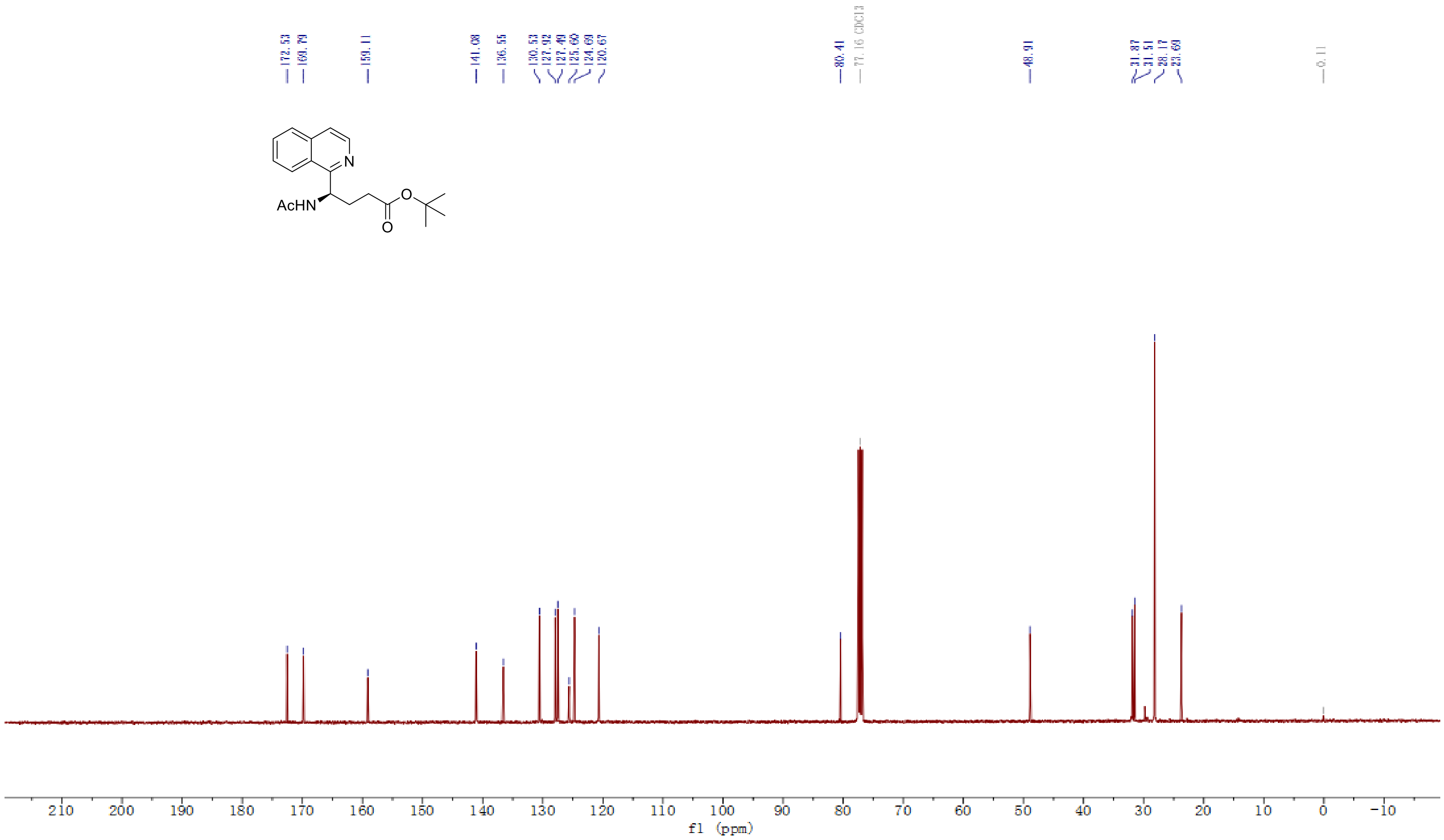

[1]H NMR (400 MHz, $CDCl_3$) (*R*)-ethyl-4-butyramido-4-(9*H*-pyrido[3,4-b]indol-1-yl)butanoate (**6b**)

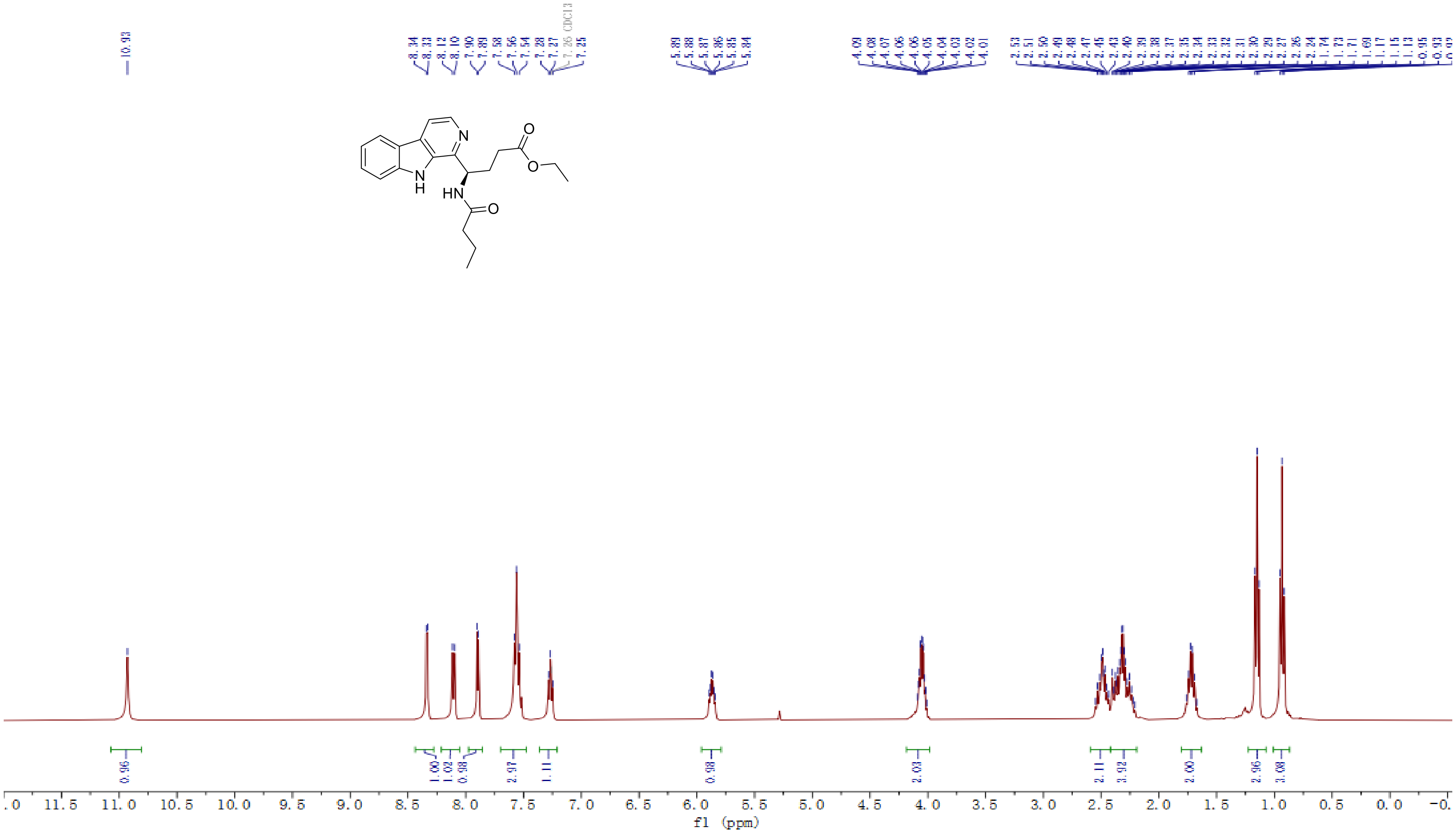

$^{13}C$ NMR (101 MHz, $CDCl_3$) (*R*)-ethyl-4-butyramido-4-(9*H*-pyrido[3,4-b]indol-1-yl)butanoate (**6b**)

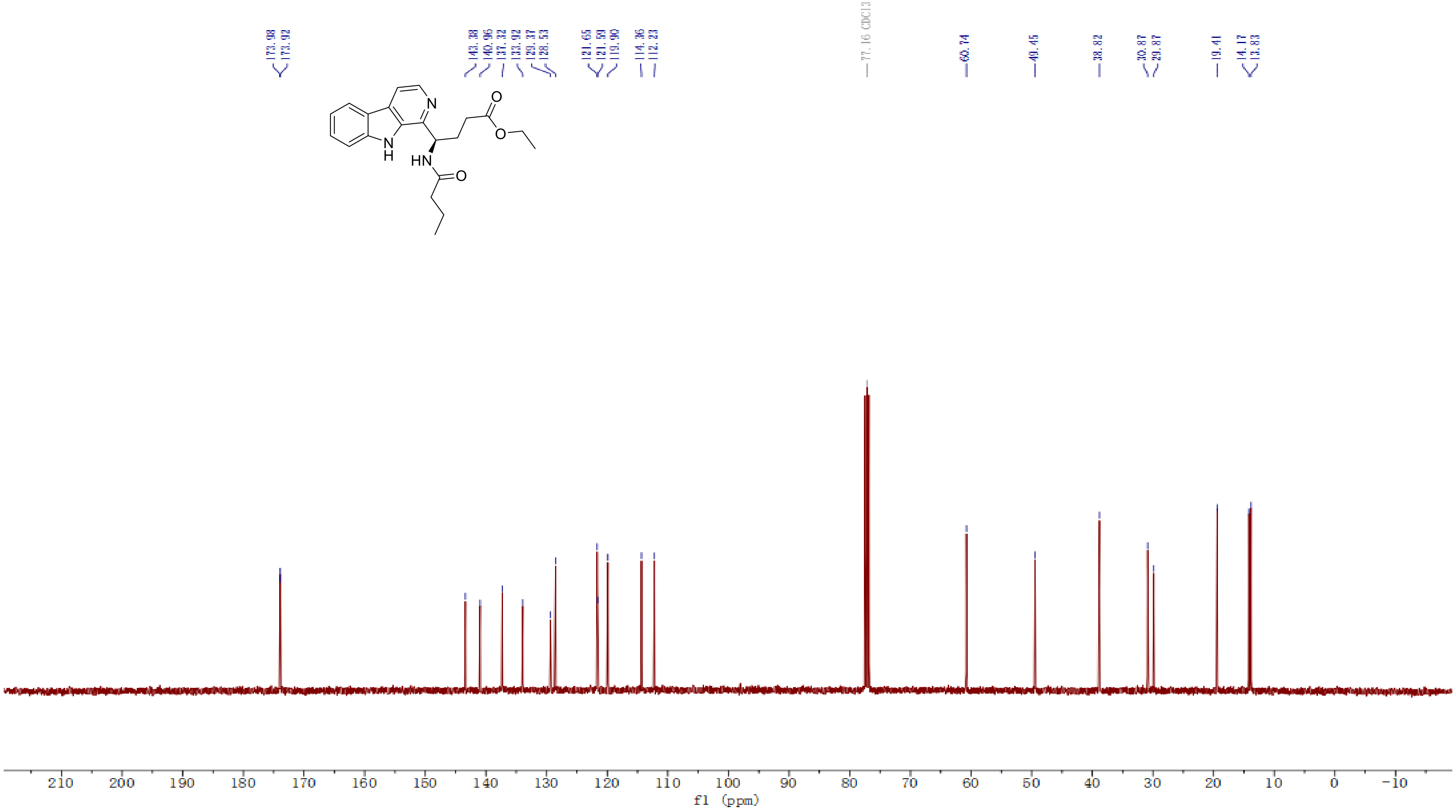

$^{1}H$ NMR (400 MHz, $CDCl_3$) (*R*)-ethyl-4-(cyclohexanecarboxamido)-4-(9*H*-pyrido[3,4-*b*]indol-1-yl)butanoate (**6c**)

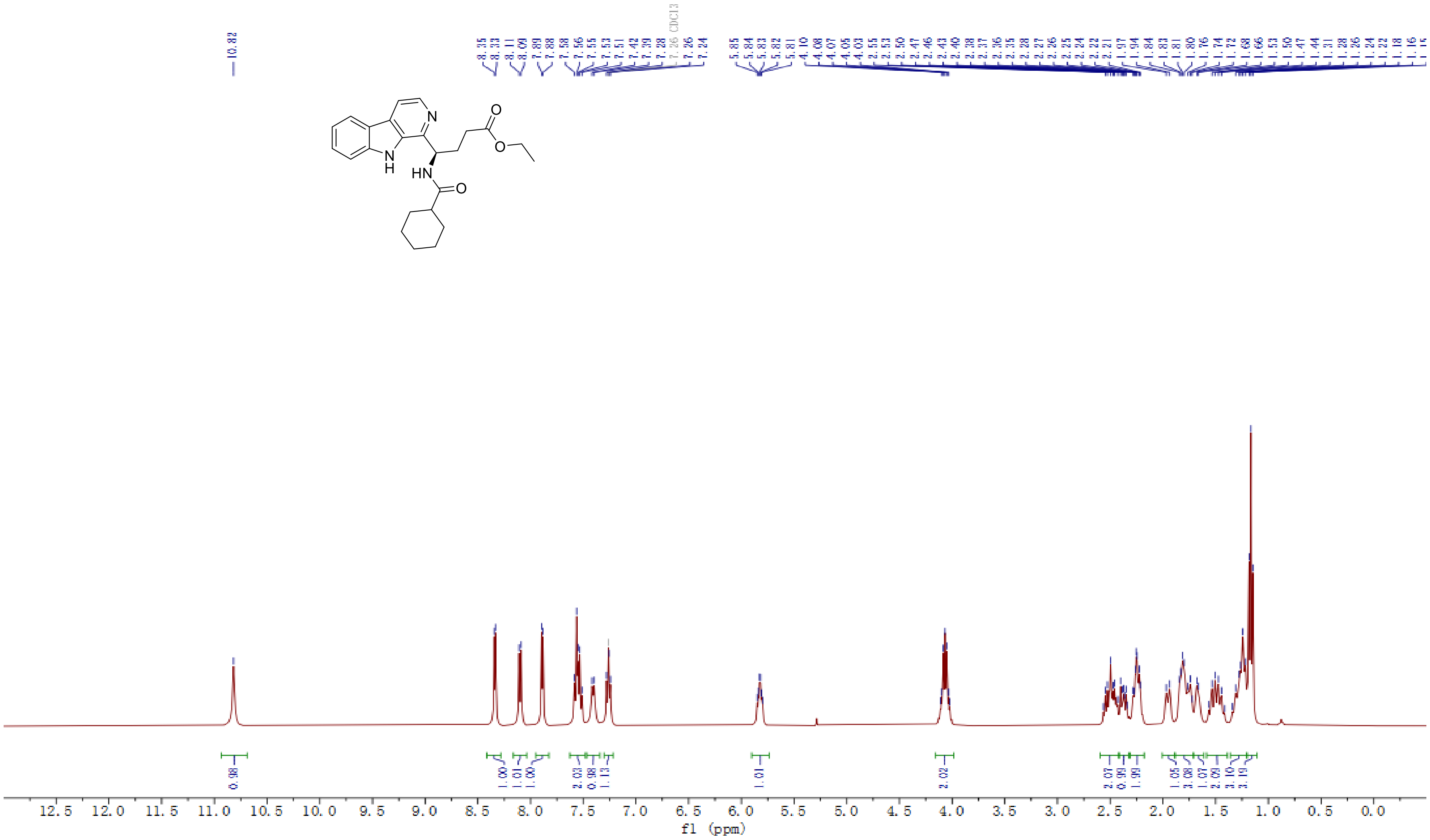

$^{13}C$ NMR (101 MHz, $CDCl_3$) (*R*)-ethyl-4-(cyclohexanecarboxamido)-4-(9*H*-pyrido[3,4-*b*]indol-1-yl)butanoate (**6c**)

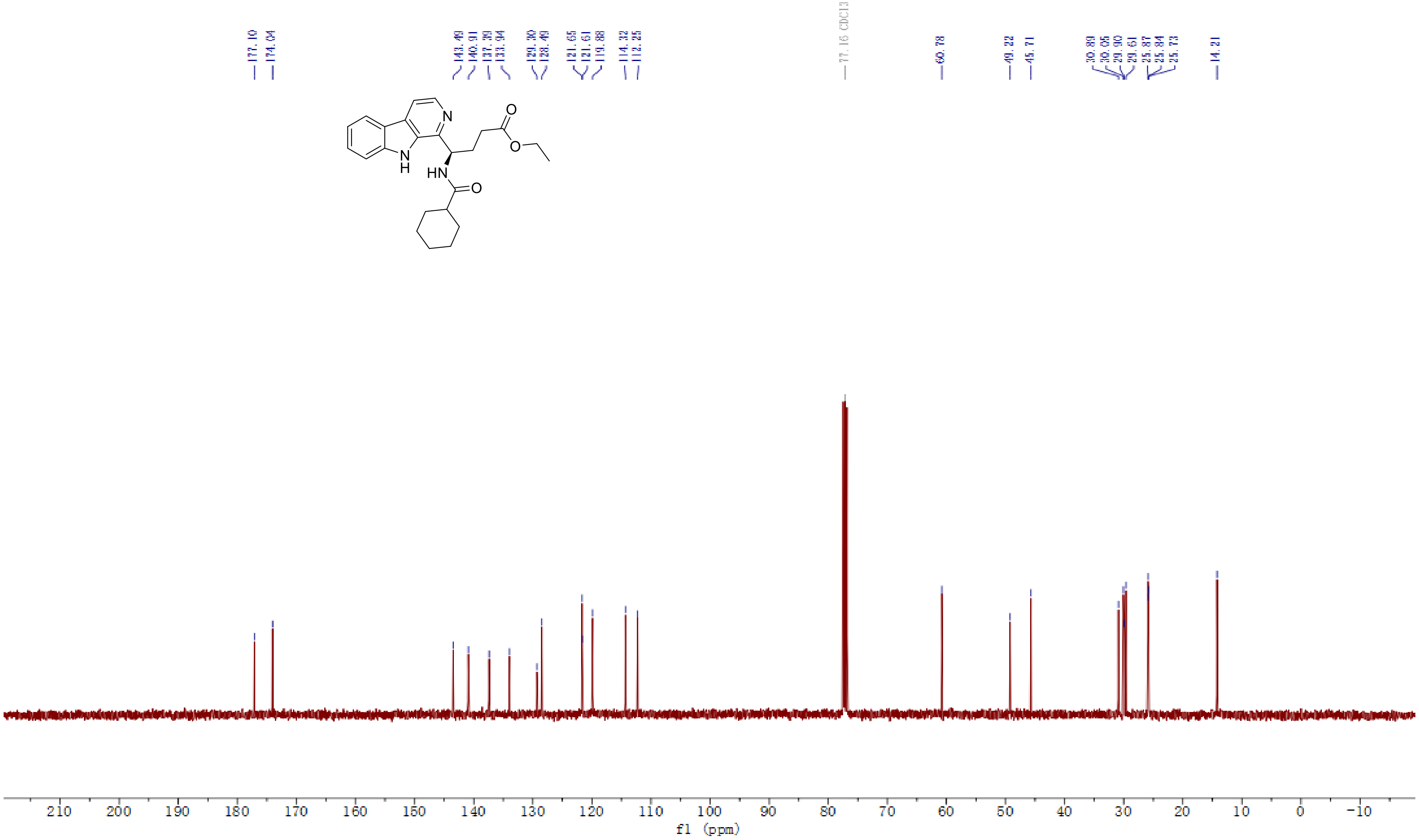

$^1$H NMR (400 MHz, CDCl$_3$) (*R*)-ethyl-4-formamido-4-(9*H*-pyrido[3,4-*b*]indol-1-yl)butanoate (**6d**)

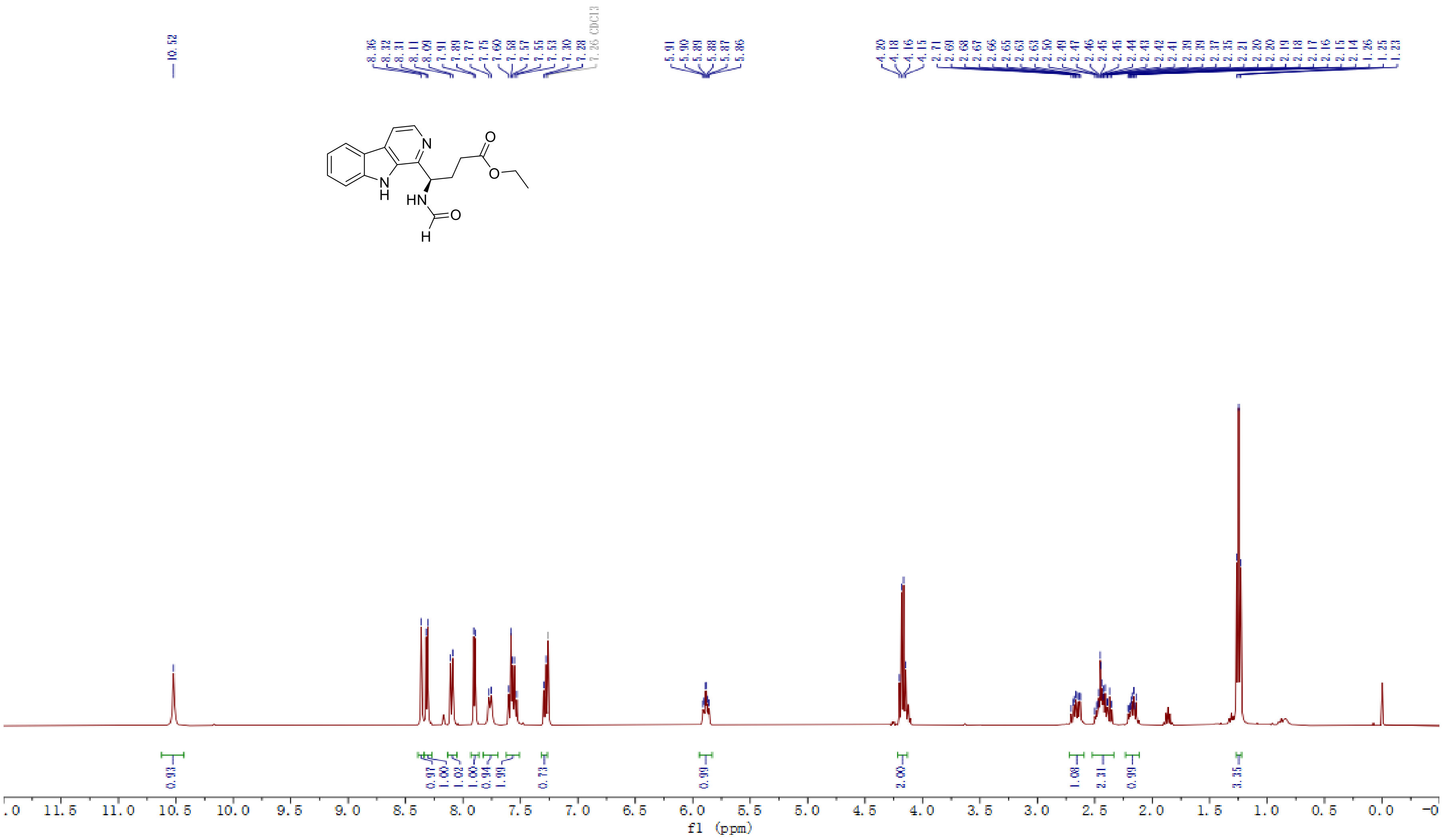

$^{13}C$ NMR (101 MHz, $CDCl_3$) (*R*)-ethyl-4-formamido-4-(9*H*-pyrido[3,4-*b*]indol-1-yl)butanoate (**6d**)

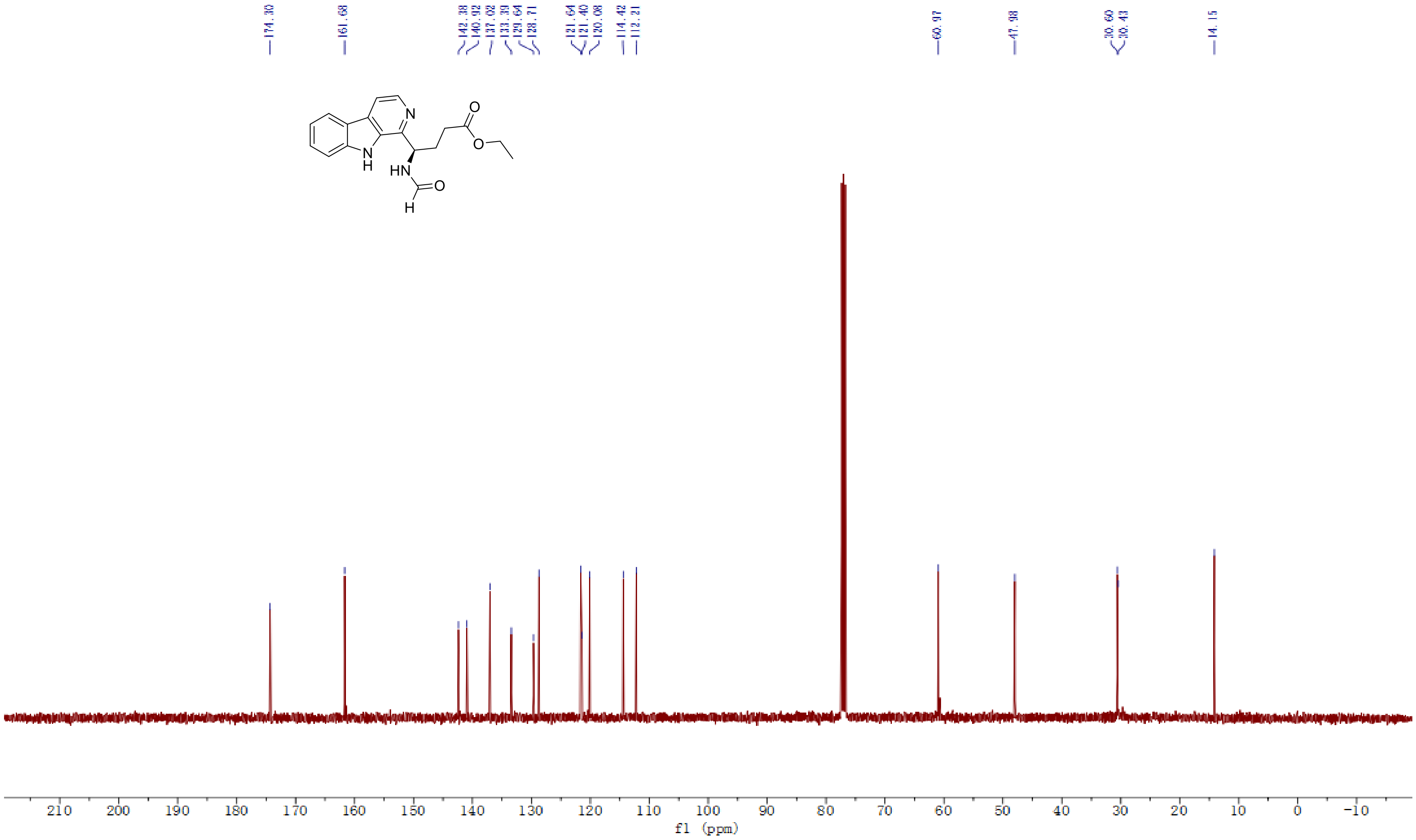

[1]H NMR (400 MHz, $CDCl_3$) ethyl-4-(*N*-methylacetamido)-4-(9*H*-pyrido[3,4-*b*]indol-1-yl)butanoate (**6g**)

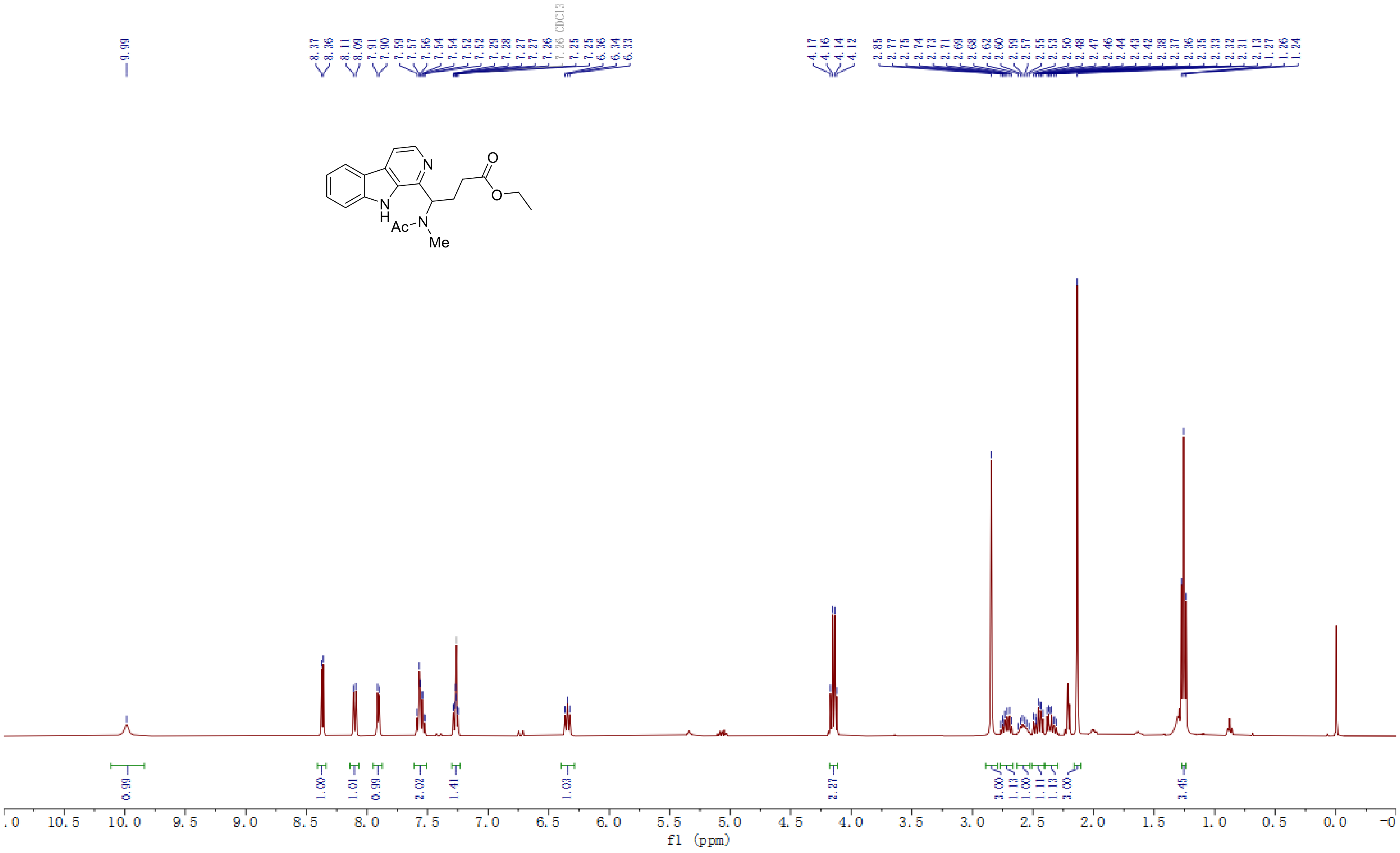

$^{13}$C NMR (101 MHz, DMSO-$d_6$) ethyl-4-(*N*-methylacetamido)-4-(9*H*-pyrido[3,4-*b*]indol-1-yl)butanoate (**6g**)

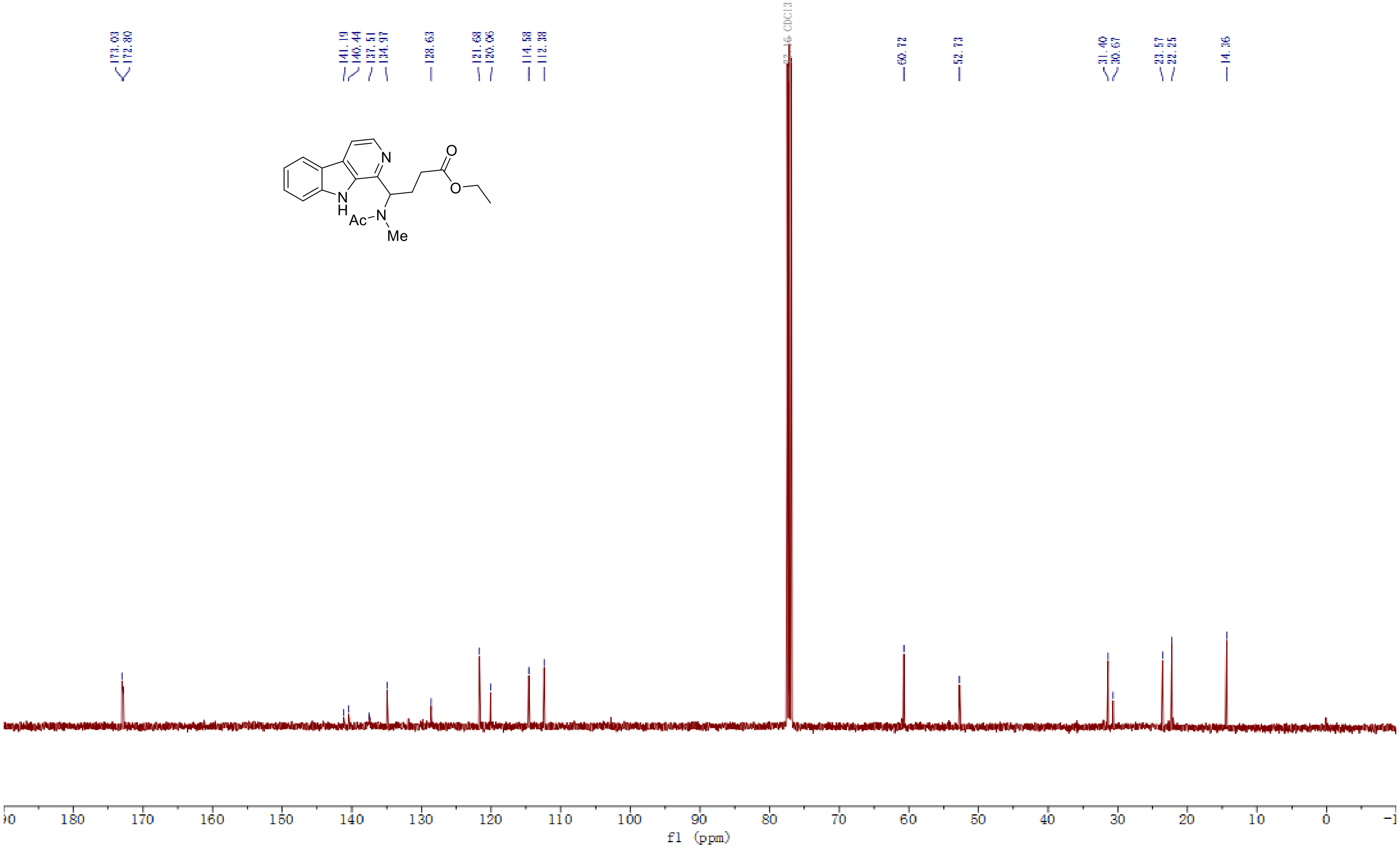

[1]H NMR (400 MHz, DMSO-$d_6$) (*R*)-5-(9*H*-pyrido[3,4-*b*]indol-1-yl)pyrrolidin-2-one (**7**)

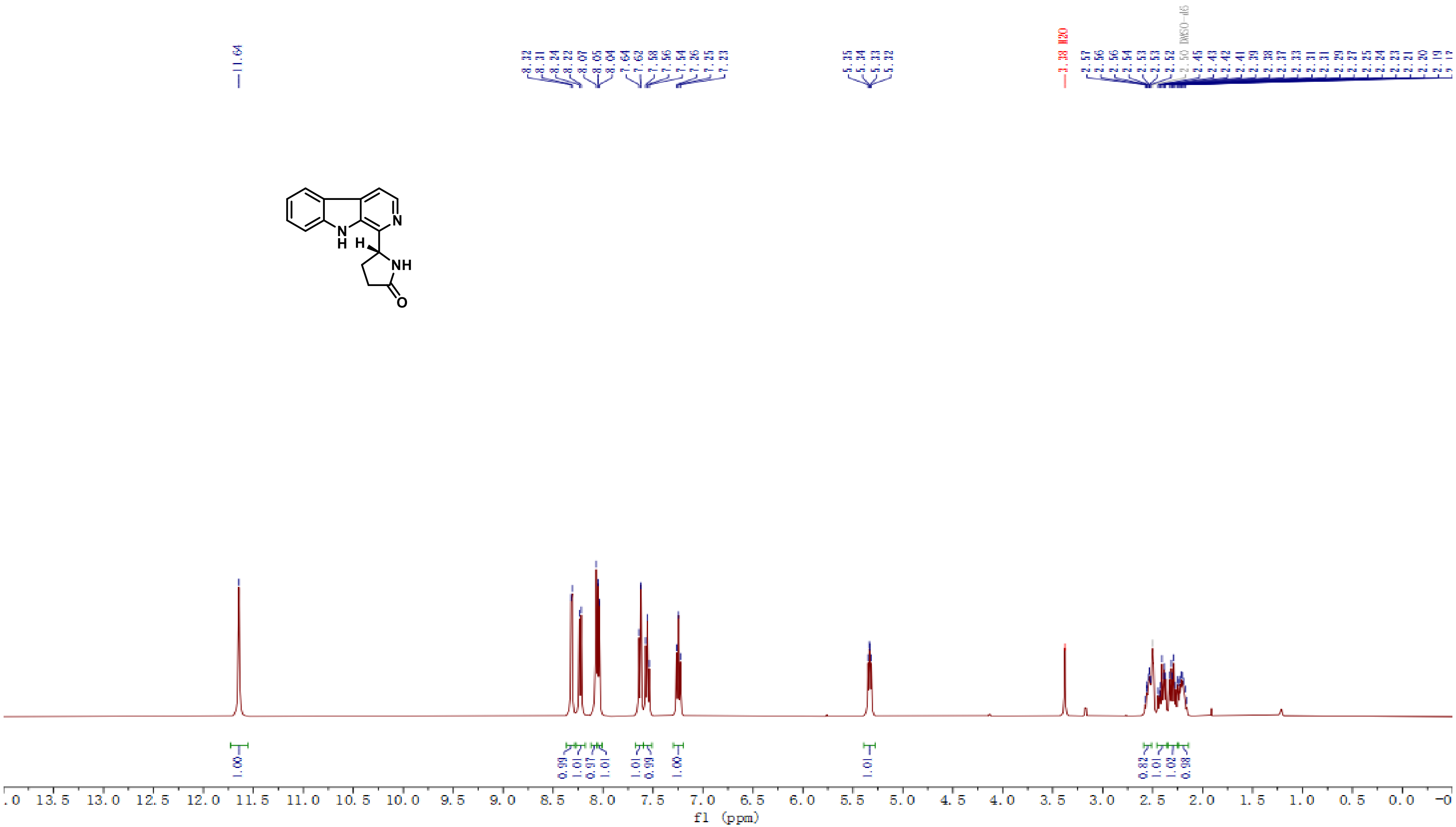

$^{13}$C NMR (101 MHz, DMSO-$d_6$) (*R*)-5-(9*H*-pyrido[3,4-*b*]indol-1-yl)pyrrolidin-2-one (**7**)

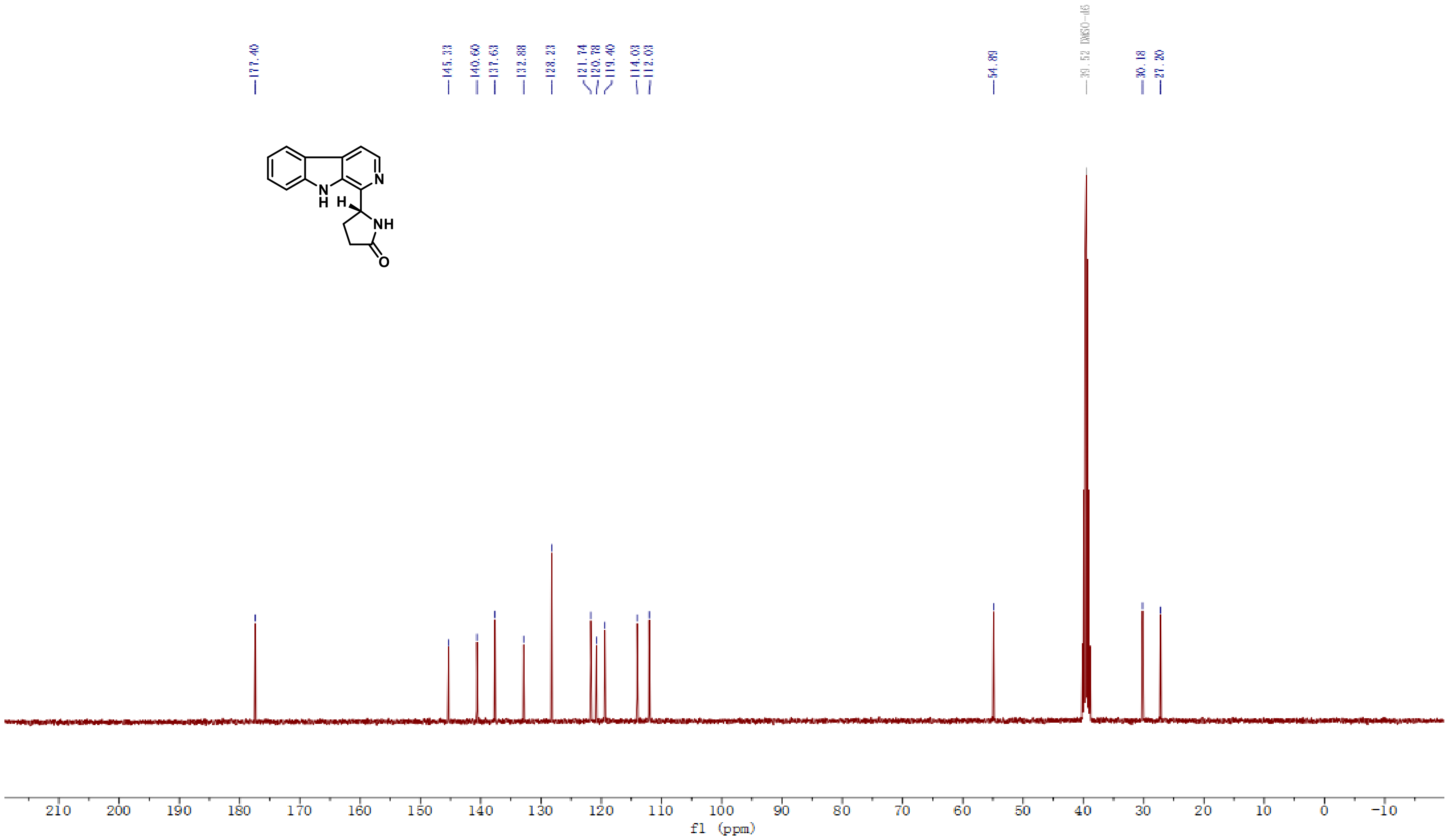

$^{1}H$ NMR (400 MHz, $CDCl_3$) (*R*)-dihydroeudistomin I (**8**)

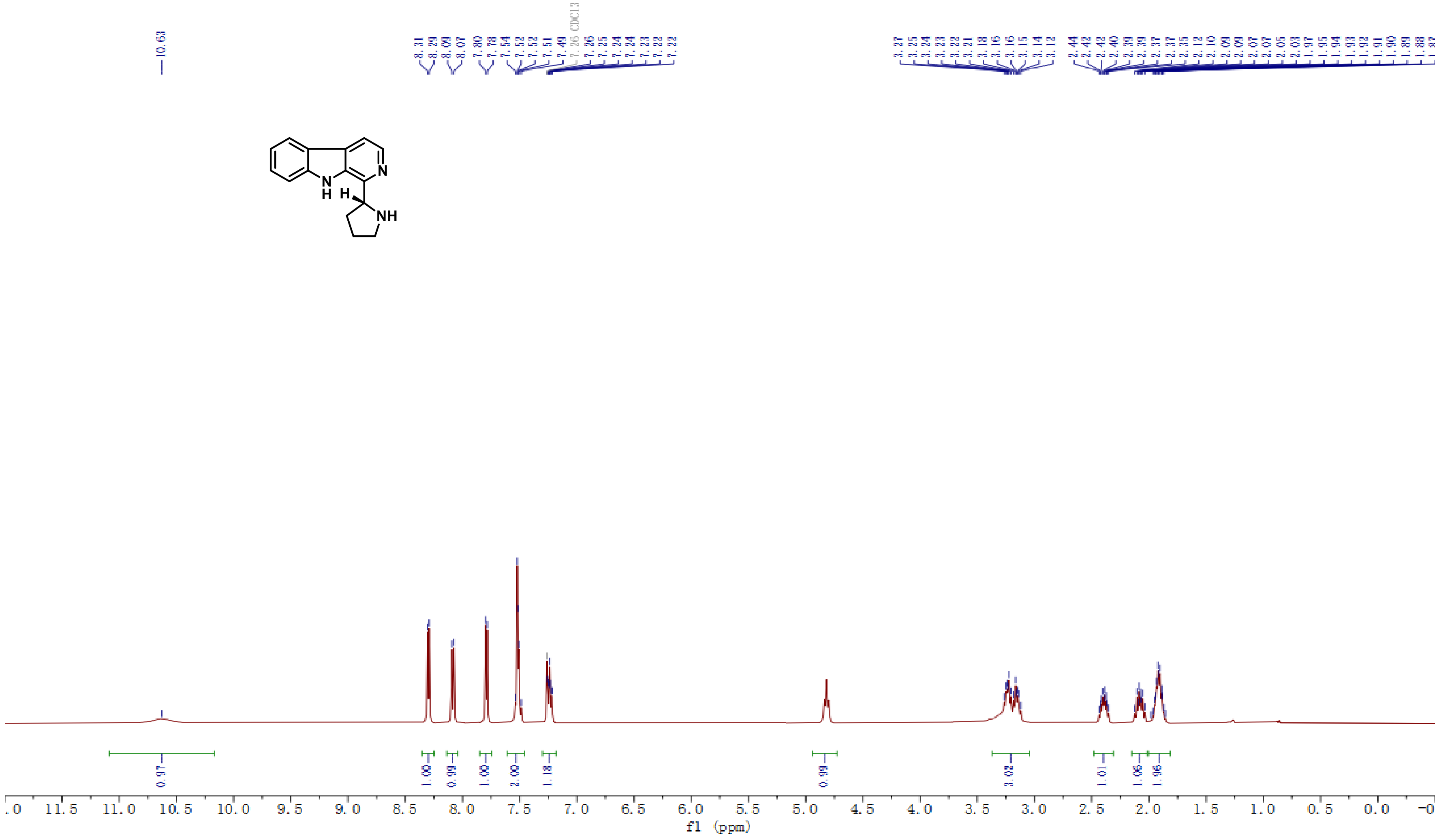

$^{13}C$ NMR (101 MHz, $CDCl_3$) (*R*)-dihydroeudistomin I (**8**)

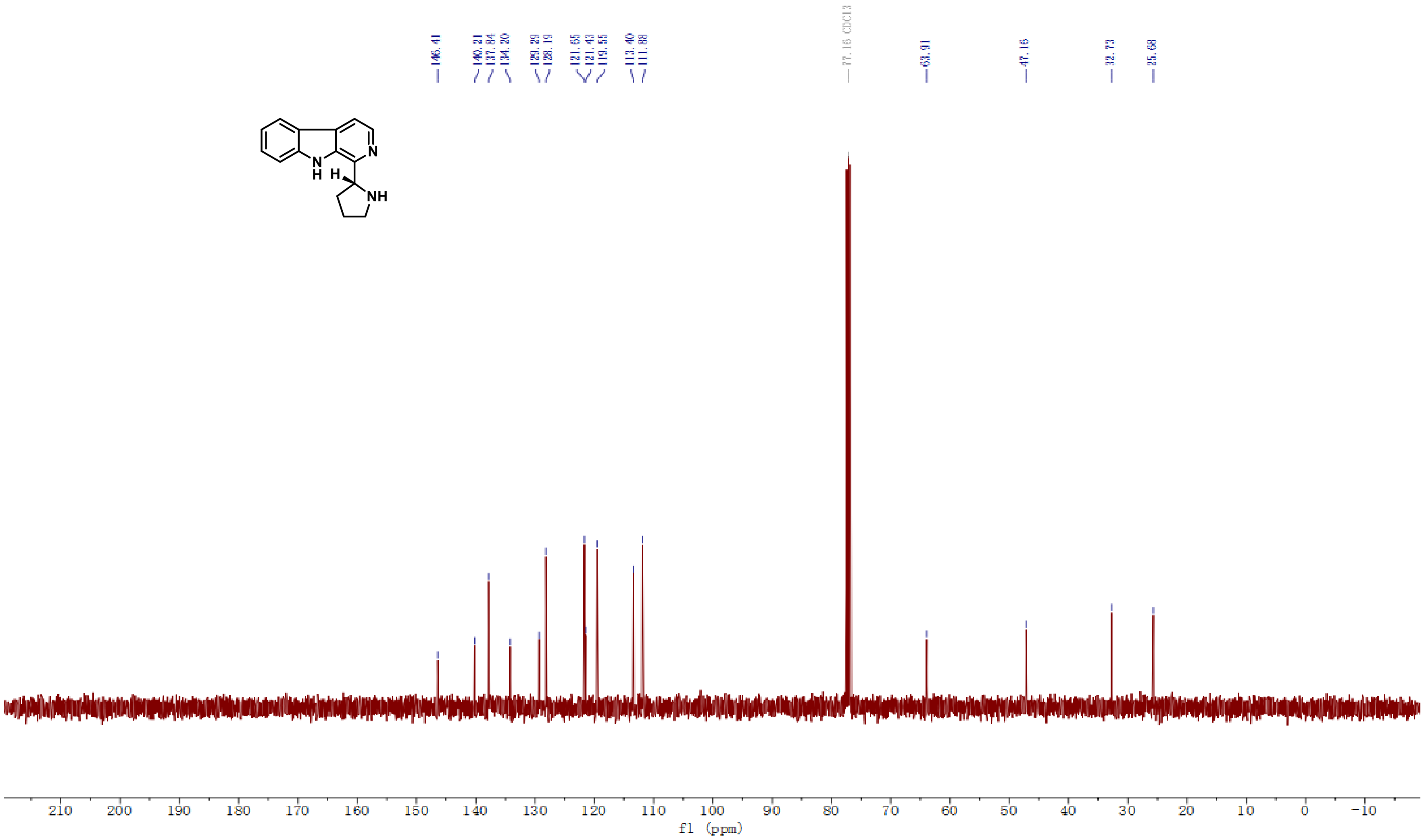

¹H NMR (400 MHz, DMSO-$d_6$) (*R*)-*N*-(4-hydroxy-1-(9*H*-pyrido[3,4-*b*]indol-1-yl)butyl)acetamide (**9**)

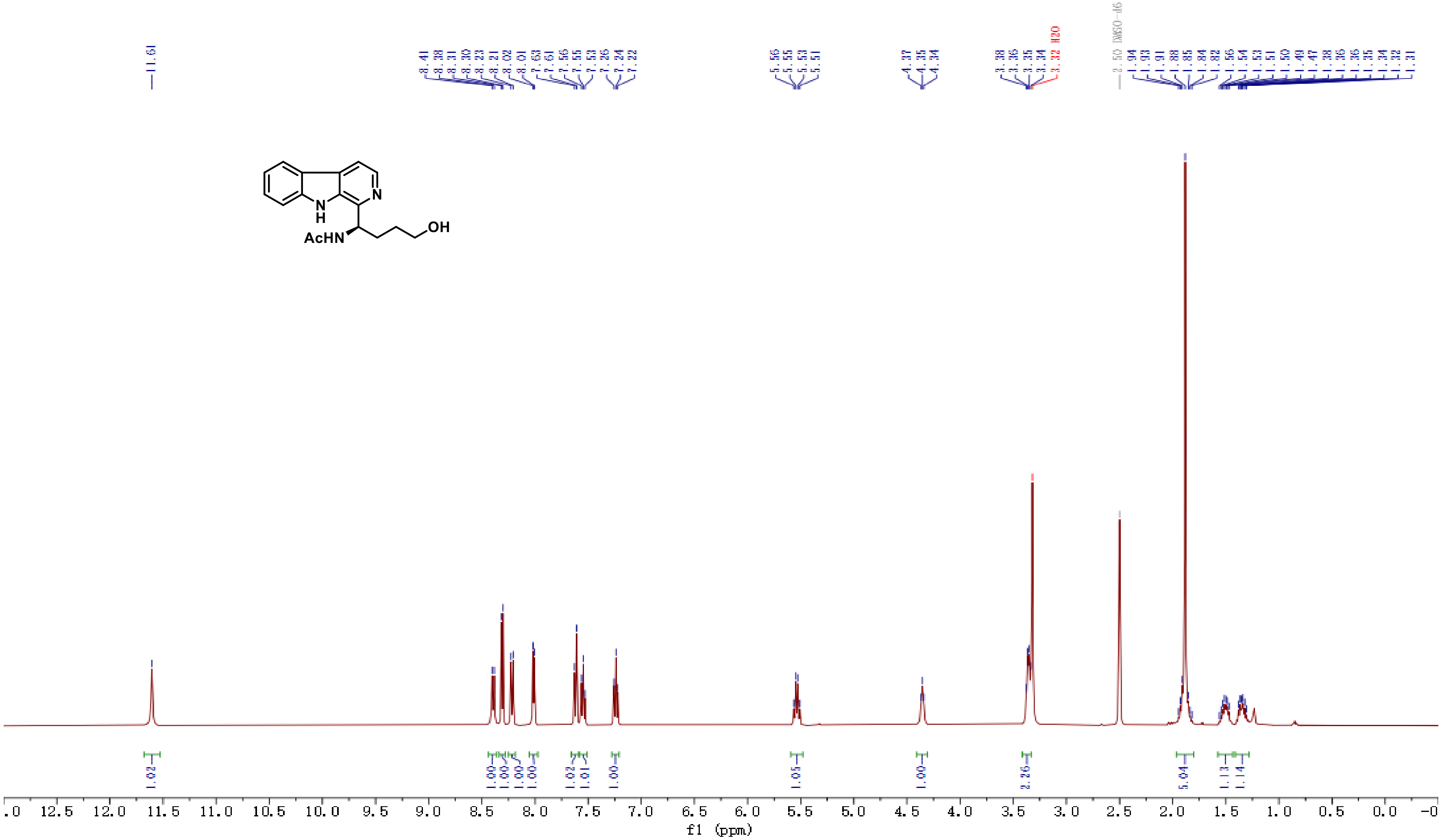

$^{13}$C NMR (101 MHz, DMSO-$d_6$) (*R*)-*N*-(4-hydroxy-1-(9*H*-pyrido[3,4-*b*]indol-1-yl)butyl)acetamide (**9**)

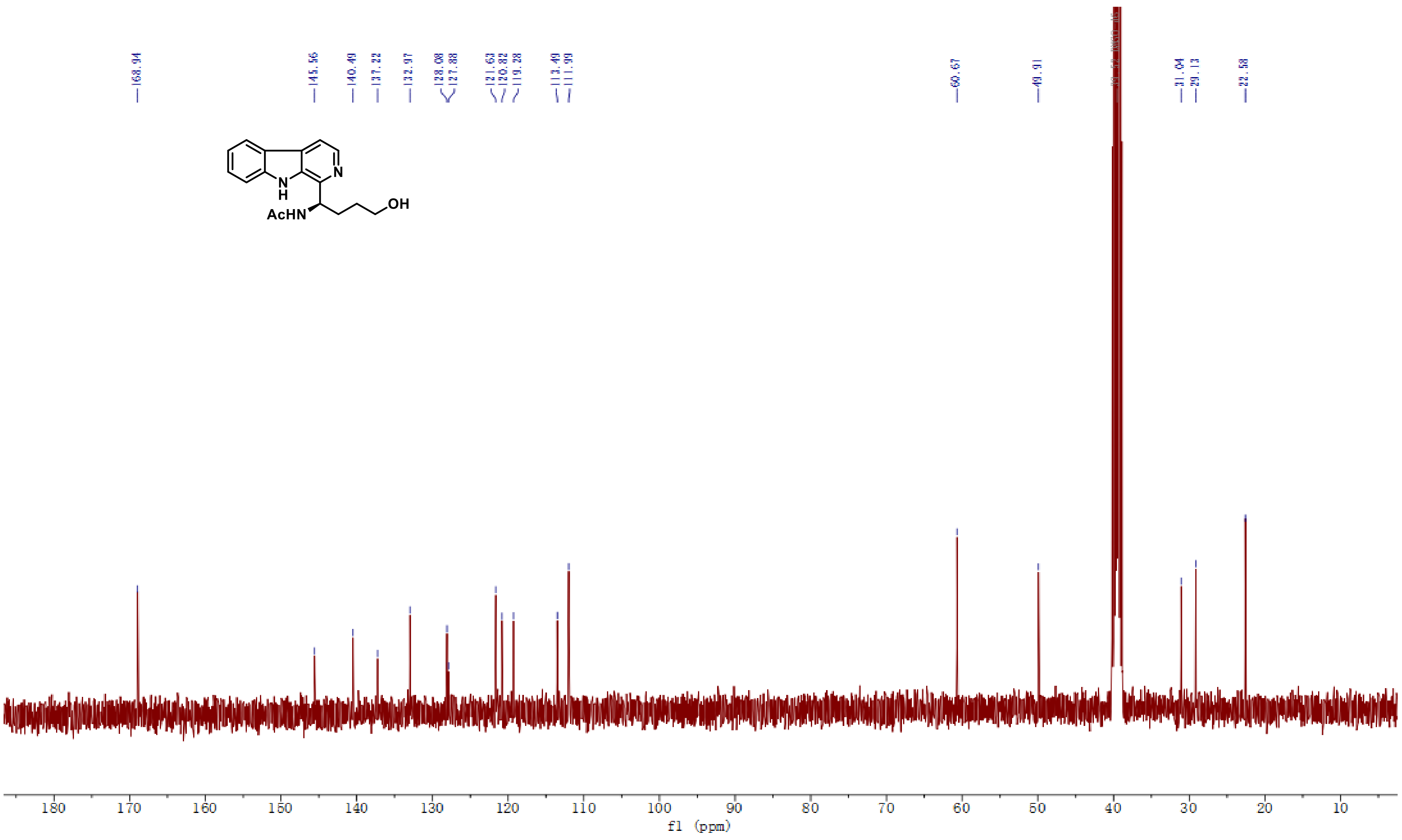

[1]H NMR (400 MHz, $CDCl_3$) *N*-((1*R*,12b*S*)-1,2,3,4,6,7,12,12b-octahydroindolo[2,3-*a*]quinolizin-1-yl)acetamide (**10**)

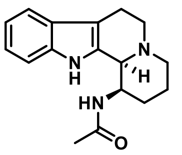

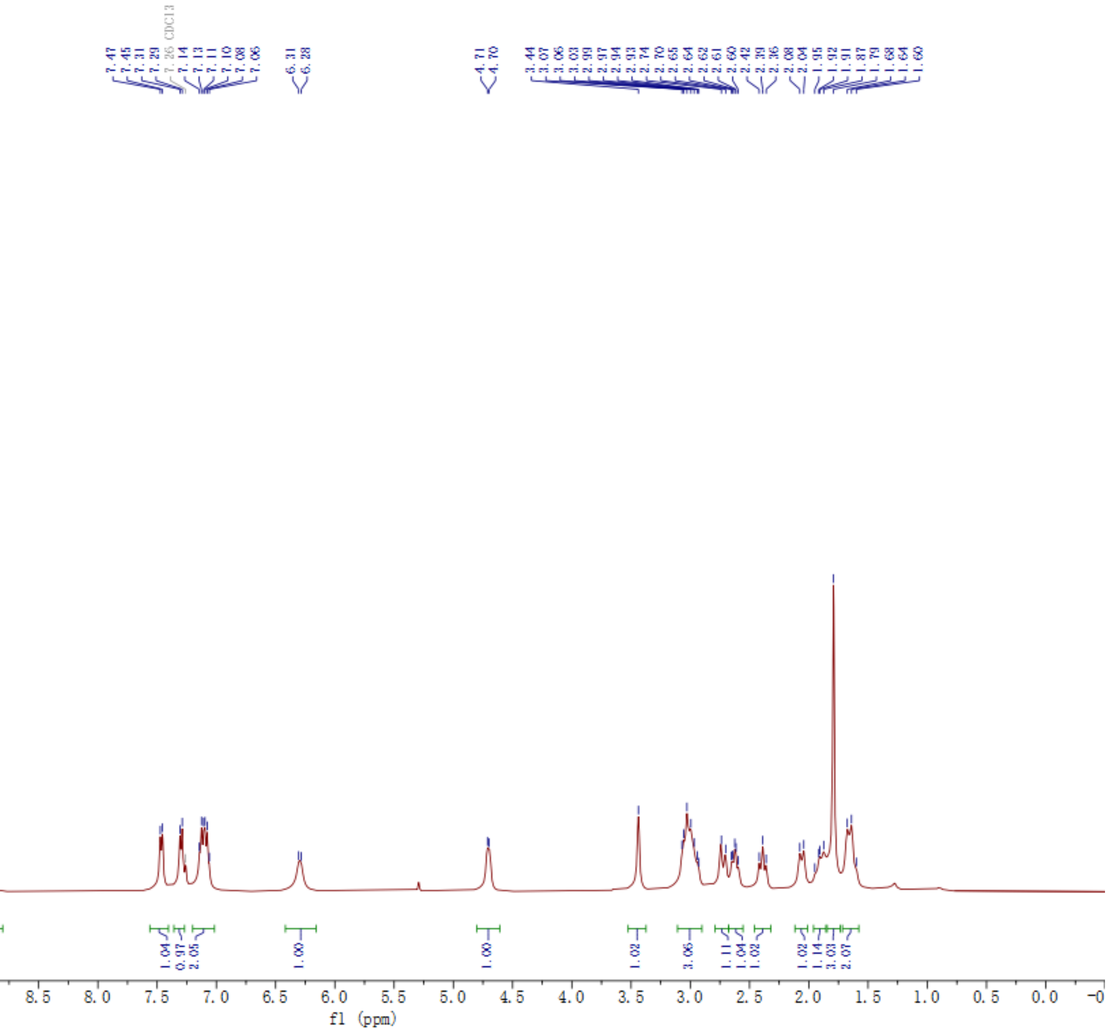

$^{13}C$ NMR (101 MHz, $CDCl_3$) *N*-((1*R*,12b*S*)-1,2,3,4,6,7,12,12b-octahydroindolo[2,3-*a*]quinolizin-1-yl)acetamide (**10**)

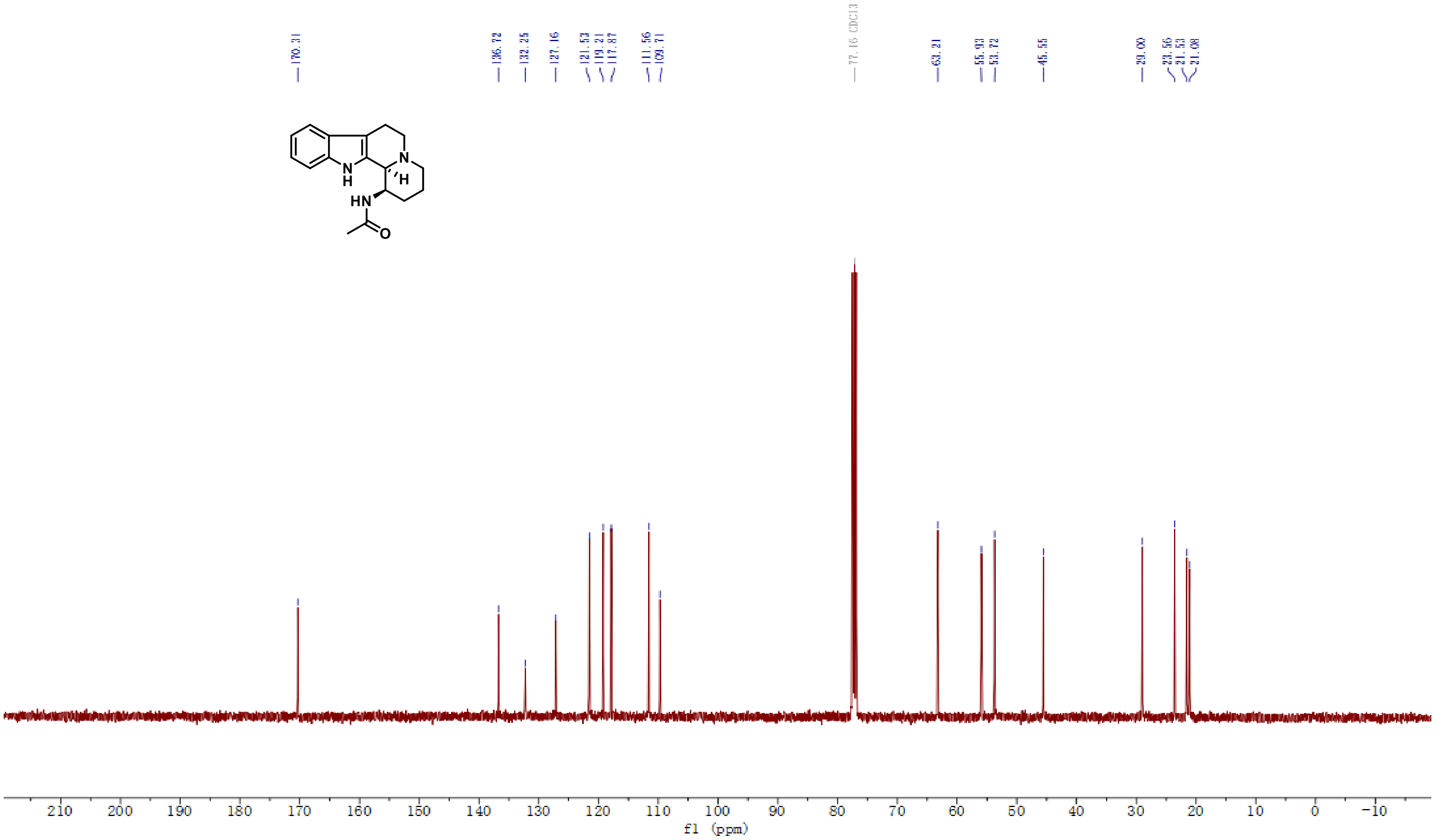

[1]H NMR (400 MHz, $CDCl_3$) (1*R*,12b*S*)-aminoindoloquinolizidine (**11**)

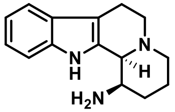

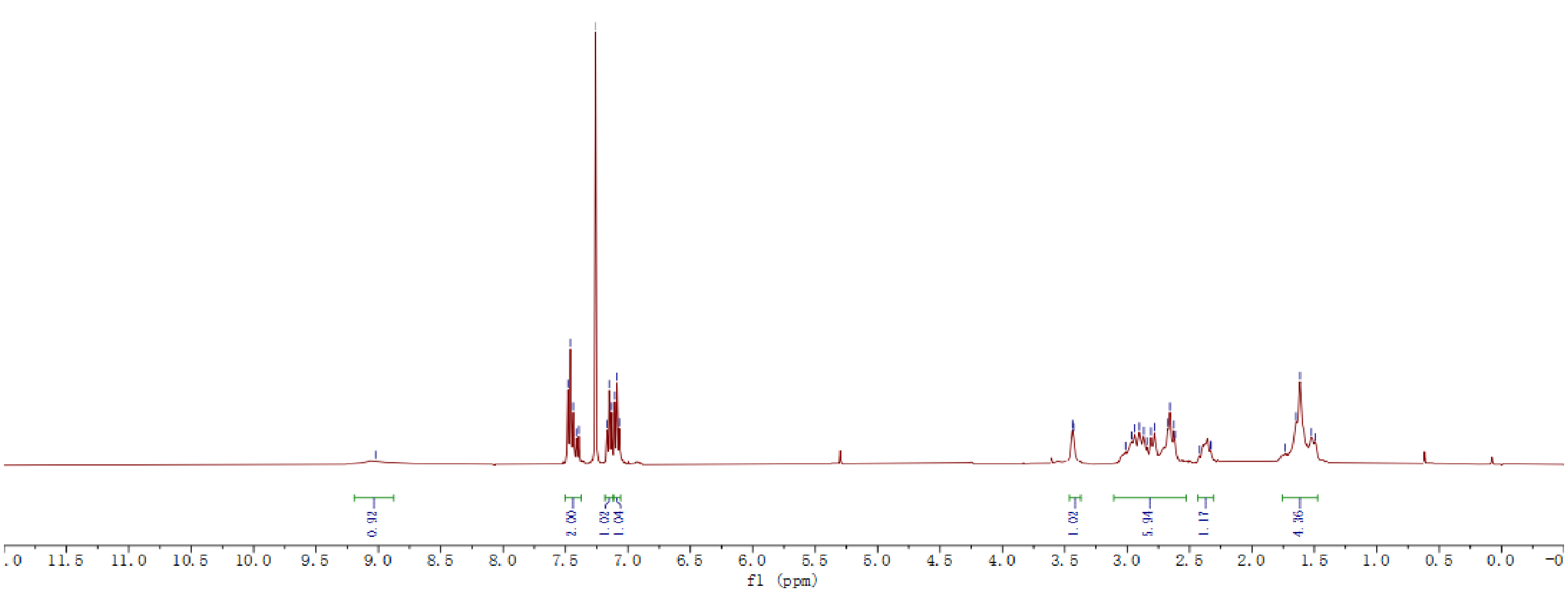

$^1H$ NMR (400 MHz, DMSO-$d_6$) (1*R*,12b*S*)-aminoindoloquinolizidine (**11**)

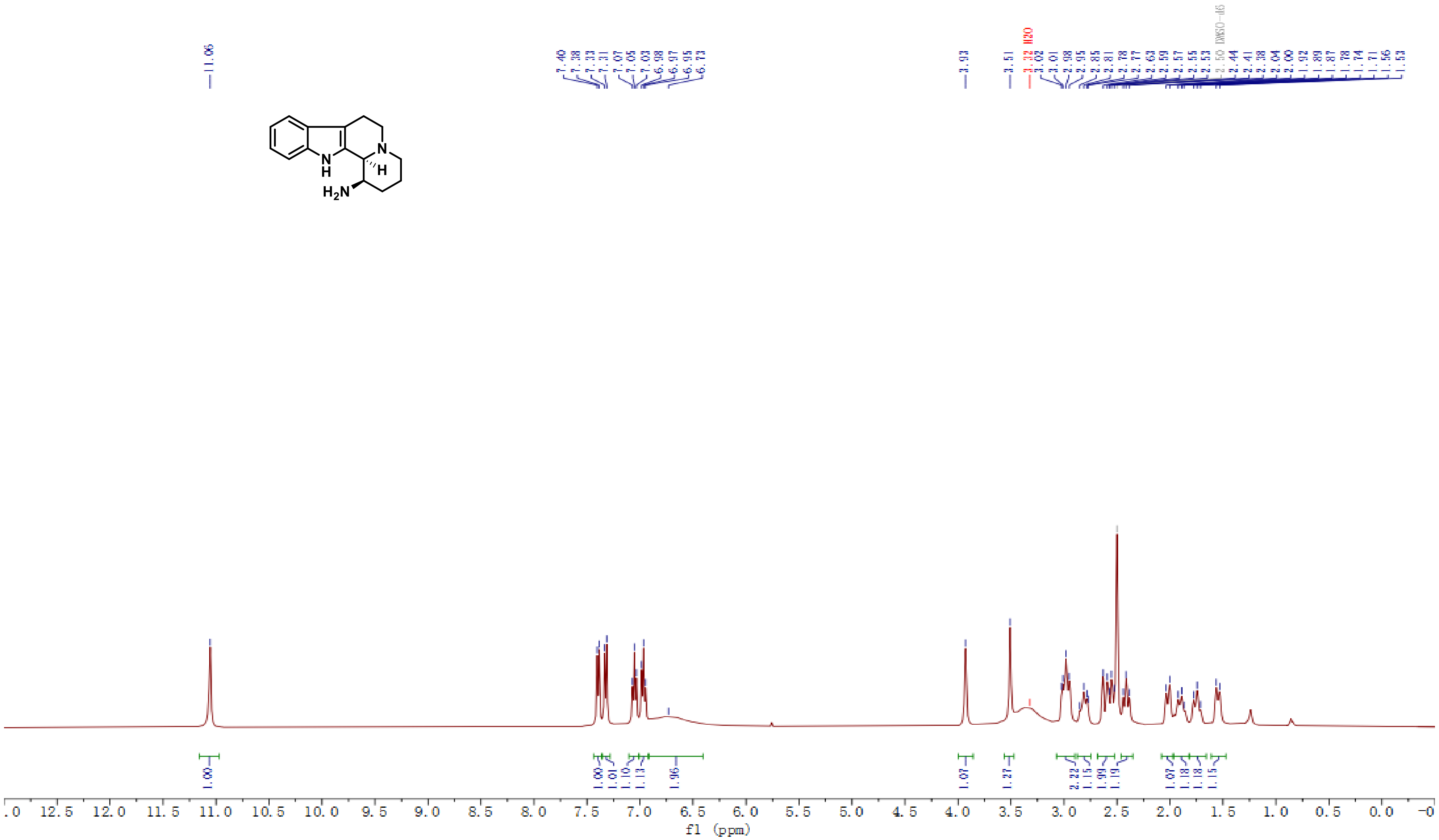

$^{13}$C NMR (101 MHz, DMSO-$d_6$) (1$R$,12b$S$)-aminoindoloquinolizidine (**11**)

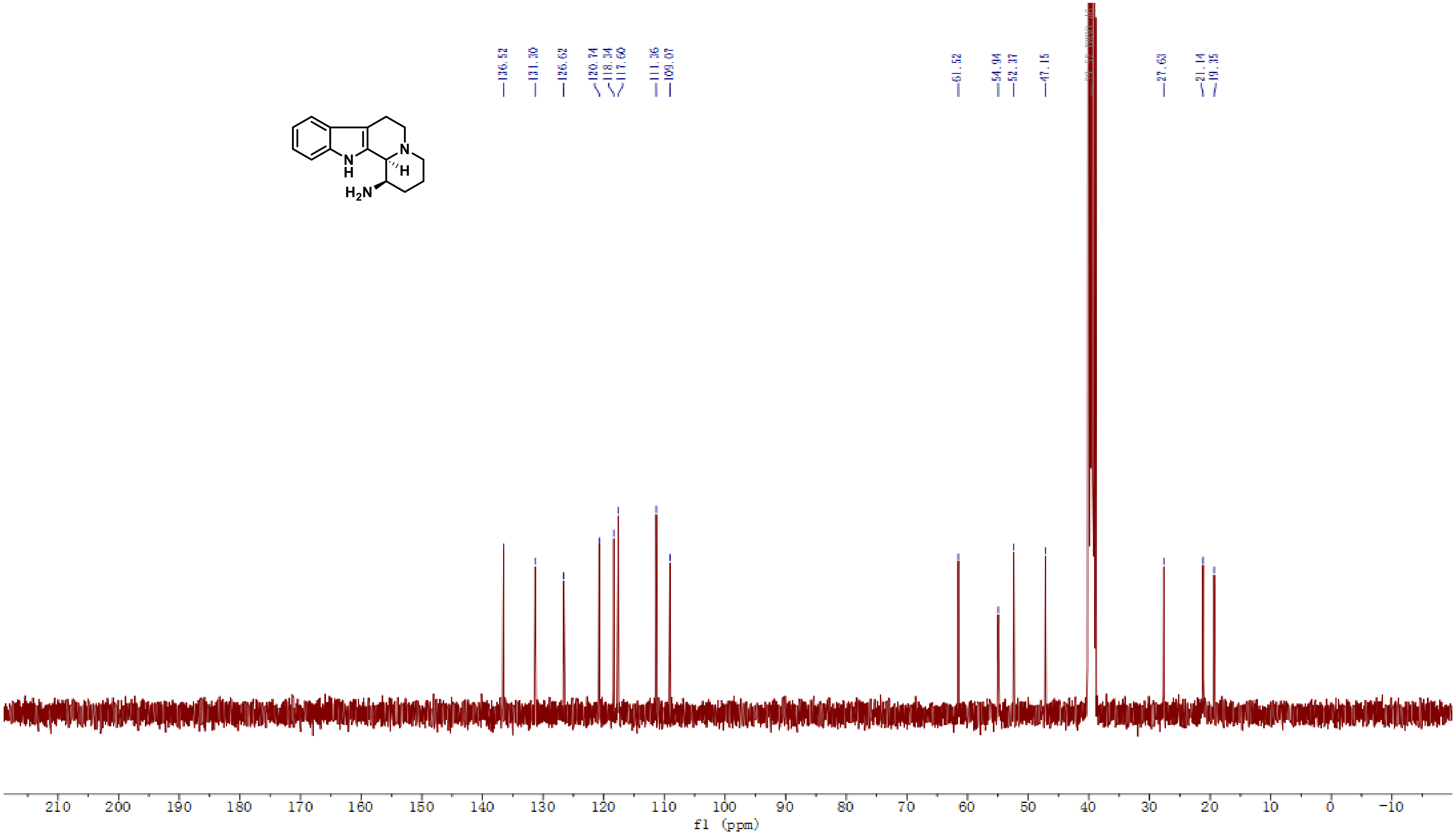

[1]H NMR (400 MHz, $CDCl_3$) (*R*)-5-(6-bromo-9*H*-pyrido[3,4-*b*]indol-1-yl)pyrrolidin-2-one (**12**)

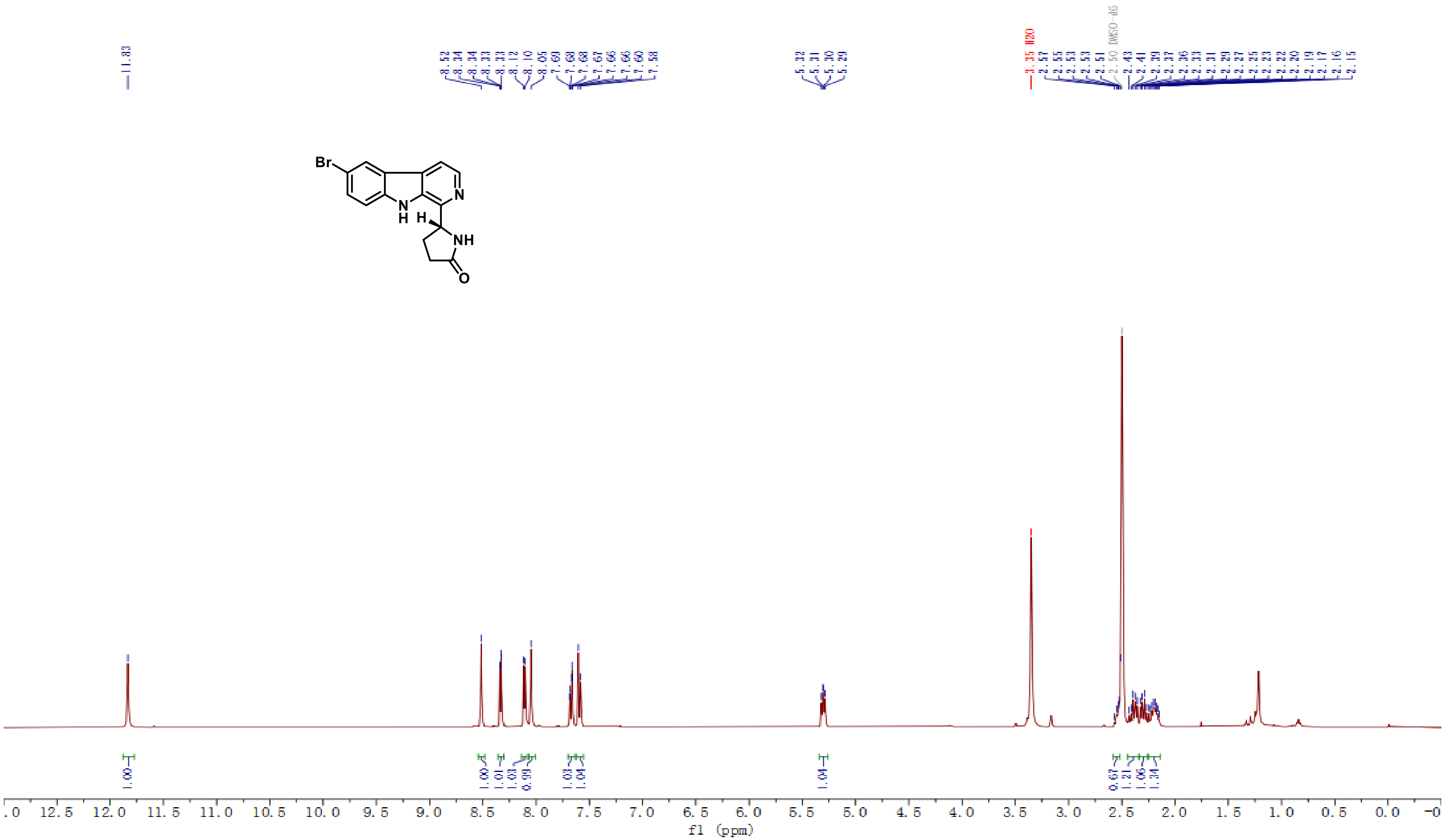

$^{13}$C NMR (101 MHz, $CDCl_3$) (*R*)-5-(6-bromo-9*H*-pyrido[3,4-*b*]indol-1-yl)pyrrolidin-2-one (**12**)

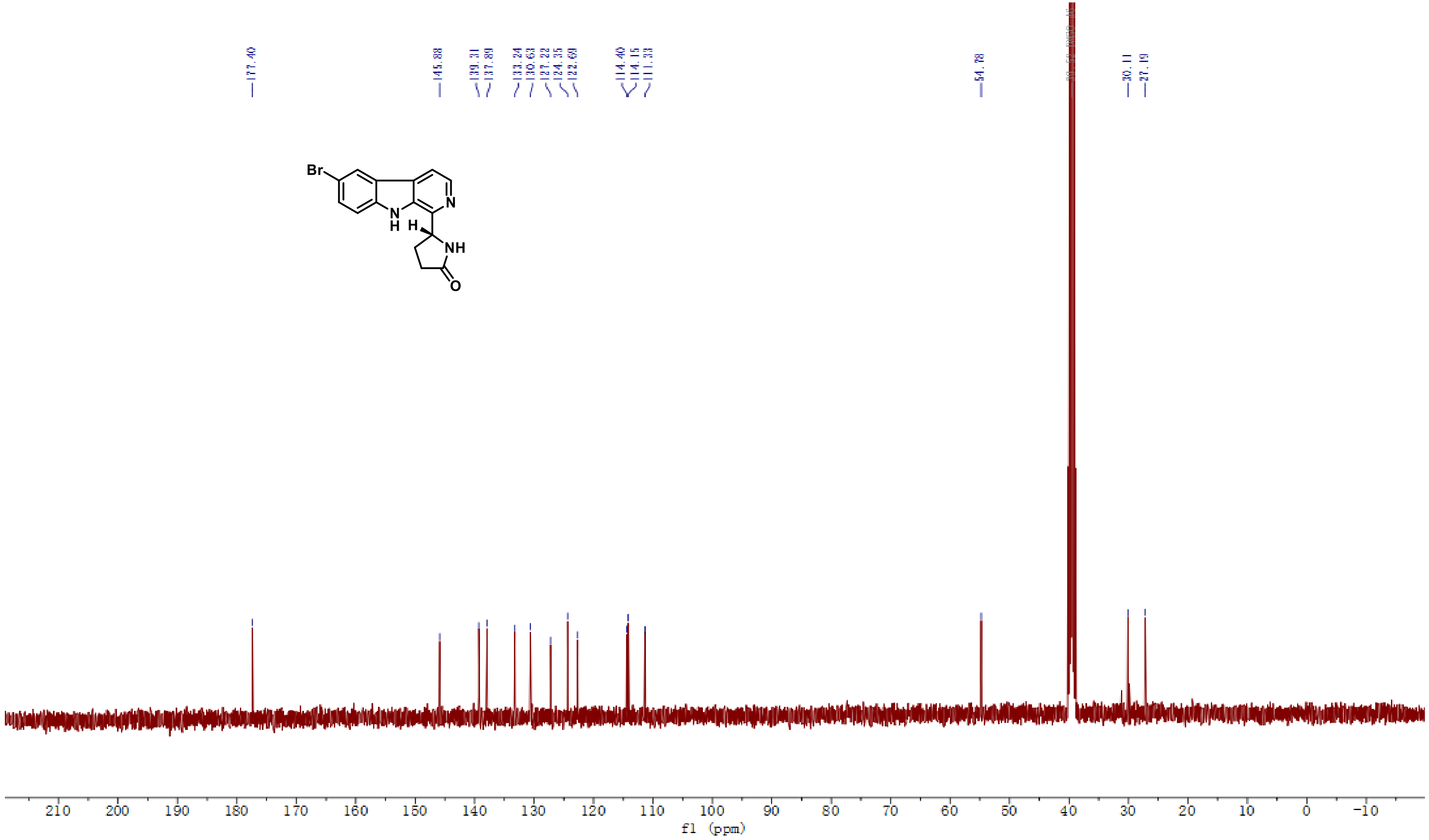

$^{1}H$ NMR (400 MHz, $CDCl_3$) (*R*)-5-((*S*)-6-bromo-2-methyl-2,3,4,9-tetrahydro-1*H*-pyrido[3,4-*b*]indol-1-yl)pyrrolidin-2-one (**14**)

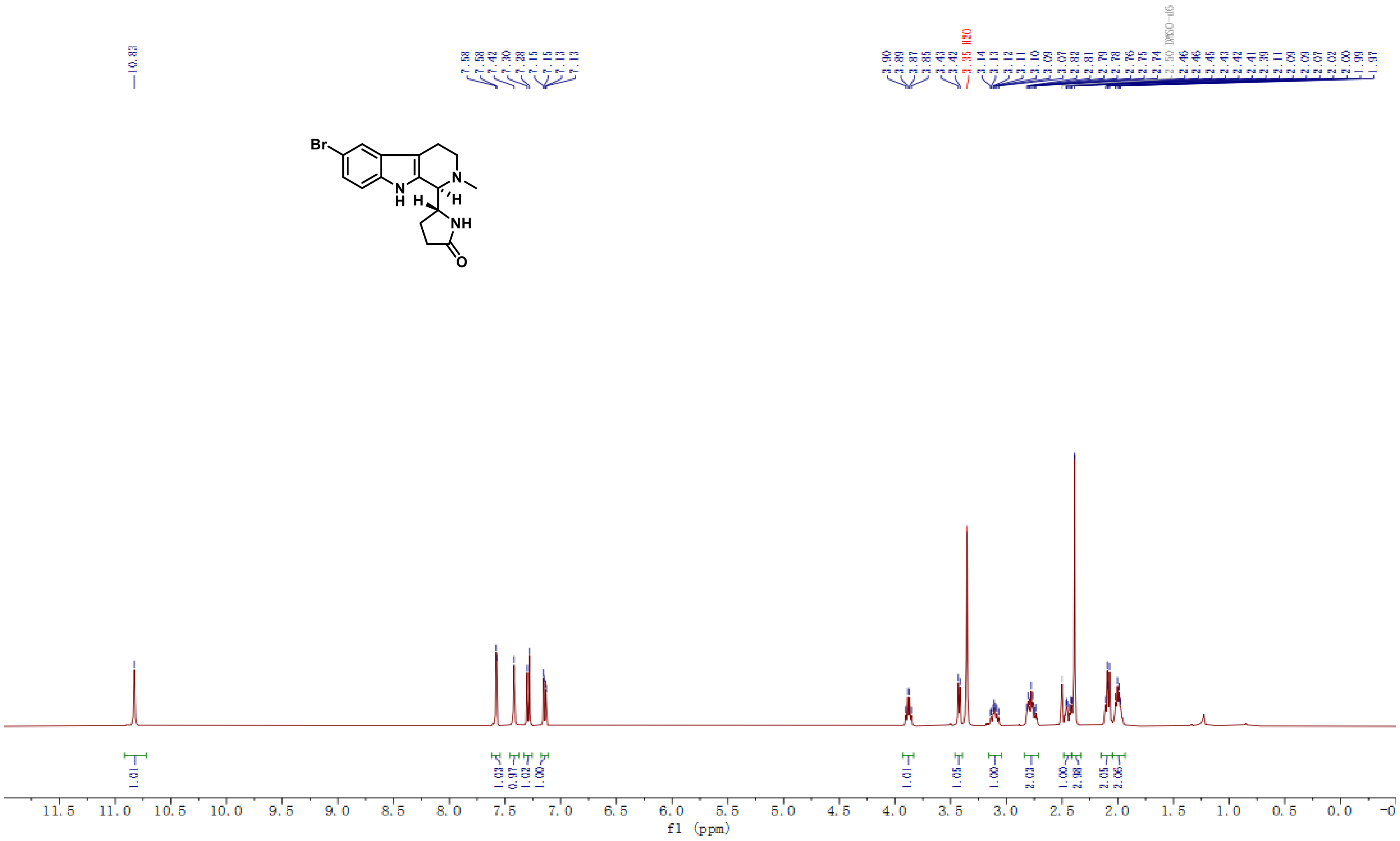

$^{13}$C NMR (101 MHz, $CDCl_3$) (*R*)-5-((*S*)-6-bromo-2-methyl-2,3,4,9-tetrahydro-1*H*-pyrido[3,4-*b*]indol-1-yl)pyrrolidin-2-one (**14**)

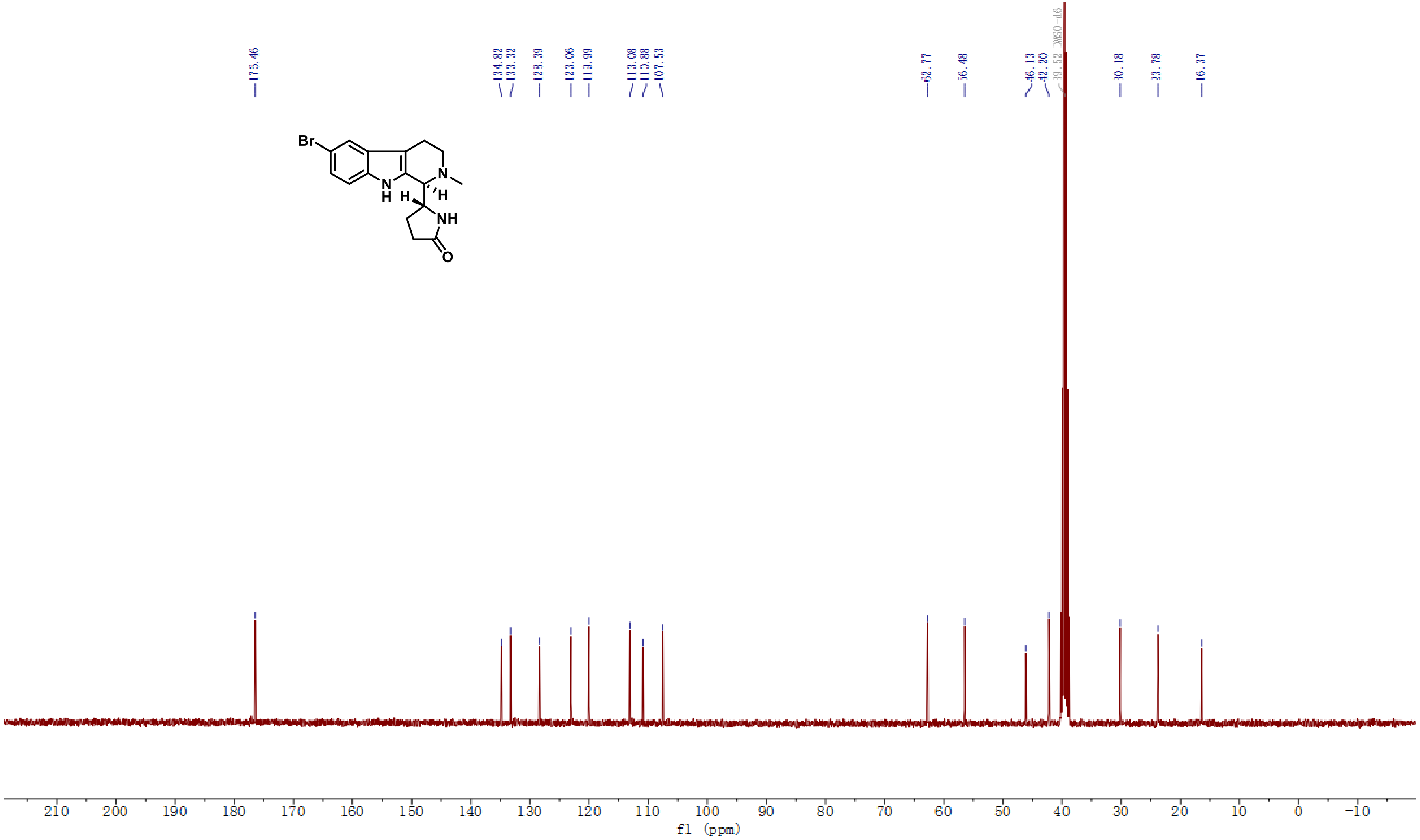

[1]H NMR (400 MHz, $C_6D_6$) **(+)-woodinine** (**15**)

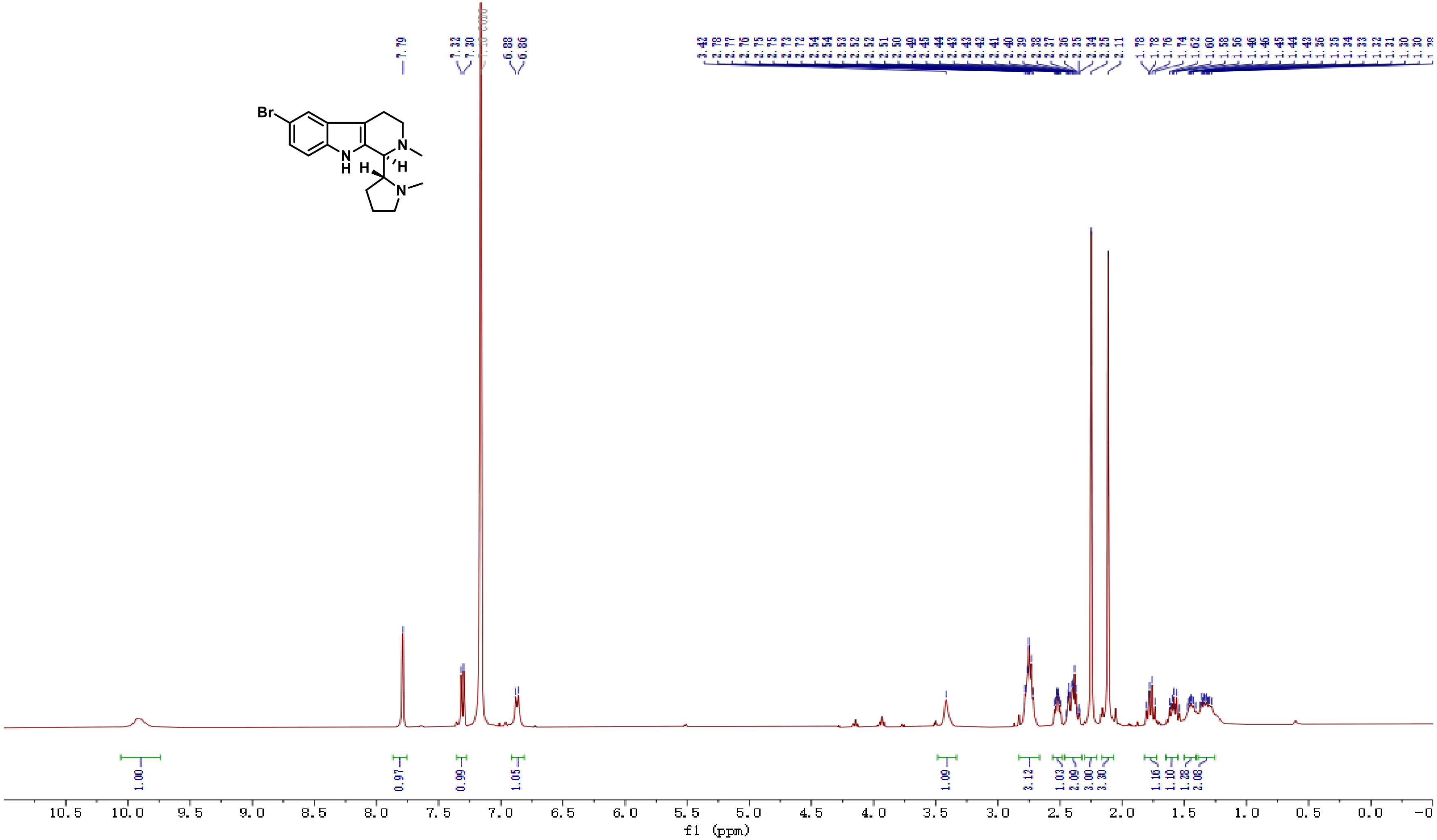

$^{13}C$ NMR (101 MHz, $C_6D_6$) **(+)-woodinine** (**15**)

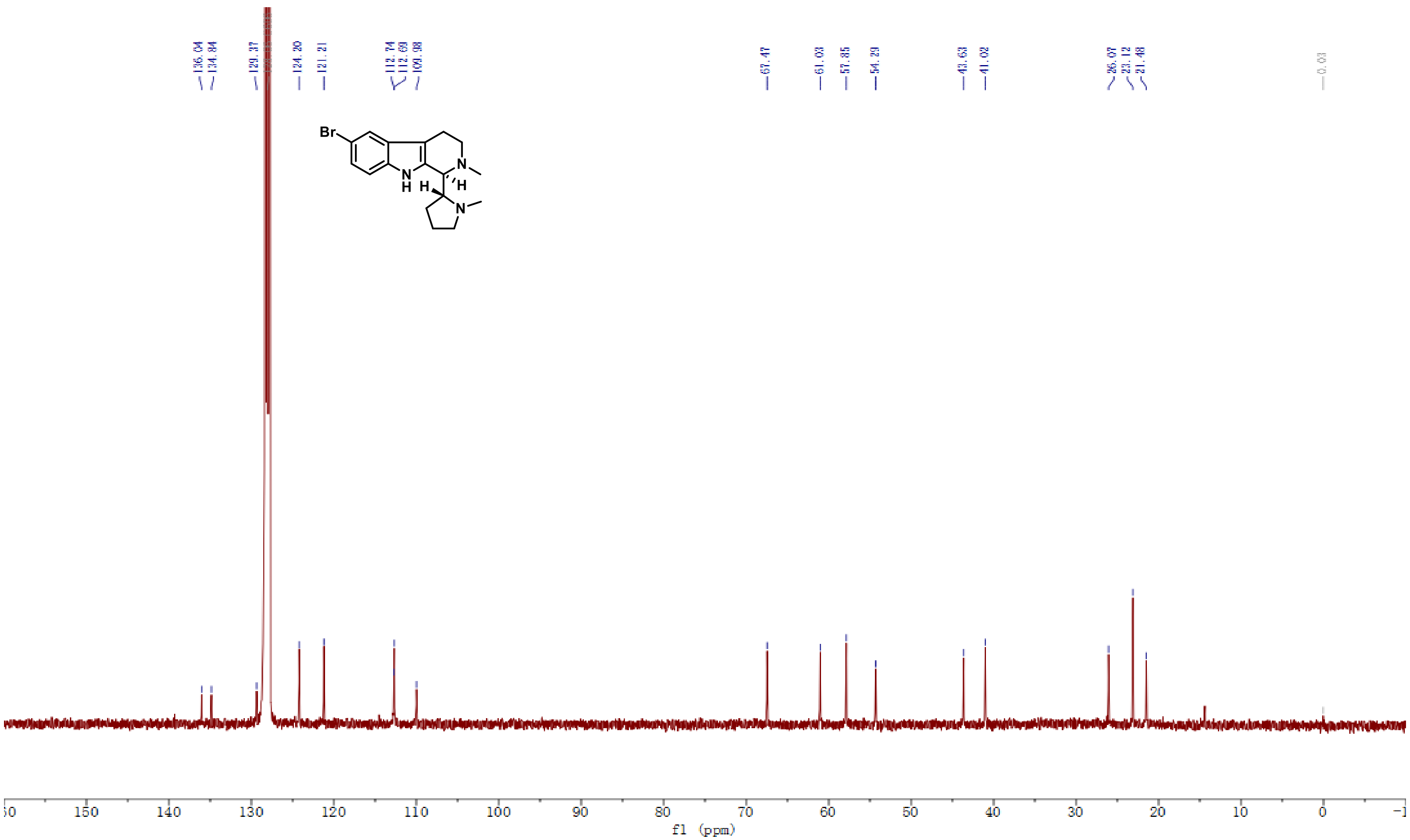

<sup></sup>

[1]H NMR (400 MHz, $CDCl_3$) ethyl 2-((2,2,6,6-tetramethylpiperidin-1-yl)oxy)acetate (**16**)

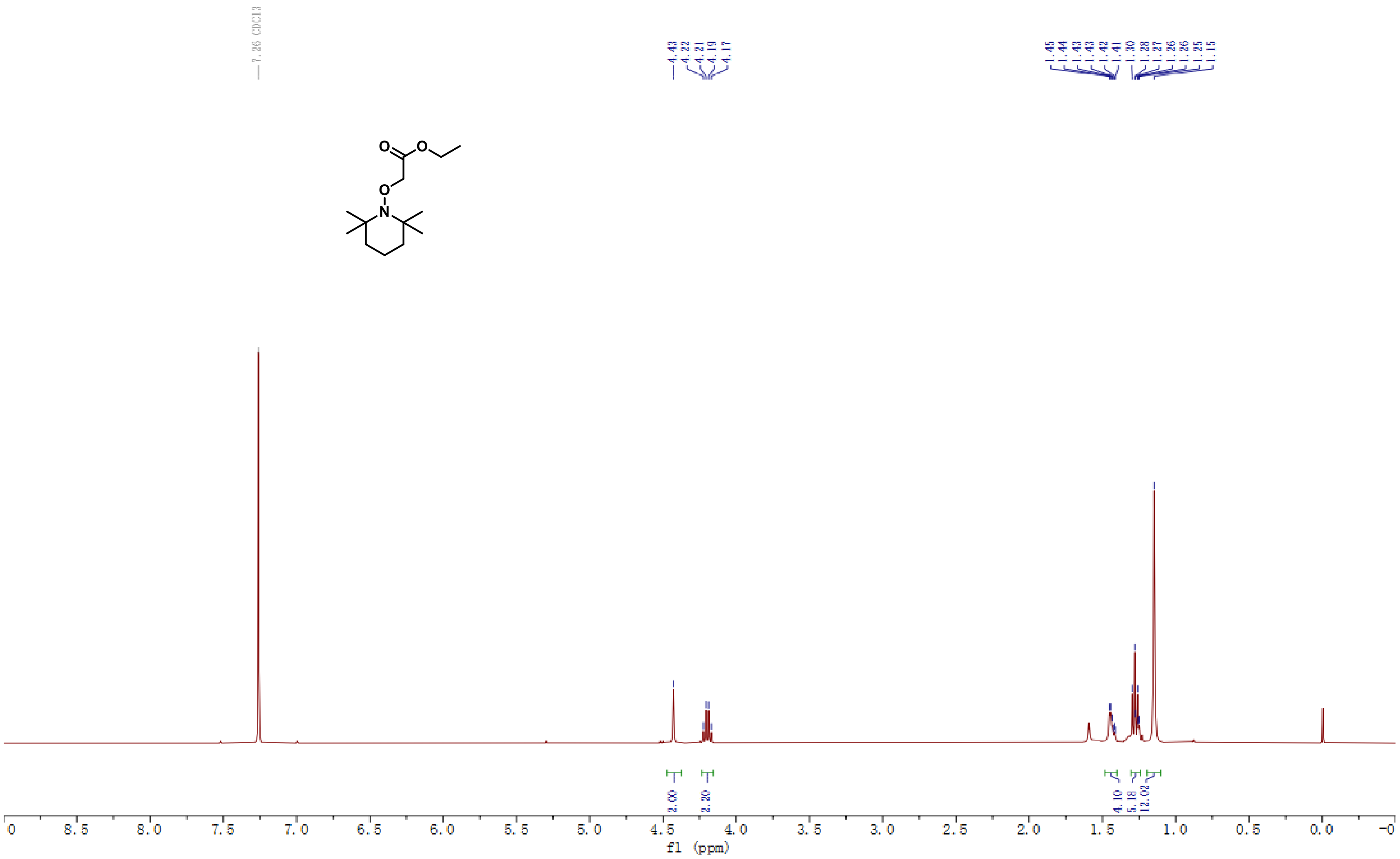

Supplement: Supplementary file 1 — Supporting Information [file ADVS-11-2402272-s001.pdf]
